# Ancient DNA connects large-scale migration with the spread of Slavs

**DOI:** 10.1038/s41586-025-09437-6

**Published:** 2025-09-03

**Authors:** Joscha Gretzinger, Felix Biermann, Hellen Mager, Benedict King, Denisa Zlámalová, Luca Traverso, Guido A. Gnecchi Ruscone, Sanni Peltola, Elina Salmela, Gunnar U. Neumann, Rita Radzeviciute, Pavlína Ingrová, Radosław Liwoch, Iwona Wronka, Radomir Jurić, Anna Hyrchała, Barbara Niezabitowska-Wiśniewska, Bartłomiej Bartecki, Beata Borowska, Tomasz Dzieńkowski, Marcin Wołoszyn, Michał Wojenka, Jarosław Wilczyński, Małgorzata Kot, Eric Müller, Jörg Orschiedt, Gunita Zariņa, Päivi Onkamo, Falko Daim, Arnold Muhl, Ralf Schwarz, Marek Majer, Michael McCormick, Jan Květina, Tivadar Vida, Patrick J. Geary, Jiří Macháček, Mario Šlaus, Harald Meller, Walter Pohl, Zuzana Hofmanová, Johannes Krause

**Affiliations:** 1 Department of Archaeogenetics, Max Planck Institute for Evolutionary Anthropology, Leipzig, Germany; 2 Institute of History, University of Szczecin, Szczecin, Poland; 3 State Office for Heritage Management and Archaeology Saxony-Anhalt and State Museum of Prehistory, Halle, Germany; 4 Department of Linguistic and Cultural Evolution, Max Planck Institute for Evolutionary Anthropology, Leipzig, Germany; 5 Department of Archaeology and Museology, Masaryk University, Brno, Czech Republic; 6 Department of Biology, University of Turku, Turku, Finland; 7 Organismal and Evolutionary Biology Research Programme, University of Helsinki, Helsinki, Finland; 8 Laboratory of Anthropology, Institute of Zoology and Biomedical Research, Jagiellonian University, Krakow, Poland; 9 Matica Hrvatska Zadar, Zadar, Croatia; 10 Doctoral School of Humanities and Art, Maria Curie-Skłodowska University, Lublin, Poland; 11 Institute of Archaeology, Maria Curie-Skłodowska University, Lublin, Poland; 12 Rev. Stanisław Staszic Museum, Hrubieszów, Poland; 13 Department of Anthropology, University of Łódź, Łódź, Poland; 14 Leibniz Institute for the History and Culture of Eastern Europe, Leipzig, Germany; 15 Institute of Archaeology, University of Rzeszów, Rzeszów, Poland; 16 Institute of Archeology, Jagiellonian University, Krakow, Poland; 17 Institute of Systematics and Evolution of Animals, Polish Academy of Sciences, Krakow, Poland; 18 Faculty of Archaeology, University of Warsaw, Warsaw, Poland; 19 State Archaeology Department of Schleswig-Holstein, Schleswig, Germany; 20 Institute Prehistoric Archaeology, Free University Berlin, Berlin, Germany; 21 Institute of Latvian History, University of Latvia, Riga, Latvia; 22 Department for Prehistory and Historical Archaeology, University of Vienna, Vienna, Austria; 23 Department of Slavic Philology, University of Łódź, Łódź, Poland; 24 Max Planck-Harvard Research Center for the Archaeoscience of the Ancient Mediterranean, Initiative for the Science of the Human Past at Harvard, Department of History, Harvard University, Cambridge, MA USA; 25 Institute of History, Czech Academy of Sciences, Prague, Czech Republic; 26 Institute of Archaeological Sciences, Eötvös Loránd University, Budapest, Hungary; 27 Institute for Advanced Study, Princeton, NJ USA; 28 Croatian Academy of Sciences and Arts, Zagreb, Croatia; 29 Institute for Austrian Historical Research, University of Vienna, Vienna, Austria; 30 Institute for Medieval Research, Austrian Academy of Sciences, Vienna, Austria

**Keywords:** Genetics, Archaeology, Population genetics

## Abstract

The second half of the first millennium ce in Central and Eastern Europe was accompanied by fundamental cultural and political transformations. This period of change is commonly associated with the appearance of the Slavs, which is supported by textual evidence^1,2^ and coincides with the emergence of similar archaeological horizons^3–6^. However, so far there has been no consensus on whether this archaeological horizon spread by migration, Slavicisation or a combination of both. Genetic data remain sparse, especially owing to the widespread practice of cremation in the early phase of the Slavic settlement. Here we present genome-wide data from 555 ancient individuals, including 359 samples from Slavic contexts from as early as the seventh century ce. Our data demonstrate large-scale population movement from Eastern Europe during the sixth to eighth centuries, replacing more than 80% of the local gene pool in Eastern Germany, Poland and Croatia. Yet, we also show substantial regional heterogeneity as well as a lack of sex-biased admixture, indicating varying degrees of cultural assimilation of the autochthonous populations. Comparing archaeological and genetic evidence, we find that the change in ancestry in Eastern Germany coincided with a change in social organization, characterized by an intensification of inter- and intra-site genetic relatedness and patrilocality. On the European scale, it appears plausible that the changes in material culture and language between the sixth and eighth centuries were connected to these large-scale population movements.

## Abstract (web summary)

Analyses of ancient human DNA show that cultural and political transformations in Central Europe during the second half of the first millennium ce were associated with movements of Slavic populations into Germany, Poland and Croatia.

## Main

This study combines a temporal transect of the Elbe-Saale region in Eastern Germany with the wide-angle view of large-scale demographic and cultural transformations that emerged similarly in other Eastern and Central European regions. Traditionally, on the basis of historical writings, this transformation is attributed to the emigration of ‘Germanic’ peoples from East-Central Europe and the arrival of a new population that contemporaries described as ‘Slavs’. These newcomers emerged after the dissolution of the Western Roman empire and mark the transition between the Migration Period (MP, late fourth to late sixth century) and the Slavic Period (SP, from the sixth or seventh century onwards).

At least since the first century bce, the lands between the Rhine and Vistula River were settled by numerous peoples and tribes for whom Roman observers used the umbrella term ‘Germani’^7^. These Germanic peoples were in contact with the Roman Empire west of the Rhine and south of the Danube, and since the late second century ce increasingly raided Roman provinces^8,9^. In the MP, many of them left and settled on Roman territory^7,8–10^, among them Vandals, Goths, Franks and Longobards^11,12^. The Thuringians stayed and established a kingdom, which included the Elbe-Saale region^13,14^. After the Franks subdued this kingdom^10,15^ in the 530s, the population declined, while some cemeteries continued^14,16^. During the seventh century, Slavs are first mentioned east of the Saale, but they soon expanded westward^17^, forming a contact zone between Slavic- and Germanic-speaking groups.

**Extended Data Fig. 1:**
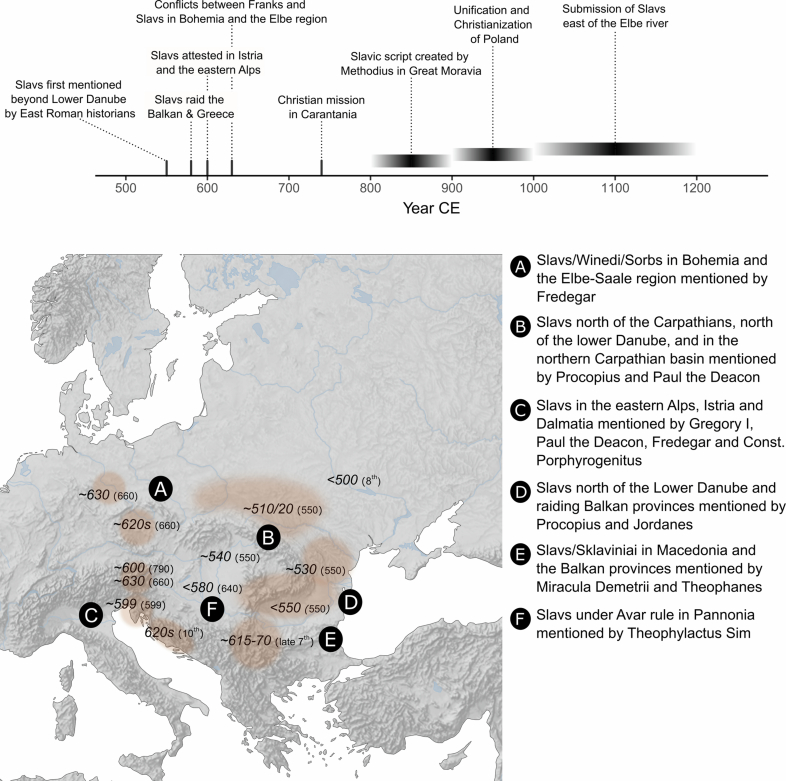
Historical overview of Slavs in Europe. The timeline lists major historical events associated with the Slavs in Central Europe. The map features schematized historical attestations for the appearance of Slavs (Sklavenoi – Slavi – Winedi). Italic numbers indicate the date of the attested event, with the respective report date in brackets. Made with Natural Earth.

The term Slavs first appears as an ethnonym in the course of the sixth century in Constantinople and later in the west (Box 1 and Supplementary Note 1.1). Written sources locate them initially north of the Lower Danube, and later in the Carpathian Basin, the Balkans and the Eastern Alps^1,2^ (Extended Data Fig. 1). Many came under the rule of the Avar steppe empire along the Middle Danube (567 ce to around 800 ce). In the seventh century, there is evidence for the presence of Slavs in much of East-Central and Southeastern Europe. Where Slavs lived, Roman, Germanic and other pre-Slavic infrastructures were usually replaced by rather simple ways of life, archaeologically characterized by small settlements of pit houses, cremation burials, handmade, undecorated pottery and modest, low-metal material culture^18^, known as the Prague-Korchak group^3,4^. (Supplementary Note 1.2). More complex social systems and regional rulership developed later in the contact zones with Byzantium and the Christian west.

The similarity of early Slavic cultures was often attributed to a swift spread of Slavs from Northeast of the Carpathians, although debates continue, not only about their geographical origin (Supplementary Note 1,1). In Poland^19^, the non-native (allochthonist) view assumes Slavic origin from Ukraine–Belarus^18^, whereas the native (autochthonist) concept asserts that their ancestors inhabited Polish territory since the Bronze Age. Some scholars doubt Slavic expansion by migrations and assume that there was ‘Slavicisation’ of existing populations^5,18,20–25^ (Supplementary Note 1,2). Previous modern^26^ and ancient DNA studies have supported gene flow into the Northern Balkans^27^ and the Russian Volga-Oka region^28^, but also argued for population continuity in Poland^29^, so that the scale and sequence of these movements and their association with ‘Slavic’ material culture has remained unclear. Eventually, this cultural transformation led to the replacement of Germanic and other languages in East-Central and Southeastern Europe and the introduction of Slavic languages, which today represent the largest linguistic group in Europe^30^. Yet, this presumed joint spread of language and material culture is difficult to trace, given that the first longer texts in Slavic were written in the late ninth century^5,31^.

Together with previously published data from Roman and early medieval Europe^27–29^, the newly analysed ancient DNA from the Elbe-Saale region and complementary data transects from the Northwestern Balkans, Poland, Latvia and Ukraine identify large-scale population movement and a major demographic shift. This can be linked to historical information about the spread of Slavic groups in the sixth to eighth centuries and provides a plausible vector for the spread of Slavic languages across much of Eastern Europe^21,23,32^.

## New ancient DNA data

**Fig. 1:**
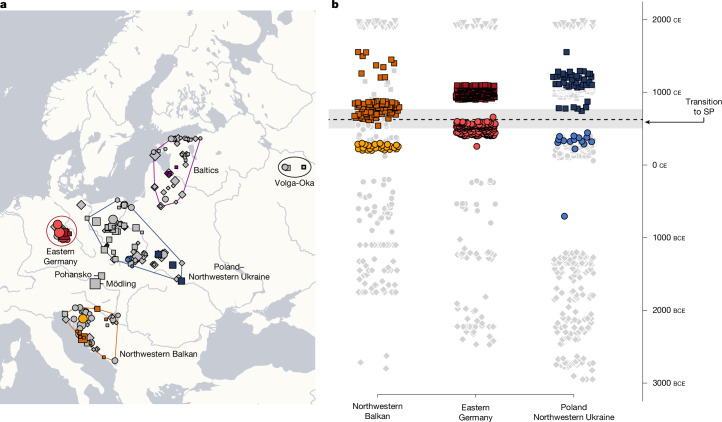
Geographic and temporal overview of ancient individuals. **a**, We assign newly reported (*n* = 550) and published genomic data (*n* = 723) to three study transects: the Northwestern Balkans (*n* = 301) (orange to yellow), Elbe-Saale region (Eastern Germany) (*n* = 483) (light and dark red), and Poland–Northwestern Ukraine (*n* = 489) (light and dark blue). We further analyse newly reported and published data from the Baltics as well as the Russian Volga-Oka region as reference, in total covering 1,840 ancient individuals. The size of the symbols corresponds to the number of individuals per site. Made with Natural Earth. **b**, Chronological sequence of 1,243 newly reported (coloured) and previously published (grey) samples analysed in this study. We selected samples from all three study regions that predate and postdate the transition to the SP. Circles, MP samples; squares, SP samples; diamonds, Bronze Age samples; triangles, present-day samples.

**Extended Data Fig. 2:**
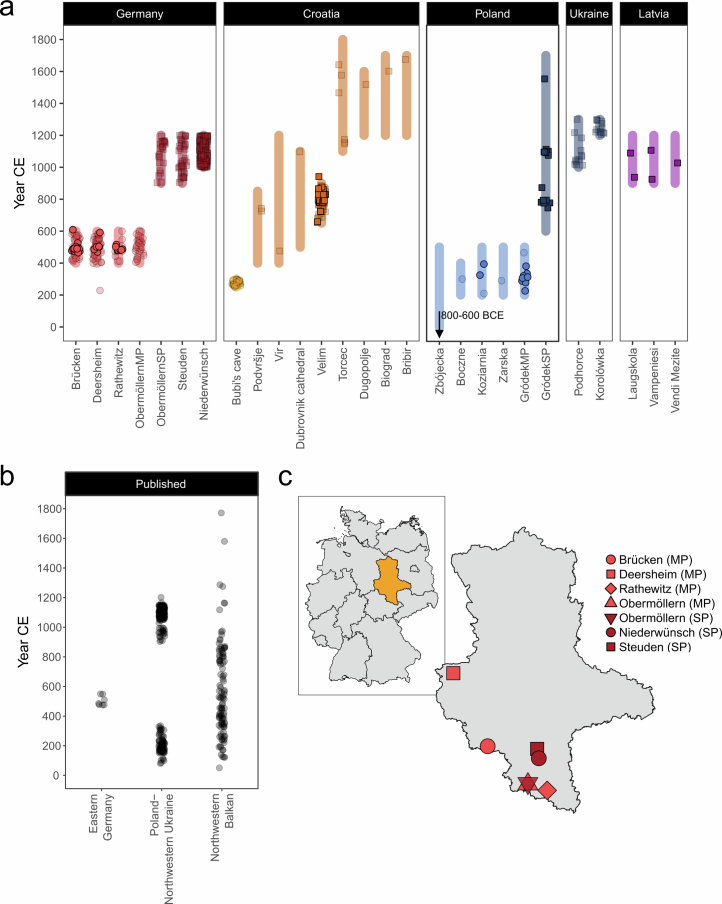
Temporal and spatial distribution of data analysed in this study. **a**, Temporal distribution of samples analysed in this study, with site occupancy ranges indicated by bars. Non-transparent symbols indicate radiocarbon-dated samples; transparent symbols are scattered uniformly along site occupancy ranges. **b**, Temporal distribution of published samples analysed in this study. **c**, Spatial distribution of Eastern German sites analysed in this study. Made with Natural Earth.

We selected skeletal remains from 591 ancient individuals from 26 different sites from Central and Eastern Europe (Supplementary Tables 1 and 2), creating, in combination with previously published data, a dense sampling transect for three regions: (1) Elbe-Saale Region in Eastern Germany as the main study area; (2) the Northwestern Balkans; and (3) Poland–Northwestern Ukraine (Extended Data Fig. 2 and Supplementary Tables 7–10). Complementary to these three transects, we generated new data and collected published data from the Baltics and Northwestern Russia to form a reference transect in the east. After hybridization DNA capture and quality filtering (Methods), genome-wide data for 555 unique individuals with a median coverage of 538k single nucleotide polymorphisms (SNPs) (on 1240k data) were available for analysis, including 359 ancient individuals from the SP, as well as 205 individuals predating the cultural transformations connected to the emergence of the Slavs (Fig. 1 and Supplementary Table 1). We analyse the ancient genome-wide data (Supplementary Table 11) together with an extended dataset of more than 11,500 present-day Europeans (Supplementary Table 3), covering all major Slavic-speaking groups, including data from more than 600 individuals belonging to the Sorbian minority in Eastern Germany^33^.

## Genetic shifts in Central Europe

**Fig. 2:**
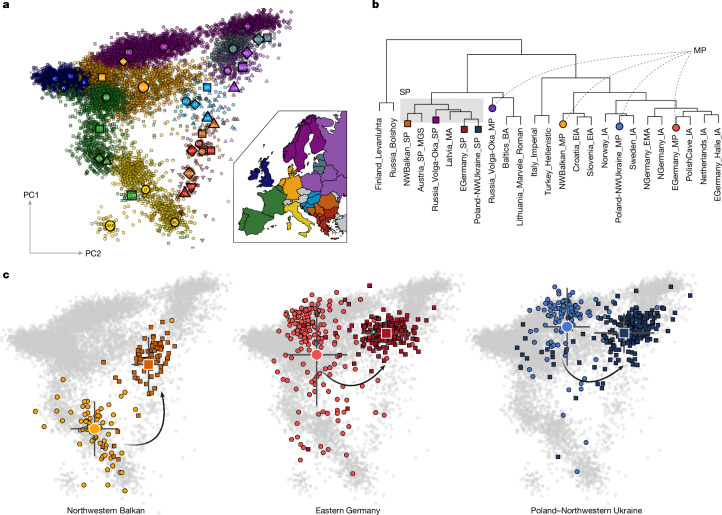
Population structure before and after the MP–SP transition. **a**, The reference PCA computed using 10,528 present-day Europeans. Large symbols indicate the mean PC1 and PC2 coordinates of the respective population. The map to the right visualizes the geographical origin of the samples. Made with Natural Earth. **b**, Hierarchical clustering (ward.D2) of Mahalanobis distances between selected ancient groups based on the first ten principal components. Circles, Roman Period and MP samples; squares, SP samples. BA, Bronze Age; EIA, Early Iron Age; EMA, Early Middle Ages; IA, Iron Age; MA, Middle Ages; MGS, Mödling; MP, migration period; SP, Slavic period. (See Methods section ‘Naming’.) **c**, Ancient genome-wide data (*n* = 835) from the Northwestern Balkans, Eastern Germany and Poland–Northwestern Ukraine projected onto the modern reference PCA. Circles, Roman Period and MP samples; squares, SP samples. Large symbols denote the mean PC1 and PC2 coordinates. Error bars indicate 2 × s.d.

**Extended Data Fig. 3:**
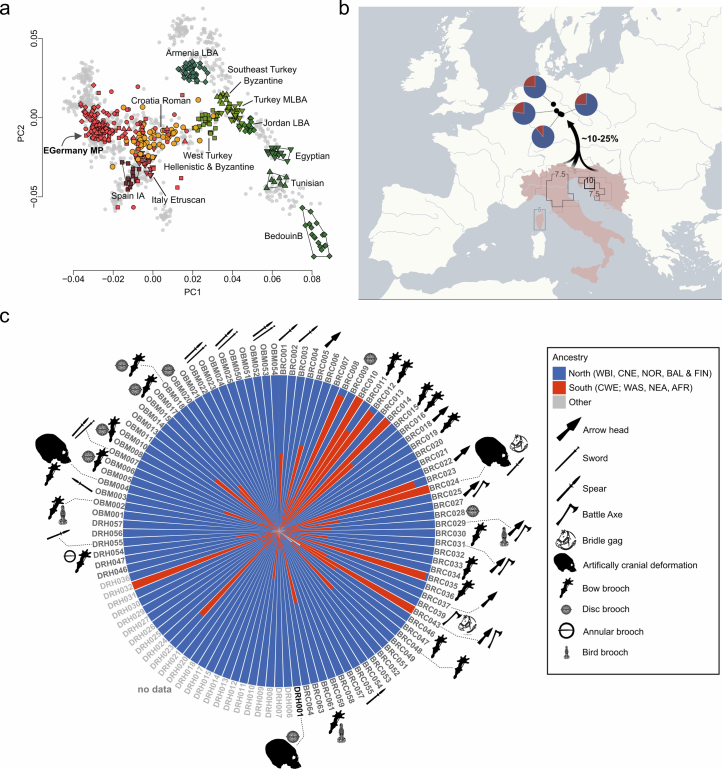
Genetic affinities of the MIgration Period BRC, DRH, OBM and RTW populations from Eastern Germany. **a**, West Eurasian PCA (present-day individuals are depicted as grey background points) with projection of BRC (n = 56), DRH (n = 49), OBM (n = 30), and RTW (n = 15) and other relevant Iron Age, Roman Period, and Late Antique populations (coloured symbols). **b**, Schematic overview of the geographic origin of ancient individuals with South European ancestry from Eastern Germany. The contours indicate the averaged maximum probability at search time 200 CE (multiplied by 100) for 10 individuals from BRC, DRH, OBM and RTW (denoting the mean prediction of the geographic regions where the ancestors of these individuals originated) as inferred using MOBEST. Results from two-way qpAdm analyses are shown as pie charts for each site. Made with Natural Earth. **c**, Schematic distribution of different artifact types among BRC, DRH, and OBM burials. The respective ancestry composition of each individual in respect to northern (WBI, CNE, NOR, BAL & FIN) and southern ancestry (CWE, WAS, NEA & AFR) (from supervised ADMIXTURE) is indicated as bar chart.

To visualize genome-wide ancestry diversity before and after the spread of Slavic groups, we performed principal component analysis (PCA) on 10,528 present-day Europeans and projected our newly reported and other relevant ancient genome-wide data onto their genetic variation (Fig. 2). When comparing the SP samples to earlier and present-day data from our three study regions, we observe that the genetic composition within the transects changed markedly between about 600 and 800 ce. In general, the Roman and MP samples that predate the arrival of Slavic groups show high genetic heterogeneity in PCA space, with most samples from Germany and Poland^29,34^ clustering with present-day continental Northern German, Dutch and Scandinavian populations (Extended Data Fig. 3 and Supplementary Figs. 6, 7, 10 and 12), whereas the Roman and MP individuals from Croatia^34^ cluster with present-day Italian and Eastern Mediterranean populations (Fig. 2c and Supplementary Note 3).

In Eastern Germany and the Northwestern Balkans, most of the genetic diversity within the Roman and MP clusters follows a north–south cline along PC1. For the Northwestern Balkans, this heterogeneity has been attributed to increasing Eastern Mediterranean ancestry that arrived subsequently to the incorporation of the region into the Roman Empire^27,35^. More unexpectedly, we detect a high number of MP individuals with non-local, Southern European ancestry in the Elbe-Saale region of Eastern Germany, although this area was never part of the Roman Empire. Using qpAdm^36,37^, we measure on average between approximately 15% and 25% of Southern European ancestry in all 4 MP sites of the region (Extended Data Fig. 3).

Both PCA-based MOBEST^38^ analysis and *F*_4_ statistics indicate that this non-local ancestry was most probably derived from contemporaneous source populations in Italy and/or the Northern Balkan Peninsula (or other areas of the Roman Empire where people of this ancestry were located) (Extended Data Fig. 3 and Supplementary Figs. 3–5). Previous studies already identified mixed communities of northern and southern ancestry in Hungary and Northern Italy that were interpreted as amalgamation between Northern European newcomers and the local romanized population^39,40^. In contrast to these earlier results, we do not find evidence that the two different ancestries were correlated to differences in material culture (Supplementary Table 4). Applying a generalized linear model, we demonstrate that neither the presence of grave goods overall, nor certain types of artefacts (such as weapons or brooches) are significantly correlated with either PCA position or ADMIXTURE profiles (Supplementary Fig. 60). Instead, we find the only significant (*P* < 0.05) correlation between ancestry and material culture among the burial constructions, where we show that individuals buried in pits feature on average higher Northern European ancestry (Supplementary Fig. 60c). The spatial organization of the burials was also not determined by similarity in ancestry. Instead, we observe that individuals were buried close to their biological relatives, within small kin groups composed of individuals with Northern European, Southern European or mixed ancestry, reflecting a high degree of admixture between individuals with different ancestry backgrounds during the MP. Consequently, our data from Eastern Germany demonstrate that the cosmopolitan character of the Roman Empire not only affected the incorporated territories but also facilitated exchange and mobility along its borders and beyond into barbarian lands (Barbaricum), resulting in an unprecedented genetic diversity in Central Europe during^34^ and, in the case of Eastern Germany, even after its existence. Although the causes and circumstances of their movement to the Elbe-Saale region remain open for speculation, these newcomers apparently adapted the fashions and traditions of the local populations, resulting in a rather homogenous material culture within a group of individuals with diverse genetic backgrounds.

**Fig. 3:**
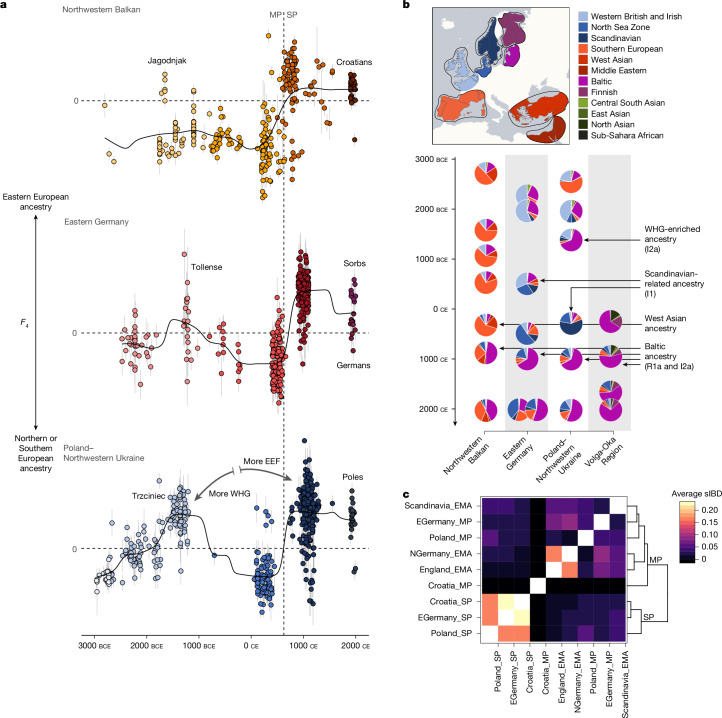
Changes in the gene pool of Central Europe. **a**, Trajectories of changes in affinity to Eastern Europe in ancient and present-day individuals from the Northwestern Balkans (*n* = 301), Eastern Germany (*n* = 483) and Poland–Northwestern Ukraine (*n* = 489), as measured using *F*_4_ statistics of the form *F*_4_(Han Chinese, test; Italy, Poland) (for the Northwestern Balkans) and *F*_4_(Han Chinese in Beijing, China (CHB), test; Denmark, Poland) (for Eastern Germany and Poland–Northwestern Ukraine). Error bars indicate 2 × s.d. **b**, Supervised ADMIXTURE modelling for the study regions. Ancient and present-day samples (*n* = 1,344) decomposed into 12 ancestral ancestry components. For different time periods, individual results were averaged and plotted according to their mean date. Influx of specific ancestries mentioned in the text are indicated using arrows. Relevant Y chromosome haplogroups associated with these autosomal ancestries are highlighted. Made with Natural Earth. **c**, Average sum of IBD segments (sIBD (in cM)) shared between nine ancient MP and SP groups. For all pairs of populations, the sum of IBD segments longer than 12 cM shared between members was calculated and normalized to the total number of pairs. The sums are depicted as symmetrical matrix. Hierarchical cluster analysis applying Ward’s minimum variance method to the columns is added as a dendrogram.

However, this diversity had collapsed in the subsequent SP (Supplementary Note 6). In contrast to the preceding MP, the genetic profile of Eastern Germany during the SP has shifted considerably and clusters nearly exclusively with present-day Slavic-speaking populations (for example, Poles and Belarussians), indicative of a fundamental replacement of genetic ancestry (Fig. 2b,c). A similar pattern is seen in the Northwestern Balkans, Poland–Northwestern Ukraine as well as the Volga-Oka region in Russia^28^, illustrating that this influx of new genetic material was not limited to certain regions but affected wide areas of Central and Eastern Europe, consistent with the rather simple, very similar archaeological horizons observed during the SP (Supplementary Figs. 10–12). To formally test whether these patterns observed from PCA are consistent with gene-flow events from the east into our study regions, we used *F*-statistics to quantify genetic affinities of SP individuals to preceding MP and succeeding present-day groups (Fig. 3a–c and Supplementary Tables 17–19). The divergence between pre-Slavic and Slavic-associated groups is verified both in the distribution of genetic distances (*F*_ST_) (Supplementary Fig. 19) as well as shared alleles (*F*_4_) (Supplementary Figs. 17–20) (Supplementary Note 4.2). Both on the population and the individual scale, SP individuals from all three study regions uniformly show less genetic affinity to the preceding local populations than to ancient and present-day groups from Eastern Europe and Baltics (Supplementary Figs. 34–38 and Supplementary Notes 4.2 and 4.4.1).

## Spread of SP ancestry across Europe

**Extended Data Fig. 4:**
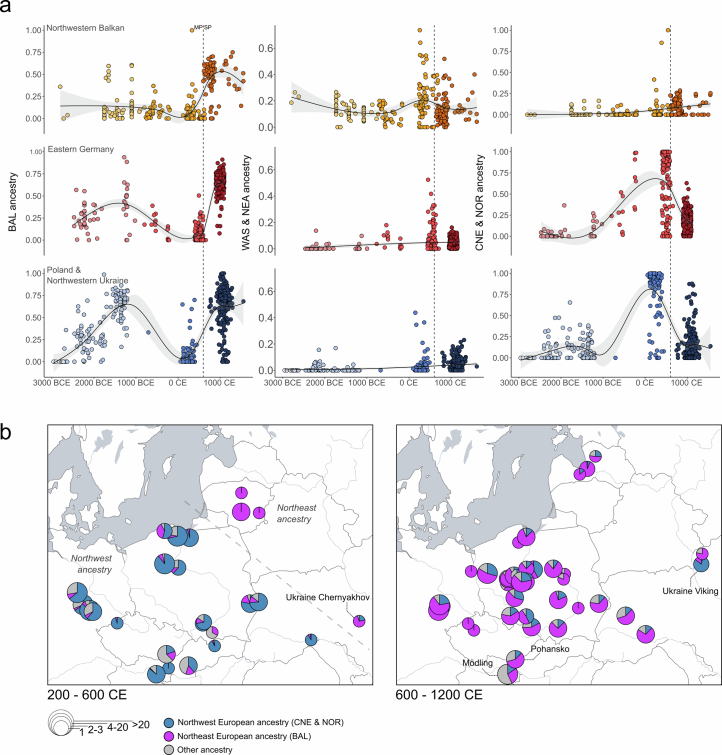
Changes in ancestry in Central Europe throughout the last 5,000 years. **a**, Trajectories of Northeastern European (BAL), East Mediterranean (WAS & NEA), and Northwest European (CNE & NOR) ancestry in ancient individuals from the Northwestern Balkans (n = 301), Eastern Germany (n = 483), and Poland-Northwestern Ukraine (n = 489), as measured using a supervised clustering approach implemented in ADMIXTURE. Error bands represent two standard errors. Made with Natural Earth. **b**, Averaged ancestry estimates from supervised ADMIXTURE analyses are shown as pie charts for sites in Central Europe before (n = 268) and after 600 CE (n = 975). Northwest European ancestry (CNE & NOR) is shown in blue, Northeastern European ancestry (BAL) in violet, and other ancestry in grey. The size of the pie charts correspond to the sample size per site.

Our results reveal that SP individuals display Baltic or Northeastern European-related ancestry that was previously absent in the three study regions. To quantitatively estimate this influx, we decomposed ancestral sources using a supervised clustering approach implemented in the software ADMIXTURE^41^. Specifically, we assembled modern populations into 12 metapopulations that serve as proxies for the source ancestries in Central Europe (Methods and Supplementary Note 4.1). Applying our ancestry decomposition to the ancient genome-wide data, we find that (despite differences in the local trajectories) Northeastern European ancestry (BAL, represented by present-day individuals from Belarus, Lithuania and Latvia) was either completely absent or only a minor ancestry component throughout most of prehistory in our study transects (Fig. 3d and Extended Data Fig. 3), accounting for 6 ± 2%, 5 ± 1% and 7 ± 2% of the total MP ancestry in the Northwestern Balkans, Eastern Germany and Poland–Northwestern Ukraine, respectively. However, consistent with PCA (Supplementary Fig. 12a–c) and *F*_4_ statistics (Fig. 3a–c), BAL ancestry increased after 600 ce and became the largest ancestry component in all three study regions, reaching 47 ± 2%, 65 ± 1% and 63 ± 2%, respectively, during the SP. Outside our three study transects, we furthermore identify a major surge of BAL ancestry (from 0% in the MP to approximately 27%) in the Avar-associated population of Mödling, Austria^42^, confirming an early arrival in the Pannonian Basin in the seventh century ce as reported by written sources, followed by substantial admixture with local groups (Extended Data Fig. 4). Only in Northwestern Russia do we detect a different trajectory: in the Volga-Oka area, the Slavic transition coincides with a significant decrease of BAL ancestry (from 65 ± 2% to 55 ± 7%), suggesting that the SP newcomers originated from a region further to the west of the Volga-Oka area where they incorporated additional ancestry not local to Eastern Europe.

**Extended Data Fig. 5:**
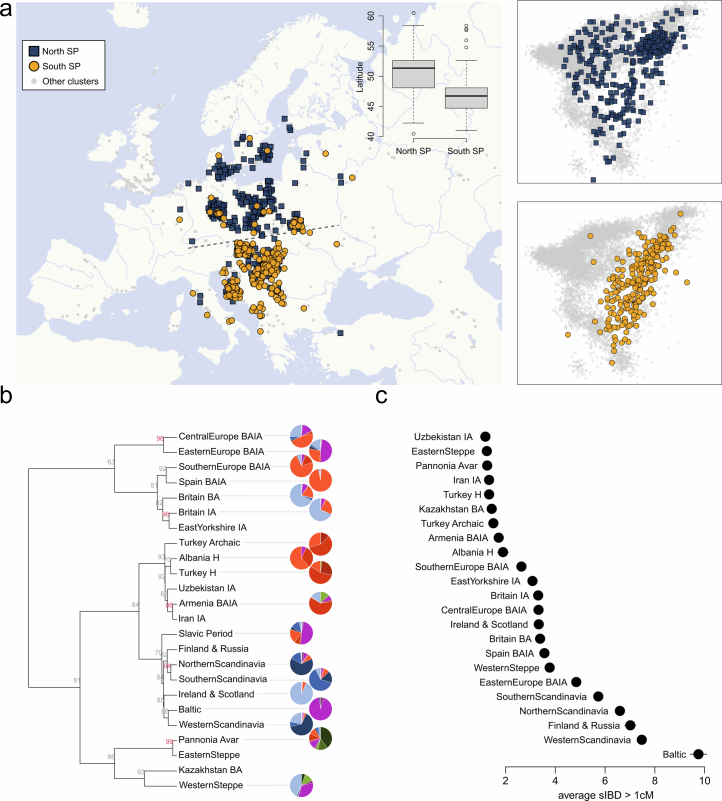
Genetic relatedness among a Slavic Period IBD-sharing community in Europe. **a**, Left: map highlighting the “Slavic Period” IBD-sharing community (1.1) (n = 602) identified applying a hierarchical cluster detection approach to a network constructed from pairwise IBD-sharing similarities between 2,657 ancient, post-Neolithic Eurasian individuals. A “Northern SP” sub-cluster (1.1.1) (n = 329) is highlighted in blue, a “Southern SP” sub-cluster (1.1.2) (n = 264) in orange. A boxplot comparison of Latitude coordinates in both clusters is indicated, showing significant differentiation among a North-South gradient (Welch Two Sample t-test; t = 16.331, df = 589.69, p < 2.2e-16). Right, ancient individuals from the Northern SP sub-cluster and Southern SP sub-cluster projected onto the modern European genetic variation. Made with Natural Earth. **b**, Hierarchical cluster analysis applying Ward’s minimum variance method to the normalized, average sIBD sharing (> 1 cM) between IBD clusters (n = 24) identified using community detection. The dendrogram and statistical support for the bifurcations from multiscale bootstrap resampling are shown. The ancestry compositions of selected relevant clusters (inferred using supervised ADMIXTURE) are indicated as pie charts. **c**, Average sIBD (> 1 cM) between the Slavic Period cluster and 23 major IBD clusters identified using community detection. Error bars indicate three standard errors.

The source for the incoming Northeastern European ancestry appears to be the same in all four regions. To showcase this shared descent, we applied ancIBD^43^ to identify segments that are identical by descent (IBD) that are shared between the MP and SP populations. We highlight that SP groups in Croatia, Eastern Germany and Poland–Ukraine share comparably large amounts of IBD with each other, despite the vast geographic distance between the three study regions, but share nearly no segments with the preceding populations (Fig. 3e and Supplementary Table 37). This IBD-sharing signal, including a large fraction of segments longer than 16 cM, clearly indicates that ancient individuals from Slavic-associated contexts descend from a common source population that migrated westwards and southwards at most a few generations earlier across Central Europe (Extended Data Figs. 5 and 10). Such evidence for large-scale population movement also explains the previously detected pattern of high levels of sharing of IBD between present-day pairs of individuals across Eastern Europe^44^ (Supplementary Fig. 56) and rejects the idea that this signal was caused predominantly by consistently low population densities^45^.

To obtain a finer-scale characterization of genetic ancestries across space and time, we applied a hierarchical cluster detection approach to a network of around 2,500 individuals constructed from these pairwise IBD-sharing similarities (Supplementary Table 38 and Supplementary Note 4.3.2). We identify a large IBD-sharing community that contains most of our new and published SP individuals as well as multiple other contemporary samples from Central and Southeastern Europe. Within this larger cluster, we identify two distinct sub-communities: one primarily includes SP individuals from north of the Carpathian Mountains, whereas the other comprises individuals buried further south. This separation may reflect two geographically diverging waves of expansion or different patterns of incorporation of the local populations (Extended Data Fig. 5). Yet, at least sporadic gene flow from Eastern Europe into Pannonia and the Balkans must have already occurred during the Iron Age and Roman Period, as we identify a substantial number of individuals within the SP cluster buried in Austria, Hungary, Serbia and Montenegro during the time period from 500 bce to 300 ce, predating the large-scale population movements of the sixth and seventh centuries (Supplementary Figs. 29a, 32a,b and 33).

**Fig. 4:**
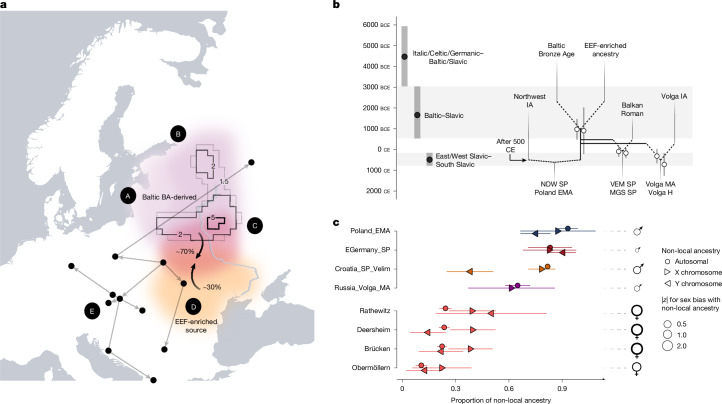
Formation of the SP gene pool. **a**, Contours indicate the averaged MOBEST maximum probability at search time 1,950 years before present for 20 individuals from Niederwünsch (denoting the mean prediction of the geographic regions where the ancestors of these individuals originated). This is supplemented by five lines of evidence: (A) ancient and present-day groups from the Baltics show the highest genetic similarity to SP individuals; (B) Bronze Age and Iron Age individuals from Estonia, Ingria and Karelia are less related to SP individuals than groups from Lithuania and Latvia; (C) populations in Western Russia feature too high proportions of Steppe and/or Siberian ancestry; (D) SP individuals are enriched in EEF and depleted in WHG ancestry compared with Bronze Age and Iron Age populations from the Baltics; (E) Putative migration directions inferred using pairwise mean sIBD sharing values between SP sites^77^ (*n* > 2). Made with Natural Earth. **b**, Comparison of linguistic split times (left) and genetic admixture dates in SP groups (right). Divergence date distributions for the Balto-Slavic and Slavic subgroups were extracted from a sample of 37,004 trees^78^. Genetic admixture dates were obtained using DATES. Error bars indicate 2 × s.d. H, historical. **c**, Sex-biased admixture in four MP and four SP populations. Shown are non-local ancestry proportions on the autosomes, X chromosome and the Y chromosome (Y-chromosome haplogroups R1a, N and I2 for SP populations; E, G, J and T for MP populations). Points denote qpAdm (autosomes and X chromosome) or maximum likelihood (Y chromosome) estimates. Estimates were obtained as described in Supplementary Notes 7.2 using ancient source groups. The corresponding data can be found in Supplementary Table 47. Error bars indicate 2 × s.d. ♂ indicates an excess of non-local males in the admixture process; ♀ indicates a non-local female bias. The size of the symbols denotes the strength of the sex bias (with |*z*| > 2 being considered significant).

Using newly generated early medieval data from the Polish site Gródek, Hrubieszów County, near the Ukrainian border, which represents some of the oldest Slavic inhumation burials from Poland (dating between the seventh and ninth centuries ce), as a proximal source (both in time and space) for the incoming BAL-enriched ancestry, we calculate using qpAdm that approximately 82 ± 1%, 83 ± 6%, 93 ± 3% and 65 ± 4% of the local gene pool in the Northwestern Balkans, Eastern Germany, Poland–Northwestern Ukraine and the Volga-Oka valley, respectively, were replaced during the SP by migrants from Eastern Europe (referred here to as ‘SP ancestry’; Methods) (Fig. 4c and Supplementary Tables 47 and 51). These results contradict a model of substantial population continuation from the Iron Age or MP to the Middle Ages in present-day Western and Central Poland, where previous research claimed an autochthonous origin of the SP gene pool^29,46,47^ (Extended Data Fig. 4). Yet more samples are needed to assess the overall degree of genetic replacement over the larger area. Applying qpAdm to model present-day groups using ancient source populations, we show that Eastern European ancestry is the dominant genetic component in all Slavic-speaking populations today and is also found in neighbouring non-Slavic-speaking groups in Central Europe and regions bordering to the south (Extended Data Fig. 7 and Supplementary Note 5). We measure the highest proportions of Eastern European ancestry in present-day Ukraine, Belarus and Poland, from where it gradually decreases to the east and south (Extended Data Fig. 7 and Supplementary Table 41). Notably, we observe a profound duality to the west, in Eastern Germany, with the present-day German-speaking population from Saxony exhibiting around 40% SP ancestry and the Slavic-speaking Sorbs of Upper Lusatia (Saxony) exhibiting 88% SP ancestry (comparable to modern Poles) (Extended Data Fig. 7). This agrees with previous studies on the genetic isolation of the Sorbs^33,48^ and is consistent with them representing the descendants of these Slavic groups that were minimally (or at least less) integrated into the reproductive networks of the expanding German-speaking settlement east of Elbe and Saale from the twelfth century onwards^49–51^. Conversely, we suggest that the German eastward expansion and earlier Frankish conquest is probably associated with the reduction in SP ancestry observed in the German-speaking population.

## Formation and origin of SP ancestry

**Extended Data Fig. 6:**
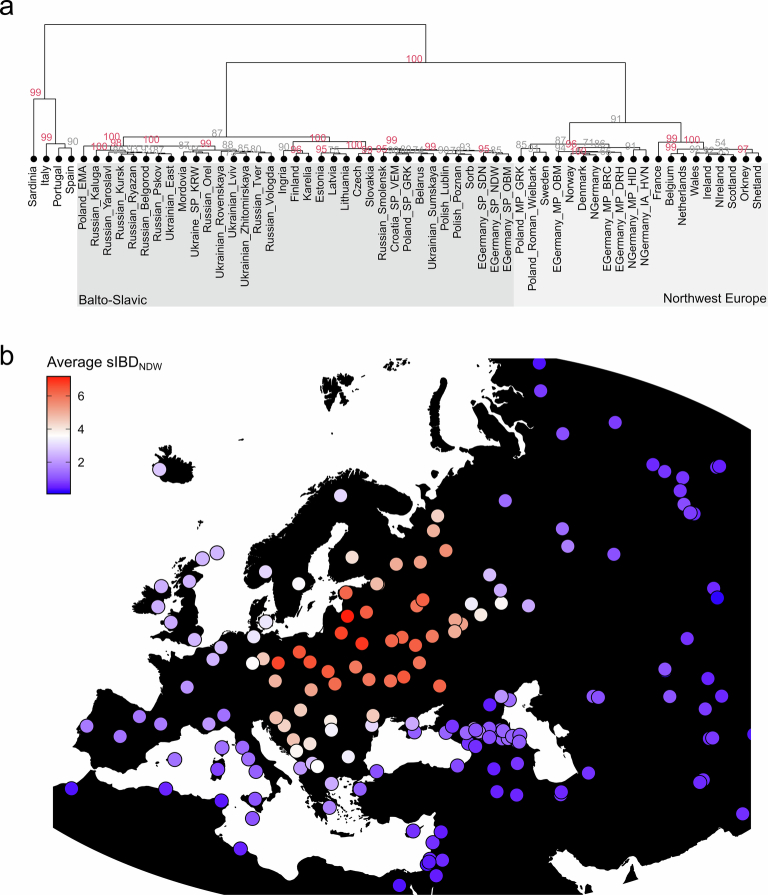
Genetic relatedness between present-day populations and ancient SP groups. **a**, Hierarchical cluster analysis applying Ward’s minimum variance method to the normalized, average IBD sharing (> 1 cM) between relevant ancient (n = 14) and present-day (n = 45) populations from Eastern and Northwestern Europe. The dendrogram and statistical support for the bifurcations from multiscale bootstrap resampling are shown. Made with Natural Earth. **b**, Normalized, average IBD sharing (> 1 cM) between SP individuals (n = 182) from Niederwünsch, Eastern Germany, and present-day European, Asian, and North African populations (n = 215).

**Extended Data Fig. 7:**
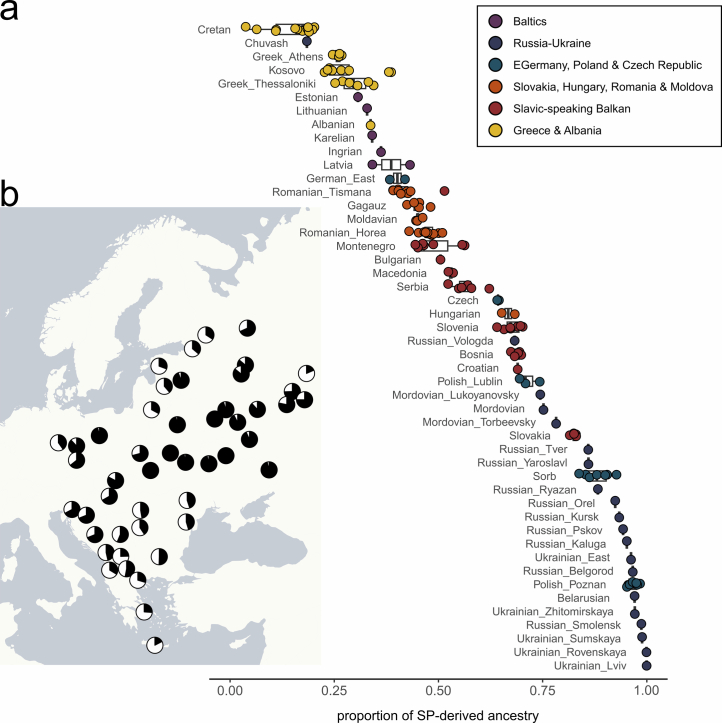
The genetic legacy of the Slavic expansion. **a**, Results from qpAdm analyses. Two- and three-way models of ancient source groups were fitted on relevant present-day groups from Southeast, Central, and East Europe (n = 48). The proportion of GRK-related (SP) ancestry is shown. For groups with multiple significant qpAdm models (p > 0.01), the GRK proportions from all feasible models are shown as box plots. Bounds of the box represent the 25^th^ and 75^th^ percentiles. The centre represents the median. Whiskers represent the smallest value greater than the 25^th^ percentile minus 1.5 times the interquartile range and largest value less than the 75^th^ percentile plus 1.5 times the interquartile range, respectively. Outliers present the minimum and maximum values in the data. **b**, Pie charts depicting the median ancestry proportions from all significant (p > 0.01), feasible qpAdm models for present-day European groups. GRK-related (SP) ancestry is shown in black, summed non-SP ancestry is shown in white. The pie charts are plotted on the sampling locations. Made with Natural Earth.

Both *F*_4_ and *F*_ST_ statistics identify the highest genetic similarity between SP individuals and present-day populations from the Baltics, Poland and Belarus (Supplementary Figs. 17–22). These are also the regions where BAL and SP ancestry (here approximated by medieval samples from Gródek) are maximized today and where the highest proportions of R1a haplotypes (specifically R1a-M458 and R1a-M558) are found among the male population. In patterns of haplotype sharing between the ancient and modern individuals, this similarity was mirrored in a distinctive IBD signal (Extended Data Fig. 6 and Supplementary Figs. 23–25): SP individuals from all three study regions share more and longer IBD fragments with Eastern Europeans than with any other Eurasian group, establishing direct genetic relatedness between present-day Balto-Slavic speakers and SP individuals in Central and Southeastern Europe (Extended Data Fig. 6 and Supplementary Note 4.3.1). This pattern of excess affinity to Northern and Northeastern Europeans is not only evident in the comparison with present-day data but also in the archaeogenetic record: Comparing the SP individuals to other ancient samples, we show that they, independent of their geographic origin, share the highest drift and largest sum of IBD with Bronze and Iron Age groups from Lithuania, Latvia and Estonia^52,53^, and are (as shown by *F*-statistics) more closely related to these individuals than to any other population in post-Neolithic Europe (for IBD see Supplementary Fig. 32c; for *F*_3_ and *F*_4_ see Supplementary Figs. 34–38; Supplementary Note 4.4.1).

Yet, in contrast to the Bronze Age Baltic samples, we note that SP individuals from all study regions exhibit substantially less Western hunter-gatherer (WHG) and more early European farmer (EEF) ancestry (Supplementary Figs. 41 and 42a and Supplementary Note 4.4.2). This suggests that the SP groups in Central Europe were already admixed, most probably between a WHG and Steppe ancestry-enriched Baltic Bronze Age-related source from the sub-Neolithic forest zone and at least one EEF-enriched source from the south. Using qpAdm, we identify various groups in Southeastern and East-Central Europe that constitute working proxies for such an EEF-enriched donor, yet we are not able to precisely identify the most likely representative (Supplementary Fig. 43). Across all fitting two-way models (*P* > 0.01) (and most non-fitting), the admixture proportions are highly similar, with the Eastern German and Polish-Northwestern Ukrainian SP samples receiving around 71% Baltic (95% confidence interval: 66.5%–76%) and around 29% (95% confidence interval: 24%–33.5%) EEF-enriched ancestry (Supplementary Fig. 43b and Supplementary Table 34). However, we highlight that the demographic trajectories that led to the formation of the SP gene pool were potentially more complex than a simple two-way admixture event. Although we calculate similar estimates of Baltic Bronze Age-derived ancestry applying a non-negative least squares approach based on PCA- and ADMIXTURE results^54^, mirroring previous results from genome-wide genealogies^55^, all models profit from the inclusion of an additional Western European source (Supplementary Figs. 40, 45 and 46). Thus, which vector population(s) ultimately transmitted EEF-enriched ancestry to the Northeast cannot be resolved fully for now (Supplementary Note 4.4.2-3).

**Extended Data Fig. 8:**
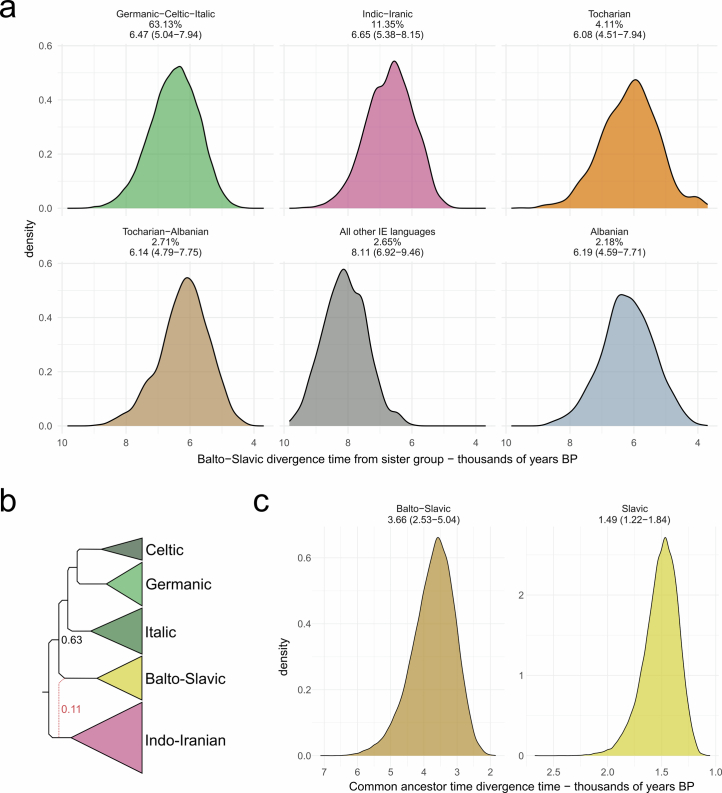
Temporal dynamics of the Slavic languages. **a**, Divergence date distributions for the Balto-Slavic family from other Indo-European languages, extracted from 37,004 trees generated in Heggarty et al.^78^. Posterior probabilities of sister-group relationship, median split times and respective 95% Highest Posterior Distributions (HPD) are indicated above. Divergence dates are given independently for each of six sister group relationships found in the posterior tree sample, in decreasing order of probability. **b**, Schematic of the underlying Maximum Clade Credibility (MCC) tree from all tree distributions. The posterior probabilities of Balto-Slavic forming a clade with Germanic-Italic-Celtic (0.63) and Indo-Iranic (0.11) are indicated. **c**, Divergence date distributions for the Baltic and Slavic languages (left) and the South and East/West Slavic languages (right), extracted from 37,004 trees generated in Heggarty et al.^78^. Median split times and respective 95% HPD are indicated above.

Assuming a two-way admixture process, using DATES (distribution of ancestry tracts of evolutionary signals)^56^ we obtained an average date of approximately 1000 bce for this admixture event that formed the SP gene pool (972 bce ± 250 for Niederwünsch and 906 bce ± 362 for Poland_EMA, respectively) (Fig. 4b and Supplementary Fig. 35). Of note, these DATES estimates overlap with the more recent part of the distribution of divergence estimates between Baltic and Slavic languages (Fig. 4b). Both phylogenetic analysis of cognate-coded basic vocabulary data (Fig. 4b and Extended Data Fig. 8) and most Indo-European linguists date the disintegration of Proto-Balto-Slavic^57–64^ to the second millennium bce^57,64^. However, since the Bayesian linguistic estimates are on average shifted a few centuries older than the admixture estimates, we highlight the possibility that the admixing Baltic-related groups spoke a language that had already begun to diverge from the language or dialect continuum of the populations further north, the former eventually becoming the Slavic languages and the latter the (present-day) Baltic languages. To identify the most plausible geographic location for this initial formation of the SP gene pool, we applied MOBEST to perform spatiotemporal interpolation of the genetic affinities of SP individuals from the study regions to approximately 5,660 previously published ancient samples from Western Eurasia, obtaining similarity probabilities across Europe that can be interpreted as proxies for geographical origin at a specific time. We set the prediction time to 1,950 years before the present, providing us the most likely origin of an individual at this time point (and thus before the demographic transition in Central Europe). Averaging the probability surfaces, we infer a region spanning the south of Belarus and north of Ukraine as the best spatial proxy for the origin of the SP individuals in our three study transects (Fig. 4a and Supplementary Figs. 33 and 34). Such a range would agree well with the area where many linguists propose the earliest development of Slavic languages and archaeologists locate the origin of Slavic-associated material culture^5^ (Supplementary Fig. 44); however, more ancient DNA (aDNA) data are needed to conclusively assess the genetic landscape of this region.

From there, Northeastern European ancestry is likely to have spread east, west and south, admixing with or even replacing the local gene pools (Fig. 4a). Although we cannot precisely measure the onset of this expansion or its duration, we highlight that DATES estimates for admixture between local and immigrant ancestries in SP individuals are generally recent and similar across the study transects, consistent with admixture processes starting in the sixth and early seventh century and agreeing with historically recorded arrival dates of Slavic groups in these regions (Extended Data Fig. 1). The detection of substantial genetic introgression from the northeast into regions in which Slavic came to be spoken^26,27,44,65,66^ indicates that the diffusion of Slavic language and Eastern European-derived ancestry were related, although the degree of their overlap cannot be ascertained. This provides a plausible explanation for the high genetic relatedness across present-day Slavic-speaking groups^26,44^, which was previously linked to the spread of the Slavic languages^44,65^. However, we highlight that such a simplified model does not capture the more complex regional dynamics that emerge from historical and archaeological evidence, and are still evident in language boundaries that do not correspond to genetic differences across the Balkans and Central Europe^66^. To investigate possible sex biases in these expansion and admixture processes, we compared estimates of SP-related ancestry on the X chromosomes and the autosomes to identify proportion differences indicative of male-biased admixture (Fig. 4c). Notably, we find no evidence for sex bias in any of the SP populations in Germany, Croatia, Poland or Russia (|*z* | <2; Fig. 4c and Supplementary Table 47). However, we observe that the previously undetected gene flow of Southern European-related ancestry into the MP population of Eastern Germany was significantly female-biased in most studied sites (Fig. 4c and Supplementary Fig. 59d).

## Social changes in Eastern Germany

**Extended Data Fig. 9:**
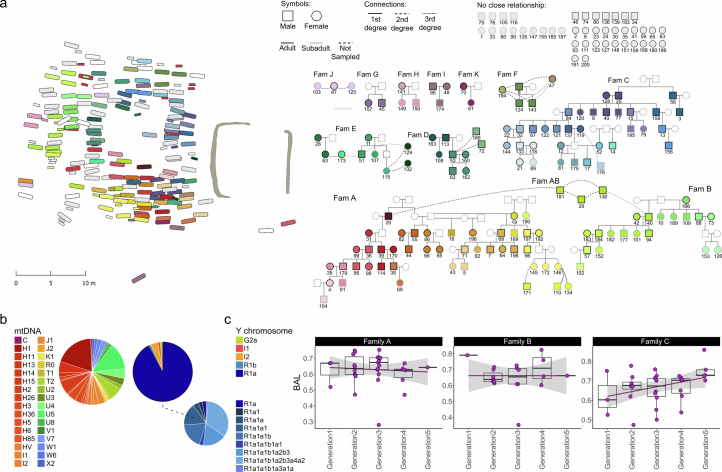
Cemetery and family structure in Eastern Germany during the Slavic Period. **a**, Reconstructed pedigrees of Niederwünsch, Eastern Germany, coloured according to family lineages. The site layout is shown on the left, representing the spatial distribution of family lineages coloured as in the pedigrees. Numbers correspond to the Genetic IDs listed in Supplementary Table 1. **b**, Distribution of mtDNA (n = 146) and Y chromosome haplogroups (n = 75). **c**, Trajectories of BAL ancestry over 5 generations across three major pedigrees of families A (n = 41), B (n = 19) and C (n = 33).

**Extended Data Fig. 10:**
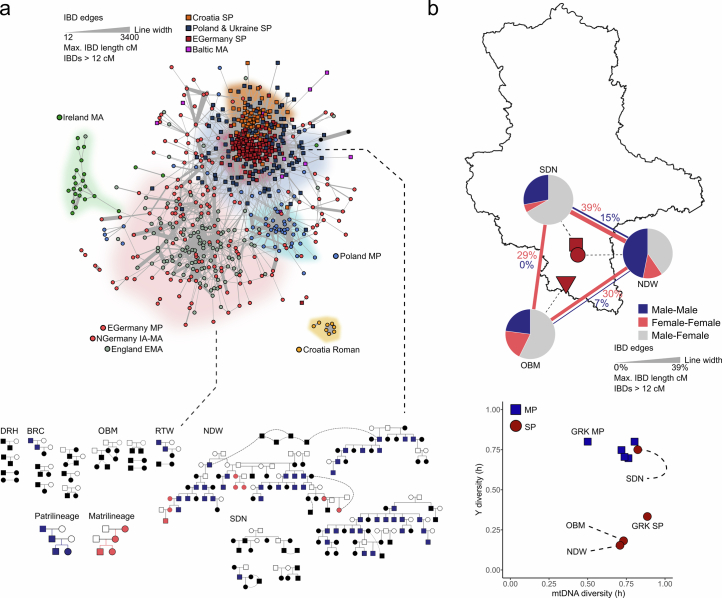
Changes in genetic relatedness between and within populations. **a**, The network on the left shows all pairs of ancient individuals (n = 674) sharing IBD segments longer than 12 cM. We note that early medieval individuals from England, Northern and Eastern Germany form a loose cluster, while Slavic Period individuals from Croatia, Eastern Germany, and Poland-Northwestern Ukraine plot tightly together. The pedigrees on the bottom were constructed from the ancient genomic data and demonstrate the shift from small to large, patrilineally-organized pedigrees in Eastern Germany following the arrival of Slavic groups. Males are shown as squares, females as circles. Empty symbols denote reconstructed individuals that are not present in the genetic data. Patrilineal sequences are highlighted in blue, matrilineal in red. **b**, The pie charts show the proportion of the total intra-site IBD shared between pairs of males and females for the SP sites Niederwünsch (n = 182), Steuden (n = 36), and Obermöllern (n = 22) in Eastern Germany. The lines connecting the pie charts depict the proportion of the total inter-site IBD shared between pairs of males and pairs of females between the three different sites. Line thickness is proportional to the fraction of shared IBD. Shown below are trends in mtDNA and Y chromosome haplotype diversity (h) calculated for seven MP & SP sites in Eastern Germany and MP & SP Gródek in Eastern Poland. Made with Natural Earth.

The Slavic groups that we studied also showed fundamentally different social organization compared with the preceding MP population (Supplementary Note 8). Most notably, we highlight more intense inter-site and intra-site genetic relatedness in the Elbe-Saale region (Extended Data Fig. 2c), reflected by patrilineally organized pedigrees that comprise large numbers of individuals (Extended Data Figs. 9 and 10a,b). The cemeteries of the preceding MP in Eastern Germany were characterized by small units of biological relatedness, mostly consisting of fewer than four first- and second-degree relatives. At the site level, we identified for each individual an average of 1.16 ± 0.18 close relatives (here defined as all relationships up to third degree) (in Brücken specifically: 0.64 ± 0.14; Supplementary Fig. 62). This pattern is also mirrored in IBD sharing within sites (which also captures distant genetic relatedness greater than third degree), with the proportion of pairs of individuals that share any IBD larger than 12 cM ranging between 1 ± 0.4%, 6.8 ± 1.8% and 5.2 ± 1.8% in Brücken, Deersheim and Obermöllern, respectively.

By contrast, during the SP, we show that the number of close relatives at the sites increased nearly sixfold to 6.41 ± 0.4%. We even observe one case of seven offspring from the same couple (Extended Data Figs. 9 and 10b). Notably, four of the seven siblings had reached reproductive age, with three of them having offspring. As most of them were male, we can assume that several grown-up daughters might had gone elsewhere to marry. Moreover, the majority of offspring being male points towards additional unsampled female siblings (to statistically account for an equivalent number of females born). Notably, for all unions (in which at least one parent was identified on site), we find 52 sons (62% of the offspring; 95% confidence interval: 51–72%) but only 32 daughters (38% of the offspring; 95% confidence interval: 28–49%) (exact binomial test; *P* = 0.03753).

More distant genetic relationships also increased. For Niederwünsch and Steuden (approximately 8 km apart), 18.3 ± 0.6% and 15.3 ± 2.1% of the pairs of individuals share IBD fragments indicative of recent common descent (more than 12 cM apart). Although occupation periods in MP sites were shorter than in Niederwünsch (preventing the emergence of large-pedigree structures), the rather short-lived site of Steuden demonstrates that the differences in intra-community relatedness between MP and SP cemeteries were not caused exclusively by differences in the duration of site occupation.

Although these extensive kinship networks evidenced a high degree of relatedness among all individuals within sites, we do not find a single case of close consanguinity (defined here as offspring of first cousin unions or closer) (Supplementary Fig. 54d). This shows profound knowledge of the lineages and deliberate avoidance of consanguinity. We also identify at least 11 cases of individuals reproducing with multiple partners, pointing to polygamy or serial monogamy. Despite a 2.7:1 ratio of half-siblings sharing the same father (95% confidence interval: 0.43–0.91) versus those sharing the same mother (95% confidence interval: 0.09–0.57), we do not find a single instance of levirate marriages as practiced in late Avar-period communities in the Carpathian Basin^67^.

In parallel with the increase of genetic interconnectedness within the sites, we also observe that the organization of the cemeteries changed, reflecting in the spatial layout the extended pedigrees. Although close relatives were buried significantly closer together than non-related individuals in both the MP and the SP, only during the SP did cemeteries feature a significant correlation between genetic and spatial distances, suggesting that the cemeteries were planned and structured around these larger kin groups (Mantel statistic based on Spearman’s rank correlation; *P* = 0.0001 for both Niederwünsch and Steuden) (Extended Data Fig. 9 and Supplementary Figs. 63 and 65). This signal is most prominent in the site of Steuden, where at least 27% of all variance in spatial distances between graves is explained by genetic relatedness.

Although the sex ratio of adults across the SP sites is balanced (Exact binomial test; *P* = 1 for Steuden, *P* = 0.08794 for Niederwünsch), females have on average significantly fewer close relatives than males (Fisher’s exact test; *P* = 0.008) and feature overall an increased pairwise mismatch rate compared with males (Wilcoxon rank sum test; test statistic (*W*) = 13,976,553, *P* = 0.04218; Supplementary Fig. 67) and a lower sum of IBD segments shared within sites (Welch two-sample *t*-test; *t* = −3.707, d.f. = 355.82, *P* = 0.0002431) (Extended Data Fig. 10c). However, females share more IBD segments between different sites than males, thereby demonstrating higher inter-site relatedness among females in contrast to higher intra-site relatedness among males (Welch two-sample *t*-test; *t* = 2.9513, d.f. = 64.952, *P* = 0.004398) (Extended Data Fig. 10c).

This demonstrates that exogenous origin was more common for females than for males, suggesting a patrilocal inheritance organization, and agrees well with the nearly exclusively patrilineally organized pedigrees (for example, 88% patrilineal lineages (95% confidence interval: 68–97%) versus 12% matrilineal lineages (95% confidence interval: 4–32%)) and the significant underrepresentation of female offspring at the sites (Extended Data Figs. 9 and 10b and Aupplementary Fig. 54). Across all SP sites in Eastern Germany, we find only one instance of mitochondrial haplogroups being transmitted further than one daughter generation. This contrasts with the preceding MP population, for which we detect no difference in the number of close relatives between males and females (Fisher’s exact test; *P* = 0.74) and no difference in pairwise mismatch rate in general (Wilcoxon rank sum test; *W* = 3,215,752*, P* = 0.06), potentially suggesting a less strictly patrilineal social system before the arrival of Slavic groups (Supplementary Fig. 67). However, owing to the overall smaller number of identified relatives, these tests might be less statistically conclusive and underestimate signals of MP female exogamy and patrilineal practices.

Patterns of patrilocal organization into kin groups are broadly similar across regions and might have contributed to the previously described turnover and homogenization of the paternal gene pool (Extended Data Fig. 10b). In particular, we find an identical pattern of correlation of spatial and genetic distances in SP Velim, Croatia (Mantel statistic based on Spearman’s rank correlation; *P* = 0.0001; Supplementary Figs. 64 and 65d) as well as evidence for patrilocality and female exogamy (for example, more close biological relatives among males than among females (Fisher’s exact test; *P* = 0.027) and higher pairwise mismatch rates among females compared to males (Wilcoxon rank sum; *W* = 156,550, *P* = 0.02377; Supplementary Fig. 67g–i)), mirroring the social stratification observed in the Elbe-Saale region. By contrast, at Velim the mean number of close relatives in the site is significantly lower than in Niederwünsch or Steuden (1.01 ± 0.17). Within a shared pattern of patrilocality, SP fine-scale organization differed substantially across Central Europe owing to the complex, regionally contingent nature of this expansion. Rather than simple replacement, partial integration of the local population was probably dependent on the fortunes of specific groups or families. However, the substantial number of individuals from sites across Eastern Germany, Croatia and Poland–Northwestern Ukraine who share comparably large amounts of IBD with each other confirms that these Slavic-associated groups were closely linked as the result of a shared biological origin and recent geographical expansion.

## Discussion

Here we present several results in a wide chronological and topographical range, based on fine-grained genetic transect studies of a number of complete cemeteries. Across Central Europe, we show that during the Roman Iron Age and subsequent MP genetic diversity increased not only within the empire, but also outside it. Southeastern Europe experienced an influx from the Eastern Mediterranean, whereas large parts of East-Central Europe were inhabited by people mainly related to modern inhabitants of Northern and Northwestern Europe. We also show a fifth and sixth century admixture of Mediterranean ancestry in Eastern Germany, prevalently but not exclusively through females. They and their offspring became well-integrated in the regional societies, and their burials show no signs of inferior status or cultural differences, a finding very different from those at early medieval cemeteries in Hungary, Italy^39^ and England^68^ with respect to incoming Northern versus local, Southern and Western European ancestry. Yet, after the fall of the Thuringian kingdom this diverse population gradually disappeared, and the SP began.

Most importantly, our results are relevant for the debate on whether the spread of Slavs was due to large migratory movements and the expansion of a homogeneous population, or to the gradual acculturation of regional populations. We were able to connect the fundamental transformations of culture in large parts of Eastern Europe to a substantial movement of people most plausibly from regions between the Eastern Baltics and the Northwestern Pontic region. This genetically inferred area agrees well with archaeological hypotheses for the origins of Slavic material culture east of the Vistula River, for instance, in the Kyivan culture (second or third to fifth centuries ce)^4,5,20,21^. However, without aDNA from this region and period, more precision remains impossible.

SP ancestry spread to many directions from the sixth century onwards. Yet we can only detect it when the immigrants stopped cremating their dead at different stages between the eighth and tenth centuries. Only few early inhumation burials (for example, Velim and Gródek) provide insights in line with more recent sites. Consequently, it remains unclear whether most of the SP ancestry arrived in a single pulse or over a prolonged time span. As described in previous publications^27^, our results indicate that sporadic individual percolation from Eastern Europe into the Balkans preceded (and potentially succeeded) any large-scale movements, pointing to long-term mobility accompanying the sixth and seventh century demographic transformation. To what extent the previous population disappeared, continued to live alongside the newcomers or mixed with them depended on the region^69–71^. Among our study regions, the shift was most marked close to the areas of origin, as in Poland and Eastern Germany. By contrast, we found more admixture with local populations in the Northwestern Balkans, the Carpathian Basin and the Volga-Oka area. Remarkably, the Slavic population buried in Eastern Germany in the late tenth to twelfth century, nearly four centuries after the first Slavic groups settled in the wider region, had little genetic exchange with the neighbouring Thuringian population, although archaeological finds demonstrate regular interactions between these groups. To some extent, this discrepancy might be caused by the selection of studied sites within a more heterogeneous genetic landscape. However, our results agree with the drastic break between the preceding Germanic material culture and that of the Early SP, probably after an intermediate phase of scarce settlement in most of the Northwestern Slavic area after the mid-sixth century^69–75^, and disagree with the model of a Slavicisation of the existing population.

This change was driven by substantial movements of people and can be linked with the dispersion of Slavic languages and the spread of a new material culture. However, our data do not support total population replacement across Eastern Europe, but point to a complex, regionally contingent demic diffusion with partial integration. SP ancestry in the Balkan Peninsula and Western Russia did not form the majority in all areas. Consequently, besides large-scale movements, several other mechanisms, including genetic and cultural assimilation of the autochthonous populations by the expanding SP gene pool, are needed to explain the ancient and present-day genetic patterns observed on the margins of the diffusion area. Where the newcomers mixed with local populations, they did so without significant gender bias.

Unlike in the MP, the SP communities in the Elbe-Saale region relied more on biological relatedness in their organization, and their large pedigrees provide valuable information about their reproductive practices and social structure. Similar to earlier Avar communities in Pannonia^67,76^, the multi-generational SP communities we studied in Eastern Germany practiced a consistent reproductive strategy based on patrilineal and local descent, female exogamy, strict avoidance of consanguinity, and—in several cases—multiple reproductive partners. Not all of these patterns observed in Eastern Germany were shared with the SP societies in the Northern Balkans, which, in many aspects of their organization, appear more similar to preceding MP groups in Croatia, Eastern Germany, Hungary or Italy^39,40^. In some of these cases, previously established social practices may have survived the substantial demographic and linguistic changes, adding another level of genetic complexity to the new set of questions concerning social organization and interaction within and between Slavic-related societies across Europe.

Box 1 What does ‘Slavs’ mean? In modern ethnic and national terminology, ‘Slavs’ denotes all speakers of Slavic languages and/or citizens of the Slavic nation states. This concept of an ethnic collective spanning several nations is much more marked than among the Germanic or Romance speakers in the rest of Europe (Supplementary Note 1). The extent to which Slavic identity mattered diverged; it was important for nineteenth and twentieth century Slavic nationalisms, Pan-Slavism and Russian imperialism, but regional or national allegiances often carried more weight. The prejudices of their western neighbours who tended to regard Slavs as culturally inferior reinforced sentiments of Slavic commonality. The question of Slavic origins, addressed in this Article, had a crucial role in ideological debates about the unity and the significance of the Slavs. It is therefore important to be precise in the scholarly use of the term. In research about the early Slavs, the meanings of the term diverge. In written sources since the sixth and seventh century in Byzantium and the west, groups of Slavs or Wends increasingly appear in a wide range of lands beyond and along the Danube and the Elbe rivers. We can make use of different sources to understand how large parts of Europe became Slavic: outside perceptions about Slavs in texts; archaeological traces of shared cultural practices among early Slavs (particularly the Prague-Korchak culture); linguistic reconstructions of a common Slavic language prior to the particular Slavic idioms; and shifts in ancestry of the medieval gene pool, which point to migrations. We should not take these disciplinary results as proxies for each other as attributes of a coherent people called Slavs; yet, they provide different perspectives on the Slavicisation of Europe during the Early Middle Ages. Combining them allows us to overcome simplistic theories of an expansion of the Slavs and instead understand the common dynamic and the different ways in which Slavic peoples began to form in many parts of Europe. We therefore use Slavs for populations named in this way in contemporary texts, without implying that they self-identified as such. These Slavic groups can be localized, but hardly circumscribed. We do not use genetic or archaeological features in regions where Slavs spread to distinguish between Slavs and non-Slavs, or between speakers and non-speakers of Slavic languages, although we assume that these phenomena overlapped to a considerable degree.

## Methods

### Naming

For better comparison of genetic differences between groups pre- and post-dating the proposed expansion of groups associated with Slavic material culture, we refer to samples dating between 300 and 600 ce as MP individuals or groups, and samples dating between 600 and 1200 ce as SP individuals or groups. Although this naming scheme is simplified compared to the well-established historical and archaeological chronologies of our three study transects, it facilitates the quantification of the genetic turnover between groups pre- and post-dating 600 ce across Central Europe. We highlight that our SP samples were selected to represent sites that are considered Slavic in an archaeological sense. Besides this cultural attribution, sites were selected for their completeness and state of archaeological description or publication. However, we do not know if the buried individuals considered themselves as belonging to a Slavic ethnic or cultural community and whether or how they understood their common descent from groups of earlier periods in Eastern Europe. Similarly, whether they spoke a Slavic language can only be hypothetically assumed. We thus prefer a temporal nomenclature that is agnostic as to genetic, cultural or linguistic designations.

When necessary, we refer collectively to SP samples with Eastern Europe-specific ancestry as having SP ancestry. This term describes an Eastern European-derived ancestry component due to a recent common origin shared among most SP individuals in all three study transects. In our analyses this ancestry is approximated using data (*n* = 8) from the Polish site Gródek, Hrubieszów County, dating between 600 and 900 ce and representing the spatially and temporally closest surrogate in our dataset to the (so far) unsampled source population in Eastern Europe. This naming system is intended to differentiate medieval SP ancestry from the present-day genetic landscape of Eastern Europe, which is (at least partially) derived from this ancient source.

In the text, we label large-scale movements of people during the Early Middle Ages as migration, adhering to the traditional use of the term in population genetics^79^. We use the term with intention since the observed movements caused significant and lasting demographic change in the genetic landscape of Europe due to the large-scale, permanent translocation of people from one region to another.

Within tables and figures, we refer to groups of published individuals by the names given in the Allen Ancient DNA resource v.54.1^80^. Sample sizes, context information and publication names can be found in Supplementary Tables 7–11. In the main text and Supplementary Notes, we used the following abbreviations for archaeological time periods: N, Neolithic; C, Chalcolithic; EBA, Early Bronze Age; MBA, Middle Bronze Age; LBA, Late Bronze Age; IA, Iron Age; RA, Roman Age; EMA, Early Middle Ages; MA, Middle Ages; H, historical.

### Permissions for archaeological research

Provenance information for samples from all archaeological sites is given in Supplementary Information Note 2, together with short descriptions of each site, the institution owning the samples (or custodians of the samples), the responsible co-author who obtained permission to analyse and the year of the permission granted.

### aDNA data generation

#### Collection of bone powder

We obtained all permissions for the work with archaeological and anthropological material in this study. Sampling of bone and teeth samples took place in the clean room facilities of the ArcheoGen laboratory at the Department of Archeology and Museology, Masaryk University in Brno, Czech Republic as well as in the clean room facilities of the Max Planck Institute for Evolutionary Anthropology. The sampling workflow included documenting and photographing the provided samples. For teeth, we either cut along the cementum–enamel junction and collected powder by drilling into the pulp chamber or accessed the pulp chamber by drilling the tooth transversally. For the petrous bones, we drilled from the outside of the bone towards the cochlear region in parallel to the auditory canal^81^. Other skeletal material such as long bones were used when no teeth or petrous bones were available. In these cases, areas with best preserved compact bone tissue were drilled. We collected between 25 and 50 mg of bone or tooth powder per sample for DNA extractions. The pulverized bone samples were sent to clean room facilities dedicated to ancient DNA work at the Max Planck Institute for Evolutionary Anthropology for further processing and aDNA acquisition.

#### ^14^C dating

New radiocarbon dates for this study were measured on the bone and tooth fragments sampled for DNA. These dates were obtained at the Curt-Engelhorn-Center Archaeometry gGmbH, Mannheim, using MICADAS-AMS. Collagen was extracted from the previously sampled bones, purified by ultrafiltration and freeze-dried. ^14^C ages were normalized to δ^13^C = −25‰. The calibration was done using the IntCal20 calibration curve^82^ and the Oxcal program^83^.

#### DNA extraction

Ancient DNA was extracted following a modified protocol of Dabney et al.^84^, as described at https://www.protocols.io/view/ancient-dna-extraction-from-skeletal-material-baksicwe, where we replaced the extended-MinElute-column assembly for manual extractions with columns from the Roche High Pure Viral Nucleic Acid Large Volume Kit^85^, and for automated extraction with a protocol that replaced spin columns with silica beads in the purification step^86^.

#### Library construction

We generated double-indexed^87^ single-stranded^88^ libraries using 25 µl of DNA extract and following established protocols^89^. We applied the partial UDG (UDG half)^90^ protocol to remove most of the aDNA damage while preserving the characteristic damage pattern in the terminal nucleotides.

#### Capture and sequencing

We enriched libraries using in-solution capture probes synthesized by Agilent Technologies for 1,237,207 SNPs along the nuclear genome^91^. Libraries were sequenced in-house on an Illumina HiSeq 4000 platform to an average depth of 20 million reads and after demultiplexing processed through EAGER^92^.

### aDNA data processing

#### Read processing and aDNA damage

After demultiplexing based on a unique pair of indexes, raw sequence data were processed using EAGER^92^. This included clipping sequencing adaptors from reads with AdapterRemoval (v.2.3.1)^93^ and mapping of reads with BWA (Burrows–Wheeler aligner) v.0.7.12^94^ against the Human Reference Genome Hs37d5, with seed length (-l) disabled, maximum number of differences (-n) of 0.01 and a quality filter (-q) of 30. We removed duplicate reads with the same orientation and start and end positions using DeDup v.0.12.2^92^. Terminal base deamination damage calculation was done using mapDamage v.2.0.6^95^, specifying a length (-l) of 100 bp. For the ten libraries that underwent UDG half treatment, we used BamUtil v.1.0.14 (https://genome.sph.umich.edu/wiki/BamUtil:_trimBam) to clip two bases at the start and end of all reads for each sample to remove residual deaminations, thus removing genotyping errors that could arise due to ancient DNA damage.

#### Sex determination

To determine the genetic sex of the ancient individuals, we calculated the coverage on the autosomes as well as on each sex chromosome and subsequently normalized the X- and Y-reads by the autosomal coverage^96^. For that, we used a custom script (https://github.com/TCLamnidis/Sex.DetERRmine) for the calculation of each relative coverage as well as their associated error bars^97^. Females are expected to have an X rate of 1 and a Y rate of 0, while males are expected to have a rate of 0.5 for both X and Y chromosomes.

#### Contamination estimation

We used the ANGSD (Analysis of Next Generation Sequencing Data) package^98^ (v.0.923) to test for heterozygosity of polymorphic sites on the X chromosome in male individuals, applying a contamination threshold of 5% at the results of method 1. For male and female samples, we estimated contamination levels on the mtDNA using Schmutzi^99^ (v.1.5.4) by comparing the consensus mitogenome of the ancient sample to a panel of 197 worldwide mitogenomes as a potential contamination source, applying a contamination threshold of 5%.

#### Genotyping

We used the program pileupCaller (v.1.4.0.2) (https://github.com/stschiff/sequenceTools.git) to genotype the trimmed BAM files of 10 UDG half libraries. A pileup file was generated using samtools mpileup with parameters -q 30 -Q 30 -B containing only sites overlapping with our capture panel. From this file, for each individual and each SNP on the 1240k panel^36,37,100^, one read covering the SNP was drawn at random, and a pseudo-haploid call was made—that is, the ancient individual was assumed homozygous for the allele on the randomly drawn read for the SNP in question. We used the parameter -SingleStrandMode, which causes pileupCaller to ignore reads aligning to the forward strand at C/T polymorphisms and at G/A polymorphisms to ignore reads aligning to the reverse strand, which should remove post-mortem damage in ancient DNA libraries prepared with the non-UDG single-stranded protocol. To maximize our resolution, we filled missing data in the single-stranded libraries with additional genotypes present in the trimmed, double-stranded but not in the single-stranded libraries.

#### Mitochondrial and Y-chromosome haplogroup assignment

To process the mitochondrial DNA data, we extracted reads from 1240k data using samtools (v.1.3.1)^101^ and mapped these to the revised Cambridge reference sequence (rCRS). We subsequently called consensus sequences using Geneious R9.8.1^102^ and used HaploGrep 2 (v.2.4.0)^103^ (https://haplogrep.uibk.ac.at/; with PhyloTree version 17-FU1) to determine mitochondrial haplotypes. For the male individuals, we used pileup from the Rsamtools package to call the Y-chromosome SNPs of the 1240k SNP panel (mapping quality ≥30 and base quality ≥30). We then manually assigned Y-chromosome haplogroups using pileups of Y-SNPs included in the 1240k panel that overlap with SNPs included on the ISOGG SNP index v.15.73 (Y-DNA Haplogroup Tree 2019-2020; 2020.07.11).

#### Identity-by-descent

We used the ancient-DNA-specific genotype caller MLE function of ATLAS^104^ (https://bitbucket. org/wegmannlab/atlas/) to call genotype likelihoods. ATLAS can also calculate the base-quality recalibration (the recal function) that we performed in batches among libraries sequenced in the same sequencing run, accounting for specific sequencing errors. ATLAS recalibration also corrects the base qualities accounting for the empirical ancient DNA damage pattern observed from the data and reduces the effect of reference bias introduced by genome mapping by relying on a list of 10 million highly conserved genomic positions across 88 mammal species downloaded from ensembl (https://grch37.ensembl.org/). We called genotype likelihoods on the whole 1000 Genomes SNPs panel of around 20 million SNPs and used these calls as input data for GLIMPSE^105^ (v.2.0.0) (https://github.com/odelaneau/GLIMPSE), applying the default parameters and using the 1000 Genomes reference panel. The function GLIMPSE_phase was used to perform simultaneous imputation and phasing on genomic chunks of 2,000,000 base pairs with a buffer of 200,000 base pairs. Samples with more than 600k SNPs exhibiting a genotype posterior of ≥0.99 after imputation were included in downstream IBD analysis. We then estimated segments that were IBD either in the context of: (1) other ancient individuals; or (2) present-day populations.

1. To investigate IBD sharing between pairs of ancient individuals we used the program ancIBD^43^. We applied the HapBLOCK function of ancIBD to perform the pairwise estimation with default parameters and only shared blocks of more than 8 cM containing more than 220 SNPs per centimorgan were considered. To further filter for possible false-positive hits in our IBD network analysis, we considered only shared IBD segments longer than 12 cM, and if a pair of individuals had segments of less than 16 cM, we included them only if they had more than one such segment.
2. To compare IBD segments shared with present-day individuals as well as estimates of effective population size, we used BEAGLE^106,107^ (v.5.2) to phase the newly imputed genotypes. Following Morez et al.^108^, the window and overlap lengths were set as wider than any chromosome (window length 380 cM and overlap length 190 cM) to maximize the information used for phasing the genomes. The 1000 Genomes phase 3 dataset (http://bochet.gcc.biostat.washington.edu/beagle/1000_Genomes_phase3_v5a) and GRCh37 genomic maps (http://bochet.gcc.biostat.washington.edu/beagle/genetic_maps/) provided by BEAGLE were used for phasing. We phased ancient and present-day data together since the BEAGLE phasing algorithm (hidden Markov model-based haplotype clustering) improves widely as the sample size increases. The identification of IBD segments was done using RefinedIBD^109^, which can also detect IBD fragments shorter than 8 cM. The window size was set to 3 cM. The minimal size for a segment to be considered shared by IBD is 1 cM, the same threshold used in Margaryan et al.^110^ and Morez et al.^108^. Finally, we removed gaps between IBD segments that have at most one discordant homozygote and that are less than 0.6 cM in length and aggregated the sum and number of IBD segments between each pair of ancient and present-day individuals.

#### Kinship estimation

To infer biological relatedness between individuals, we applied two independent approaches:

1. We calculated the pairwise mismatch rate^111^ in all pairs of individuals from our pseudo-haploid dataset to double-check for potential duplicate individuals and to determine first-, second- and third-degree relatives. For this purpose, we also used BREADR^112^ which utilizes Bayesian posterior probabilities for the classification of the genetic relationships.
2. We estimated the parts of the genome that are IBD in all pairs of individuals. We used KIN^113^ as the primary IBD-based method, although we validated the relatedness estimates with the method LcMLkin^114^. Both methods use genotype likelihoods to estimate the three *k* coefficients (*k*_0_, *k*_1_ and *k*_2_), which define the probability that two individuals have zero, one or two alleles that are IBD at a random site in the genome.

#### Diversity and population size

We calculated estimates of genetic diversity and effective population size using three different approaches.

1. *Inbreeding estimation.* We calculated the length of runs of homozygosity (RoH) using the software HapROH^115^ (v.0.6) in our pseudo-haploid data. A SNP cutoff of 300k SNPs was used, as well as the default 1000 Genomes reference panel. The number of RoH of size 4–8 cM per individual reflects the rate at which distant relatives have children, providing information about the sizes of mate pools (*N*_e_) averaged over the hundreds of years prior to when individuals lived; offspring of close kin unions are reflected by sums of RoH >50 cM across the genome in runs of homozygosity of >12 cM.
2. *Variability in population structure*. We used the FSTruct^116^ package to quantify the variability of the *Q* matrix (which contains the row vectors of ancestry coefficients for each individual) outputted by supervised ADMIXTURE. The ancestry variability is measured in the ratio ${F}_{{\rm{ST}}}/{F}_{{\rm{ST}}}^{\text{max}}$ (where ${F}_{{\rm{ST}}}^{\text{max}}$ is the maximal value of the fixation index *F*_ST_). We generated 1,000 bootstrap replicate matrices for computing ${F}_{{\rm{ST}}}/{F}_{{\rm{ST}}}^{\text{max}}$ to compare bootstrap means and identify significantly different variabilities.
3. *Effective population size*. We used the method IBDNe^117^ to estimate ancestry-specific historical effective population size from around 4 generations to around 50 generations ago using identity-by-descent segments inferred from our imputed diploid data. We removed IBD segments shorter than 2 cM as well as IBD segments from avuncular and closer relationships using the parameter filtersamples: true. Confidence intervals for the estimated effective population size were calculated using 500 bootstrap replicates.

### Population genetic analysis

#### Dataset

We merged our ancient DNA data with previously published datasets of ancient individuals reported by the Reich Lab in the Allen Ancient DNA Resource v.54.1 (https://reich.hms.harvard.edu/allen-ancient-dna-resource-aadr-downloadable-genotypes-present-day-and-ancient-dna-data) (1240k SNP panel)^80^. For comparisons with present-day groups, we compiled and curated a high-resolution, quality-filtered reference dataset containing genotypes for 426,135 SNPs (the intersection of several different Affymetrix and Illumina chip types) from 12,176 contemporary individuals sampled from 49 (mostly European and West Asian) populations from previously published datasets as described in Gretzinger et al.^68^. Sample sizes are given in Supplementary Table 3. We produced four different datasets:

1. The whole 1240k panel (1240 K; 1.2 M SNPs). Used exclusively for comparison between ancient individuals as well as ancIBD and qpAdm analysis.
2. The overlap between the 1240k and HO panel (1240KHO; 0.6 M SNPs). Used for HO West Eurasian PCA.
3. The overlap between the 1240k and high-resolution panel (1240KEU; 0.4 M SNPs). Used for most analyses including ancient and present-day populations, e.g. PCA, *F*_4_ statistics and ADMIXTURE.
4. The overlap between the 1240KHO and the high-resolution panel (1240KHOEU; 0.3 M SNPs). Used for the calculation of SP ancestry proportions in present-day populations as well as *F*_ST_ and RefinedIBD analysis.

#### Principal components analysis

We carried out PCA using the smartpca software v.16000 from the EIGENSOFT package (v.6.0.1)^118^. We computed principal components on three different sets of European and West Asian populations and projected ancient individuals using lsqproject: YES and shrinkmode: YES.

1. West Eurasian PCA^100^. Poplist: Abkhasian, Adygei, Albanian, Armenian, Balkar, Basque, BedouinA, BedouinB, Belarusian, Bulgarian, Canary_Islander, Chechen, Chuvash, Croatian, Cypriot, Czech, Druze, English, Estonian, Finnish, French, Georgian, Greek, Hungarian, Icelandic, Iranian, Italian_North, Italian_South, Jew_Ashkenazi, Jew_Georgian, Jew_Iranian, Jew_Iraqi, Jew_Libyan, Jew_Moroccan, Jew_Tunisian, Jew_Turkish, Jew_Yemenite, Jordanian, Kumyk, Lebanese, Lezgin, Lithuanian, Maltese, Mordovian, North_Ossetian, Norwegian, Orcadian, Palestinian, Polish, Russian, Sardinian, Saudi, Scottish, Sicilian, Spanish, Spanish_North, Syrian, Turkish, Ukrainian.
2. European PCA^68^. Poplist: Norway, Sweden, Denmark, Netherlands, Belgium, Germany, England, Wales, Scotland, NIreland, Ireland, Orkney, France, Spain, Portugal, Finland, Lithuania, Latvia, Estonia, Mordovia, Russia, Belarus, Poland, Ukraine, Slovakia, Hungary, Italy, Slovenia, Croatia, Bosnia, Serbia, Romania, Bulgaria, Montenegro, North Macedonia, Albania, Greece.
3. Northern European PCA^68^. Poplist: Norway, Sweden, Denmark, Netherlands, Belgium, NGermany, England, Wales, Scotland, NIreland, Ireland, Orkney, France, Finland, Lithuania, Latvia, Estonia, Mordovia, Russia, Belarus, Poland.

#### *F*-statistics

*F*_3_ and *F*_4_ statistics were computed with ADMIXTOOLS v.3.0^119^ (https://github.com/DReichLab). *F*_3_ statistics were calculated using qp3Pop (v.435). For *F*_4_ statistics, we used the qpDstat (v.755) and with the activated *F*_4_ mode. Significant deviation from zero can be interpreted as rejection of the tree population typology ((Outgroup, X);(Pop1, Pop2)). Under the assumption that no gene flow occurred between Pop1 and Pop2 and the Outgroup, a positive F-statistic suggests affinity between X and Pop2, whilst a negative value indicates affinity between X and Pop1. Standard errors were calculated with the default block jackknife 5 cM in size. As outgroups for *F*_3_ and *F*_4_ statistics, we either used haploid genotypes from YRI or CHB.

#### Fixation index

We calculated *F*_ST_ using smartpca software v.16000 from the EIGENSOFT package (v.6.0.1)^118^ with the fstonly, inbreed, and fsthiprecision options set to YES.

#### Inference of mixture proportions

We estimated ancestry proportions using qpWave^36,120^ (v.410) and qpAdm^36^ (v.810) from ADMIXTOOLS v.3.0^119^ with the allsnps: YES and inbreeding: YES options. Standard errors were calculated with the default block jackknife 5 cM in size. We used three basic sets of outgroups:

1. OldAfrica, WHGB, Turkey_N, Afanasievo^79^. This set was adapted from Patterson et al.^79^ and used to infer ancestry components from WHGs, EEFs and Yamnaya/Poltavka pastoralists (OldSteppe).
2. YRI.SG, Poland, Finland, Sweden, Denmark, Ireland, Wales, Italy, Spain, Belgium and the Netherlands^68^. This set was adapted from Gretzinger et al.^68^ and used to infer ancestry components from SP individuals in present-day Central and Eastern Europeans.
3. OldAfrica, WHGB, Russia_Ust_Ishim.DG, CHG, EHG, Iran_GanjDareh_N, Israel_Natufian_published, Jordan_PPNB, Laos_Hoabinhian, OldSteppe, Turkey_N, Russia_MA1_HG, Morocco_Iberomaurusia. This set was adapted and modified from Antonio et al.^34^ to investigate the formation of the SP gene pool.

To analyse potential sex bias in the admixture process, we used qpAdm to estimate SP admixture proportions on the autosomes (default option) and on the X chromosome (option “chrom: 23”) using the abovementioned outgroups. Following the approach established by Mathieson et al.^121^, *z*-scores were calculated for the difference between the autosomes and the X chromosome using the formula ${\rm{z}}=\frac{pA-pX}{\sqrt{\sigma {A}^{2}+{\sigma X}^{2}}}$ where *pA* and *pX* are the SP admixture proportions on the autosomes and the X chromosome, and *σA* and *σX* are the corresponding jackknife standard deviations^121^. Thus, a negative *z*-score means that there is more SP admixture on the X chromosome than on the autosomes, indicating that the SP admixture was female-biased.

#### Ancestry decomposition

We performed model-based clustering analysis using two different approaches in ADMIXTURE:

1. We applied ADMIXTURE^41^ in unsupervised mode using modern and ancient individuals at *K* = 2 to 12. Variants with minor allele frequency of 0.01 were removed and PLINK was used for linkage disequilibrium (LD) pruning with a window size of 200, a step size of 5 and an *R*^2^ threshold of 0.5.
2. We applied ADMIXTURE^41^ in supervised mode using modern reference populations at *K* = 12. This analysis was run on haploid data with the parameter “haploid” set to all (=“*”). To obtain point estimates for populations, we averaged individual point estimates and calculated the s.e.m. As modern references we used the groupings listed in the Supplementary Notes 4.1. The *Q* matrix of this ADMIXTURE analysis was also used as input for FSTruct as described by the authors^116^.

#### Maximum likelihood tree

We constructed maximum likelihood trees using TreeMix (v.1.12)^122^. For each tree, we performed a round of global rearrangements after adding all populations (-global) and calculated 100 bootstrap replicates to assess the uncertainty of the fitted model (-bootstrap). Sample size correction was disabled.

#### Admixture dating

Admixture dates were calculated using DATES (Distribution of Ancestry Tracts of Evolutionary Signals) (v.4010)^56^, which calculates the decay of ancestry covariance coefficients between every pair of available overlapping SNPs between the test individuals and the source populations over increasing-genetic-distance window. We used standard settings with default bin size of 0.001 Morgans applied in our estimates (flag “binsize: 0.001”). We used a standard of 29 years per generation to convert the generation times in years since admixture.

#### Language dating

We use Bayesian phylogenetic inference to estimate the ages of language divergence as described^78^. Analyses were performed on the IE-CoR database that stores data on cognate relationships (shared word origin) between 161 Indo-European languages, in a reference set of 170 basic meanings (https://iecor.clld.org). Divergence date distributions for the Balto-Slavic and Slavic subgroups were extracted from the sample of 37,004 trees resulting from the main analysis of Heggarty et al.^78^. A critical evaluation of this approach is discussed in Supplementary Note 9. However, we highlight that these computational estimates for the splits of the Baltic and Slavic languages compare well with estimates produced by methods of traditional Indo-European linguistics. These estimates are based on the reconstructed rapidity of diversification relative to that observed in the other branches of Indo-European (some of which is recorded in writing thousands of years before Baltic and Slavic), the nature of contacts and convergence between Balto-Slavic and other languages (IE and non-IE), and the character of the reconstructible Proto-Balto–Slavic vocabulary—but also attempted links to the archaeological picture^57^. The dating of the split of Proto-Balto–Slavic is generally in agreement with the results of traditional historical linguistics as described above; and earlier glottochronological approaches have yielded similar dating—for example, an estimation at the fifteenth and fourteenth century bce^123^.

#### Software

All software used in this work is publicly available. List of software and respective versions: AdapterRemoval (v.2.3.1), Burrows–Wheeler Aligner (v.0.7.12), DeDup (v.0.12.2), mapDamage (v.2.0.6), BamUtil (v.1.0.14), EAGER (v.1), Picard tools (v.2.27.3), Sex.DetERRmine (v.1.1.2) (https://github.com/TCLamnidis/Sex.DetERRmine), ANGSD (v.0.915), Schmutzi (v.1.5.4), PMDtools (v.0.50), pileupCaller (v.1.4.0.2), samtools (v.1.3.1), Geneious (R9.8.1), HaploGrep 2 (v.2.4.0), READ (https://bitbucket.org/tguenther/read) (vf541d55), KIN (v.3.1.3), lcMLkin (https://github.com/COMBINE-lab/maximum-likelihood-relatedness-estimation) (v.0.5.0), BREADR (https://github.com/jonotuke/BREADR) (746316 f), PLINK (v.1.90b3.29), smartpca (v.16000; EIGENSOFT v.6.0.1), qp3Pop (v.435; ADMIXTOOLS v.3.0), qpDstat (v.755; ADMIXTOOLS v.3.0), qpWave (v.410), qpAdm (v.810), DATES (v.4010), ADMIXTURE (v.1.3), TreeMix (v.1.12), GLIMPSE (https://github.com/odelaneau/GLIMPSE) (v.2.0.0), BEAGLE (v.5.4), RefinedIBD (v.17Jan20.102), FSTruct (https://github.com/MaikeMorrison/FSTruct) (d39827e), hapROH (v.0.6), ancIBD (https://pypi.org/project/ancIBD) (v.0.4), GLIMPSE (https://github.com/odelaneau/GLIMPSE) (v.2.0.0), MOBEST (https://github.com/nevrome/mobest.analysis.2022) (v.26f929e). Data visualization and descriptive statistical tests were performed in R (v.4.1.1). The following R packages were used: Rsamtools (v.2.12.0), vegan (v.2.6-2), factoextra (v.1.0.7), ggplot2 (v.3.3.6), ggExtra (v.0.10.0), ggforce (v.0.3.3), rnaturalearth (v.0.1.0), sf (v.1.0.-8), raster (v.3.5-21), rgdal (v.1.5-32), spatstat (v.2.3-4), maptools (v.1.1-4), gstat (v.2.0-9), sp (v.1.5-0), labdsv (v.2.0-1), rcarbon (v.1.5.1), magrittr (v.2.0.3), dplyr (v.1.0.9), reshape 2 (v.1.4.4), and tidyverse (v.1.3.2). Y-chromosome and mtDNA haplogroups were determined using the ISOGG SNP index (v.15.73) and PhyloTree (v.17-FU1) reference databases, respectively.

### Reporting summary

Further information on research design is available in the Nature Portfolio Reporting Summary linked to this article.

## Online content

Any methods, additional references, Nature Portfolio reporting summaries, source data, extended data, supplementary information, acknowledgements, peer review information; details of author contributions and competing interests; and statements of data and code availability are available at 10.1038/s41586-025-09437-6.

## Supplementary information

Supplementary information Supplementary Notes 1–9, including Supplementary Figs. 1–68 and additional references.

Reporting Summary

Supplementary Data Supplementary Data including Supplementary Tables 1–51.

## Data Availability

Unmapped, raw sequencing data (fastq files) from the newly reported ancient individuals will be available on publication from the European Nucleotide Archive under accession number PRJEB81250. A poseidon package of the genotype data analysed in this paper is available on the Poseidon Community Archive (https://www.poseidon-adna.org/#/archive_explorer). Previously published genotype data for ancient and present-day individuals was reported by the Reich laboratory in the Allen Ancient DNA Resource v.54.1 (https://reich.hms.harvard.edu/allen-ancient-dna-resource-aadr-downloadable-genotypes-present-day-and-ancient-dna-data). The Genome Reference Consortium Human Build 37 (GRCh37/hg19) is available via the National Center for Biotechnology Information under accession number PRJNA31257. The revised Cambridge reference sequence is available via the National Center for Biotechnology Information under NCBI Reference Sequence NC_012920.1. Published genotype data for the present-day British sample are available from the Wellcome Trust Case Control Consortium (WTCCC) via the European Genotype Archive (https://www.ebi.ac.uk/ega/) under accession number EGAD00010000634. Published genotype data for the present-day Irish sample are available from the WTCCC via the European Genotype Archive under accession number EGAD00010000124. Published genotype data for the present-day Lithuanian sample are available from https:// figshare.com/articles/Patterns_of_genetic_ structure_and_adaptive_positive_selection_in_ the_Lithuanian_population_from_ high-density_SNP_data/7964159. Published genotype data for the Dutch sample are available by the GoNL request process from The Genome of the Netherlands Data Access Committee (DAC) (https://www.nlgenome.nl). Published genotype data for the rest of the present-day European samples are available from the WTCCC via the European Genotype Archive under accession number EGAD00000000120 and from the Estonian Biocentre (https://evolbio.ut.ee/).
