# Supplementary material for: Ancient DNA connects large-scale migration with the spread of Slavs
Source: Nature. 2025 Sep 3;646(8084):384–93. doi: 10.1038/s41586-025-09437-6 (PMC12507669; doi:10.1038/s41586-025-09437-6)
Supplement: Supplementary file 1 — Supplementary Notes 1–9, including Supplementary Figs. 1–68 and additional references. [file 41586_2025_9437_MOESM1_ESM.docx]

## Supplementary Notes

[**Supplementary Notes 1**](#_1ctvd6xmosfn)

[**1. Supplementary introductions 3**](#_3j48tw6c9k3q)

[1.1 A historical overview of Slavic expansion 3](#_tg0w3n713n5x)

[Problems of Slavic identity 3](#_2ascgy3g7ka0)

[The Roman Empire and its neighbors 5](#_318jdugnuo6l)

[The Migration Period in East-Central Europe 6](#_8xqynfi6vgyw)

[The emergence of Slavs 8](#_mjytnaudkkid)

[Slavic diffusion and Slavicization 10](#_5f1ldso0ezm)

[1.2 The archaeological debate on Slavic expansion 13](#_dcb0ls1v1sef)

[**2. Site descriptions 16**](#_h2pdb8gjvwk2)

[Croatia 16](#_a20twmqsnit3)

[Biograd - Glavice (BIO) 16](#_81lcqnh1kd73)

[Bribir - Glavica (BRI) 16](#_tryb55hjk10e)

[Bubi's cave (BBC) 18](#_z3yqvavy2xao)

[Dubrovnik cathedral (DUC) 19](#_6q30q8x4222k)

[Dugopolje - Vučipolje (DUG) 20](#_cnoaw8rtcxlx)

[Podvršje - Glavčine (POD) 21](#_tr4a0sbdhprh)

[Torčec - Cirkvišće (TOR) 22](#_28r8vu6r5sin)

[Velim (VEM) 22](#_lwdpb9lw9n8t)

[Vir - Smratina (VIR) 24](#_sp4clmap48li)

[Eastern Germany 24](#_5wpa36jnjhu9)

[Brücken (BRC) 24](#_q1m7o9qft55r)

[Deersheim (DRH) 26](#_q91rh9kol5ok)

[Niederwünsch (NDW) 28](#_j6g1t9uvnetg)

[Obermöllern (OBM) 30](#_shj80yxa4ami)

[Thuringian Period (OBM_MP) 30](#_fl7q6w7ong6c)

[Slavic Period (OBM_SP) 32](#_t56feg7csorr)

[Rathewitz (RTW) 33](#_blemacreufg6)

[Steuden (SDN) 34](#_dshtcikv80b0)

[Latvia 35](#_dqbfyi9ipu4l)

[Laukskola burial site (LAU) 35](#_sv5pjd84itoi)

[Mežīte burial site (MEZ) 36](#_lqwlmgoxgcc6)

[Vampenieši burial sites (VAM & DOV) 37](#_4fi3ij9m5f0h)

[Poland 39](#_v5dpeo9k1ac6)

[Boczne Rockshelter at Ogrojec (PC1) 39](#_btvmlbvxstd9)

[Gródek (GRK_MP & GRK_SP) 39](#_nlmg7la0oy1h)

[Koziarnia Cave (PC1) 42](#_txkohj1k91s8)

[Żarska Cave (PC1) 43](#_evclgeywdzf2)

[Zbójecka Cave (PC2) 43](#_kzxdbjxg7yv4)

[Northwestern Ukraine 44](#_ndzb7ptzl4uf)

[Korolivka/Korolówka (KRW) 44](#_1l8reiyd7tey)

[Pidhirtsi/Podhorce (PDH) 45](#_5c18hz740exp)

[**3. Genetic affinities of the pre-SP/MP populations 46**](#_dwjtfjjcfoo4)

[3.1 Croatia 46](#_iq95nsfw9bnf)

[3.2 Eastern Germany 51](#_emn04enlm43n)

[3.3 Poland 57](#_ph0reeunogtv)

[3.4 Volga-Oka area 61](#_n7y5fyer9iaq)

[**4. Genetic affinities of the SP population 64**](#_gk5nw7oeivy)

[4.1 PCA & ADMIXTURE 64](#_n4qn742ih3n2)

[4.1.1 PCA 64](#_ze2on3xanc0h)

[4.1.2 unsupervised & supervised ADMIXTURE 67](#_3tp2gplfg07)

[4.2 F-statistics 74](#_bmibeh47e07d)

[4.2.1 F4 statistics 74](#_wlrwuod0znf4)

[4.2.2 FST 80](#_j08vr88ddhem)

[4.3 IBD sharing 84](#_8h0y0qd254en)

[4.3.1 Present-day comparisons 84](#_idhaad8hhmg1)

[4.3.2 Ancient DNA comparisons 89](#_96l5b1ehqtfr)

[4.4 Affinities to ancient population 99](#_34yvuf23o002)

[4.4.1 F3 and F4 statistics 99](#_lu9bpi4eof3s)

[4.4.2 Admixture with southern sources. 110](#_gas1rxuavjvg)

[4.4.3 Possible admixture scenarios 119](#_giv8qww36j14)

[4.5 Extended Discussion I 126](#_hux0b9kbn41z)

[**5. Present-day Europe 131**](#_hs5rgrtbk47h)

[**6. Diversity, Admixture and Population size 139**](#_z5cnmw6q3iks)

[**7. Uniparental markers and sex bias 145**](#_auawcbkfpit6)

[7.1 Uniparental markers 145](#_wskh9v386oiq)

[7.1.1 MP 145](#_wdgjssm75778)

[Eastern Germany 145](#_5dt94esvt5is)

[Poland & Northwestern Ukraine 146](#_fc8755rrt09)

[Northwestern Balkan 146](#_etbi1gd63lkn)

[7.1.2 SP 147](#_jnnf74wfo9fx)

[Eastern Germany 147](#_9zyil6f4ocdy)

[Poland & Northwestern Ukraine 148](#_mgcn7da71xru)

[Northwestern Balkan 149](#_pxupuwka61lb)

[7.1.3 Extended Discussion II 149](#_lstkvsnnpf7r)

[7.2 Sex bias 155](#_rglow33swdy6)

[**8. Changes in social structure 157**](#_1o34rq2gi33w)

[8.1 Grave goods 157](#_mzz9k6ihvevj)

[8.2 Spatial Structure 161](#_txjcmlx440p9)

[8.3 Relatedness 167](#_mnd9awfxn426)

[**9. Debates about the emergence of Slavic language and languages 171**](#_hislgzoryzpe)

[**References 173**](#_51hxjwow6s5j)

## 1. Supplementary introductions

### 1.1 A historical overview of Slavic expansion

(Walter Pohl)

#### Problems of Slavic identity

At first glance, it may seem obvious that Slavs shared a common language and cultural habitus, and in most cases, also a common origin, both genetically and from some homeland. The modern Slavic nations of Europe each have their own Slavic language and their homeland, where they arrived around the same time, in the second half of the first millennium CE. In this sense, the present article may be taken as confirming this simple model of Slavic identity and commonality. However, at a closer look this model has several weaknesses. First, both the linguistic and the genetic uniformity of modern nations is limited, and many of them have changed their shape and population considerably over the past 100 years or so. Second, genetic and linguistic differences largely grow with increasing distance, but their dynamic often does not coincide: Even in Europe today, languages may differ within genetically similar populations (Hungarians, Rumanians and eastern Germans are genetically rather close to Slavic populations), and recent migration may increase genetic multiplicity within one language group (as in Britain or France). Third, we cannot project notions of relative linguistic unity from modern nations with their mass media and national education systems onto sparsely-populated regions more than 1000 years ago.

Fourth, in the 1^st^ millennium CE, ‘Slavs’, like ‘Germans’, was not an ordinary ethnonym denoting a clearly-identifiable bounded ethnic unit. Both names are almost exclusively transmitted as Roman ethnographic categories describing the population of wide and thinly-populated, “barbarian” spaces beyond the boundaries of civilization. For many centuries, there are no traces of self-identification as “Slavs”. That certainly does not mean that there were none - we lack texts written by Slavs altogether. Yet there is in fact an influential theory in scholarship arguing that the early Slavs only gradually adopted the Roman term for them for self-designation[^1^](https://paperpile.com/c/UPmHk7/GOPpA). That may be going too far, but so is the contrary assumption that proud and self-assured Slavs imposed their name on huge stretches of land between the Volga and the Elbe from the start. The ancient Germans, whose name Julius Caesar had introduced as an ethnographic category in the 1^st^ century BCE, as a rule did not identify as ‘Germani’, but under the names of smaller tribal or ethnic units. In the case of the earliest Slavs, we have fewer particular ethnic names, and some are derived from their settlement area; but again, that may be due to the initially scarce communication between Slavs and Romans.

Fifth, and that is the main research problem, the different types of evidence we have do not add up to a coherent image of Slavic expansion. Since the mid-6^th^ century CE, the written sources offer an increasing number of often precisely-dated perceptions by southern and western neighbors, which are patchy, but can fairly easily be incorporated into a narrative of south- and westward expansion of Slavs, such as the one offered below. Some of the reports in 6^th^-to-8^th^-century texts may be less reliable than others, but as a whole they can hardly be dismissed. They give some clues, but leave much space for debate as to the character of the Slavicisation of much of Eastern Europe: Was it due to migration, expansion or diffusion of Slavs, or to the Slavicisation of regional populations? And they say little about the northern regions of Eastern Europe.

The archaeological evidence can fill that gap, and it has become standard to regard the so-called Prague-Korchak culture as an expression of Slavic expansion, characterized by simple handmade pottery, sunken huts, cremation burials, and a reduced number of durable objects found in settlements and burial sites. Indeed, these criteria enable a clear demarcation from the archaeological cultures of the Roman Empire, of the post-Roman kingdoms and of peoples of the steppe. Yet none of these features are exclusive to Slavs, but are attested in a far wider geographic and temporal range. As Florin Curta has proposed, the regional differences within the supposed Slavic sphere are more substantial than the distinctions with respect to neighboring cultures[^2^](https://paperpile.com/c/UPmHk7/QYHrb). He has also insisted on a second problem: Traces of migration and of the long-distance transfer of recognizable sets of cultural features are scarce, and the supposed Slavic homelands northeast of the Carpathians seem to experience population growth rather than emigration in the period in question. Archaeological debates about these issues face the problem that on many of the sites in question, few objects are characteristic and can be dated (and the sophisticated typologies of minute particularities of Prague-type pots may not offer clear chronological criteria).

A further contradiction arises from the linguistic evidence. The first longer Slavic texts that have been transmitted were written by Christians engaged in Slavic missions in the late 9^th^ and 10^th^ centuries, and they constitute a main body of evidence for linguists who seek to reconstruct the development of a common Slavic language. Later written texts in single Slavic languages, and of course their modern structure and vocabulary, have been used to reconstruct their eventual split. Many linguists have noticed the surprising uniformity (i.e. lack of dialectal diversification) of the Common Slavic language they had reconstructed. Such similarity would imply a clearly-bounded, homogeneous group with intense internal exchanges and communication. The archaeological record provides little evidence for such supra-regional exchanges. Until deep in the 8^th^ century, there seem to have been entire regions in East-Central Europe that were thinly populated or uninhabited, for instance, Eastern Austria or Northern Poland. The only polity that could have promoted the spread of a rather uniform Slavic language was the Avar Empire, and it is an attractive hypothesis that such a language was diffused as a *lingua franca* in its realm.

Now, archaeogenetic results add significantly to this picture. In this paper, we present evidence for an incisive population shift in Eastern Germany, Poland and the Czech Republic, and substantial gene flow from a region northeast of the Carpathians to Southeastern Europe, sometime between the 6^th^ and the 8^th^ centuries. This means that numerous people must have moved in the course of that period. As we hardly have any samples from those critical centuries due to the cremation habit, the genetic results give no information about the time and circumstances under which these north-easterners moved. The results of the present study highlight the long-term impact of the spread of the Slavic-period gene pool, which still determines the genetic profile of many inhabitants of Eastern Europe down to today. However, that does not mean that the process necessarily followed a linear trajectory. The archaeological evidence suggests that different regions in Eastern Europe were affected rather differently and at different times in the 6^th^ and 7^th^ centuries[^2^](https://paperpile.com/c/UPmHk7/QYHrb). In retrospect, we can conceive of it as an expansion of the Slavs. However, which of the groups involved identified or were regarded as Slavs and/or spoke a Slavic language is far from clear. We can hypothesize rather different scenarios how the Slavicisation of Eastern Europe came about.

Can we treat all these inconsistencies in the simple model of a coherent ethnic, genetic, cultural and linguistic Slavic identity as exceptions or outliers? That would have two grave disadvantages. One is the scholarly one: If we “normally” expect that genetic, cultural, linguistic affiliations and self-identification/outside perceptions as a bounded ethnic group should match, we could infer from one to the other or take one as proxy for the other. A person with Northeastern European ancestry in the second half of the 1^st^ millennium CE could then be supposed to have spoken Slavic, and regarded him/herself as a Slav. Such a-priori assumptions would then create a circular argument - the more Slavs we identify in this way, the likelier it would then be to find even more. Such short-cuts are methodologically unsound. They would block the way to studying the different rhythms of cultural, linguistic, ethnic, political Slavicisation and genetic admixture. Recent interdisciplinary and archaeogenetic studies of the Avar Empire in the 6^th^ to 9^th^ century Carpathian Basin have shown that unification of cultural habitus and the fading of ethnic plurality do not move at the same speed as genetic admixture[^3^](https://paperpile.com/c/UPmHk7/RIikQ).

The other disadvantage lies in the ideological baggage that comes with the name “Slavs” and the several instances of ideological and political uses of the term in history and in the present. What they have in common is the emphasis on a common Slavic identity that is mostly geared to particular political uses. That could be linked to the idea of a Slavic empire including all Slavs, often promoted by Russian imperialism. The notion of a common Slavic homeland could also be employed to argue for the pre-eminence of the state that now holds this territory, for instance Poland. On the other hand, the idea that the Slavs were in fact one people could also be employed by Westerners to underline their backwardness, and the ephemeral character of Slavic states and nations. All these uses and misuses rely on an inclusive concept of identity, in which origin, language, culture and political organization have to match. Should this norm not be reached, a power group might feel entitled to use force to terminate what is regarded as “abnormal” - for instance, subdue Slavic groups that claim to have a separate identity, or to evict non-Slavs from Slavic territories (or vice versa). Whatever the results of archaeogenetic research, under no circumstances can they be used to legitimize such ideological constructs.

#### The Roman Empire and its neighbors

In the first half of the 1^st^ millennium CE, Europe was clearly divided in two parts, largely separated by the Rhine and the Danube. To the west and south of this continental divide lay the provinces of the Roman Empire, ruled by a Latin-speaking elite and rather densely inhabited by a very heterogeneous population that had been mixed by high mobility and deliberate or forced migrations[^4,5^](https://paperpile.com/c/UPmHk7/FRUJk+Wv71f). To the north and east of the two rivers, there were thinly-populated lands, whose scattered inhabitants the Romans regarded as “barbarians”, and roughly classed into two ethnographic categories: between the Rhine and Vistula rivers, the *Germani*, the ancient Germans, and in the steppes north of the Black Sea, the Scythians, an ancient name now used as an umbrella term for steppe peoples[^6^](https://paperpile.com/c/UPmHk7/szD37). Both were dreaded as dangerous enemies but also appreciated as courageous warriors: the *Germani* mostly as ferocious foot soldiers and the steppe peoples as expert fighters on horseback[^7^](https://paperpile.com/c/UPmHk7/b8FhQ). Both groups received a lot of attention for their attacks on the Roman Empire, but also as allies and auxiliary troops of Rome. Between the two zones, east of the Vistula and in the forests north of the steppe zone, there were further groups who were noticed less, and with whom communication and conflict were mostly less intense. Among them, there were the Aesti in the Baltic countries and the Venethi to the south of them. From the name of the latter, the Germans derived a general designation for their eastern neighbors, *Wends*, analogous to (and alliterating with) **walhoz*/Welsh/Vlachs for their western neighbors[^8^](https://paperpile.com/c/UPmHk7/FyEkC).

When the Western Roman Empire broke up in the 5^th^ century, both the steppe riders and the Germanic peoples played an important part in the process. The Huns pushed some of the Germanic groups into Roman territory and subdued others, built an empire along the Middle Danube, and under Attila (d. 453) exerted exceptional pressure on the Romans[^9^](https://paperpile.com/c/UPmHk7/KFxeB). After Attila’s death, Hun power collapsed; in due course, they were replaced by the Avars who had arrived in 557/58 from eastern Central Asia and ruled along the Middle Danube from 567 to c. 800[^10^](https://paperpile.com/c/UPmHk7/y6eI6). These two groups of steppe riders had an impact on the decay of Roman power, not least, by setting other groups in motion; but they hardly left any lasting mark on the regions they had governed. Unlike the steppe riders, some of the Germanic peoples – Goths, Vandals, Burgundians, Franks – successfully integrated themselves in Roman provinces, where they gradually built their kingdoms[^11^](https://paperpile.com/c/UPmHk7/uH0Ma). Their kings – Alaric I, Geiseric, Theoderic the Great, Clovis – are still household names in historical narratives. They all played a part in replacing the Western Roman empire and in transforming the Roman order in its provinces[^12^](https://paperpile.com/c/UPmHk7/M6G6C). However, most of their kingdoms were short-lived. Even where the names of the peoples stuck on the state or on the region – France, Burgundy, Lombardy – the Germanic language disappeared, and the Romance-speaking majority population adopted the identity of their former lords. Seen from this angle, the transformation that happened in the Eastern half of the continent from the 6^th^ century onwards is all the more striking: Within a century or two, almost all the lands east of the Elbe and the Adriatic became Slavic[^1^](https://paperpile.com/c/UPmHk7/GOPpA). This process equally affected former Roman provinces, steppe realms and Germanic settlement areas, and turned them into mainly Slavic-speaking countries. The shift was much more complete than in the western countries where the Germanic peoples had built their kingdoms; and yet, it received much less attention in the written sources. This relative scarcity of contemporary reports, as compared to other parts of Europe, makes it hard to describe how so many regions became Slavic, and even harder to explain why that happened.

#### The Migration Period in East-Central Europe

In the middle of the 1^st^ century BCE, Julius Caesar had introduced into Roman ethnography and political terminology the umbrella term Germani for the population that lived east of the Rhine. Whether or not that corresponded to any self-identification remains unclear[^13^](https://paperpile.com/c/UPmHk7/RwPWt). The Romans soon noticed that this population consisted of numerous tribes and peoples. Around the turn of the Common Era, the armies of Augustus failed to conquer the Germanic lands between the Rhine and the Elbe, and the Rhine remained the frontier of the Empire. However, being the neighbors of the Romans affected these groups in manifold ways. Rome interfered in conflicts within and between Germanic tribes, played them off against each other and sought to install friendly kings over them. The Empire attracted ambitious young men as soldiers in Roman service, resettled defeated groups as dependent peasants on Roman territory, and organized or tolerated slave hunts among barbarian populations. Along the frontier, that led to the formation of larger confederacies that gradually developed into peoples, such as the Franks and Alamanni on the right bank of the Rhine from the late 3^rd^ century onwards. Farther away from the Roman frontier, a greater number of names are attested in the 3^rd^ and 4^th^ centuries, whose movements and whereabouts are not easy to trace, and who seem to have repeatedly regrouped[^14^](https://paperpile.com/c/UPmHk7/Y045i). Among others, we hear of Angles and Saxons close to the North Sea, Warni and Rugi to the east of them, Hermunduri, Thuringians and probably Longobards in the Elbe-Saale region, Burgundians and Juthungi along the upper Main. Over time, Roman imports, career opportunities in the imperial army, occasional raiding and competition between different groups transformed these “barbarian” societies and increased the social weight of professional warriors, whose position depended on a peaceful or violent transfer of resources from the Roman Empire into Germania.

Around 400, this balance shifted. This was partly due to the gradual social changes in barbarian societies described above, but was triggered by a dynamic set off elsewhere by the arrival of the Huns north of the Black Sea in 375, by the resulting flight of the Goths into the Empire and towards the west, and by the increasing political instability within the Roman Empire[^9,15^](https://paperpile.com/c/UPmHk7/KFxeB+ZMgez). In the 400s, Vandals, Alans and Suebi from north and south of the Carpathians marched into Gaul, Spain and later Africa, and Burgundians threatened the Rhine frontier. In the 410s, Saxons, Angles and Jutes began to move across the North Sea to Britain. From the 430s to the 450s, many groups were drawn into Attila’s armies that attacked the Balkan provinces, Gaul and Italy, or were drafted by the Romans against the Huns. Franks settled in northeastern Gaul, often by agreement with imperial authorities. The almost ceaseless power struggles in the Western Roman Empire drew many barbarian warriors into Italy and Gaul. In 476, an officer of the Roman army with a barbarian background staged a coup and ousted the last Western emperor. The success of the Burgundian, Gothic and Frankish kingdoms in the second half of the 5^th^ century constituted a new pole of attraction for ambitious warriors. In the late 5^th^ century, Longobards appeared at the Danube and eventually formed a kingdom in Pannonia.

In the Elbe-Saale region and the lands around it, we can assume that in the course of these events the population decreased, although to a lesser degree than farther east. Archaeological evidence from the 5^th^ century indicates some clear social changes. While in the Lower Elbe region to the North older cremation practices persisted, cremation and inhumation burials oriented north-south were largely replaced in the Elbe-Saale area by west-east inhumation burials, often furnished with signs of status, such as jewelry in female and weapons in male graves. These weapons were partially influenced by Roman and East Germanic or Hunnic-associated types. The traditional long houses were replaced by smaller wooden buildings, and the quality of local crafts decreased. It seems that traditional hierarchies eroded, and displays of status and military masculinity may have reflected the emergence of a new and more precarious social order[^11,16,17^](https://paperpile.com/c/UPmHk7/uH0Ma+3XRJl+jH63f). The present paper adds a lot to our knowledge about the communities of the period in the Elbe-Saale-region, in which funerary displays of status did not depend on origin, ancestry or relatedness. These communities were constituted by cohabitation, and only rarely by networks of biological relatedness. Even people of southern background, most likely women and children abducted during raids into Roman provinces and their offspring, were buried with similar signs of status.

Politically, the second half of the 5^th^ century offered new opportunities for the warrior elites that had remained in Germania. In the course of the 5^th^ century, larger ethnic groups and regional units emerged. After the fall of the Hun Empire in 454, several new regional kingdoms were established in the Carpathian Basin, most of them short-lived. The only one that maintained its rule well into the 6^th^ century was the realm of the Gepids along the Tisza River; many others migrated into the core regions of the Empire[^18^](https://paperpile.com/c/UPmHk7/n8NGj). In 567, the Longobards ended a long conflict by destroying the Gepid kingdom, and collected a multi-ethnic army that moved into Italy in 568, where they established their kingdom. At the same time, the Avars, who had arrived in Europe from Eastern Central Asia only a decade earlier, occupied the entire Carpathian Basin[^10^](https://paperpile.com/c/UPmHk7/y6eI6).

In the Elbe-Saale region, a Thuringian kingdom was established during the middle of the 5^th^ century that soon extended its control over the neighboring regions, integrating people of different ethnic origins[^19^](https://paperpile.com/c/UPmHk7/aikft). In the early 6^th^ century, its royal family became Christian, and was regarded as a match for its more powerful neighbors. King Herminafrid married a daughter of the Ostrogothic king Theodoric the Great and gave his sister in marriage to the Longobard king Wacho, forming a defensive alliance against the Franks. Yet the resources at the disposal of the Thuringians did not match those available on former Roman territory, and in 531 the Franks overwhelmed their realm. The Frankish kings succeeded where Augustus had failed, and established some control over the lands between Rhine and Elbe[^20^](https://paperpile.com/c/UPmHk7/1KaGe). This region was subsequently governed from the 6^th^ century onwards by the newly formed duchies of the Thuringians, the Alamanni and the (newly emerged) Bavarians, while the Saxons in the north formed a loose confederacy – most of them more or less under Frankish suzerainty. Ultimately, from about 630 onwards, Frankish counts were appointed to offices in Thuringia. The initiative had clearly passed from the inhabitants of the areas beyond the former Roman frontier to those who now ruled over Roman provinces – and to new groups the Romans had not heard of before the mid-6^th^ century: Avars and Slavs.

#### The emergence of Slavs

People identified as Slavs are first mentioned by two historians writing in Constantinople soon after 550: Jordanes, whose “*Getica*” are a history of the Goths written in Latin, and Procopius, whose Greek history of Emperor Justinian’s wars also recounts the Gothic War (535-554), in which the East Romans conquered Italy. Both works refer to Slavic incursions in the Balkan provinces from the 530s, and both attach some ethnographic remarks, which show that knowledge about the new enemy was as yet limited, and that both authors sought to link these *Sclaveni*, as they called them, to previously known peoples. The works of ancient authors have to be subjected to source criticism, and assessed for intention, access to information and literary models[^21^](https://paperpile.com/c/UPmHk7/7USVJ).

Jordanes[^22^](https://paperpile.com/c/UPmHk7/n61Do) came from a Alan-Gothic family of Roman army officers serving on the Lower Danube frontier, where they surely had up-to-date information about potential enemies across the border; and he used a now-lost Gothic history by the Roman senator in Ostrogothic service, Cassiodorus. He mentions that the 4^th^-century Gothic king Ermanaric, who ruled north of the Black Sea, subdued the *Venethi*, from whom three peoples of Jordanes’s day have sprung, *Venethi*, *Antes,* and *Sclaveni* (Getica 23.119). In an earlier passage (Getica 5.34), *Sclaveni* and *Antes* are presented as parts of the *Venethi,* who live north of the Carpathians, beginning at the source of the Vistula River. The *Sclaveni*, Jordanes says, live between “the city of Novietunum and the lake called Mursianus” (most probably, north of the Lower Danube from Mursa, today’s Osijek in Croatia, to a place close to its estuary) to the Dnestr and to the Vistula in the north, while the *Antes* settle between the Dnestr and the Dnieper. These, he says, are only the principal groups of a wide-ranging population “whose names vary now by family and place”. For Jordanes, the Slavs were only a recently-emerged part of a broader population that he classes as “Venethi”. Greek sources of the period hardly use the name Venethi. Later on, “*Winedi*” was a German term for the Slavs, surely derived from the Venethi, whom Tacitus (Germania c. 46) already mentions as the eastern neighbors of the Germani in c. 100 CE. It is possible that Jordanes, who was of Gothic origin, already used this Germanic designation for their eastern neighbors here. His account has been the object of much scholarly debate. However, his localization of Slavs is not implausible for c. 550 CE; Slavic incursions could indeed be expected all along the Lower Danube; their habitat could well have stretched northward to the forest zone between the Carpathians and the Dnestr, where the Antes lived. Jordanes reflects a situation in which the Slavs represented only part of a larger population, the one who lived north of the Danube, closest to the Byzantines. He uses the traditional name Venethi, already attested in Tacitus’ *Germania*, for those north of the Carpathians. His account indicates that at the time, there was no clearly circumscribed entity called “Slavs”, but rather, three related groups whose exact relationship and localisation was as yet unknown in Constantinople.

The second author is Procopius[^23^](https://paperpile.com/c/UPmHk7/l81UN), who wrote his history of the Gothic War in part from lived experience as an advisor to the East Roman commander Belisarius. The earliest date at which he mentions Slavs is after the destruction of the kingdom of the Heruli in c. 508, when one part of them decided to return and sent messengers to their ancient homeland on the island of Thule (probably in modern Denmark), touching “all the tribes of the Sclaveni” and then “crossing a large tract of deserted land” in the course of their journey (Wars 6.15). Whether that is a mid-6^th^ century perception or an early-6^th^ century attestation remains unclear. In 537, a cavalry troop composed of Huns, Antes and Slavs took part in the siege of Rome (5.27); otherwise, Slavs in the Roman army, and Slavic horsemen are rarely mentioned in the period. In fact, Procopius ascribes to Slavs and Antes a “Hunnic character in all its simplicity” (7.14.29). Slavs at the margins of the Carpathian Basin appear in c. 550 as armed supporters of an unsuccessful Longobard pretender for the throne; they may have come from north of the Lower Danube, or also from the foothills of the Carpathians (7.35). Who the Slavs were clearly was an issue in mid-6^th^-century Constantinople, as both texts show, and answers were tentative, building both on reliable information and ethnographic stereotypes that supported the mental mapping of new peoples by comparing them with familiar ones.

In the wars of the Eastern Romans against the Avars, Slavs are repeatedly attested in Avar service, often as boatmen in dug-outs helping to cross rivers or attacking from the water. At this point, some must have lived in the Carpathian Basin, where they fought against Byzantine armies attacking the Avars in the 790s. The historians of the period, Menander and Theophylact Simocatta, also provided extensive accounts of Slavic raids in the Balkan provinces that intensified in the 580s, and of Byzantine counter-attacks north of the Lower Danube, where Slavs feature as experts in amphibian warfare or withdraw to forests and wetlands if attacked. A military handbook dated to c. 600, the co-called *Strategikon* of Maurice, devotes one of four sections about Byzantium’s chief enemies to the Slavs[^24^](https://paperpile.com/c/UPmHk7/r1yUA).

Elsewhere, Slavs were only noticed towards the end of the 6^th^ century. A letter by Pope Gregory I written in 599/600 provides a secure date for a Slavic raid in Istria, and thus both for their presence east of the Adriatic, and the appearance of the hitherto unattested name “Slavs” in Italian texts. The late-8^th^ century *History of the Longobards* by Paul the Deacon speaks of Slavs in the Eastern Alps around the same time, pushing as far as Aguntum/Lienz in what is now Eastern Tyrol – information probably derived from the *Historiola* of Secundus written in c. 610. In their wars against the Bavarians, they were supported by the Avar khagan. This information corresponds to the historical and archaeological indications that the late Roman sites in southern Noricum were all abandoned around the end of the 6^th^ century. *The Histories* by Bishop Gregory of Tours finished in c. 590 do not mention Slavs. The next major historical work produced in the Frankish kingdoms, the so-called Fredegar Chronicle, a compilation concluded c. 660, mentions Slavs also called Wends in the western periphery of the Avar Empire, probably in Bohemia. In the 620s, with the help of a Frankish trader called Samo, they freed themselves from the Avar yoke and made him king. In c. 630, they also defeated the Frankish king Dagobert, which encouraged the Sorbs, who according to the text had paid tribute to the Franks for a long time, to secede from Frankish overlordship, and attack the Thuringian duchy.

In the course of the 7^th^ century, Slavs also settled in the Roman provinces of the Balkan Peninsula, which they had begun to raid in the 530s[^25^](https://paperpile.com/c/UPmHk7/x791X). The chronology of these settlements is debated, but in spite of extensive Avar and Slavic raids, the Romans still exercised sufficient control of this territory to be capable of staging several counter-offensives north of the Danube until 602, when an army dispatched to spend the winter in Slavic lands in what is now Walachia mutinied and overthrew the emperor Maurice. In the 610s, most urban centers in the Balkans were destroyed in Avar and Slavic raids, apart from some coastal strips that remained under Roman control[^10^](https://paperpile.com/c/UPmHk7/y6eI6). Regional Slavic settlement units under their own kings emerged more clearly in the second half of the 7^th^ century, often under a treaty with the imperial authorities[^1^](https://paperpile.com/c/UPmHk7/GOPpA). In the 680s, Turkic-speaking Bulgar horsemen began to subdue many of these Slavic groups and established a sizable steppe realm in the eastern parts of the Balkan Peninsula and north of the Lower Danube.

#### Slavic diffusion and Slavicization

The written sources, then, yield a number of traces of Slavic south- and westward movements in the course of the 6^th^ and early 7^th^ centuries. Most of these reports are connected with military activities, often in conjunction with Avar interventions. There is, of course, a double bias in these texts: First, they mostly deal with the zone of interest to the East Roman Empire, and a little later to the Frankish and Longobard kingdoms. And second, they mostly focus on military encounters and similar headline news, rather than on peaceful and long-term changes of population. One of the few exceptions is a story reported by Theophylact Simocatta (Historiae VI.2) for the 590s about a small group of traveling Slavs encountered by a Roman troop[^26^](https://paperpile.com/c/UPmHk7/BJR5h). They were carrying musical instruments but no weapons, and claimed that they lived by the shores of the western ocean (the Baltic Sea), and had been sent to apologize to the khagan of the Avars that they could not send a contingent to his army for lack of military abilities. Whatever the factual basis of this story was, it shows that when Theophylact wrote in the 640s, the East Romans were aware that Slavs lived as far away as the Baltic Sea, and that many of them might be quite peaceful. Some scholars argue that this account cannot be taken as independent evidence of the Slavs’ presence on the Baltic but only as evidence for the survival of ancient ethnography in the 6^th^ and 7^th^ centuries (which often attributed extraordinary qualities to people living on the fringes of the known world)[^27^](https://paperpile.com/c/UPmHk7/2WxCK). However, the description in the Strategikon also offers alternative perceptions to the image of ruthless Slavic plunderers. If they cannot get ransom for their captives, it says, they invite them to stay in their community as free members. The text also warns officers not to trust former Romans who live in Slavic communities and offer their services; they might now be loyal to the enemy and lead the Roman contingent into an ambush. These and other passages may represent traces of Slavicisation of former inhabitants of Roman provinces.

What also becomes clear from the written sources is that most early Slavic communities had relatively little social differentiation, division of labor and weak or no hierarchies. The archaeological record shows hardly any signs of representation of status or identity, neither in the settlements nor in funerary ritual[^28^](https://paperpile.com/c/UPmHk7/ES1FT). This also meant much less extraction of surplus than in the western kingdoms, and even more so in the East Roman Empire, and must have made this an attractive model. Over time, social differentiation set in, especially in regions exposed to influences from more status-oriented societies. North of the Lower Danube in the 6^th^ century, from where large bands of raiders came, commanders and regional leaders are attested, and fibulae and further signs of status can be found, which are absent from most other areas of Slavic settlement[^1^](https://paperpile.com/c/UPmHk7/GOPpA). Unfortunately, for the period after 600 information about Slavs becomes patchier in Byzantine historiography, whereas it gradually becomes more frequent in the West. In Bohemia, a contact zone to the Frankish kingdoms, fortified hilltop settlements are attested in the 7^th^ century, both in written and archaeological sources. In 8^th^-century Carantania, in the Eastern Alps, Slavic dukes were Christianized, and leaders were buried with a mix of Avar and Frankish prestige goods[^29^](https://paperpile.com/c/UPmHk7/AJsxv). That was not a steady process, but depended on conditions in the respective region. In the 9^th^ century, regional lordships had formed in many areas inhabited by Slavs. Yet they rarely strove to expand by subduing other Slavic groups; rather, they were still vulnerable to interventions by foreign powers that had more resources at their disposal - Franks, Eastern Romans or steppe peoples.

Stable Slavic powers emerged only gradually in the course of the 9^th^ and 10^th^ centuries, and Christianization usually went along with the establishment of a hierarchy after the Western or Byzantine model: Moravia and Croatia in the 9^th^, Bohemia, Poland and the Rus’ in the 10^th^ century[^30^](https://paperpile.com/c/UPmHk7/iTVIC). Remarkably, a zone roughly along Elbe and Oder, and between the Baltic Sea and Bohemia (in today’s northeastern Germany and western Poland) retained a more tribal and stubbornly pagan structure until the 12^th^ century, although it was now surrounded by the Christian Roman Empire and its Saxon Duchy to the west, and by the Christian Polish monarchy of the Piast dynasty to the east. Niederwünsch lay west of this deliberately traditionalist zone close to Saxon strongholds and ecclesiastical centers such as Merseburg, where a bishopric was founded in 968 among a still partly pagan population. The southern Elbe Slavic area, and with it the Elbe-Saale region, had been under the influence of the Frankish Empire since the 9^th^ century and was subjugated in the Ottonian period, becoming a stable part of the East Frankish and German-Roman Empire from around 1000. The high degree of northeastern European ancestry that we found may partly be due to the Christian ban on marrying pagans, preventing or decelerating admixture events prior to the Christianization of the area.

The complex historical evidence briefly summarized above, and the archaeological record set out in more detail below, have so far been interpreted in diverse, often contradictory ways[^1,30^](https://paperpile.com/c/UPmHk7/iTVIC+GOPpA). Late antique and medieval authors relied on origin legends that often linked new peoples with previous ones with a similar name or way of life. In the 19^th^ century, the national revival in Slavic studies used a similar model to reveal the ancient and noble past of Slavic nations, and link them by an ancient Slavic brotherhood. In the romantic period, the first half of the 19^th^ century, the shared language and the ancient epics and poems written in this idiom were regarded as an expression of the innermost soul of a people, a pure and simple model for moderns to follow. Therefore, linguists tried to decrypt the origin of the people by discovering the most ancient names of rivers and mountains, or the oldest words for types of vegetation[^31^](https://paperpile.com/c/UPmHk7/g8tSj). Slavicists could never agree where that ancient homeland of the Slavic language actually was; the Pripjet Marshes or the Northern Carpathian foreland were among the favorites. Toward the end of the 19^th^ century, the essence of a people was often sought in its “racial” traits; yet that played a more important role in German nationalism than in the search for Slavic identity.

The historical narrative that corresponded to all these paradigms was the expansion of well-defined and circumscribed peoples that wandered or expanded from an original homeland, and conquered a new country (the case of the Hungarians) or settled in empty lands (more frequent in the origin narratives of Slavic peoples). This interpretation could rely on the legends transmitted in many medieval chronicles, and was easy to communicate to a wide audience. Critique of this model began in research on the Germanic peoples of the Migration Age after 1945 in the works of Reinhard Wenskus and Herwig Wolfram[^14,32,33^](https://paperpile.com/c/UPmHk7/SJ7Sd+Y045i+HNqRw). The written sources allowed tracing a complex process of ethnogenesis in which ethnic groups formed, while their composition shifted repeatedly. It became clear that methodological caution was needed in integrating historical, archaeological and linguistic material, because the groups constituted on the basis of the evidence from one discipline did not necessarily correspond to those of another.

This critical approach was eventually also applied to Slavic studies. It turned out that the search for an “original homeland” was not the most interesting question to ask, and perhaps not a meaningful at all. “Becoming Slavs”, and eventually, becoming Bohemians, Croats or Poles should rather be regarded as a process, in which identification as Slavs, material culture and Slavic language increasingly converged[^25^](https://paperpile.com/c/UPmHk7/x791X). Florin Curta pushed the critique one step further by questioning whether a shared Slavic identity actually existed from the start, and when it mattered to whom[^1^](https://paperpile.com/c/UPmHk7/GOPpA). He assumed that “Slavs” was first of all a categorization used by the Eastern Romans to describe a new type of enemy - a Roman invention rather than a clearly circumscribed group identity. Curta’s extensive work has triggered valuable debates, and has helped much to dismantle circular arguments in which written, archaeological and linguistic pieces of evidence were used to create a linear model of a “Slavic migration”[^2^](https://paperpile.com/c/UPmHk7/QYHrb). Yet for many scholars, he has pushed the deconstruction of “the early Slavs” too far. The 6th-century authors who mention Slavs may have had an agenda, and insufficient information about their exact localisation and ways of life; some may also have projected information from the time in which they wrote into the past they described. Historical source criticism is well equipped to deal with such distortions. One can reasonably discuss whether Slavs appeared in the different regions of eastern Central Europe a few decades earlier or later. Following Jordanes, it would also be attractive to assume that the name Slavs originated among those who lived north of the Lower Danube, and then spread northward. However, it is unlikely that they were locals who had recently become Slavs.

Now, the results of the present paper make it hard to deny substantial movements from a region between the Baltic countries and the northeastern foreland of the Carpathians towards the Elbe, the Adriatic and the Upper Volga. However, this does not mean that the traditional narrative of an expansion of “the” Slavs has now received confirmation. In the vast region from where the ancestry of the Slavs came according to the archaeogenetic results, we have no indication that a people called “Slavs” existed. Furthermore, as Florin Curta has emphasized, they did not transfer from any homeland a distinct “Slavic culture” as defined by archaeologists in post-6^th^ century East-Central Europe. Neither can we be sure who exactly in the region of origin spoke the common Slavic language that can be reconstructed from much later written evidence. “The Slavs” were the result, not the precondition of the movements from the northeast.

Although these movements could be called “migrations” by many current definitions, it is preferable to avoid this term that may be taken to mean a linear movement by a coherent group from an old to a new homeland. “Expansion” also carries the misleading notion of a given unit that acquires new settlement areas. What the written sources tell us about Slavic movements in the 6^th^ century is mostly more haphazard, and often happens under foreign leaders, most of all the Avars; it seems to have responded much to external stimuli. Perhaps “(demic) diffusion” comes closest to what we can plausibly assume. The result, however, was in many respects surprisingly coherent: a population called “Slavs” and/or “Wends” by their neighbors, with a very homogeneous common Slavic language and some cultural features shared not by all, but by many Slavs. Some of that commonality seems to have developed in the course of diffusion, by adjustment and adaptation. Under the difficult climatic conditions of the 6^th^/7^th^ century and after a devastating pandemic, the Slavic way of life seems to have been well suited for survival[^34^](https://paperpile.com/c/UPmHk7/ryq1F). Yet many of those who began to fill the almost empty spaces of eastern Central Europe must have had something in common before that: not least, as we now know, a considerable degree of shared genetic ancestry.

Genetic results can tell us little about the processes by which that came about. However, we already have considerable fine-grained data about regional differences in the degree of admixture and the impact of the diffusion of Slavs. From later evidence, we also learn something about the social practices by which regional Slavic communities formed. Instead of engaging in a renewed polemic between old paradigms and set-piece solutions for the many questions connected with the Slavicization of Eastern Europe, the results of our research can be used to reconstruct a much more differentiated picture of an essential shift in the population history of an often-neglected part of Europe.

### 1.2 The archaeological debate on Slavic expansion

(Felix Biermann)

Archaeologists associate the early Slavs in Central and Eastern Europe with a widespread cultural phenomenon, defined by handmade, undecorated pottery, the custom of cremation burial (usually in urns) and mostly rather small settlements characterized by pit houses or ground-level blockhouses. This archaeological horizon is predominantly referred to as the Prague Group or Prague-Korchak Group[^35,36^](https://paperpile.com/c/UPmHk7/qtBso+IrXn3); in the north of the West Slavic region, the Sukow or Sukow-Szeligi Group is used to describe a slightly more recent variant of the same phenomenon[^37^](https://paperpile.com/c/UPmHk7/myi8o). Other cultural phenomena associated with the early Slavs, such as the Penkovka and the Kolochin groups, are not of major relevance in the context of this study due to their areas of distribution[^35,38^](https://paperpile.com/c/UPmHk7/qtBso+prnsU).

The Prague group and its offshoots can be dated to the 6^th^-8^th^ centuries. However, the chronological classification is often not easy because there are only a few well-dated finds – such as coins or elite material culture – among the relics of the early Slavic period, in which rather simple economic and social conditions prevailed. Radiocarbon dates are therefore important, although they are imprecise and prone to error. Only dendrochronological data, which is usually obtained from wood preserved in groundwater from wells, provides a precise and reliable chronological classification. Wooden wells were excavated in many early Slavic settlements[^39^](https://paperpile.com/c/UPmHk7/GUKHc). This method has revolutionized chronological ideas about the Early Slavic period in many regions. It quickly became apparent that the Early Slavic culture did not appear everywhere at the same time, but with major chronological differences. While it can be dated to as early as the 6^th^ century in Romania and Slovakia (also on the basis of written information about the Slavic settlement there), to the 6^th^/7^th^ centuries in Bohemia (esp. Prague-Roztoky, ^14^C: 560-645 CE), to the first half of the 7^th^ century in the case of Lesser Poland (dendrochronological dating of the settlement at Kraków Wyciąże, site 5B – 628-635 CE), it cannot be documented before the second half of the 7^th^ century in Northern Poland and Northeastern Germany[^40^](https://paperpile.com/c/UPmHk7/tSbTw). In any case, it is clear that the Prague group or similar cultural phenomena were at the beginning of development wherever we can prove Slavic population in the later Middle Ages through historical or linguistic sources.

On the basis of the early Slavic culture characterized by undecorated pottery, which appears very similar from the Balkans to the Baltic Sea, from the Elbe to the Dnieper, very different developments then took place. The 8^th^ century in particular saw a powerful economic and social development in many regions, for example on the Baltic Sea and in the Danube region, in the course of which there was the formation of rulerships and the building of strongholds, the first proto-urban settlements, an increase in the quality of pottery and other material culture as well as a strong increase in crafts and trade. These developments, which differed from region to region, will not be discussed further here[^41–43^](https://paperpile.com/c/UPmHk7/PA1Ic+eCGn1+X3SQx).

The widespread expansion of early Slavic culture is the subject of controversial debate[^44,45^](https://paperpile.com/c/UPmHk7/w4KVm+ED8le). The change of the cultural model is usually explained by the migration of groups of people, whereby in the course of the history of research both large tribal groups and small associations (for example on the basis of clans) were considered, which gradually penetrated into areas that had lost population during the Migration Period. The Slavic expansion was also repeatedly seen in connection with the invasion of Central Europe by the Avars from the 6^th^ century onwards and the establishment of their large Khaganate. The appearance of Slavic-associated artifacts, dwellings or burial rites in a particular area is explained not only by the immigration of Slavic populations, but also by the adoption of the Slavic cultural model by a pre-existing population. This applies in particular to the south-east European expansion areas, in which the Slavs immigrated to quite densely populated provinces of the Byzantine Empire[^46^](https://paperpile.com/c/UPmHk7/zy9lQ). In the northern regions, such processes of “Slavicisation” are often less plausible to reconstruct because the regions in which we can prove early Slavic settlement were previously only sparsely populated or not populated at all[^47^](https://paperpile.com/c/UPmHk7/Ncpzo). In the north-eastern German region in particular, archaeological evidence, often dated by dendrochronology, points to a longer settlement gap between the Germanic peoples who previously settled there, who can be traced back to the middle of the 6^th^ century, and the Slavs who have been identifiable since the last third of the 7^th^ century. Moreover, there is a complete break in the archaeological cultural model[^48,49^](https://paperpile.com/c/UPmHk7/q3jNX+Nu0hz).

In Bohemia and the Eastern Alps, we often lack archaeological evidence for contacts between Slavs and remains of the previous population, but it can often be assumed. A clear example for such contacts is a bone with runic inscription discovered at Slavic settlement in Lány (Czech Republic). This artifact provides archaeological evidence of contacts between Germanic-speaking people, writing in the runic alphabet (elder futhark), and people associated with the Slavic cultural model[^50^](https://paperpile.com/c/UPmHk7/kiD8w).

In addition to such migration models, there is also the idea that the early Slavic cultural model essentially spread through communication among a sedentary indigenous population. There is even a model according to which the Slavs were formed as a result of their being named by the Byzantines: From the contact areas of the 6^th^ century on the lower Danube, this foreign designation would have spread as self-identification across the inhabitants of large parts of Central and Eastern Europe. The central element of the whole affair, the Slavic language, would have been transmitted and then established in the context of the Avar Khaganate[^2^](https://paperpile.com/c/UPmHk7/QYHrb). However, this argument has many weak points[^51^](https://paperpile.com/c/UPmHk7/PbLin).

If the Slavic expansion can be explained by long-distance movements of people, then there must be a starting area. There is also a controversial debate about this area, formerly known as the “original homeland” (“*Urheimat*”), in which the Slavic language and culture developed. In Poland in particular, it was long argued that the Slavs had developed there since the Bronze Age. This so-called “autochthonist” model developed in the 19^th^ century and became very influential in the period between the world wars, not least due to its many ideological and political uses. It is still advocated in Poland but has long since lost its significance as a mainstream trend[^52^](https://paperpile.com/c/UPmHk7/a5qjk). In fact, the arguments in favor of this model are not very viable[^44,53^](https://paperpile.com/c/UPmHk7/Ut2y7+w4KVm).

In Poland and beyond, the already very old “allochthonist” thesis prevails today that the Slavic language group developed in western parts of Eastern Europe, in the area of Ukraine and Belarus, during the first half of the first millennium. This is supported not only by linguistic research and historical constellations, but also by archaeological cultures from the Roman Iron Age and Migration Periods in this area, which already anticipate characteristics of the Prague culture. The so-called Kyivan culture (in its early phase also referred to as “post-Zarubintsy” culture) of the 2^nd^ to 5^th^ centuries is repeatedly referred to here, which in terms of pottery, house construction and settlement patterns already shows similarities with those early medieval cultural phenomena that we can surely associate with the early Slavs[^38,54,55^](https://paperpile.com/c/UPmHk7/qVkx4+yeaBR+prnsU).

From there, the Slavs began to spread in the course of the Migration Period, initially to the south-west, towards the Byzantine provinces in the 6^th^ century, and a little later also to the west, into eastern Central Europe. The expansion progressed in stages. Its outermost limits were not reached in the north-west (in eastern Holstein on the Baltic coast) until the end of the 7^th^ century. This model has many arguments in its favor and is correspondingly popular but is by no means certain in all aspects[^56,57^](https://paperpile.com/c/UPmHk7/6S0WE+uJLF6).

An even more fundamental discussion concerns the question of whether and to what extent archaeological material culture can be used to draw conclusions about linguistically and culturally defined groups. The long and controversial debate about “ethnic interpretations” cannot be recapitulated in detail here. However, the following can be stated: Similar stylistic and functional characteristics of archaeologically recorded material culture can reflect the dense communication network that connects people due to shared or neighboring settlements, close contacts and, in particular, as a result of a common language. On the other hand, Slavic communities were receptive to the integration of individuals from diverse backgrounds, as substantiated by archaeological, historical, and linguistic evidence.

## 2. Site descriptions

### Croatia

#### Biograd - Glavice (BIO)

Region: Dalmatia

Coordinates: 43.93"N 15.46"E

Occupation period: 1200 CE - 1700 CE

Samples: 1

Sample IDs: BIO001

Sex Ratio: 1F

Provenance note: Samples from Biograd – Glavice were provided by the Croatian Academy of Sciences and Arts. Permission to analyse was granted to coauthor Johannes Krause in 2022.

Contact person: Mario Šlaus

The archaeological site Biograd – Glavice is located on the highest point of the peninsula where the town Biograd na Moru developed, giving it the name "Glavice", or "Head(s)". It is situated approximately 30 km south-east of the town of Zadar on Croatia’s Adriatic coast. The micro-toponym Glavice is situated in an area immediately northeast of the Biograd cathedral of St. Anastasia. The Biograd cathedral is a three-nave, three-apsed basilica with rounded buttresses, similar to structures dated to the second half of the 9^th^ century. Archaeological excavations carried out in 2016 identified the presence of six graves in the Glavice micro-toponym that were located in a 4.5 x 6 meters excavation area. Five of these graves were explored. The complete lack of movable finds makes dating the graves difficult, but their position suggests they are medieval graves dated between the 6^th^ – 15^th^ centuries. No traces of brick structures were found, indicating that either the Biograd cathedral was not located in the surveyed area or it was so thoroughly destroyed in the early 20^th^ century that no foundation traces remain. In anthropological terms, the recovered osteological material is characterized by poor preservation and the fact that one of the recovered individuals, a subadult aged between 5 – 10 years at time of death, exhibits osteological evidence for the presence of tuberculosis[^58,59^](https://paperpile.com/c/UPmHk7/hDR8m+D9VK4).

#### Bribir - Glavica (BRI)

Region: Šibenik-Knin

Coordinates: 43.92"N 15.84"E

Occupation period: 1200 CE - 1700 CE

Samples: 1

Sample IDs: BRI006

Sex Ratio: 1F

Provenance note: Samples from Bribir - Glavica were provided by the Croatian Academy of Sciences and Arts. Permission to analyse was granted to coauthor Johannes Krause in 2022.

Contact person: Mario Šlaus

While life in the broader vicinity of Bribir traces back to the early Neolithic period, the Glavice micro-toponym appears to have been continuously inhabited since the Bronze Age, when the Liburnians, an Illyrian tribe, erected a fortress at this location. Graves dating from this era have been discovered along the road ascending the southern slope of Glavica towards Vratnice, marking the main entrance to the prehistoric settlement. Artifacts from these graves indicate the thriving lifestyle of the Liburnians as well as their extensive interactions with other Mediterranean cultures, primarily Greeks, Phoenicians, and the inhabitants of the Apennine Peninsula. In contrast to neighboring peoples and tribes along the eastern Adriatic coast, the Liburnians engaged with the Romans at an early stage. Their integration into the Roman Empire, initiated around the mid 2^nd^ century BC, was prompted by continual threats from neighboring Greeks at sea and Delmats on land. Under Roman influence, Varvaria evolved into a frontier town against the Delmats, with whom the Romans, supported by allies, engaged in conflict for approximately 150 years until the dawn of the new era.

Excavations across Glavica have unearthed ancient remnants scattered throughout the area. These include the ruins of townhouses and villas, often featuring monumental cisterns. Traces of streets and sewage canals from antiquity are visible in various locations, along with remnants of sacred structures. However, knowledge of late antique period findings from Bribirska Glavica remains limited, with a focus on secular buildings and fortified ramparts.

Recent research revealed an eight-leaf rotunda dating back to the late Antique period, its walls leaning against a late antique tomb containing two sarcophagi. Early medieval architectural remnants are prevalent across nearly every micro-location investigated on Bribir Glavica. Evidence of substantial burning layers in multiple areas suggests a violent demise to the late Antique period, probably during the Slavic migrations in the mid-7^th^ century.

Following the destruction of the Liburnian-Roman horizon, a new early medieval fortified town (castrum) emerged atop the ruins. However, knowledge about secular and Christian structures in Bribir, an important county center till the 12^th^ century, remains incomplete. Bribir Glavica rose to prominence in the 12^th^ and 13^th^ centuries, becoming a well-organized social and political hub of Croatia, particularly under the rule of the noble Šubić family (nobiles/comites/principes Breberienses), who ascended to become the most influential Croatian nobles during this period. Ottoman conquests in the first decades of the 16^th^ century and the subsequent later population movements and ethnic changes interrupted that continuity and separated Bribir from historical developments in the western parts of Croatia.

Cemeteries were discovered in five different locations in Bribir Glavica. The oldest burial grounds lie outside the city ramparts, at Vratnice and Novi Put. Based on jewelry unearthed from these sites, inhumations here likely date to a period from the 9^th^ to the 12^th^ centuries. During the 12^th^ and 13^th^ centuries, cemeteries were relocated within the ramparts of the castrum, near the Church of St. John the Evangelist. These new burial grounds, situated on the western side of the present-day village cemetery, succeeded the older late Antique and early Medieval burials in the immediate vicinity. In the early 14^th^ century, two new cemeteries were established in Glavica, one near the late Medieval Church of St. Stephen, along the southern wall of the village cemetery, and the other next to the Church of St. Mary in the Dol area. These cemeteries remained in use until the fall of Bribir to the Ottomans in 1523. Jewelry recovered from graves in these locations exhibit characteristics of Gothic goldsmithing, including a prevalence of granulated three-strand earrings and cast signet rings sometimes adorned with inset stones. Additionally, remnants of blacksmithing trade items such as iron mushroom-shaped belt buckles, spurs, and a 13^th^ century sword have been found. Notable features of these cemeteries include family-tiered tombs, a novelty in burial practices in Bribir, and massive stone covers over the graves, particularly in the Church of St. Mary. One of these covers, positioned inside the narthex along the north wall of the church, features a relief carving of an angel with narrow wings resembling the coat of arms of the Šubić family. Within the narthex, a gothic capital with similar heraldic symbols suggests the possibility of a mausoleum beneath an arcosolium in that location. The human osteological material was recovered from ossuary type burials that contained a minimum of 158 individuals. In anthropological terms, the recovered osteological material is characterized by a high incidence of skeletal indicators of intentional violence including perimortem traumas, sharp force lesions, craniofacial injuries, and ‘parry’ fractures to the ulna, as well as a very high incidence of lepromatous leprosy. At least 12 individuals exhibit clear osteological signs of the disease[^60^](https://paperpile.com/c/UPmHk7/n8nwj).

#### Bubi's cave (BBC)

Region: Karlovac

Coordinates: 45.34"N 15.47"E

Occupation period: 250 CE - 300 CE

Samples: 35

Sample IDs: BBC001, BBC002, BBC003, BBC004, BBC005, BBC006, BBC007, BBC008, BBC009, BBC010, BBC011, BBC012, BBC013, BBC014, BBC015, BBC016, BBC017, BBC018, BBC019, BBC020, BBC021, BBC022, BBC023, BBC024, BBC026, BBC027, BBC029, BBC030, BBC031, BBC032, BBC033, BBC034, BBC035, BBC037, BBC038

Sex Ratio: 11F, 24 M

Provenance note: Samples from Bubi's cave were provided by the Croatian Academy of Sciences and Arts. Permission to analyse was granted to coauthor Johannes Krause in 2022.

Contact person: Mario Šlaus

Bubijeva jama or Bubi’s cave is located in the Srnjak forest near the village of Siča, approximately nine kilometers southwest of Karlovac in Karlovac County, Croatia. The site was accidentally discovered in 1999 when a boy named Tihomir Boljar, nicknamed Bubi, almost fell into the narrow entrance of the pit cave while picking mushrooms. Subsequent speleological and archaeological research revealed that the cave had a depth of 21 meters and consisted of three parts. In the central hall lay the scattered skeletal remains of 30 individuals along with their grave goods, all of them covered with a thin layer of travertine. Although subterranean waters had scattered the skeletal remains and associated grave goods making them not in situ, there is no doubt that the site is a single, closed archaeological unit that has remained preserved since ancient times. It is believed that no one had entered the cave since the time of the ancient burial with the travertine layer and constant temperature and humidity preserving the remains. The excavated and archaeologically documented area is slightly under 192 square meters. The terminus post quem for the cemetery's use was determined as AD 259, based on recovered coins - the latest being an Antoninianus coin minted in Asia sometime between AD 258 and AD 259 depicting Cornelia Salonina, the wife of Emperor Gallienus. Notable metal finds include a heavily profiled bronze fibula, a silver-wire decorative pin, an iron axe, an awl, six iron keys, seven iron wedges, two clamp couplings, and a nail. Ceramic finds comprise the remains of at least 28 different vessels: nine pots, four jugs, two bowls, two plates, a cup, and a lid, while other ceramic fragments were too fragmented to define. These vessels were mainly intended as grave goods placed beside the deceased. Additional finds include a glass paste bead, a flint item possibly used for starting fires, and a pendant made from a piece of deer antler. Anthropological analysis revealed the presence of a minimum of 35 individuals: nine subadults, ten women, and sixteen men. There were no signs of violent death or infectious diseases. The presence of small bones of the wrist and feet suggests that this was not a secondary burial. Additionally, numerous animal bones, primarily domestic animals including pigs, sheep, and goats, were found. Most of these animal bones exhibit signs of being cooked and prepared for consumption, with cut marks indicating meat processing. One possible reason for this very unusual burial in a 21 meters deep cave may be the so-called plague of Cyprian that affected the Roman Empire from around AD 250 to 270[^61,62^](https://paperpile.com/c/UPmHk7/ceoYr+owngr).

#### Dubrovnik cathedral (DUC)

Region: Dalmatia

Coordinates: 42.64"N 18.11"E

Occupation period: 500 CE - 1100 CE

Samples: 1

Sample IDs: DUC001

Sex Ratio: 1M

Provenance note: Samples from Dubrovnik cathedral were provided by the Croatian Academy of Sciences and Arts. Permission to analyse was granted to coauthor Johannes Krause in 2022.

Contact person: Mario Šlaus

Following the strong earthquake that hit the general Dubrovnik area in 1979 archaeological excavations beneath the Dubrovnik cathedral, in the micro-toponym known as Bunićeva Poljana, were scheduled. Despite the fact that the baroque cathedral constructed between 1672 and 1713, suffered significant damage in the earthquake, all efforts were made to archaeologically investigate the site. Excavations began in 1981 and revealed that the cathedral had undergone numerous rebuilds and modifications throughout its history. Originally constructed between 1131 and 1157 it was gradually expanded upon and enveloped by a new Romanesque three-nave basilica. The stratigraphy of the site necessitated an expansion of the research area, ultimately resulting in the exploration of the entirety of Bunićeva Poljana area. By the conclusion of the excavation in 1987, a complex architectural network spanning 1200 m^2^ within the historical core of the city had been uncovered. This discovery significantly altered the understanding of Dubrovnik's early history, revealing a complex arrangement of buildings and structures beneath Bunićeva Poljana. These included a pre-Romanesque cathedral, later adapted and functionally linked to the Romanesque capital; a four-leafed building; a passage/corridor; multiple burials and tombs spanning seven layers; a defensive wall; remnants of a bell tower-baptistery from the Romanesque cathedral; and residential buildings dating back centuries. Based on the relationships between these structures, the observed partitions, upgrades, and stylistic characteristics, researchers tentatively date the complex to three distinct horizons: a) a late Antiquity horizon, b) an early medieval horizon and c) a mature Middle Ages (Romanesque) horizon. In anthropological terms, the recovered osteological material derives from 50 individuals whose graves had been excavated with, however, no archaeological information available for these finds. Because of this, dating the recovered skeletons is difficult and the assumption is that they are all medieval graves dated between the 6^th^ – 15^th^ centuries. The assemblage is characterized by a high frequency and antemortem traumas, the presence of one perimortem trauma in an adult male, and clear osteological evidence for the presence of lepromatous leprosy in two adult individuals[^63,64^](https://paperpile.com/c/UPmHk7/F8VX3+Iu98j).

#### Dugopolje - Vučipolje (DUG)

Region: Split-Dalmatia

Coordinates: 43.57"N 16.59"E

Occupation period: 1200 CE - 1600 CE

Samples: 1

Sample IDs: DUG001

Sex Ratio: 1M

Provenance note: Samples from Dugopolje - Vučipolje were provided by the Croatian Academy of Sciences and Arts. Permission to analyse was granted to coauthor Johannes Krause in 2022.

Contact person: Mario Šlaus

The first historical record for the settlement of Dugopolje is dated to 1283 in a document that defined the village boundaries. Its history throughout the late medieval period is strongly connected with the nearby fortress of Klis, one of the most important defensive fortifications in southern Croatia. The late medieval period in Croatia is characterized by the end of the rule of Hungarian-Croatian kings from the Arpad dynasty that was followed by feudal anarchy and constant skirmishes with Venetians for control of Dalmatia and the eastern Adriatic sea-ways, as well as frequent military incursions from the Ottomans in the 15^th^ and 16^th^ century. In the fall and winter of 2004/2005, archaeological research was conducted near Dugopolje, approximately twenty kilometers north-east of Split, along the route of the planned Split-Dubrovnik highway. Researchers investigated a total of 170 late medieval graves that contained the remains of 362 individuals. Seventy graves were covered with so-called ‘stećak’ stone slabs, with twelve of these slabs being decorated. The most common grave finds recovered were coins dating from the 13^th^ to the 16^th^ centuries discovered in twenty graves. Other finds include bracelets, three-berry earrings, rings, pins, textiles, and glass beads. The graves were generally oriented in a west-east direction with some minor deviations, although six graves were oriented in a south-north direction. Based on recovered coins and jewelry, burials in the cemetery can be traced as far back as the late 13^th^ century. The cemetery was in constant use from this time to the time this area was incorporated into the Ottoman empire in the 16^th^ century. No evidence of burials beyond this period has been found. In anthropological terms, the recovered osteological material is characterized by a very high incidence of skeletal indicators of intentional violence including perimortem trauma, sharp force lesions, craniofacial injuries and ‘parry’ fractures to the ulna that most likely reflect the violent history of this part of Dalmatia during the late medieval and early historic period, as well as a high frequency of cribra orbitalia in subadults[^65–68^](https://paperpile.com/c/UPmHk7/zQsBt+Go5Ya+Raqq4+xxx6V).

#### Podvršje - Glavčine (POD)

Region: Zadar

Coordinates: 44.25"N 15.32"E

Occupation period: 400 CE - 850 CE

Samples: 2

Sample IDs: POD001, POD002

Sex Ratio: 2M

Provenance note: Samples from Podvršje - Glavčine were provided by the Croatian Academy of Sciences and Arts. Permission to analyse was granted to coauthor Johannes Krause in 2022.

Contact person: Mario Šlaus

The site of Podvršje - Glavčine is located approximately 16 km north-east of Zadar, along the road that leads from Zadar to the island Pag. The site was excavated during several archaeological campaigns from 2002 to 2007, and subsequently in 2012 and 2015. An early Christian complex consisting of double churches (basilicas) with ancillary rooms and a cemetery was discovered. In addition to architectural remains, numerous fragments of stone furniture, architectural decorations, and ceramic and glass vessels dating to the late Antique period were recovered. As indicated by archaeological evidence and radiocarbon analysis, the entire complex was destroyed by fire in the first half or middle of the 7^th^ century. Based on recovered pottery sherds from the excavation of the oratory, construction of the double basilicas began during the first half of the 5^th^ century. The southern church, pastophorium (diaconicon), and baptistery with its cross-shaped baptismal font on the southern side were likely built in the middle or latter half of the 5^th^ century and during this period the chapel on the eastern side of the baptistery was also constructed. The northern church displays early Byzantine stylistic features dating its construction to the time of Justinian, most likely sometime between the 530 and 560. The cemetery is located on the western side of the churches’ front. A total of 30 graves, arranged in more or less regular rows, were excavated. They represent typical examples of late Antique graves including stone sarcophagi, walled tombs, graves under tegulae, and two infant burials in amphorae. Only four graves yielded finds. These include a mediterranean type buckle made of silver, a pair of golden earrings, a cross-shaped silver fibula and a fish-shaped iron fibula. Among the predominantly late Antique finds, fragments of early medieval ceramic vessels with Slavic technological and typological characteristics are particularly noteworthy. Six such vessels were found, mainly in the front part of the northern church. These included both handmade and slow-wheel pottery, as well as both decorated and undecorated pieces. The decorated examples featured a dominant motif of wavy lines between parallel lines. All the vessels were represented by sherds of pots made of purified clay tempered with calcite grains, with color variations depending on the firing process. These pots date to the second half of the 7^th^ century and the first half of the 8^th^ century. Radiocarbon analysis was performed on osteological remains from four graves. Grave 22 was dated to between 430 and 605, grave 25 to between 430 and 580, and grave 26 to between 440 and 600. One grave (G29) stands out due to its construction method, using a drystone wall technique, and was subsequently dug into the floor of the northern church. Radiocarbon analysis of the osteological material from this grave dates it to the early medieval period, specifically between 692 and 887. In anthropological terms the osteological assemblage is characterized by a high incidence of cribra orbitalia and linear enamel hypoplasia in subadults[^69–71^](https://paperpile.com/c/UPmHk7/snYsM+T08Vr+1FLVA).

#### Torčec - Cirkvišće (TOR)

Region: Koprivnica-Križevci

Coordinates: 46.22"N 16.87"E

Occupation period: 1100 CE - 1800 CE

Samples: 6

Sample IDs: TOR001, TOR002, TOR003, TOR004, TOR005, TOR006

Sex Ratio: 2F, 4M

Provenance note: Samples from Torčec - Cirkvišće were provided by the Croatian Academy of Sciences and Arts. Permission to analyse was granted to coauthor Johannes Krause in 2022.

Contact person: Mario Šlaus

The archaeological site of Torčec - Cirkvišče is situated northwest of modern day Torčec (in Podravina, northern Croatia), on a slightly raised terrace of the Drava river, east of the road connecting Torčec to Đelekovac. While noted previously in archaeological surveys, the site gained particular attention in 1999 with the discovery of a ceramic vessel containing the skull of a dog that was unearthed during plowing. The dog's head had been deliberately placed within the vessel, presumably for ritual purposes, around the mid 13^th^ century. This discovery stands as a unique find in Croatia with the closest parallels found in Hungary. Initial archaeological investigations of the site took place in 2002, with additional excavations carried out in 2009, as well as from 2011 to 2015. The remains of a small parish church were discovered, along with 383 skeletal graves. The two oldest graves date back to the late 8^th^ and early 9^th^ centuries, while the other graves belong to the parish cemetery and span from the mid 12^th^ century to the early 18^th^ century. With some minor deviations the graves were oriented in a west-east direction with the deceased placed in a supine position although two of the recovered skeletons were placed face down. The recovered grave finds are few and primarily consist of simple belt buckles and rings. Of interest is one individual who was found to have a rare type of rhomboid buckle, and one that was recovered with a small hoard of 18 coins carried in a partially preserved leather pouch on his belt. In anthropological terms, the osteological assemblage exhibits clear osteological evidence for the presence of lepromatous leprosy (in one female), numerous perimortem injuries inflicted by sharp bladed weapons, and a high frequency of cribra orbitalia in subadults. Additionally, one individual from this series, a female aged 25-30 years at the time of death, was found to be suffering from malaria caused by Plasmodium falciparum. Ancient Plasmodium DNA was extracted from an unidentified piece of calcified tissue that resembled a hydatid cyst[^66,72–74^](https://paperpile.com/c/UPmHk7/5njcg+kE4bv+Go5Ya+ZAU5q).

#### Velim (VEM)

Region: Zadar

Coordinates: 43.88"N 15.72"E

Occupation period: 650 CE - 900 CE

Samples: 67

Sample IDs: VEM001, VEM002, VEM003, VEM004, VEM005, VEM006, VEM007, VEM008, VEM009, VEM010, VEM011, VEM012, VEM013, VEM014, VEM015, VEM016, VEM017, VEM018, VEM019, VEM020, VEM021, VEM022, VEM023, VEM024, VEM025, VEM026, VEM027, VEM028, VEM029, VEM030, VEM031, VEM032, VEM033, VEM034, VEM035, VEM036, VEM037, VEM038, VEM039, VEM040, VEM041, VEM042, VEM043, VEM044, VEM045, VEM046, VEM047, VEM048, VEM049, VEM050, VEM051, VEM052, VEM053, VEM054, VEM055, VEM056, VEM057, VEM058, VEM059, VEM060, VEM061, VEM062, VEM063, VEM064, VEM065, VEM066, VEM067

Sex Ratio: 35F, 32M

Provenance note: Samples from Velim were provided by the Croatian Academy of Sciences and Arts. Permission to analyse was granted to coauthor Johannes Krause in 2022.

Contact person: Mario Šlaus

Velim is an early medieval Old Croat bi-ritual cemetery located in the micro-toponym Velištak in the Dalmatian hinterland, approximately 25 km north-west of Šibenik in the Croatian county Zadar. The site was discovered in 2004 during rescue archaeological excavations conducted during the construction of a feeder road that is now part of the Zagreb to Split highway. It was excavated in 2004, and subsequently in 2005 and 2013. A surface area of approximately 1400 square meters was explored from which 150 skeletal graves and 27 cremation graves were excavated. In the vast majority of cases the skeletal graves are oriented in an east-west direction (with the head in the west and the feet in the east) although some graves have a north-south orientation. Likewise, in the vast majority of cases the body of the deceased individual was placed in a supine position, although sporadic cases of flexed position were also noted. Graves were built on a square plan made from amorphous stones arranged in irregular rows. Most contain a headboard and footboard, each made from a larger stone slab although in some cases these were absent. The bottom of the grave usually consists of compacted earth but some graves exhibit a floor covered with smooth flat stones. The graves were covered with large irregular stone slabs. Grave finds recovered from the cemetery include jewelry consisting of simple earrings and earrings with three spiral pendants, necklaces with glass beads, rings, and one half-moon shaped pendant. Recovered everyday items include iron needles, flints and one bucket. Clay pots are present in most graves with some graves containing two pots, one by the head, and the other by the feet. Some of the pots were covered with thin flat stone plates and contained animal bones (generally cow and pig) that most likely represent faunal remains that served to accompany the deceased as a provision for the afterlife. Most graves also contained iron knives of various lengths. These knives were recovered from both male, female, and subadult graves. Weapons were infrequent and were recovered only in male graves. They include iron arrow heads and long battle knives. Of interest is the fact that some graves contained objects from earlier historical periods. These include a neolithic flint knife, an iron age pin, a Roman era bronze key as well as Roman era glass. Based on the recovered archaeological artifacts and ^14^C dating of human osteological samples from 31 graves, the cemetery was in use from the 7^th^ to the 9^th^ century. In anthropological terms the assemblage exhibits clear osteological evidence of skeletal tuberculosis (in one male and one female), lepromatous leprosy (in one female), ankylosing spondylitis (in one male) and boasts a very high percentage of individuals with antemortem traumas. Thirty of the 52 males preserved sufficiently enough to give an accurate diagnosis exhibit antemortem traumas, 16 of which were the direct result of intentional interpersonal violence. Stable carbon and nitrogen isotope analysis shows that individuals buried in the cemetery had a diet significantly different from the Late antique populations that preceded them in this part of Croatia. While antique populations existed on a diet of wheat, barley, animals fed upon C3 plants and significant amounts of marine resources: fish, and fish products such as garum (fish sauce), the inhabitants of Velim existed on a diet that completely ignored marine resources and was based on C4 plants, primarily millet, and animals fed upon C3 plants[^75–77^](https://paperpile.com/c/UPmHk7/Z0esR+xHxSU+QNBLx).

#### Vir - Smratina (VIR)

Region: Zadar

Coordinates: 44.31"N 15.06"E

Occupation period: 400 CE - 1200 CE

Samples: 1

Sample IDs: VIR001

Sex Ratio: 1M

Provenance note: Samples from Vir - Smratina were provided by the Croatian Academy of Sciences and Arts. Permission to analyse was granted to coauthor Johannes Krause in 2022.

Contact person: Mario Šlaus

Vir is an island on the Croatian coast of the Adriatic Sea with an area of approximately 22 km2. It lies in Dalmatia, north of the city of Zadar and is connected to the mainland via a road bridge. The main village on the island is the eponymous village of Vir. The micro-toponym Smratine is located 250 meters from the northern coast of the island and 2.7 kilometers from the center of Vir, in the broader area of today's Lozice settlement. Between 2013 and 2018 seven archaeological campaigns were conducted at the Smratine site that have so far yielded 17 graves. Seven of these date to the Middle Ages and are linked to a newer church, while ten belong to early Christian times. Two sacral structures were investigated at the site. The older church, built in the 6^th^ century, is a single-nave structure with a longitudinal floor plan, featuring a polygonal apse on the outside and a semicircular apse on the inside. A smaller church, likely from the Romanesque period, was later constructed within the nave of the older church, and is characterized by a longitudinal shape, a semicircular apse, and two pairs of interior belts. Along the south wall of the older church, a children's tomb (tomb 13) was discovered, containing the remains of seven children and two early Christian fibulae—one bird-shaped and one plate fibula. The construction of the younger church, probably from the Romanesque period (12^th^-14^th^ centuries), incorporated 21 fragments of early Christian spolia, indicating it was built using materials from the older church. The analyzed osteological material dates from the 12^th^ century and is characterized by a high frequency of antemortem and perimortem injuries that typically result from intentional interpersonal violence[^78^](https://paperpile.com/c/UPmHk7/THFr9).

### Eastern Germany

#### Brücken (BRC)

Region: Saxony-Anhalt

Coordinates: 51.44"N 11.19"E

Occupation period: 400 CE - 600 CE

Samples: 57

Sample IDs: BRC001, BRC002, BRC003, BRC004, BRC005, BRC006, BRC007, BRC008, BRC009, BRC010, BRC011, BRC012, BRC013, BRC014, BRC015, BRC016, BRC017, BRC018, BRC019, BRC020, BRC021, BRC022, BRC023, BRC024, BRC025, BRC027, BRC028, BRC029, BRC030, BRC031, BRC032, BRC033, BRC034, BRC035, BRC036, BRC037, BRC038, BRC039, BRC043, BRC045, BRC046, BRC047, BRC048, BRC049, BRC050, BRC051, BRC052, BRC053, BRC054, BRC055, BRC057, BRC058, BRC059, BRC060, BRC061, BRC063, BRC064

Sex Ratio: 33F, 24M

Provenance note: Samples from Brücken were provided by the State Office for Heritage Management and Archaeology Saxony-Anhalt and State Museum of Prehistory. Permission to analyse was granted to coauthor Johannes Krause in 2022.

Contact person: Arnold Muhl, Jörg Orschiedt, Ralf Schwarz, Harald Meller

The early medieval burial ground at Brücken, located in the Mansfelder Land district, was thoroughly investigated and completely recorded in 2020 in preparation for a construction project. The site is situated east of district road 2298, approximately 1.3 kilometers south of the village of Brücken, covering an excavation area of 6,525 m². Comprehensive documentation of the excavation is available.

A total of 76 graves containing 79 individuals of various ages and sexes were uncovered. Additionally, a sacrificial pit was found containing 5 horses, 4 cattle, and 2 dogs, all of which were killed relatively young, aged 2-5 years, and were predominantly male (analysis conducted by Carola Oelschlägel). The skeletal remains were subjected to anthropological, and radiometric analyses. However, about a third of the human bones analyzed for radiocarbon dating at the Curt-Engelhorn-Zentrum Archäometrie in Mannheim required a second sampling due to low collagen content. Samples were also taken from the human remains for genetic and isotope analyses. Consequently, 12 individuals could not be dated or sampled due to their poor preservation and lack of bone tissue.

Initial assessments of the ^14^C values and characteristic grave goods suggest a Germanic-Thuringian utilization phase of the site between approximately 460 and 540 CE. The grave inventories indicate a clear social hierarchy among the interred, ranging from valuable weapons and jewelry to inferior working tools and, in some cases, no grave goods at all. These findings, combined with the period of burial, suggest that the site served as a burial ground for the inhabitants of a manorial farmstead. The grave goods, including armor, household items, amulets, and clothing accessories, feature local Thuringian material culture as well as objects from distant regions. For instance, Frankish tableware and Alamannic jewelry suggest contacts with the Rhineland and southwestern Germany.

The Brücken cemetery's size is typical for burial grounds in the early medieval Thuringian kingdom (c. 455-531). A comparison with other published Thuringian cemeteries—though many have not been fully documented—shows that more than one hundred burials are rarely found[^79^](https://paperpile.com/c/UPmHk7/IJqlx). About 60 cemeteries and burial sites from this period are known in Saxony-Anhalt, but only the Brücken necropolis has been fully identified and extensively documented. This dataset provides a unique opportunity for demographic calculations based on reliable figures for a localized settlement. Moreover, the results of the strontium isotope and genetic analyses can verify the extent to which traditional ethnographic interpretations (typology, stylistics, find mapping) correlate with the origin and ancestry of the individuals.

The associated farmstead has not yet been discovered. However, based on settlement studies[^80,81^](https://paperpile.com/c/UPmHk7/E5j7C+02WLQ) and the terrain, it was likely located within a radius of approximately 500-1,000 meters. The location was strategically chosen, lying between the Harz Mountains and the Kyffhäuser Mountains within the Golden Aue, an area known for its fertile soils, favorable climate, moderate terrain, and good water supply[^82^](https://paperpile.com/c/UPmHk7/AQ1AF). The site is on a ridge protected from flooding, within a corridor about three kilometers wide between the Pfüffeler Bach to the south and the small river Helme to the north. Records from Carolingian and Ottonian sources indicate that several long-distance roads converged in this area, meeting near the modern village of Brücken to cross the once marshy Helme and connect with a military road running northeast. This strategic location likely played a crucial role in the settlement’s establishment, providing access to rare goods from abroad.

The anthropological analysis of the skeletal remains is limited by their predominantly poor to moderate preservation, affecting age determination, sex assessment, and the identification of pathological conditions. Overall, 29% of the individuals were subadults, including the infans and juvenile age classes. Younger adults (20-40 years) constituted 35%, while older adults (40-60 years) made up 36%. Morphological sexing indicated that 44% of the adults were certainly or probably female, whereas only 22% were determined to be male or probably male.

However, genetic sex determination revealed discrepancies regarding the individuals identified as probably female. Some poorly preserved skeletal remains, initially classified as potentially female, were genetically identified as male. This discrepancy is partly due to the poor preservation of the pelvis and the general gracility of the long bones and skulls. Consequently, the gender ratio shifts to a more balanced distribution: 32% female and 29% male.

Pathological analysis revealed a high frequency of dental diseases in 51% of individuals, including calculus, periodontal disease, caries, and apical processes. Additionally, degenerative diseases, particularly of the spine, were detected in 29% of older adults. Healed traumas were present in 5% of the postcranial skeleton and 2.5% of the skulls. A notable finding is an artificially deformed skull from feature 46, identified in a robbed grave of a 45- to 55-year-old man, whose sex was also genetically confirmed. This individual is currently the only man with an artificially deformed skull found in Eastern Germany.

#### Deersheim (DRH)

Region: Saxony-Anhalt

Coordinates: 51.98"N 10.78"E

Occupation period: 400 CE - 600 CE

Samples: 51

Sample IDs: DRH001, DRH006, DRH007, DRH008, DRH009, DRH010, DRH011, DRH012, DRH013, DRH014, DRH015, DRH016, DRH017, DRH018, DRH019, DRH020, DRH021, DRH022, DRH023, DRH024, DRH025, DRH026, DRH027, DRH028, DRH029, DRH030, DRH031, DRH032, DRH033, DRH034, DRH035, DRH036, DRH037, DRH040, DRH041, DRH042, DRH043, DRH044, DRH045, DRH046, DRH047, DRH048, DRH049, DRH050, DRH051, DRH052, DRH053, DRH054, DRH055, DRH056, DRH057

Sex Ratio: 33F, 24M

Provenance note: Samples from Deersheim were provided by the State Office for Heritage Management and Archaeology Saxony-Anhalt and State Museum of Prehistory. Permission to analyse was granted to coauthor Johannes Krause in 2022.

Contact person: Arnold Muhl, Jörg Orschiedt, Ralf Schwarz, Harald Meller

Excavations at Deersheim (Harz district, Saxony-Anhalt, site 16) on the Grandberg were carried out by employees of the Halle Museum from 1964-1969. The findings and discoveries were published in 1983 by J. Schneider. The excavations led to the discovery of 27 body graves – two of which contained two dead people –, 17 urn graves and eight horse graves, with one person each buried in a single horse grave (grave 49) and a double horse grave (grave 48). Dogs were also found in three horse graves. Almost all of the body graves were oriented west-east, including those of the horses. Only grave 30, located on the eastern edge of the cemetery, was facing north. Two large wooden chamber graves (graves 10, 14) are notable among the grave types.

The period of occupancy extended from around 450-565. Based on the burial rites, grave goods and burial areas, three consecutive periods of occupancy can be distinguished. The oldest are urn graves, followed by body graves from 500 onwards, the furnishings of which show a direct political and social connection with the Thuringian kingdom. The destruction of the kingdom by the Franks in 531 had a direct effect on the composition of the grave goods, as the level of furnishings dropped and influences from the groups settled in the vicinity, generally referred to as Saxons, came to the fore. These can be proven as newcomers by north-south oriented graves (graves 30, 45) on the eastern edge of the cemetery. Therefore, the occupation of the burial ground did not end around 531 like the cemeteries in the core area (see Rathewitz and Obermöllern), but survived the caesura, as the population settled in the northern Harz was far away from the theaters of war. The social position of the leading family(s) apparently did not change, whose women continued to wear gold-plated silver brooches and imported precious millefiori pearls from Italy.

The body burials grouped around the urn graves of the oldest occupation section include the graves of two adults (graves 9, 55), one (grave 9) can be identified as a man's grave, and two children (graves 17, 18) and probably belong to a family group. The group to the east, consisting of two men's graves (graves 10, 15), two women's graves (graves 14, 29) and five children's graves (graves 24-26, 28, 30), served as a burial site for two families. While the adults were buried around the children in the second phase of occupation, in phase 3 they lay one above the other in grave shafts (graves 42, 44).

As the majority of the adult graves had been robbed when they were discovered, the wealth of the group of people affected by looting can only be guessed at from the remains left in the grave. However, these are enough to identify them as members of a local noble family. Gold and gold-plated silver jewellery and imported vessels are particularly expressive of social status: these include wheel-turned pattern-burnished ware from the core area of the Thuringian Empire, as well as silver spoons, cups and drinking bowls made of mottled glass or glass decorated with thread overlays, wooden buckets with bronze fittings and basins made of bronze sheet from the Frankish Empire. An Ostrogothic belt buckle and Pannonian wheel-turned jugs found their way to Deersheim via the Thuringian Empire. Riding horses that followed their owner into death and were buried on the edge of the cemetery were an expression of the noble way of life, as were the burial of dogs together with horses (graves 43, 47, 49). In addition to single horses, pairs were also buried. Twice (graves 48, 49) people were buried together with horses. Riding accessories have only been found in two graves, a man's grave from phase 2 (grave 9) and a woman's grave from phase 3 (grave 44/I). These include gold-coated bridle gags, iron bits and golden strap ends decorated with almandine, again testifying to the high social status of the people buried in the graves. Other objects indicating nobility, such as swords or magnificent belt accessories, fell victim to the looting. The fact that they were deliberately taken shows that the robbery of the body graves must have taken place shortly after the burial. The graves therefore not only reflected the status of the deceased person externally, the deceased or their social position was probably known, and the grave robbers may even have been present at the burial ceremonies. Remarkably, offerings in the form of cattle skulls under stones and various animal sacrifices were found in two of the robbery pits.

This wealth was, however, directly linked to the establishment of the Thuringian Kingdom, into whose sphere the local population was included. The same applies to the acceptance of corpse burial, while the Saxons normally cremated their dead. This form of burial still characterized Phase 1 in the Deersheim cemetery, and differs drastically from the burial customs of the Thuringians in the core area at that time (see the grave fields of Obermöllern and Rathewitz). Only one urn grave stands out for the addition of an imported bronze basin, and even indicates social differentiation for the 5th century. The character of the population buried in the Deersheim grave field is testified to by the ceramic repertoire, which is rooted in local traditions and was handed down until around 565[^16,83^](https://paperpile.com/c/UPmHk7/LUxWO+3XRJl).

#### Niederwünsch (NDW)

Region: Saxony-Anhalt

Coordinates: 51.35"N 11.8"E

Occupation period: 1000 CE - 1200 CE

Samples: 201

Sample IDs: NDW001, NDW002, NDW003, NDW004, NDW005, NDW006, NDW007, NDW008, NDW009, NDW010, NDW011, NDW012, NDW013, NDW014, NDW015, NDW016, NDW017, NDW018, NDW019, NDW020, NDW021, NDW022, NDW023, NDW024, NDW025, NDW026, NDW027, NDW028, NDW029, NDW030, NDW031, NDW032, NDW033, NDW034, NDW035, NDW036, NDW037, NDW038, NDW039, NDW040, NDW041, NDW042, NDW043, NDW044, NDW045, NDW046, NDW047, NDW048, NDW049, NDW050, NDW051, NDW052, NDW053, NDW054, NDW055, NDW056, NDW057, NDW058, NDW059, NDW060, NDW061, NDW062, NDW063, NDW064, NDW065, NDW066, NDW067, NDW068, NDW069, NDW070, NDW071, NDW072, NDW073, NDW074, NDW075, NDW076, NDW077, NDW078, NDW079, NDW080, NDW081, NDW082, NDW083, NDW084, NDW085, NDW086, NDW086, NDW087, NDW088, NDW089, NDW090, NDW091, NDW092, NDW093, NDW094, NDW095, NDW096, NDW097, NDW098, NDW099, NDW100, NDW101, NDW102, NDW103, NDW104, NDW105, NDW106, NDW107, NDW108, NDW109, NDW110, NDW111, NDW112, NDW113, NDW114, NDW115, NDW116, NDW117, NDW118, NDW119, NDW120, NDW121, NDW122, NDW123, NDW124, NDW125, NDW126, NDW127, NDW128, NDW129, NDW130, NDW131, NDW132, NDW133, NDW134, NDW135, NDW136, NDW137, NDW138, NDW139, NDW140, NDW141, NDW142, NDW143, NDW144, NDW145, NDW146, NDW147, NDW148, NDW149, NDW150, NDW151, NDW152, NDW153, NDW154, NDW155, NDW156, NDW157, NDW158, NDW159, NDW160, NDW161, NDW162, NDW163, NDW164, NDW165, NDW166, NDW167, NDW168, NDW169, NDW170, NDW171, NDW172, NDW173, NDW174, NDW175, NDW176, NDW177, NDW178, NDW179, NDW180, NDW181, NDW182, NDW183, NDW184, NDW185, NDW186, NDW187, NDW188, NDW189, NDW190, NDW191, NDW192, NDW193, NDW194, NDW195, NDW196, NDW197, NDW198, NDW199, NDW200

Sex Ratio: 116F, 85M

Provenance note: Samples from Niederwünsch were provided by the State Office for Heritage Management and Archaeology Saxony-Anhalt and State Museum of Prehistory. Permission to analyse was granted to coauthor Johannes Krause in 2022.

Contact person: Felix Biermann, Harald Meller

Large cemetery sections or almost completely investigated early and high medieval Slavic cemeteries are still rare in the Elbe-Saale region. All the more significant was the discovery and investigation of the cemetery at Niederwünsch (Saale district) in 2009/2010 as part of the archaeological investigations by the Saxony-Anhalt State Office for Archaeology and Monument Preservation on the Erfurt/Halle-Leipzig ICE line. The site is located around 500 m from Niederwünsch on the edge of the Klobikauer Grund. Niederwünsch is mentioned with a Slavic name in the Hersfeld tithe register as early as the 9th century. During the excavations, 188 graves were found, which adjoined a presumed wooden sacral building in the east (Fig. 1). The boundaries of the burial ground could be reached to the north, south and east. Only the western boundary lay outside the area under investigation. In view of the topography, only a few graves can be surmised to the west of the excavation boundary, as the terrain here merges into a pronounced depression. The number of graves within the excavation area is likely to have been significantly higher. An unknown number of graves have been destroyed as a result of agricultural development of the area and medieval and modern soil interventions.

Structurally, the necropolis is a distinct row cemetery. In some places, especially in the centre and on the eastern edge of the cemetery, there are clear concentrations of graves, which are so massive in places that it is difficult to trace the original order of the graves. As with the partial interruptions in the rows of graves, these appear to be group formations that probably indicate the social relationships of the deceased. Close social ties are particularly evident in dislocation burials, true double burials and additive graves as well as superposition burials in the same grave pit. All burial pits, as well as the burials themselves, were orientated west-east, although there were often insignificant deviations from the ideal west-east axis. Genuine grave goods were rare in the cemetery and were generally limited to symbolic objects (e.g. eggs). Female costume jewellery was far more common. In addition to beads and finger rings, these were mainly the typical Slavic temple rings of the 11^th^/12^th^ century (Fig. 2). These and similar finds also define the main period of the burial ground. There were no regular elite graves or graves that stood out from the crowd. Rather, it can be observed that it is primarily the female individuals of the infans I/II and Juvenus age classes who were the most richly endowed. Further social components can be identified with regard to grave construction. The majority of the graves had a simple grave shaft with vertical walls and a flat floor. Occasionally, these grave shafts were also narrowly stepped in the upper area, apparently in connection with some evidence of a wooden covering. A clear western and Christian background is shown by 11 head niche graves, which were mainly found in the eastern half of the cemetery near the sacred building (Fig. 3).

A further and particularly remarkable form of grave construction is represented by niche graves of the so-called “Podmola type”, which are characterised by a burial niche built into the longitudinal wall of the burial pit below ground (Fig. 4). While the head niche graves show that a well-known type of grave with Christian connotations is attested here within a late Slavic, Christianised burial community of an assimilated population, niche graves of the Podmola type appear to be of a completely different origin. Podmola-type niche graves were found in 13 cases. This type of grave construction, hitherto unknown in the north-west Slavic region, occurs in the 6^th^ to 9^th^ century, especially north and east of the Danube, in the Avar region, the Slavic area of Slovakia and in Moravia. In the 10^th^ and 11^th^ centuries, only a few sites with niche graves were found in these areas. There are isolated graves from the 12^th^/13^th^ century and even from the 16^th^/17^th^ century. Such niche graves have also been found in the Oechlitz cemetery, only a few kilometres from Niederwünsch, and possibly in the area of the deserted settlement of Potenitz (Saalekreis). Since the graves of the 9^th^/10^th^ century AD from Oechlitz date significantly earlier than those from Niederwünsch, it can be assumed that the special, “foreign” grave form was passed on in this small region due to the short distance between the two cemeteries. It would appear that the local population adopted customs that had been established at least locally by immigrants in the Saale region at an unspecified time.

Despite the lack of a demonstrable, direct link to a settlement, the Niederwünsch cemetery shows all the characteristics of an early form of Christian church cemetery, which is also characterised by the head niche graves. This is supported by the dense occupancy in rows, the exclusive or predominant west-east orientation of the burials, the proximity to a sacred building and the almost complete absence or only very rare occurrence of genuine grave goods. The costume jewellery in the form of temple rings, finger rings and beads that frequently appears in Niederwünsch is, as elsewhere, in the typical Slavic tradition. This fact, like the occurrence of niche graves of the podmola type, also shows that independent elements of the local Slavic population of the Saale region were and could be continued long after the adoption of Christianity and the associated burial customs[^84–86^](https://paperpile.com/c/UPmHk7/663vy+Glnvn+NnyRb).

#### Obermöllern (OBM)

##### Thuringian Period (OBM_MP)

Region: Saxony-Anhalt

Coordinates: 51.16"N 11.67"E

Occupation period: 400 CE - 600 CE

Samples: 30

Sample IDs: OBM001, OBM002, OBM004, OBM006, OBM010, OBM014, OBM015, OBM017, OBM018, OBM019, OBM020, OBM022, OBM024, OBM003, OBM005, OBM007, OBM008, OBM009, OBM011, OBM012, OBM013, OBM016, OBM021, OBM023, OBM025, OBM050, OBM051, OBM052, OBM053, OBM054

Sex Ratio: 13F, 17M

Provenance note: Samples from Obermöllern were provided by the State Office for Heritage Management and Archaeology Saxony-Anhalt and State Museum of Prehistory. Permission to analyse was granted to coauthor Johannes Krause in 2022.

Contact person: Arnold Muhl, Jörg Orschiedt, Ralf Schwarz, Harald Meller

Excavations at Obermöllern (Burgenlandkreis, Saxony-Anhalt, site 1) were carried out by employees of the Halle Museum in 1925, 1927 and 1931. The findings and discoveries were published by F. Holter in 1925 and B. Schmidt in 1976. The excavations led to the discovery of 31 inhumation graves (1-6, 8-32). Since there is no find report for graves 21-27 and graves 25-27 were not properly inventoried, B. Schmidt reconstructed the grave inventories 25-27 based on grave names.

The period of occupation extended from 475–531. The end of the cemetery's occupation corresponds to the destruction of the Thuringian kingdom by the Franks in 531, which led to the destruction of the settlements, deportations and the flight of the population. This particularly applies to the noble estates, as can be seen from the end of occupancy in the associated cemeteries. The Obermöllern burial ground is one such noble cemetery. Evidence of this is provided by men's graves with swords (grave 16, 31) and in particular with full armament consisting of sword, lance and shield (graves 10, 15, 24, 26, 27 [without lance], 28) and women's graves with gold-plated silver brooches (graves 2 [1o+1u], 3 [2o], 5 [2u], 6 [1o+2u], 9 [2o+2u], 13 [1o+1u], 20 [2o+2u], 21 [1], 22 [2+1], 23 [2]; o: location on the chest or upper body, u: location in the pelvis or thigh area). Four swords have a damascened blade, which increases the value of the weapon and thus the social prestige of the owner (graves 15, 16, 26, 27). Since nine noble women – grave 21 belonged to a small child (infans II) – face eight men with swords, eight noble families could have been buried in the burial ground. The gold-plated silver brooch with almandine inlays found in child grave 21 and the gold-plated silver buckle with almandine inlays found in child grave 24 attest to inherited status. Gold bracteates with mythological content were found as amulets in two women's graves, in grave 6 on the mouth and in grave 20 together with three decorated gold disc pendants on the neck. It is noteworthy that horse burials as an expression of the mounted warrior nobility are missing from the Obermöllern burial ground.

Another category restricted to noble courts is the wheel-turned pattern-burnished pottery, which was found in nine graves, seven of which came from noble graves. The social status of two others cannot be determined (graves 24, 29). However, it is likely to be higher than that of people who were buried with simple bowls and who could have belonged to the entourage of the noble court. The graves containing arrowheads (grave 8) and lances (grave 11) could be attributed to men-at-arms. The infant in grave 4, who died at the age of 12-15 months and was buried with a bowl and an iron spearhead, was also born into such a class. The presumed boy wore a necklace of 42 glass beads around his neck.

According to the brooches, the women in graves 2, 6, 20, 22 and 23 can be identified as Thuringians, while the women in graves 9 and 13 could be of foreign origin. A member of the last generation remaining in Obermöllern and buried in the burial ground (grave 20) fled to the Alemanni in Schretzheim (grave 219) in 531.

Two women have tower skulls (graves 2, 6), a manipulation of the skull in infancy that goes back to the Huns and was adopted by the Ostrogoths, their closest allies. The woman in grave 5, who was found with such a skull, died in senile age. Of the three intervals calculated based on the 14C age (1556±27 BP), the middle one from 476-500 AD (24.3%) would provide the most plausible time span for the woman's age of around 20 years determined by ^14^C and the dating of the brooches that were found with her. Since combs in the present form were only in use until 480, it is conceivable that the woman was born during the last years of Attila's life (†453). The shape of the head would then indicate that the woman was born in the middle Danube region. The custom of tower skulls was also practiced by the Ostrogoths after the end of Hun rule. The tower skull of the woman in Grave 6 is based on this tradition. Her costume corresponds exactly to that of a Thuringian woman from the period 500-515. It is difficult to judge at what age she received her jewelry, as she also reached a senile age (60-70 years). Calibration of the 14C age (1621±19 BP) leads to four intervals, of which the second youngest between 500-509 AD (8.3%) fits the brooch equipment best. If the woman received her brooches at the age of 20, then she would have been born around 480 at the earliest and died around 540. Otherwise, the second oldest interval of 464-475 AD (12.2%) would indicate a birth around 450 and thus still in the time of Attila, but then the woman would have received her jewelry only at the advanced age of 50, which is unlikely, and would have died 10-20 years later[^16,87^](https://paperpile.com/c/UPmHk7/lAuD1+3XRJl).

##### Slavic Period (OBM_SP)

Region: Saxony-Anhalt

Coordinates: 51.16"N 11.67"E

Occupation period: 900 CE -1100 CE

Samples: 24

Sample IDs: OBM026, OBM027, OBM032, OBM033, OBM034, OBM036, OBM037, OBM039, OBM041, OBM044, OBM046, OBM047, OBM049, OBM028, OBM029, OBM030, OBM031, OBM035, OBM038, OBM040, OBM042, OBM043, OBM045, OBM048

Sex Ratio: 13F, 11M

Provenance note: Samples from Obermöllern were provided by the State Office for Heritage Management and Archaeology Saxony-Anhalt and State Museum of Prehistory. Permission to analyse was granted to coauthor Johannes Krause in 2022.

Contact person: Arnold Muhl, Jörg Orschiedt, Ralf Schwarz, Harald Meller

Excavations at Obermöllern (Burgenlandkreis, Saxony-Anhalt, site 9, Hornbogens Acker) were carried out by employees of the Halle Museum in 1929. The findings and discoveries were published by H. Rempel in 1966. The excavations led to the discovery of 23 body graves (I-XX, XXIII, A, B). The evidence of skeletal remains (characters) at different depths in the graves (Roman numerals) indicates multiple burials, some of which come from family burials. In addition to the individual graves, Rempel lists the following grave differentiations and depths: IIIb under IIIa, IVa-e, VIIa (skull 1.0 m deep) and VIIb (skeletal remains with grave goods 1.15 m deep), VIIc (skeletal remains 1.35 m deep) under grave VIIb, IX and IXa, Xa and Xb (man and woman 80 cm deep), Xc (child 45 cm deep, Xd (child 50 cm deep), XIVa (child 60 cm deep), XIVb (skeletal remains 1.50 m deep), XVII (2 skulls), XVIII (1 skull; Rempel lists 3 skulls). Only in grave IIIa does Rempel indicate a west-northwest-east-southeast orientation.

The majority of the burials – apart from a bird's egg from Grave I and an animal bone from Grave XI – contained no grave goods (Graves I, IVa, IVb, IVd, IVe, V, VI, VIIa, VIIc, VIII, IXa, Xa, Xb,m Xc, Xd, XI, XII, XIII, XIVa, XVI, XVII, XVIII, XIX, XX, XXIII, A, B). Four graves were children's burials (Graves IVa, IVb, Xc, Xd). In two other graves the grave goods consisted only of a knife (Grave IVc) and a bronze ring (Grave XV). In Graves IIIa, VIIb and XIVb the iron hoops, attachments and handles of wooden buckets were found. While two of these graves had no other grave goods, the dead woman in grave IIIa can be assigned to the local upper class based on two silver temple rings. The dead women in graves II and IX also belong to this class. The latter is also the most richly furnished women's grave in the cemetery. In addition to four silver temple rings, the dead woman in grave IX wore two bronze neck rings, two bronze finger rings and a necklace made of 118 glass beads, which included five silver-colored hollow double beads, three blue or light gray hollow triple beads and four cylindrical mosaic beads. Among the female upper class graves, burials II and IIIa show the symbolic addition of chicken eggs. On the other hand, rider's grave A indicates a male upper class grave. In it, next to the dead man buried in a west-east direction, lay a horse with its head turned towards its master (Grimm 1961, 112). This indicates a mounted warrior. The horse was missing its hind legs and hip bones, parts of which were found on the head of the deceased buried in grave B.

The population buried in the Obermöllern cemetery is mostly Slavic, among whom a small number (graves II, IIIa, IX, A) represent the local upper class. The lady of grave IX and the rider (warrior) of grave A possibly represent the German-Thuringian feudal lords. Accordingly, the temple rings of the lady of grave IX differ from the commonly found Slavic examples in their inner width of over 2 cm and the bent hook end. The dead woman also wore neck rings, an unusual costume for Slavic women[^88–90^](https://paperpile.com/c/UPmHk7/Jsmpa+7T6kN+yHI8h).

#### Rathewitz (RTW)

Region: Saxony-Anhalt

Coordinates: 51.11"N 11.9"E

Occupation period: 400 CE - 600 CE

Samples: 16

Sample IDs: RTW002, RTW003, RTW004, RTW005, RTW006, RTW007, RTW008, RTW009, RTW010, RTW011, RTW012, RTW013, RTW014, RTW015, RTW016, RTW017

Sex Ratio: 10F, 6M

Provenance note: Samples from Rathewitz were provided by the State Office for Heritage Management and Archaeology Saxony-Anhalt and State Museum of Prehistory. Permission to analyse was granted to coauthor Johannes Krause in 2022.

Contact person: Arnold Muhl, Jörg Orschiedt, Ralf Schwarz, Harald Meller

Excavations at Rathewitz (Burgenlandkreis, Saxony-Anhalt, site 1) were carried out by employees of the Halle Museum between 1955 and 1957. The findings and discoveries were published by B. Schmidt in 1976. The excavations led to the discovery of 18 body graves and a horse burial with a dog. However, only the graves that were cut into during construction work were examined.

The period of use extended from approx. 450-531. The end of the cemetery's use might correspond to the destruction of the Thuringian kingdom by the Franks in 531, which led to the destruction of the settlements, deportations and the flight of the population. This particularly might have affected the noble estates, as can be seen from the end of occupancy in the associated cemeteries. The Rathewitz burial ground is one such noble cemetery. Evidence of this is provided by men's graves with swords and, in particular, with full weapons consisting of sword, lance and shield. The youngest grave (Grave 14) is dated to the time of these events, which had significant consequences for the Thuringian Empire, by a coin of the Ostrogoth king Athalaric (526-534). The noble warrior therefore still likely belonged to the era of the protective and defensive alliance against the Franks forged by the Ostrogoth king Theodoric the Great (493-526), whose stability and effectiveness faded after his death and was difficult for his successor Athalaric to maintain. The family ties to the Amal dynasty, which existed through the marriage of the Thuringian king Hermenefried (ca. 510-534) to Amalaberga, Theodoric's niece, were the basis for the enduring loyalty to the Ostrogoths even after Theodoric's death. The second warrior grave with full armament (grave 19) is also associated with the late phase of the Thuringian Empire (515-531), as shown by the shield thorn buckle. In contrast, the men's graves from the period between 450 and 500 found during the excavation do not contain any swords, which testifies to their lower social status. Whether men's graves with swords were also buried at that time can only be assumed due to the limited excavation area. At least two women's graves (graves 8, 16) have been preserved from this period, each of which bore a brooch made of gold-plated silver appropriate for a noble lady. The gold-plated bronze brooch of the Rositz type, a brooch shape that suggests that the woman buried in grave 15 was of Herulian descent, also conveys a corresponding status.

In addition to swords and gold-plated silver jewelry, luxury goods in the form of Thuringian wheel-turned pattern-burnished pottery are found in five graves, as another characteristic of a noble court. In one grave (Grave 8) there is also a vessel made of mottled glass imported from the Frankish Empire. A horse burial with a dog completes the spectrum of characteristics otherwise typical of noble cemeteries.

The warriors who were not of noble birth served as men-at-arms at the noble court. The arrowheads found in the graves identify them as archers, among whom the warrior in Grave 2 also had an axe as a weapon. The three-winged arrowhead in the chest of the man in Grave 1 is likely to have been the cause of death, given its location near the spine.

The variety of the brooches from graves from the period 450-500 indicates that the wearer had different origins. In addition to local women (graves 3, 8, 16), women of potentially foreign origin can also be identified, whose original homeland may be found among the Heruli (grave 15), Lombards (grave 10) and Angles (grave 12). The Heruli woman may have fled to the Thuringians as a result of the defeat inflicted on her tribe by the Lombards in 508 in the area of South Moravia/Lower Austria, as a result of which the Heruli king Radulph died[^16^](https://paperpile.com/c/UPmHk7/3XRJl).

#### Steuden (SDN)

Region: Saxony-Anhalt

Coordinates: 51.41"N 11.76"E

Occupation period: 900 CE - 1200 CE

Samples: 36

Sample IDs: SDN001, SDN002, SDN003, SDN004, SDN005, SDN006, SDN007, SDN008, SDN009, SDN010, SDN011, SDN012, SDN013, SDN014, SDN015, SDN016, SDN017, SDN018, SDN019, SDN020, SDN021, SDN022, SDN023, SDN025, SDN026, SDN027, SDN028, SDN029, SDN030, SDN031, SDN032, SDN033, SDN034, SDN035, SDN036, SDN037

Sex Ratio: 18F, 18M

Provenance note: Samples from Steuden were provided by the State Office for Heritage Management and Archaeology Saxony-Anhalt and State Museum of Prehistory. Permission to analyse was granted to coauthor Johannes Krause in 2022.

Contact person: Arnold Muhl, Jörg Orschiedt, Ralf Schwarz, Harald Meller

Excavations at Steuden (Saalekreis, Saxony-Anhalt, site 1) were carried out in 2008 by employees of the State Office for Monument Preservation and Archaeology of Saxony-Anhalt. The excavation results were first presented by U. Müller in 2014. The excavations were limited to the width of the planned road of 7 m and led to the discovery of 30 body graves, of which 29 were single graves and one was a triple burial (grave 22), which can be culturally assigned to the Slavs. While the western edge of the cemetery was recorded in the area of the excavation area, the occupation continued eastwards beyond the excavation boundaries. The dead were buried in a stretched-out supine position in a west-east direction with their heads to the west, with the bodies in many cases wrapped in cloths. Some graves had partial or complete stone settings in the form of grave borders or as covers.

The cemetery is divided into two parts, a northern part with 18 graves and a southern part with 12 graves. In the northern part of the cemetery, the graves were arranged in two north-northwest-south-southeast rows and in the southern part in two north-south rows. In the northern part of the cemetery, findings 11 and 19 showed overlapping graves (11/1 under 11/2, 19/1 under 19/2). In the southern part of the cemetery, stone settings were found in five graves (graves 6-10), in the northern part only in two (graves 19, 32). The triple burial (grave 22) contained the bodies of a man, a woman and a child and can be interpreted as a family burial.

In total, only 3 burials were equipped with jewelry.

Grave 6, which was covered with limestone slabs, is one of the most richly decorated graves. It contained the body of an 8-10 year old child who wore a bronze ring on the ring finger of her right hand and two silver rings on her temples. Six snail shells were found in the pelvic area, four of which had holes drilled through them. Based on their location, they could have been kept in a bag. The temple ring of the young woman buried in the neighbouring grave 8 was also made of silver. On the middle finger of her right hand she wore a ring made of pointed oval bronze sheet with capped ends. Small temple rings with one blunt and one widened S-shaped end and finger rings made of pointed oval bronze sheet are common among the Slavs in the Elbe-Saale region. A child who died at the age of 4-6 and was buried in the northern part of the cemetery (grave 27) also had jewelry as grave goods. On the right side of the skull, three octahedron-shaped wire structures made of silver wire and a glass bead were found, which are interpreted as earrings. Comparable earrings are made of closed silver sheet octahedrons. They date from the 10^th^ century and the first half of the 11^th^ century. The same dating can be applied to the temple rings made of thick wire and wide S-loops.

Based on the orientation of the graves, it is believed that a two-phase pattern can be seen in the occupation of the burial ground, with the graves with jewelry belonging to the younger burial layer. However, as the use of silver jewelry shows, social reasons play a role in the furnishing of the graves. Nevertheless, the younger phase would belong to the 10^th^ century and the first half of the 11^th^ century.

The use of silver wire for the temple rings allows us to recognize a social differentiation of the local burial community based on the females. Among the Slavs, silver jewelry is reserved for a local upper class. The general population wore temple rings made of bronze wire, if at all. With regard to the burials of children, it is evident that social status was inherited[^91^](https://paperpile.com/c/UPmHk7/g7M2r).

### Latvia

#### Laukskola burial site (LAU)

Region: Livonia

Coordinates: 56.84"N 24.38"E

Occupation period: 900 CE - 1200 CE

Samples: 2

Sample IDs: LAU001, LAU002

Sex Ratio: 2F

Provenance note: Samples from Laukskola burial site were provided by the Institute of Latvian History, University of Latvia. Permission to analyse was granted to coauthor Johannes Krause in 2022.

Contact person: Gunita Zariņa

The Laukskola archaeological site was located on approx. 10 m high first terrace of the Daugava river bank (now flooded by Riga HPP reservoir) in Salaspils parish, on the right bank of the Daugava river, about 25 km from Riga, opposite Daugmale hillfort. In addition to the Liv burial site, the Laukskola archaeological complex also included two Liv villages – one linked to the E part of the burial site and the other to the W part. V. Ģinters excavated 24 graves in this burial site in 1936 and 1937. The site was thoroughly explored by A. Zariņa in 1967–1975, excavating about 10 ha of which 5 ha were covered by the burial site; 586 burials were found.

Burials at this site began in the late 10^th^ century and ceased in the early 13^th^ century. Although Laukskola site belongs to the category of flat burial sites, at least 16 individual barrows appeared during the last phase of burials (the second half of the 12^th^ century). Three types of burials have been identified: predominantly inhumations (425), a smaller number of cremations (165), and 20 symbolic burials. The largest numbers of cremations (more than a half of the total) date from the late 10^th^ century and the first half of the 11^th^ century; later, the number of new cremations decreased and during the last phase they accounted for only one-fifth of all burials. All of the deceased were buried with the head-oriented NW: adults in 0.5–1.3 m deep pits, children in shallower graves. In several cases, the floor of a pit was covered with a layer of white sand, while in some burials a layer of ferns and herbs covered by furs lined the bottom of a coffin. Bodies were buried in coffins, both log coffins and board coffins; in some cases coffins had been painted white. Ritual significance may be attributed to burnt grain found in some graves, eggs as a symbol of rebirth, as well as offerings of animals, especially dogs. Often (50% of all burials) earthenware vessels, presumably containing food, were placed in a grave. Evidence of bonfires was found at many places in the burial site.

The deceased were laid out in the grave wearing festive dress and ornaments, the men were buried together with their weapons; in some instances tools have been found among the grave goods, presumably related to the occupation of the deceased, as well as supplementary items (an axe, awls, shears, spindle whorls, a basket holding household objects, amulets, etc.) placed by the head or at the feet of the deceased or in the backfill of a grave. Quality and number of grave goods show that individuals interred at this site belonged to a socially differentiated society. The burials of rich, presumably noble, warriors stand out with grave goods including, along with other luxurious items, swords, spearheads made of damascene steel with silvered sockets, as well as spurs, remnants of shields, other weapons.

The set of ornaments present in female burial inventory also reflects their social status and wealth. A breast chain ornament was found in 56% of female burials. This set of ornaments consists of a pair of bronze tortoise brooches or dress-pins with attached chain holders and rows of chains with chain spacers. This ornament could be worn together with various amulets – tusks of a bear, amber pendants, stylised animal figurines, keys, a needle-box, a knife with a sheath ornately decorated with bronze fittings. Spiral bracelets were worn, as well as hollow ornamented bracelets, neck-rings, necklaces of glass beads and cowrie shells, with coins and bracteates as pendants, penannular brooches, and spiral finger-rings.

Only 23% of the burials were without any grave goods whatsoever, which is seen as evidence for interment at this site of members of an arguably well-off community. Vends were buried at this site as well as Livs. In addition, four Scandinavian and seven Semigallian burials were found[^92^](https://paperpile.com/c/UPmHk7/aQytP).

#### Mežīte burial site (MEZ)

Region: Courland

Coordinates: 57.32"N 25.28"E

Occupation period: 900 CE - 1200 CE

Samples: 1

Sample IDs: MEZ002

Sex Ratio: 1F

Provenance note: Samples from Mežīte burial site were provided by the Institute of Latvian History, University of Latvia. Permission to analyse was granted to coauthor Johannes Krause in 2022.

Contact person: Gunita Zariņa

The flat burial ground at Mežīte is located in Lauciene (formerly Lībagi) parish, on a small hill about 70 m N of Mežīte hillfort. The site was discovered in 2008, and archaeological excavations were carried out between 2009 and 2011 under the supervision of E. Guščika and A. Vasks. A total area of 450 m^2^ was excavated.

Although the territory of the burial field has been farmed for a long time and a gravel mine was established there, the survey revealed the presence of six burials, four of which were intact. Burials had occurred mainly in the higher part of the hill. Bodies were buried uncremated, spaced well apart, in about 0.45–0.7 m deep graves. The pits were oriented to the S–N or SE–NW (with some exceptions), and most probably different orientations depended on sex of the buried. The only intact male burial (burial 1) was oriented with the head pointing S, whereas that of a female (burial 2) – with the head pointing N. Judging by intact burials and stray finds of human bones, both adults and children had been interred at this. Almost all bodies had been laid out in a stretched supine position with arms folded in various ways (across the chest, over the hips, or straight along the sides). One exception is the burial of a child (burial 3), whose legs were also bent at the knees. There was no evidence for the presence of a coffin in any of the burials, but stone structures were an important component of several burials. Stones have been found both in the backfill of the burial pit (burials 1, 3) and densely packed over the deceased (burials 2, 6). Grave goods were also found for all burials. The most typical items include bronze penannular brooches and spiral finger-rings, neck-rings of bronze wire with spirals and pendants of animal teeth, iron awls, knives, and broad-bladed axes. Two strings of beads made of glass mass and bronze, strung on bronze wires, were discovered in disturbed burial 5, and bronze dress-pins with a triangular head in female burial 2.

The large number of little bronze rings and ringlets found here point to the presence of decorated fabrics. Additionally, one burial pit with two iron stirrups at the top was discovered, but no human remains were found therein.

All of the burials and artifacts found can be dated to the period of late 11^th^ century – 12^th^ century, which is also consistent with the dates attributed to other sites studied at Mežīte complex. The burial site at Mežīte is currently one of the most extensively studied Late Iron Age flat burial fields, located in Northern Kurzeme, that contain inhumations, and is probably associated with the Vends[^93,94^](https://paperpile.com/c/UPmHk7/8QIYa+7hF2J).

#### Vampenieši burial sites (VAM & DOV)

Region: Livonia

Coordinates: 56.85"N 24.21"E & 56.83"N 24.24"E

Occupation period: 900 CE - 1200 CE

Samples: 2

Sample IDs: DOV001, VAM001

Sex Ratio: 2F

Provenance note: Samples from the Vampenieši burial sites were provided by the Institute of Latvian History, University of Latvia. Permission to analyse was granted to coauthor Johannes Krause in 2022.

Contact person: Gunita Zariņa

Both Vampenieši burial sites were located on the upper end of Dole Island – a big island in Daugava river - on the high bank of Sausā Daugava branch, a few hundred meters from each other. Presently the sand sediments of the upper half of the island have been consumed in building Riga HPP dams and the place is flooded by Riga HPP reservoir.

Vampenieši I is a flat 10^th^–13^th^ century burial field, attributed to Livs. Excavations of it were carried out by E. Šnore in 1966, 1967, 1969, and 1971–1974. 198 burials, ritual pits, as well as sites of hearths were excavated. Inhumations predominate (124). There were also 63 cremations, including 29 male and 34 female, accompanied by grave goods. In all, 2137 artifacts were recovered.

Inhumations were found at a depth of 0.4–1.2 m, cremated remains at a depth of up to 0.4 m. Bodies were buried in log coffins, and, from the mid-11^th^ century onwards, also in coffins made of boards nailed together. An additional compartment was found at the head of some log coffins, possibly containing food. Bodies were laid out in a stretched supine position, with the head oriented towards the NW and arms set in different positions. Cremated remains were placed in small-diameter nests or in square-shaped grave pits. There was an almost equal number of male and female burials.

The 11^th^ - 13^th^ centuries Vampenieši II burial site is approximately 400 m downriver; it contains a number of Liv barrows. Barrows had been damaged by grave robbers. Holes that were dug were later leveled by bulldozers, and during archaeological investigations only a few barrows were evident.

Archaeological excavations in the burial ground were carried out in 1966–1967 by E. Šnore. In total, 116 burials were excavated; they contained 897 artifacts. The dominant type of burial were inhumations. Also, 15 cremation burials were uncovered, of these most (11) had grave goods typical of male burials. In inhumations the dead were laid in coffins made of boards fastened together with nails, in a stretched supine position, with arms folded in various ways. The dead were buried in rectangular grave pits, 0.4–1.2 m deep. Adults were buried with their heads in the NW direction while children in the N direction.

Cremation took place outside the territory of the burial field. Cremation graves were dug at the depth of approx. 0.3 m, the calcined bones, and goods that were partially melted in the pyre were placed in small nests or, in some cases, in wooden coffins.

Grave goods for women included mainly jewellery, whereas for men – weapons, brooches, belts. Knives were found as grave goods for both men and women. There are also a significant number (73) of graves of non-adult individuals grouped together. Some graves (especially of boys) contain luxurious items.

The richest ornaments were found in graves from the second half of the 11^th^ century and the early 12^th^ century. An integral part of the grave goods for 11^th^ century burials is a long pectoral chain ornament with tortoise brooches, openwork chain holders, and chain dividers. Necklaces of glass beads and cowrie shells with pendants are also typical, as well as one to six neck-rings (most often with button and hook terminals and twisted rings with loop terminals). Several hollow bracelets or spiral bracelets were worn on each arm, as were spiral finger-rings and shield-shaped finger-rings. A bone comb in a sheath, an iron knife in a leather sheath with bronze fittings, a needle-box, an earthenware pot, and, sometimes, eggshells are typical grave goods. A broad-bladed axe was usually included in grave goods for men, as well as two socketed spearheads, and ornaments (bracelets, penannular brooches), leather belts decorated with bronze fittings[^95^](https://paperpile.com/c/UPmHk7/91W6r).

### Poland

#### Boczne Rockshelter at Ogrojec (PC1)

Region: Lesser Poland

Coordinates: 49.72"N 21.57"E

Occupation period: 200 CE - 400 CE

Samples: 1

Sample IDs: PC1001

Sex Ratio: 1M

Provenance note: Samples from Boczne Rockshelter at Ogrojec were provided by the Faculty of Archaeology, University of Warsaw. Permission to analyse was granted to coauthor Zuzana Hofmanová in 2022.

Contact person: Małgorzata Kot

The rockshelter (Polish: Schronisko Boczne w Ogrojcu; Prądnik Korzkiewski, Wielka Wieś commune, Cracow district) is a small niche situated ca 50–59 m above the valley, maximally 16 m long, but this decreases into a small chamber ca 3–4 × 1.5 m wide. The cavity has two openings—the lower is large, ca 4 × 5.8 m wide and easily accessible, whereas the upper is smaller and is reached via a small chimney open to the rock surface. It is light and dry. Importantly, the rockshelter is situated only a few meters north of Upper Cave at Ogrojec. Together with the latter site, Boczne Rockshelter at Ogrojec was excavated in 1901–1902 by Stanisław Jan Czarnowski, who unearthed dozens of pottery pieces, flint, bone and metal objects while exploring the uppermost humic level, ca 0.7–0.8 m thick. Notably, for the most part these finds correspond with the Neolithic and Middle Ages. Apart from a single younger or late Roman Age/Migration Period pottery find there is only one single artifact which may be conditionally dated to the period in question— an iron head of a pole arm of II.1 type as defined by Piotr Kaczanowski. Such forms frequently appear in the Przeworsk culture from the Early Roman Period to phase C1a, although similar objects incidentally appear later, during the Middle Ages. Among the numerous animal bones from Czarnowski’s research, which are stored in the State Archaeological Museum in Warsaw, human remains from two adult individuals were identified. One of the bones in question (a rib) produced a radiocarbon date 1610 ± 30 BP (1475 cal. BP, 1536-1408 95% CI) (Poz-129824)[^96–98^](https://paperpile.com/c/UPmHk7/iT4H5+jp77G+AYmZy).

#### Gródek (GRK_MP & GRK_SP)

Region: Hrubieszów

Coordinates: 50.79"N 23.94"E

Occupation period: 200 CE - 500 CE (GRK016-GRK024) & 600 CE - 900 CE (GRK004, GRK008, GRK010, GRK011, GRK013-GRK015) & 1000 CE - 1200 CE (GRK001, GRK002, GRK005 - GRK007, GRK009, GRK012) & 1400 CE - 1700 CE (GRK003)

Samples: 24

Sample IDs:

MP: GRK016, GRK017, GRK018, GRK019, GRK020, GRK021, GRK022, GRK023, GRK024

SP: GRK001, GRK002, GRK003, GRK004, GRK005, GRK006, GRK007, GRK008, GRK009, GRK010, GRK011, GRK012, GRK013, GRK014, GRK015,

Sex Ratio: 13F, 11M

Provenance note: Samples from Gródek were provided by the Rev. Stanisław Staszic Museum, Hrubieszów. Permission to analyse was granted to coauthor Zuzana Hofmanová in 2022.

Contact person: Marcin Wołoszyn

The complex of sites on a loess hillock in Gródek (the site is known also as Gródek nad Bugiem / Gródek upon the Bug River; Hrubieszów County, Lublin Voivodeship, Poland) extends along the left bank of the Bug River at the Polish-Ukrainian border – from the mouth of the Huczwa River to the Bug in the north to the vast and marshy depression called Królewski Kąt in the south. It covers an area of about 30 hectares in total and comprises five main archaeological sites. Unfortunately, the eastern side of the Bug (in Ukrainian territory) is far less explored.

The northernmost site is site 1A, bounded by the Huczwa River to the north and an artificial gorge to the south. The most visible element is the hillfort – site 1A, dated to the 9^th^ or 10^th^/11^th^ to the 13^th^ century (no modern excavations have been conducted on the rampart). Most likely, this site should be identified with the stronghold of Volyn’ recorded – for the first time in the description of events from 1018 – in the oldest chronicle of Rus’ (the so-called Primary Chronicle). An Early Medieval inhumation cemetery was identified inside the stronghold (site 1A), providing 466 graves (and 469 burials – two graves contained double burials; excavations in 1952–1955; unfortunately, many of the skeletons – which are stored in Wrocław – were lost during the 1997 floods [nowadays, in the storing facility of the Polish Academy of Sciences, Wrocław there are remains of 197 individuals]). Radiocarbon dates from this cemetery are in the 11^th^ century, with continuity in the 12^th^ and 13^th^ centuries. Some of these were elite burials (13 graves contained the remains of silk textiles). Traces of a much older settlement were also discovered at site 1A, including the remains of a Roman Period cemetery (destroyed by the Early Medieval cemetery mentioned above).

To the south of site 1A is located site 29 with the remains of a Roman Period settlement, traces of a late 5^th^ century Germanic (or in any case pre-Slavic) settlement, and grave finds from Tribal and Early Medieval Periods. Further south is the Lusatian culture cemetery (site 1B), separated by a gorge from the multicultural site 1C. The earliest traces of human occupation at this site are dated to the Late Palaeolithic Period (Bromme-Lyngby and Swiderian cultures). The area was intensively settled throughout the Neolithic Period (the Linear Pottery culture settlement, the Lublin-Volhynian culture cemetery, the Funnel Beaker culture settlement, the Globular Amphorae culture settlement trace), Bronze Age and Early Iron Age (the Strzyżów culture cemetery, the Mierzanowice culture settlement trace, the Trzciniec culture treasure, the Vysotskaya/Wysocko culture cemetery), and the Roman Period (the Wielbark culture/Masłomęcz group cemetery). In 1983, a warrior burial was also discovered there, furnished with a sword, the antler mounts of a reflex bow and elements of a bow case (12^th^–13^th^ centuries). The southernmost site is the multicultural site 6, with the most intensive settlement being linked to the Roman Period (the Masłomęcz group settlement).

Two cemeteries (sites 1A [the cemetery within the stronghold, destroyed by medieval graves] and 1C) and two accompanying settlements (sites 6 and 29) have been dated to the Roman Period. The Roman Period skeletons are traditionally linked to the Goths (the so-called Masłomęcz group). This cemetery was previously investigated by various archaeologists (the most important being Andrzej Kokowski) in 1957, 1961, 1984–1987, 1990–1993, 2001, 2006, 2017, 2019–2021. It is a biritual cemetery, with a definite predominance of inhumation graves and a whole spectrum of corpse treatment and post-funeral procedures, including the frequent opening of graves by the inhabitants of this site during the Roman period (i.e., if we believe the traditional ethnic interpretation, Goths). Six basic groups of inhumation burials can be distinguished: anatomically complete, with undisturbed bone structure; in graves with a so-called “robbery” excavation; partial graves in a grave pit without a clear trace of excavation; so-called partial graves (secondary partial) in a grave pit with traces of opening, probably without robbery intentions; so-called partial with an entrenched/added child grave, usually an infant; and biritual (inhumation-cremation in one grave pit). Establishing the number of graves recorded to date is extremely difficult. The main difficulty is the destruction of the features as a result of deep ploughing and soil erosion. At present, the number can be estimated at 206 at least, with less than half of the cemetery having been investigated. It should be stressed that this is an estimate of the number of graves, not burials. In some of the grave pits, more than one burial was discovered, with “extra” burials, usually child burials, being dug into/added to existing graves, or the remains – usually incomplete – of more than one individual deposited in a single grave pit (either simultaneously or during secondary grave opening). The cemetery dates from the late 2^nd^/early 3^rd^ century BC (phase B2/C1–C1a) to the early 5th century (phase D1). The skeletal remains subjected to genetic studies come from eight inhumation burials discovered (2019–2021) in six graves in the southern, youngest part of the cemetery, dated to phases C3 and D1 (300/310–400/410). Five of these belong to children, including neonates, with two neonatal burials buried in adult graves, as well as a single adult skull.

The 14C dates made in 2025 confirm the chronology based on the analysis of the archaeological finds themselves. Only one grave (G1C_OB.38) is older and can be linked to the earliest phase of the cemetery (late 2^nd^ - first half of the 3^rd^ century BCE), which is also not negated by the artefacts discovered in it. The burial of a newborn baby (G1C_Ob.38A) dug into this grave (G1C_OB.38) is about a century later.

The medieval skeletons (i.e., of Slavic population) selected for genetic studies were also from the cemetery at site 29 (excavations 2017–2019). Of 15 inhumations then brought to light, 11 were arranged in rows, oriented E-W, with the head pointing west, and radiocarbon dating ranged between the 11^th^ and 12^th^ centuries. A large and deep feature (No. 9), 3x3 meters and 1.5 meters deep, was excavated in 2019. Its fill of several layers produced a modest quantity of pottery and animal bones, but also fragments of cremated human bone. Apparently, burials of infants (neonates, infans I) had been inserted at different depths into the sides of a large pit. They had been deposited inside small pits dug next to the sides of the large feature on its north and east side. The radiocarbon dates obtained for these skeletal elements correspond to the Tribal Period (8^th^ –9^th^ centuries). It should be emphasized that from the Gródek area we also know of individuals with Late Avar finds – additional evidence of settlement activity at the time when feature No. 9 was created.

Numerous burials, including skeletal remains from the Roman Period as well as from the 8^th^ to 10^th^ Century (before the Christianisation of Poles and Rus), a significant series of radiocarbon dates but also the location of the site in the border zone between Western and Eastern Slavs indicate that the site at Gródek may become crucial for studies on demographic and ethnic transformations in East-Central and Eastern Europe during the first millennium AD.

#### Koziarnia Cave (PC1)

Region: Lesser Poland

Coordinates: 50.21"N 19.79"E

Occupation period: 200 CE - 500 CE

Samples: 2

Sample IDs: PC1008, PC1009, PC1016

Sex Ratio: 1F, 2M

Provenance note: Samples from Koziarnia Cave were provided by the Faculty of Archaeology, University of Warsaw, and the Institute of Systematics and Evolution of Animals PAS, Cracow. Permission to analyse was granted to coauthor Zuzana Hofmanová in 2022.

Contact person: Małgorzata Kot, Jarosław Wilczyński

The cave lies in Koziarnia Gorge (Ojców, Skała commune, Cracow district), ca. 10 m above its bottom, and comprises a 90 m long and up to 10 m wide corridor narrowing towards the end, preceded by an arcade-like opening, nowadays quite sizable (7.5 × 6 m), although much smaller in the 1880s, when the first explorations took place at the site. Since then it has been excavated several times, revealing numerous archaeological finds from different periods, from the Palaeolithic up until the 20^th^ century. Notably, the unearthed archaeological assemblages included numerous finds from the Roman and Migration Periods. Regarding Holocene-era human activity, the majority of the cultural assemblages were discovered in the cave entrance zone in the 1950–1960 s by W. Chmielewski, in the humus layer, which was over 1.5 m thick. The same stratum provided many stray human bones, which were recently recognized among the faunal assemblage stored in the Institute of Systematics and Evolution of Animals PAS in Cracow. The archaeological assemblages are partly kept in the Institute of Archaeology and Ethnology, Polish Academy of Sciences. In Koziarnia Cave, precisely dated artifacts were few. Worth mentioning is a massive bronze buckle with a frame thickened on one side, representing type ML.H.15. This type is well known in the millieu of Przeworsk culture, especially at sites placed within the Liswarta basin (southern Poland), such as the layered cemeteries of the Dobrodzień type or the necropolis in Opatów, Kłobuck district, in the last phase of its use early in phase D. The cave also yielded two fragments of an iron sheet in the shape of an arc segment bent up. Although there are no direct indications of their function, one may wonder if these were not parts of a box-shaped ferrule from a large buckle characteristic of the Migration Period), albeit similar ferrule construction (which possibly served for keeping organic amulets or fragrances) appeared as early as phase C2. Among the finds from Koziarnia there are also two glass beads similar to types TM 266–271, which were in use especially during phases C1–D, and a clay cylindrical rattle with a handle and pea seed inside, which was radiocarbon dated to 1705 ± 30 uncal BP. Much less certain is the moment of deposition of a silver denarius of Antoninus Pius, minted in AD 139, already found in the cave in the 1870s.

Among the numerous stray human bones recently identified in the set of faunal assemblage stored in ISEA PAS, five were radiocarbon dated to 1605 ± 30 BP (1473 cal. BP, 1535-1408 95% CI), 1650 ± 30 BP (1534 cal. BP, 1687-1414 95% CI), 1655 ± 30 BP (1540 cal. BP, 1688-1415 95% CI), 1670 ± 30 BP (1561 cal. BP, 1692-1420 95% CI), 1735 ± 30 BP (1623 cal. BP, 1703-1547 95% CI) (Poz-97814, Poz-97812, Poz-97813, Poz-97815, Poz-97816, respectively). Apart from these, a bone belonging to a young individual (Infans I, < 1 y.o.), unearthed during the most recent excavations carried out in the cave in 2017 by M. Kot, was radiocarbon dated to 1745 ± 22 (1636 cal. BP, 1706-1570 95% CI) (MAMS-47132)[^97–102^](https://paperpile.com/c/UPmHk7/iH9xg+jp77G+gXSdZ+Sw5WG+3NiZ2+AYmZy).

#### Żarska Cave (PC1)

Region: Lesser Poland

Coordinates: 50.16"N 19.7"E

Occupation period: 200 CE - 500 CE

Samples: 2

Sample IDs: PC1004, PC1005

Sex Ratio: 2M

Provenance note: Samples from Żarska Cave were provided by the Institute of Archaeology, Jagiellonian University. Permission to analyse was granted to coauthor Zuzana Hofmanová in 2022.

Contact person: Michał Wojenka, Jarosław Wilczyński

The cave is situated within the top part of Żary Gorge (Żary, Krzeszowice commune, Cracow district), ca. 20 m above its bottom, within a group of limestone rocks. This is a large one-chambered cave, around 30 m wide and 15–20 m long, accessed from a wide but very low opening, not visible from a distance. The site was explored in 1879 and was determined, for unknown reasons, as being archaeologically sterile. Archaeological excavations carried out in 2012 and in 2014–2015 allowed this statement to be verified and in fact revealed a rich Holocene-era stratigraphic sequence, recently comprehensively examined. The lower parts of this sequence yielded numerous Eneolithic Baden culture artifacts, while its upper section produced a large amount of Roman/Migration Period and later finds. Finally, it should be noted that the upper humic level yielded numerous disarticulated human remains, which produced radiocarbon dates appropriate to the Late Roman or Early Migration Periods and contributed to the development of the research which is presented here in this paper. The human bones are now kept in the Institute of Systematics and Evolution of Animals PAS in Cracow. Apart from numerous pottery sherds dated to the C1–D phases, Żarska Cave yielded an iron buckle with non-thickened frame, which is probably linked with the Przeworsk cultural environment. It is similar to types ML.H.3–8, occurring in phases C2–D. Human bones belonging to three individuals were radiocarbon dated to the Roman and/or Migration Period and produced following datings: 1755 ± 30 and 1755 ± 30 (1644 cal. BP, 1713-1549 95% CI) (two datings for a single Infans I individual − Poz-58069, Poz-67070); 1660 ± 30 (1546 cal. BP, 1690-1416 95% CI) and 1655 ± 30 (1540 cal. BP, 1688-1415 95% CI) (two datings for a single Infans II individual − Poz-107244, Poz-67069) and 1655 ± 30 (1540 cal. BP, 1688-1415 95% CI) (an adult; Poz-112853). Worth noting is that due to the ^87^Sr/^86^Sr analysis, the individual producing the latter dating (Poz-112853) was of non-local origin (the other two individuals are not determined)[^98,103–105^](https://paperpile.com/c/UPmHk7/9ftYS+PRP9Y+ABjxN+AYmZy).

#### Zbójecka Cave (PC2)

Region: Lesser Poland

Coordinates: 49.69"N 20.27"E

Occupation period: 800 BCE - 600 BCE (PC2001) & 200 CE - 500 CE

Samples: 1

Sample IDs: PC2001

Sex Ratio: 1F

Provenance note: Samples from Zbójecka Cave were provided by the Faculty of Archaeology, University of Warsaw. Permission to analyse was granted to coauthor Zuzana Hofmanová in 2022.

Contact person: Małgorzata Kot

The cave is situated on the right slope of the so-called Jamki Gorge (Ojców, Skała commune, Cracow district), not far from the valley of Sąspówka—a main tributary of the Prądnik river. It is characterized by three entrances to a short, cramped, narrow and descending passage, leading to a small chamber which branches out into two corridors, the right of which is narrow (ca 2–5 m), relatively high (3–4 m) and long (the total length of the cave is 189 m). Worth noting is the main entrance to the cave, which is around 1.6 m high and narrow. This is one of the earliest Polish caves ever examined, but only through amateur excavations, which took place mainly in the cave chamber, in 1871 (J. Zawisza and A. Ślusarski) and 1872 (F. Römer). The excavations produced several artifacts, mainly from the Neolithic and the Roman Age as well. Notably, the 1870s excavations were preceded by a series of accidental discoveries of human bones made by local people. The archaeological assemblages from Zbójecka Cave are stored in the National Museum in Wrocław.

In 2021, the inside of the cave witnessed a surface survey (led by M. Kot), which yielded three human bones belonging to the three different individuals (adult and two Infans I). The bones in question were radiocarbon dated and produced following data: 1695 ± 30 (1584 cal. BP, 1695-1531 95% CI), 1635 ± 30 (1500 cal. BP, 1585-1410 95% CI), 1670 ± 30 (1561 cal. BP, 1692-1420 95% CI) (Poz-150575, Poz-150576 and Poz-150577, respectively)[^97,98,102,106–108^](https://paperpile.com/c/UPmHk7/jp77G+mKJ9g+jzWIl+3NiZ2+AYmZy+MDFSD).

### Northwestern Ukraine

#### Korolivka/Korolówka (KRW)

Region: Western Ukraine

Coordinates: 48.74"N 25.99"E

Occupation period: 1200 CE - 1300 CE

Samples: 13

Sample IDs: KRW001, KRW002, KRW003, KRW004, KRW005, KRW006, KRW007, KRW008, KRW009, KRW010, KRW011, KRW013, KRW014

Sex Ratio: 9F, 4M

Provenance note: Samples from Korolivka were provided by the Laboratory of Anthropology, Institute of Zoology and Biomedical Research, Jagiellonian University, Krakow. Permission to analyse was granted to coauthor Zuzana Hofmanová in 2022.

Contact person: Iwona Wronka, Radosław Liwoch

Korolivka (Ukrainian: Королівка, Polish: Korolówka) is a village in Chortkiv Raion of Ternopil Oblast in western Ukraine. In 1934, a cave (now part of the Optymistychna Cave) was discovered in Korolivka, where human remains and artifacts were found. In the same year, a small-scale survey was carried out under the leadership of Tadeusz Sulimirski, with the main purpose of collecting anthropological (skeletal fragments, including skulls) and archaeological material (a silver ring, a copper alloy belt ring, 3 copper temple rings, an iron axe, 2 glass bracelets, 2 clay pots) from the surface of the passageways, so that they would not be destroyed by casual visitors to the cave. In the places where they were found, a layer of moist and sticky loam was explored to a depth of 40 cm. The human remains did not show a systematic arrangement, but were carried relatively dumped in one place. The finds should be dated to the 1^st^ half of the 13^th^ century. It seems that this cave archaeological site was not an atypical Old Ruthenian cemetery, but a place where the victims of one of the Mongol invasions of western Rus', perhaps the Batu Khan invasion of 1240, lie.

Anthropological material from Korolivka is stored at the Center for Nature Education at Jagiellonian University, while archaeological artifacts are kept at the Department of Old Collections of the Archaeological Museum in Krakow.

#### Pidhirtsi/Podhorce (PDH)

Region: Western Ukraine

Coordinates: 50.78"N 23.78"E

Occupation period: 1000 CE - 1300 CE

Samples: 12

Sample IDs: PDH001, PDH002, PDH003, PDH004, PDH005, PDH006, PDH007, PDH008, PDH009, PDH010, PDH011, PDH012

Sex Ratio: 3F, 9M

Provenance note: Samples from Pidhirtsi were provided by the Laboratory of Anthropology, Institute of Zoology and Biomedical Research, Jagiellonian University, Krakow. Permission to analyse was granted to coauthor Zuzana Hofmanová in 2022.

Contact person: Iwona Wronka, Radosław Liwoch

Pidhirtsi (Ukrainian: Підгірці; Polish: Podhorce) is a village in Zolochiv Raion, Lviv Oblast of Ukraine, located about 80 km east of Lviv. In the hamlet of Plisnesk (Ukrainian: Пліснесько; Polish: Pleśnisko), which is part of the village of Podhorce, there is a medieval (7^th^-13^th^ century) archaeological complex, consisting of an impressive stronghold, settlements and necropolises, including an elite cemetery from the late 10^th^ - 1^st^ half of the 11^th^ century. The site contains artifacts of the Prague culture, the Rayky (Luka-Raykovetska) culture, as well as numerous finds of the Old Ruthenian culture. The stronghold was destroyed around 1240/1241 by the Mongol invasion of Batu Khan. In 1881-1883 the stronghold and cemeteries were excavated by Teodor Ziemięcki. His most important finds came from the so-called great barrows, which are evidence of the westward expansion of the Rus' state during the reign of Kiev Prince Vladimir Svyatoslavich. In the 20^th^ and 21^st^ centuries, excavations at the site were carried out by Ukrainian researchers.

Anthropological material from T. Ziemięcki's excavations is stored at the Center for Nature Education at Jagiellonian University, while archaeological artifacts are kept at the Department of Old Collections of the Archaeological Museum in Krakow.

## 3. Genetic affinities of the pre-SP/MP populations

### 3.1 Croatia

In order to understand the genetic affinities of our new Roman Period Croatian samples, we performed principal components analysis (PCA) (Methods) by projecting the 26 BBC and 97 published ancient genomes[^109^](https://paperpile.com/c/UPmHk7/Ejy7a) from the Northwestern Balkans onto the first two principal components constructed from 940 present-day West Eurasian individuals from the Human Origins (HO) dataset[^110^](https://paperpile.com/c/UPmHk7/yFRI7). A key feature of the present-day data is the presence of a genetic cline running along PC1 and connecting European and West Asian populations. This cline is composed of individuals from present-day southern Italy, Sicily, the Balkan Peninsula, Greece, Cyprus, Malta and Turkey. Late Bronze Age (LBA) and Iron Age (IA) genomes from the Northwestern Balkan (specifically Slovenia and Croatia) cluster to the left of this cline, in between present-day French and Northern Italian individuals and close to ancient Etruscan samples from Italy. In contrast, published Roman and Late Antique genomes from the Northwestern Balkan plot on top of the European-West Asian cline, similar to present-day Greek and Southern Italians, with some outlier individuals being shifted further East along PC1 in the direction of Hellenistic and Byzantine samples from the Anatolian Peninsula[^111^](https://paperpile.com/c/UPmHk7/Roybd) (Fig. S1a).

This observations is consistent with previous ancient DNA analyses on the Imperial and Late Antique Period in Central Italy, Croatia and Serbia, demonstrating an ancestry shift towards Western Asia[^109,112,113^](https://paperpile.com/c/UPmHk7/PvwZ8+cvmfh+Ejy7a) following the Roman empire’s territorial expansion to the East Mediterranean. It might be the result of long-term settlement of people from major Eastern urban centers such as Ephesus, Corinth, or Constantinople, yet, individuals with additional Eastern Mediterranean ancestry are found in smaller frequencies already during the Republic Period, indicating (at least) small-scale mobility between the regions well before the eastward expansion of the Empire[^112,114^](https://paperpile.com/c/UPmHk7/PvwZ8+JZft4).

However, while this signal is present in basically all previously reported Roman colonies (such as Zadar, Sisak, Mursa or Sirmium) and military fortresses (e.g. Tilurium or Emona) on the Northern Balkan Peninsula, we do not observe this shift in our Roman BBC samples. Instead, with the exception of two outliers (BBC022 & BBC023), all genomes plot on top of the preceding Late Bronze Age and Iron Age genetic diversity. Indeed, while the Bubi’s Cave (BBC) genomes have on average significantly lower PC1 coordinates than the published Roman Period individuals from Croatia (Welch Two Sample t-test; *t* = -4.423, *df* = 41.022, *p* = 7.008e-05), their mean PC1 coordinates do not significantly differ from the Late Bronze Age and Iron Age average (Welch Two Sample t-test; *t* = 1.9996, *df* = 42.474, *p* = 0.05197). The maintenance of the same geographical pattern along PC1 points to some degree of local continuity at the site from the Late Bronze Age until the Height of the Roman Empire as well as only minor ancestry contributions from the East Mediterranean.

Since this shift is also accompanied by an increase in Neolithic Iranian-derived ancestry[^111–113,115–117^](https://paperpile.com/c/UPmHk7/G4FfS+PvwZ8+XKskX+Roybd+nlauq+cvmfh), we estimate ancestry proportions for Iron Age and Roman Period sites in Italy and Southeastern Europe. We applied qpAdm[^118,119^](https://paperpile.com/c/UPmHk7/XohM7+vFjQb) to calculate estimates of the mixture coefficients α_WHG_, α_EEF_, α_Steppe_, and αIran_N using the outgroups described in Petterson et al. 2021[^120^](https://paperpile.com/c/UPmHk7/iIPiM) (Fig. S1b, Table S32).

We observe that αIran_N is consistently higher in Roman Period groups compared to Iron Age populations. Specifically, we measure only 2.9% ± 1.9% Iran_N-related ancestry in BBC, yet 19.5% ± 2.9% in Mursa, 29% ± 2.8% in Tilurium, or 16% ± 2.1% in Zadar. Consequently, BBC features an ancestry composition more similar to Croatia_EIA, Slovenia_EIA, Spain_IA, and France_SouthEast_IA2, agreeing with the PCA-based observation of demographic continuity. The qpAdm results are further supported by *F*-statistics: compared to Roman Period groups showing excess Iran_N-related ancestry, Balkan Iron Age populations and the BBC individuals share an equal (|*Z*| < 2) or higher (*Z* > 2) fraction of alleles with present-day Spanish (as proxy for the local, pre-Roman Iron Age ancestry) than with present-day Greeks (as proxy for East Mediterranean ancestry) (Fig. S1c, Table S42). On the other hand, Roman groups from Italy, Croatia, and Serbia are significantly closer related to present-day Greeks than to present-day Spanish (*Z* < -2).

Finally, to directly measure the amount of East Mediterranean and Middle Eastern ancestry in Roman Period Southeastern Europe, we applied our supervised ADMIXTURE[^121^](https://paperpile.com/c/UPmHk7/7NnVp) model at *K* = 12 to 108 Bronze Age, Iron Age, Roman Period, and Late Antique groups from Southern Europe and West Asia. We find that the BBC individuals, as indicated by PCA and qpAdm modeling, feature only minor ancestry contributions from West Asia, specifically 13% ± 2% WAS and 4% ± 1% NEA ancestry, comparable to fractions measured in the preceding Iron Age population (Croatia_EIA: 11% ± 1% WAS and 0% NEA). In contrast, we measure variable yet consistently higher proportions of WAS ancestry in most other Roman Croatian sites[^109^](https://paperpile.com/c/UPmHk7/Ejy7a), e.g. 34%, 53%, 28%, 33% and 22% in Dragulin, Policija, Sipar, Tilurium, and Zadar, respectively. Applying Ward’s minimum variance method to a symmetrical matrix of euclidean distances calculated from the ADMIXTURE ancestry profiles, we show the highest similarity of the BBC genomes with Bronze and Iron Age groups from Austria, Slovenia, and Croatia, while other Roman Croatian sites cluster with Bronze and Iron Age populations from the Southern Balkan (here: Greece, Albania, NMacedonia, Montenegro and Bulgaria) or Western Asia (e.g. Turkey and Israel) (Fig. S2).

Summarizing, we reconstruct considerable variability in the demographic input from the Eastern Mediterranean into Southeastern Europe during Roman times. Ranging between 74% Eastern Mediterranean (WAS + NEA) contribution in Policija (comparable to the proportions measured in Casal Bertone or Grosseto, Italy (67% each) to less than 11% in Mursa, these estimates demonstrate that the genetic impact of immigration from the East did not only affect the Imperial capital and heartland but was widespread across the southeastern extent of the Empire. However, in the case of Croatia and Serbia, large towns, cosmopolitan trade centers and Roman colonies (such as Viminacium/Kostolac or Iadera/Zadar or Mursa/Osijek) might have been impacted more substantially, as some smaller sites exhibit nearly no or only neglectable influx of Eastern Mediterranean ancestry.

Specifically, while this demographic input from the Eastern Mediterranean was profound in most published Croatian sites[^109^](https://paperpile.com/c/UPmHk7/Ejy7a), we measure only a minor contribution of West Asian ancestry to the newly reported population of BBC, indicating that rural areas were less affected by migration from the East than the larger urban centers (Fig. S1b/c,S2). Consequently, the total amount of West Asian ancestry in the Northwestern Balkan might be systematically overestimated due to the focus on cosmopolitan trade centers and Roman necropolises.


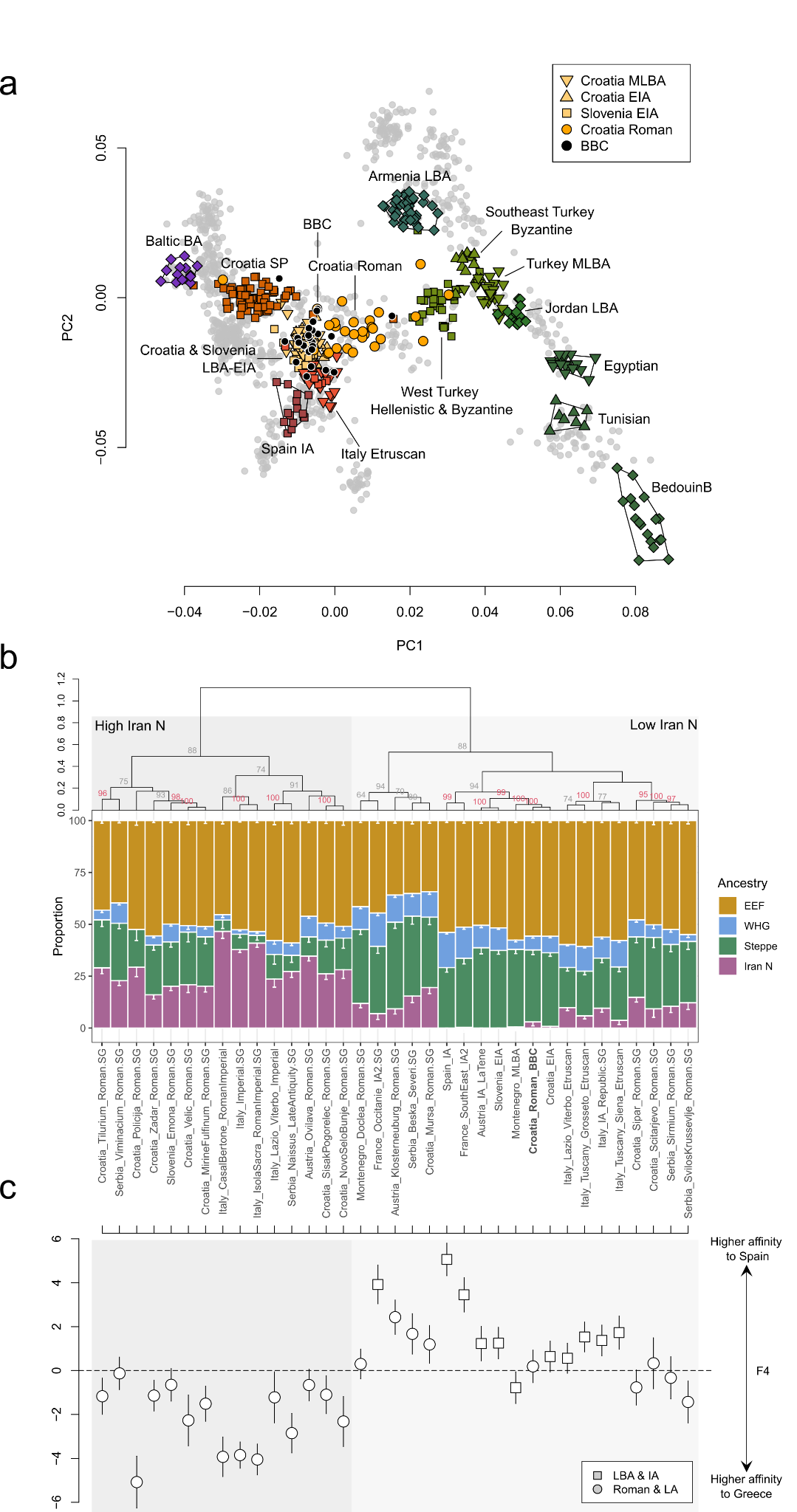


***Supp. Fig. 1. Genetic affinities of the Roman Period Bubi’s Cave population from Croatia****. a) West Eurasian PCA (present-day individuals are depicted as grey background points) with projection of BBC (n = 26) and other relevant Iron Age, Roman, and Late Antique populations (n = 336) (coloured symbols). b) Results from qpAdm analyses. A four-way model was fitted on 35 relevant ancient groups from Southern Europe. Error bars indicate one standard error. Columns were ordered according to hierarchical cluster analysis applying Ward’s minimum variance method. The dendrogram and statistical support for the bifurcations from multiscale bootstrap resampling are shown. c) Results of the F_4_-statistic of the form F_4_(CHB, Test; Greece, Spain) (*1000). The point estimates are shown in the same order as in panel b). Error bars indicate two standard errors.*


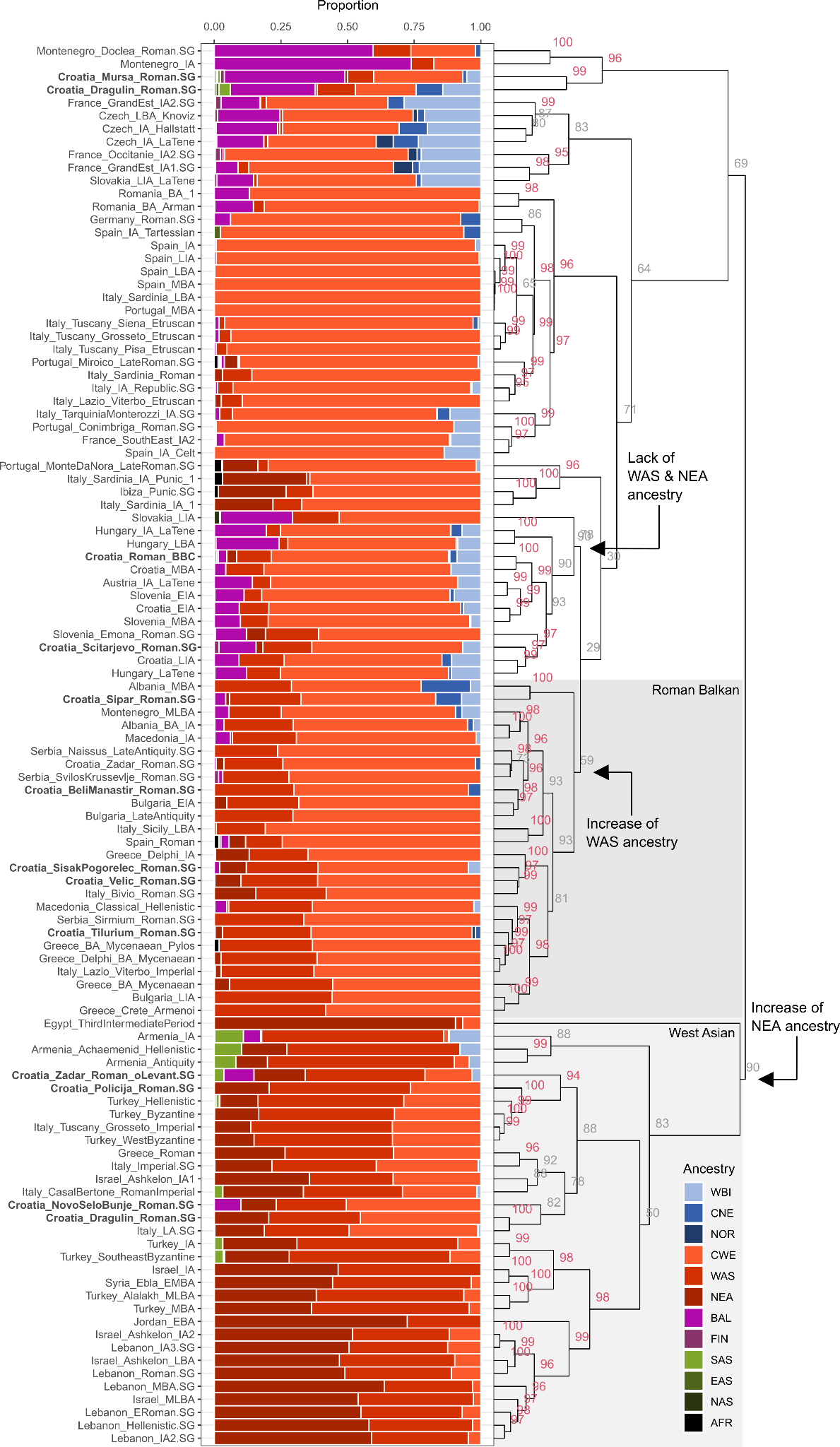


***Supp. Fig. 2. Results from ADMIXTURE analysis.*** *Relevant ancient individuals from southern Europe and the eastern Mediterranean (n = 703) were decomposed into 12 ancestral sources using a supervised clustering approach. Columns were ordered according to hierarchical cluster analysis applying Ward’s minimum variance method. The dendrogram and statistical support for the bifurcations from multiscale bootstrap resampling are shown.*

### 3.2 Eastern Germany

In order to understand the genetic affinities of our new Migration Period (MP) (here: Thuringian Period) samples from Eastern Germany, we performed principal components analysis (PCA) (Methods) by projecting the new BRC, DRH, RTW and OBM individuals and published ancient genomes onto the first two principal components constructed from 940 present-day West Eurasian individuals from the Human Origins (HO) dataset as described earlier[^110^](https://paperpile.com/c/UPmHk7/yFRI7) (Ex. Fig. 2a). Interestingly, similar to the previously published Roman Period samples from Croatia, we observed a notable cline of ancient individuals ranging between present-day Northwestern European (such as Norwegians, Icleanders, Scottish, and Orcadians) and Southeastern European populations from the Balkan Peninsula. While the majority of the Thuringian Period genomes from Eastern Germany cluster together with Northwestern European individuals, a considerable portion of genomes plots on top of the Roman Period Croatian diversity and present-day Balkan groups in general (e.g. BRC005, BRC010, BRC011, BRC012, BRC023, BRC024, BRC029, BRC032, BRC035, BRC051, BRC060, BRC063, DRH023, DRH032, RTW002, RTW003, RTW007, RTW011, RTW012, OBM005, OBM013, OBM017). However, a few individuals are shifted even further along PC1, showing excess affinity to Hellenistic and Byzantine samples from Anatolia (e.g. BRC001, BRC008, BRC017, BRC043, RTW004, RTW006) or Bronze Age genomes from Armenia (e.g. DRH043, BRC046, BRC048).

Although the presence of outlier individuals shifted into the direction of Iron Age genomes from Spain and Italy indicates some degree of heterogeneity among the incoming Southern European ancestries, we highlight that the majority of individuals plot on top of ancient and present-day groups from the Balkan Peninsula. We applied MOBEST[^122^](https://paperpile.com/c/UPmHk7/xUFfF) to perform spatiotemporal interpolation of the genetic affinity of 10 high-coverage (>600,000 SNPs) individuals with more than 50% Southern European/West Asian-derived ancestry (summarizing CWE, WAS and NEA components from ADMIXTURE) to ~5,660 previously published ancient genomes, obtaining similarity probabilities across Roman Period Europe that can be interpreted as proxies for geographical origin. At search time 200 CE, the averaged maximum probability indicates a geographical origin along the Northern Adriatic, either in Northern Italy (>7.5) or Western Croatia (> 10) agreeing with their position in the PCA space (Ex. Fig. 2b). While similar gene pools might have been present also in other territories of the (former) Roman Empire, we can assume that sometime during the Roman to Migration Period a considerable proportion of Southern European/West Asian-related ancestry entered Eastern Germany, most likely via regular interaction with the Roman Empire.

To investigate the extent of this influx of southern European ancestry, we used qpAdm with 4 unadmixed 5^th^ century individuals from Hiddestorf[^123^](https://paperpile.com/c/UPmHk7/tySmG), Lower Saxony, as the local ancestry source and Roman Period individuals from Rome[^112^](https://paperpile.com/c/UPmHk7/PvwZ8), Italy, as proximate sources for newly arriving ancestry. Via this qpAdm[^118,119^](https://paperpile.com/c/UPmHk7/XohM7+vFjQb) model, we infer an αItaly_Imperial of 22% ± 1%, 24% ± 2%, 11 ± 1%, and 24 ± 1% for Brücken (BRC), Deersheim (DRH), Obermöllern (OBM), and Rathewitz (RTW), respectively, indicating a general influx on the scale of ~10% to ~25% (Ex. Fig. 2b).

This affinity to ancient groups from the Balkans and Eastern Mediterranean was furthermore verified using i) group-based F_4_ statistics of the form F_4_(CHB, Test; EGermany_South, EGermany_North) (Table S45), ii) individual-based F_4_ statistics of the same form (CHB, Test; EGermany_North, EGermany_South) (Table S43) and iii) individual-based F_4_ statistics of the form F_4_(CHB, EGermany_South; EGermany_North, Test) (Table S44). The *Test* population iterates here through 50 Iron Age, Roman Period, and early medieval groups from Southwestern and Southeastern Europe as well as the Eastern Mediterranean. Only Individuals with more than 70% South European ancestry (CWE, WAS, NEA) or 90% North European ancestry (WBI, CNE, NOR) are considered and grouped together as EGermany_South and EGermany_North, respectively.

In general, we find that Roman Period groups from Southeastern Europe (e.g. Italy, Croatia, Slovenia) as well as populations of Iron Age and Roman Period West Asia (e.g. Israel, Lebanon, and Turkey) are significantly closer related (*Z* < -2) to the EGermany_South group than the EGermany_North group, agreeing with the high proportions of WAS and NEA ancestry in EGermany_South inferred by ADMIXTURE. On the other hand, pre-Roman Iron Age groups from Slovenia, Croatia and Italy are symmetrically-related to both groups, indicating that the EGermany_South individuals exhibit excess West Asian ancestry that was only introduced in substantial quantities after the expansion of the Roman Empire to the East (Fig. S3).

A similar pattern emerges from the individual F_4_ statistics: Iron Age individuals from Greece (Greece_Delphi_IA), the Lebanon (Lebanon_IA3) and Byzantine individuals from Turkey (Turkey_WestByzantine and Turkey_EarlyByzantine) are on average closest related to EGermany_South than to EGermany_North (Fig. S5). While this demonstrates that EGermany_South individuals exhibit excess affinity to ancient West Asian populations, the majority of their ancestry is however (most likely) Southern European-derived. In particular, in the individuals-based statistics F_4_(CHB, EGermany_South; EGermany_North, Test), we observed the highest genetic similarity between EGermany_South individuals and Iron Age groups from Italy (Italy_Tuscany_Grosseto_Etrsucan and Italy_Lazio_Viterbo_Etrsucan), Croatia (Croatia_EIA), Slovenia (Slovenia_EIA) and Spain (Spain_IA) (Fig. S4). On the other hand, EGermany_South individuals are significantly closer related (*Z* < -2) to the EGermany_North individuals than to West Asian groups such as Turkey_IA, Lebanon_ERoman, or Turkey_SoutheastByzantine.

Consequently, we suggest that the EGermany_South individuals feature variable proportions of ancestry from population(s) with dominantly Iron Age Southern European ancestry but also excess affinity to the Eastern Mediterranean/West Asia. Such an ancestry composition is characteristic for the Iron Age populations of the Southern Balkans (e.g. Albania, Greece, North Macedonia and Bulgaria) or later Roman Period groups in Italy and the Northern Balkans (who received additional West Asian-related ancestry after the incorporation into the Roman Empire[^112,113^](https://paperpile.com/c/UPmHk7/PvwZ8+cvmfh)).


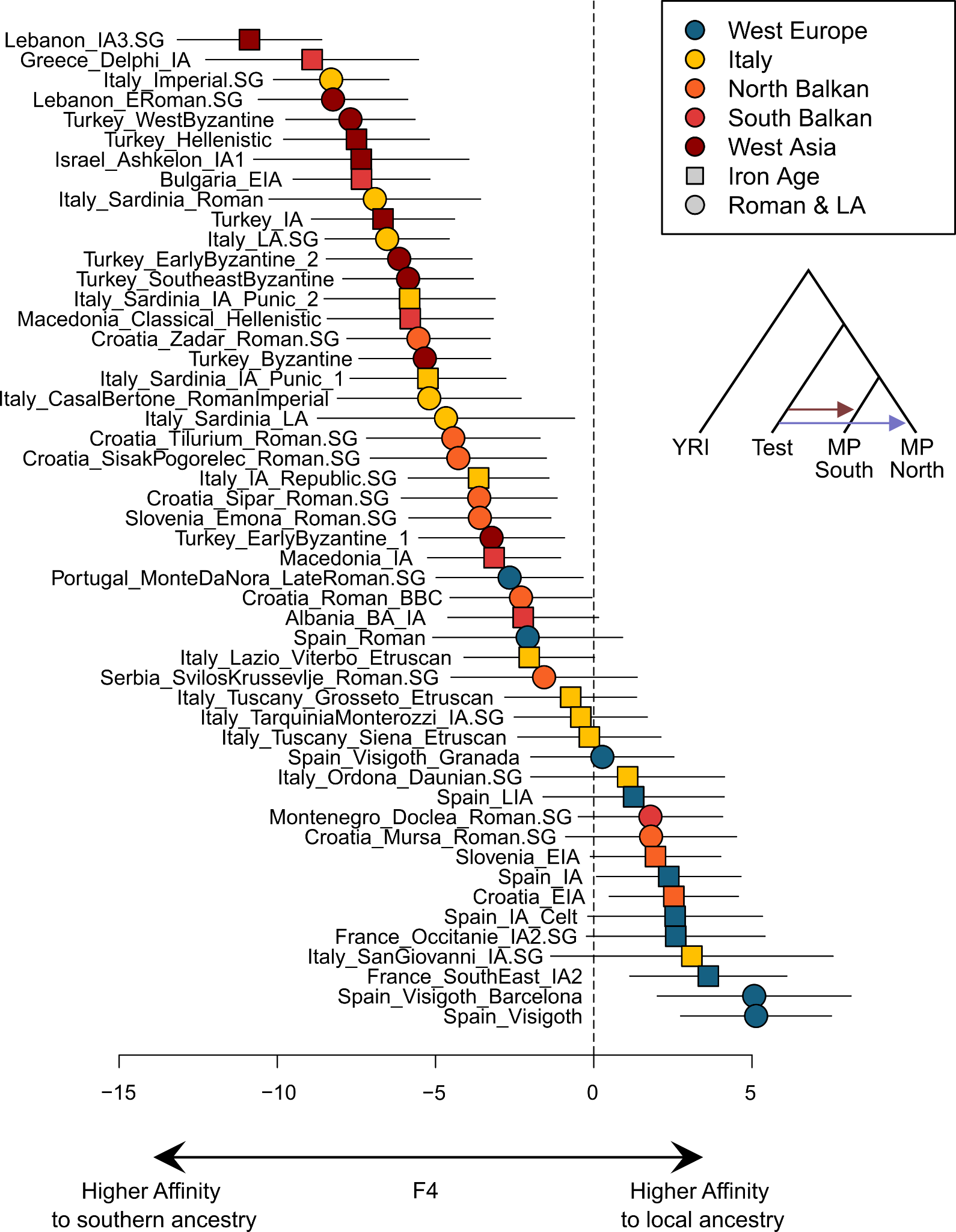


***Supp. Fig. 3. Genetic affinities of individuals from Eastern Germany with southern European ancestry.*** *Shown are the results of the F_4_-statistics of the form F_4_(YRI, Test; EGermany_MP_South, EGermany_MP_North) for 50 relevant ancient southern European and eastern Mediterranean populations. Only Individuals with more than 70% southern European ancestry (CWE, WAS, NEA) (n = 23) or 90% northern European ancestry (WBI, CNE, NOR) (n = 67) are considered and grouped together. Error bars indicate two standard errors.*


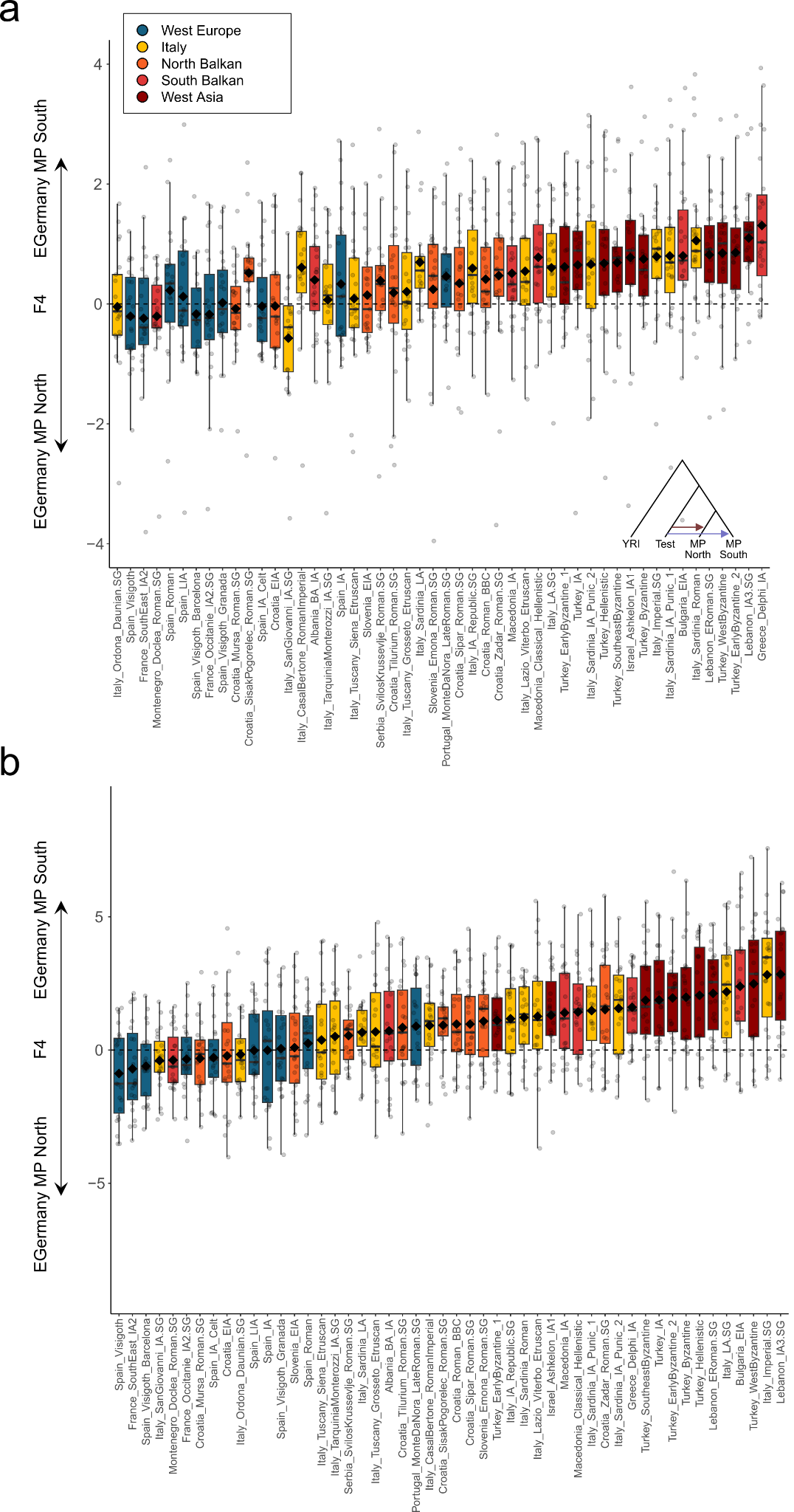


***Supp. Fig. 4. Genetic affinities of individuals from Eastern Germany with southern European ancestry.*** *Shown are the individual results of the F_4_-statistic of the form F_4_(YRI, Test; EGermany_MP_North, EGermany_MP_South) for 50 relevant ancient southern European and eastern Mediterranean populations for 23 BRC, DRH, OBM, and RTW individuals with more than 70% South European ancestry (CWE, WAS, NEA) as boxplots. Bounds of the Box represent the 25^th^ and 75^th^ Percentile. The center represents the median. Whiskers represent the smallest value greater than the 25^th^ Percentile minus 1.5 times the interquartile range and largest value less than the 75^th^ Percentile plus 1.5 times the interquartile range, respectively. Outliers present the minimum and maximum values in the data. b) Shown are the corresponding Z-values for each of the F_4_-statistics.*


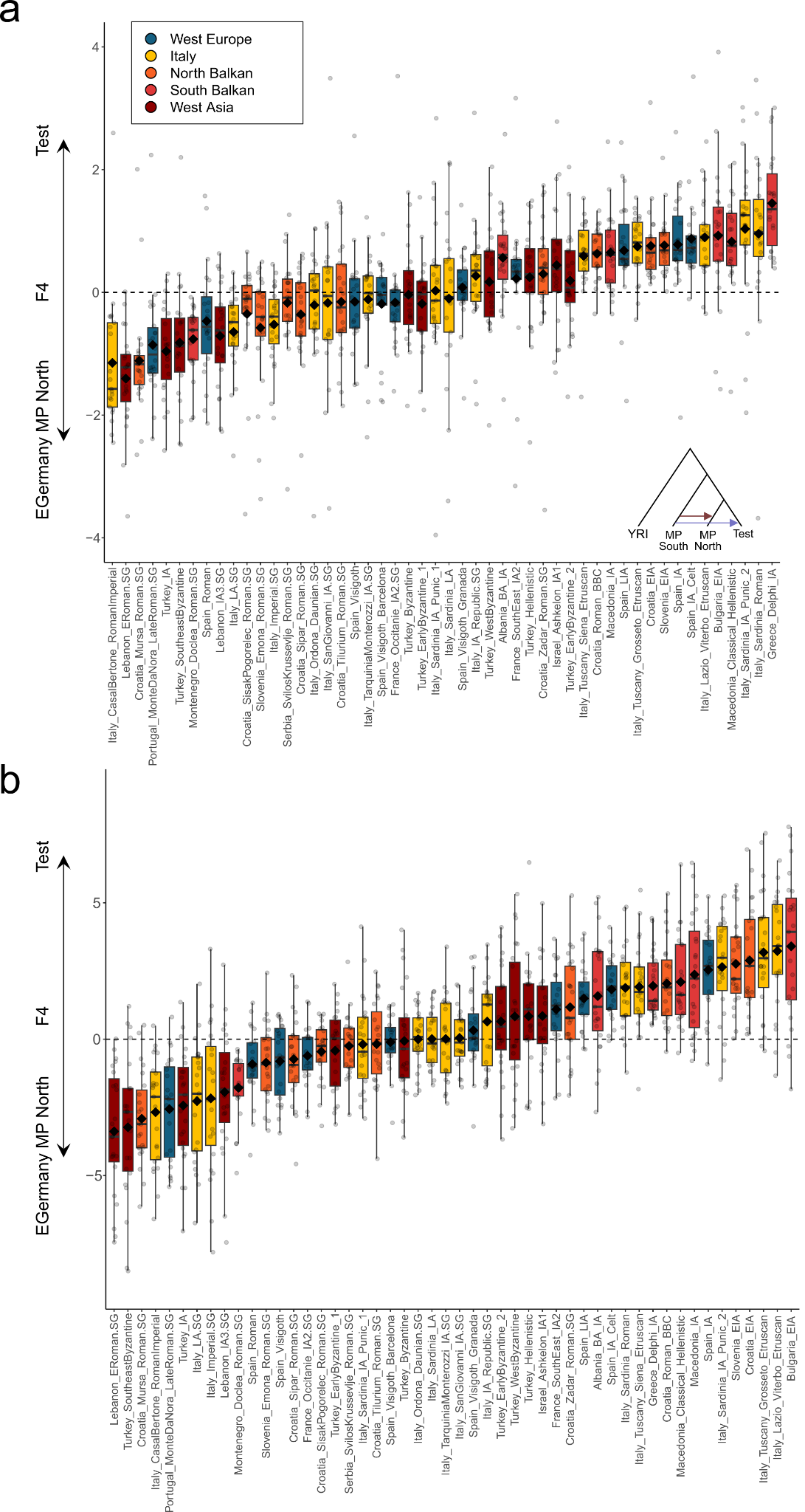


***Supp. Fig. 5. Genetic affinities of individuals from Eastern Germany with southern European ancestry.*** *a) Shown are the individual results of the F_4_-statistic of the form F_4_(YRI, EGermany_MP_South; EGermany_MP_North, Test) for 50 relevant ancient South European and East Mediterranean populations for 23 BRC, DRH, OBM, and RTW individuals with more than 70% South European ancestry (CWE, WAS, NEA) as boxplots. Bounds of the Box represent the 25^th^ and 75^th^ Percentile. The centre represents the median. Whiskers represent the smallest value greater than the 25^th^ Percentile minus 1.5 times the interquartile range and largest value less than the 75^th^ Percentile plus 1.5 times the interquartile range, respectively. Outliers present the minimum and maximum values in the data. b) Shown are the corresponding Z-values for each of the F_4_-statistics.*

### 3.3 Poland

In order to understand the genetic affinities of the Iron Age/Roman Period samples from Poland, we performed principal components analysis (PCA) by projecting the new 9 Gródek and 6 Polish Cave individuals as well as published ancient genomes[^109,124^](https://paperpile.com/c/UPmHk7/LbEH5+Ejy7a) from Northern Europe onto the first two principal components constructed from 9,603 present-day Northern European individuals as well as 10,528 present-day European individuals from the European (EU) dataset (Fig. S6a). We observe that our Roman Period genomes from Gródek as well as previously published Iron Age genomes from Poland (mostly associated with the Wielbark culture) cluster relatively homogeneously on top of present-day Swedes and Norwegians and do not overlap with present-day Poles or other Balto-Slavic-speaking populations. Specifically, they also plot closely together with Iron Age genomes from Sweden and Norway[^125^](https://paperpile.com/c/UPmHk7/ZKQ13) and, to a lesser degree, with Roman Period individuals from Northern Germany (e.g. Häven, Hiddestorf, and Issendorf)[^123^](https://paperpile.com/c/UPmHk7/tySmG). The only exceptions from this pattern are the three individuals PCA0002, PCA0451 and PCA0494, who plot within the diversity of SP and present-day genomes from Poland and the Baltics. While PCA0451 and PCA0494 are not ^14^C dated and thus might be actually younger (potentially dating to the SP), PCA0002 is conclusively dated to the Roman Period via relative PCA0037 (85-235 CE 95% CI, both from Kowalewko). Interestingly, PCA0037 plots close to the (Scandinavian-like) main cluster of the Polish Roman Period group, yet is slightly shifted along PC2 into the direction of PCA0002 as well as of Iron Age and present-day Baltic groups. Consequently, the two relatives most likely attest ongoing admixture between groups associated with the Wielbark culture and their Eastern neighbours in the Baltics. Other examples of such Scandinavian-Baltic admixture might be PCA0103 (Masłomęcz) and PCA0032 (Kowalewko), who are likewise located in between the Scandinavian and Baltic clusters.

The excess affinity of the Polish Roman Period population to Northwestern European groups (compared to Northeastern Europeans) is also evident in quantitative measurement of genetic affinity. Both F_ST_ and IBD-sharing statistics demonstrate that the published Wielbark genomes[^109,124^](https://paperpile.com/c/UPmHk7/LbEH5+Ejy7a) and novel Gródek individuals are genetically most similar and closest related to present-day Germanic-speaking populations, especially Swedes. Precisely, the Wielbark culture genomes show the lowest F_ST_ estimates to Swedes (0.026248 ± 0.000236), followed by Danish, Norwegians, Northern Germans, Dutch and English (0.02706 ± 0.000245) (Fig. S6b, Table S24). In contrast, F_ST_ with Slavic-speaking populations is considerably higher, e.g. Poles (0.02869 ± 0.000274) or Ukrainians (0.028213 ± 0.000548). The same pattern is evident in terms of IBD-sharing, with the Wielbark individuals sharing on average 5.02 ± 0.02 cM IBD segments with present-day Swedish and 4.61 ± 0.03 cM IBD segments with present-day Danish, yet only 3.41 ± 0.11 cM with present-day Poles or 3.17 ± 0.17 with present-day Ukrainians (Fig. S7c). Noteworthy, we observe a similar signal comparing to ancient groups: The Iron Age genomes from Poland share 5.52 ± 0.28, 5.44 ± 0.31, and 5.29 ± 0.19 cM IBD with Roman/Late Antique individuals from Häven[^123^](https://paperpile.com/c/UPmHk7/tySmG) (Northern Germany), Hiddestorf[^123^](https://paperpile.com/c/UPmHk7/tySmG) (Northern Germany), and Obermöllern (Eastern Germany), but only 2.78 ± 0.05 cM with the successive Slavic Period population of Poland (including also 15 SP genomes from Gródek)[^124^](https://paperpile.com/c/UPmHk7/LbEH5) (Fig. S7c).

When applying our supervised ADMIXTURE[^121^](https://paperpile.com/c/UPmHk7/7NnVp) model at *K* = 12 to 46 Bronze Age, Iron Age, Roman Period, and early medieval groups from Central and Northern Europe, we find that Wielbark individuals exhibit on average 54% ± 4% NOR (Northern Scandinavian) and 21 ± 3% CNE (Southern Scandinavian or North Sea coast) ancestry, but only neglectable fractions of BAL ancestry (7% ± 2%), which is the most dominant ancestry among genomes from the succeeding Slavic period (63% ± 2%) (Fig. S7a). An almost identical pattern is seen in Gródek where we observe a pronounced shift in ancestry, from 43% ± 11% NOR, 32% ± 8% CNE and 14% ± 6% BAL ancestry to 2% ± 1% NOR, 7% ± 2% CNE, and 65% ± 4% BAL ancestry, suggesting a major population change at the site. Applying Ward’s minimum variance method to a symmetrical matrix of euclidean distances calculated from the ADMIXTURE ancestry profiles, we observe the highest ancestry similarity between our new Gródek samples and the published Wielbark samples within the diversity of ancient populations from the Scandinavian Peninsula (e.g. Denmark_LN, Denmark_IA, Sweden_EarlyViking, Sweden_IA, Norway_IA, Sweden_Late_N, and Norway_Viking).

We applied MOBEST[^122^](https://paperpile.com/c/UPmHk7/xUFfF) to perform spatiotemporal interpolation of the genetic affinity of 10 high-coverage (>600,000 SNPs) Wielbark culture genomes to ~5,660 previously published ancient genomes, obtaining similarity probabilities across Roman Period Europe that can be interpreted as proxies for geographical origin (Fig. S7b). At search time 0 CE, the averaged maximum probability indicates a geographical origin in Scandinavia, especially Central and Southern Sweden (> 6), agreeing with their position in the PCA space.

Interestingly, our 1 Early Iron Age and 5 Roman Period samples (mostly associated with the Przeworsk culture) from 4 Cave sites located in Southeastern Poland (dating between 800 BCE and 500 CE) (Fig. S8b) do not show this Northern Scandinavian affinity. Instead, they are shifted in PCA along PC1 to the south, in the direction of Iron Age genomes from the Netherlands and Eastern Germany (Fig. S6a, 8a). And indeed, 5 out of the 6 individuals show ancestry compositions mostly represented by Southern Scandinavian CNE (32% ± 9%) and complemented by minor contributions from the South (CWE) and East (BAL) (Fig. S8a). Only the oldest sample (PC2001), which dates to 800-600 BCE, appears to be different, featuring mainly Southern European CWE (~67%) and Eastern European BAL (33%) ancestry. This might indicate that Scandinavian (specifically: Swedish-related ancestry) did not reach the Southeast of Poland during most of the Early Iron Age and arrived only during the later Roman Iron Age. Most importantly, our results suggest that sometimes after the Middle Bronze Age, Scandinavian-related ancestry from Sweden reached the North of Poland and subsequently spread over most of its present-day territory. This would agree well with the traditional interpretations that equate the emergence of the Wielbark culture with the expansion of the Goths who originated from Scandinavia according to Jordanes’ Getica (4.25)[^126–128^](https://paperpile.com/c/UPmHk7/utjsU+GkusC+woc5j). Furthermore, our results indicate that the area of present-day Poland, especially the South, was genetically heterogeneous during the Iron Age and Roman Period and home to diverse groups with Scandinavian, Central- and Eastern European ancestries.


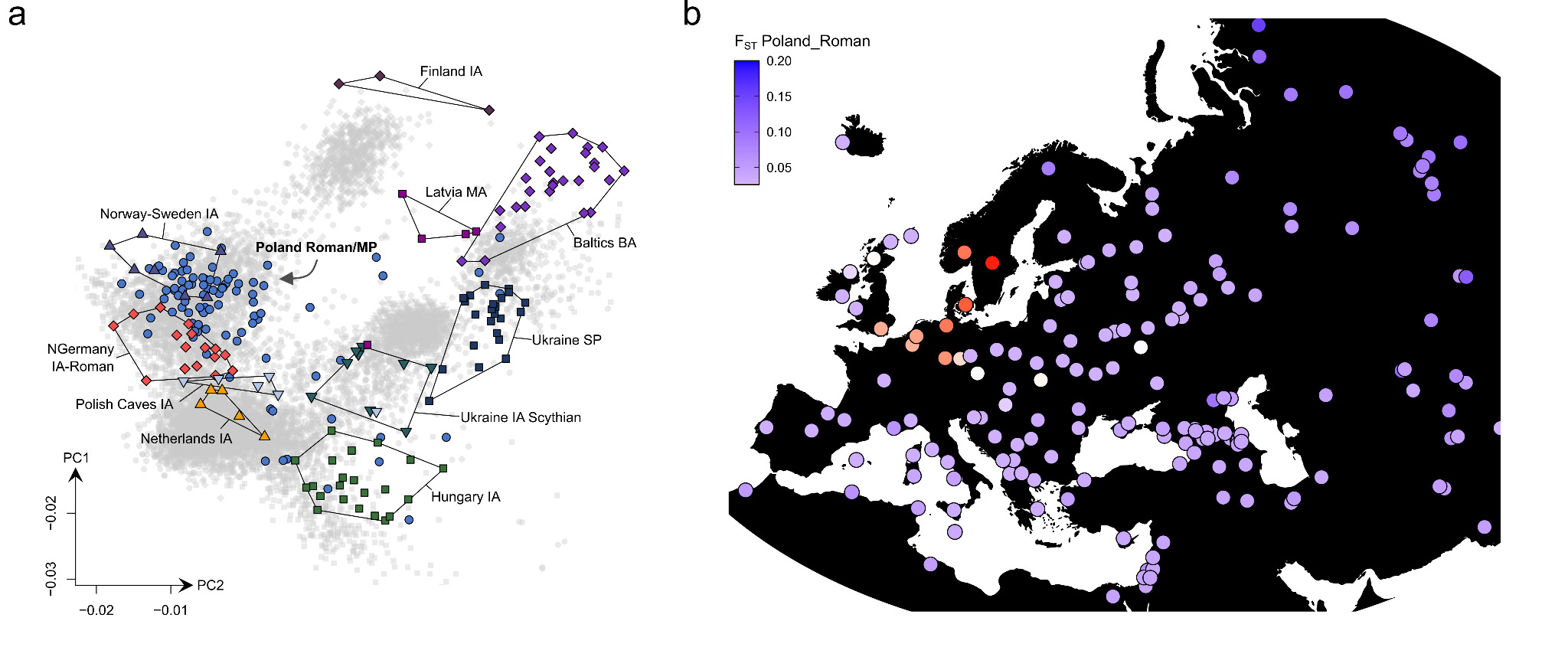


***Supp. Fig. 6. Genetic affinities of the Roman Iron Age and Migration Period population from Poland.*** *a) Northeast European PCA (present-day individuals are depicted as grey background points) with projection of novel and previously published IA/MP individuals from Poland (n = 100) and other relevant Bronze and Iron Age populations (n = 89) (coloured symbols). b) F_ST_ point estimates between the MP population of Poland (n = 94) and 215 present-day European, Asian, and North African populations.*


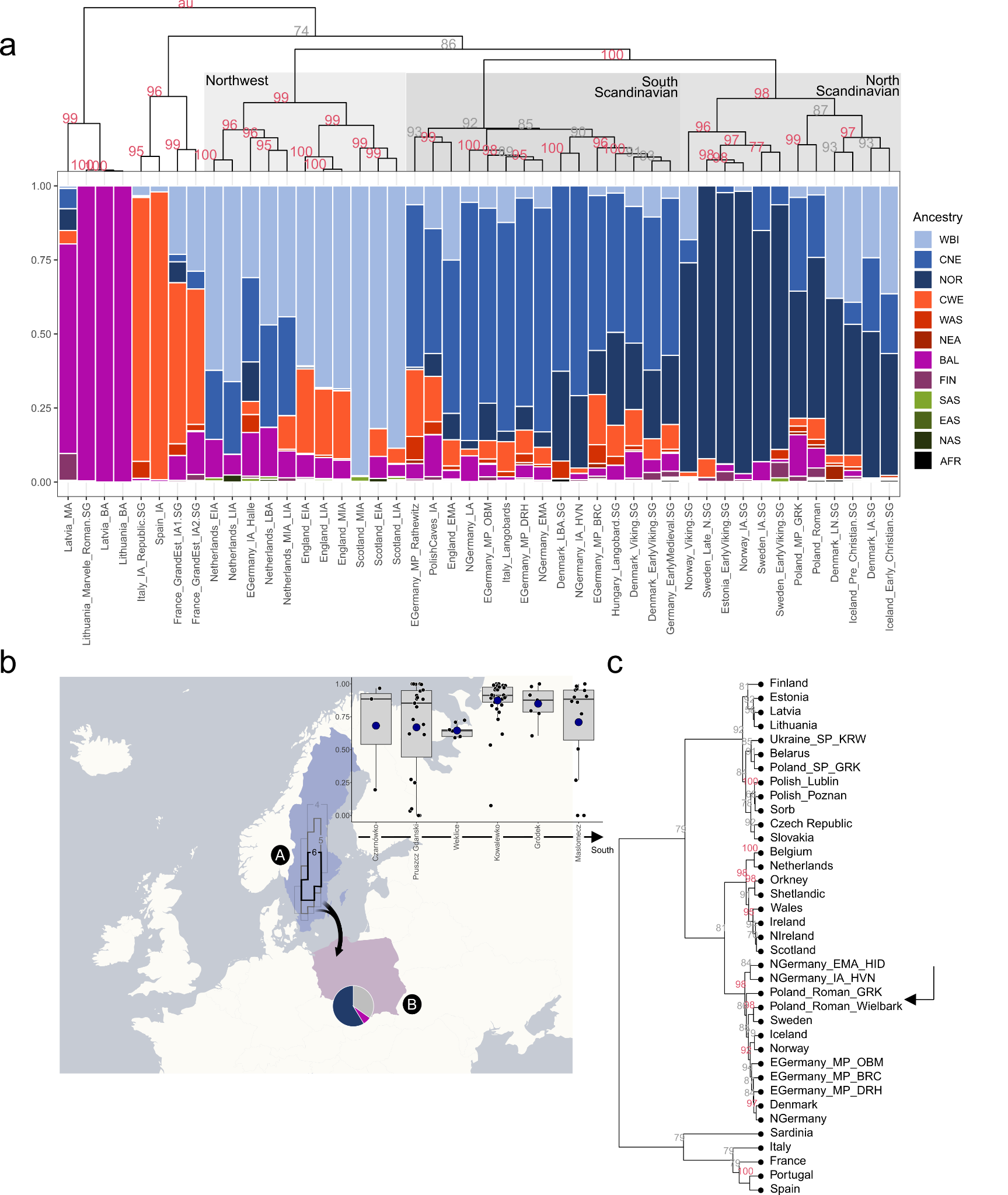


***Supp. Fig. 7. Genetic affinities of the Roman Iron Age and Migration Period population from Poland.*** *a) Results from supervised ADMIXTURE analysis. Relevant ancient individuals from Central, Western and Northern Europe (n = 1147) were decomposed into 12 ancestral sources using a supervised clustering approach. Columns were ordered according to hierarchical cluster analysis applying Ward’s minimum variance method. The dendrogram and statistical support for the bifurcations from multiscale bootstrap resampling are shown. b) Schematic overview of the geographic origin of previously published MP individuals from present-day Poland (n = 94). The contours indicate the averaged maximum probability at search time 100 CE (multiplied by 100) for 10 individuals (denoting the mean prediction of the geographic regions where the ancestors of these individuals originated) as inferred using MOBEST. Averaged ancestry estimates from supervised ADMIXTURE analyses are shown as pie charts for all Roman Period individuals. NOR ancestry is shown in blue, BAL ancestry in violet, and other ancestry is indicated in grey. A boxplot comparison of Scandinavian-related ancestry (CNE+NOR) across sites (ordered by Latitude) is indicated, showing no substantial differentiation among a North-South gradient. c) Hierarchical cluster analysis applying Ward’s minimum variance method to the normalized, average IBD sharing between 37 relevant ancient and present-day populations from Northeastern and Northwestwen Europe. The dendrogram and statistical support for the bifurcations from multiscale bootstrap resampling are shown.*


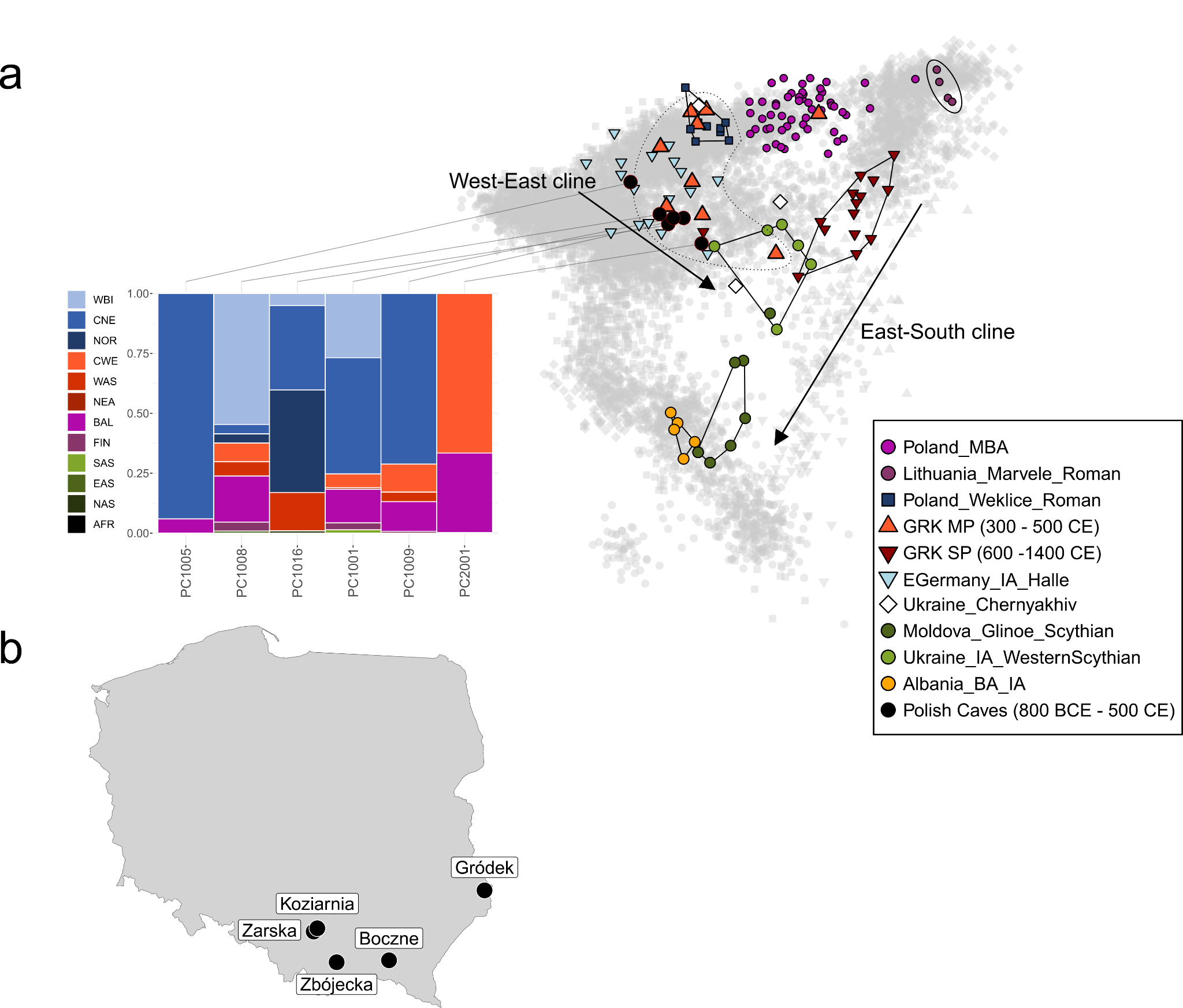


***Supp. Fig. 8. Genetic affinities of the Early and Roman Iron Age individuals from Poland.*** *a) European PCA (present-day individuals are depicted as grey background points) with projection of novel and previously published MBA/IA/MP/SP individuals from Poland (n = 111) and other relevant Bronze and Iron Age populations (n = 42) (coloured symbols). Novel samples from diverse Polish Caves (n = 6) are shown in black and their ancestry profiles from supervised ADMIXTURE analysis are indicated. b) Spatial distribution of Polish IA and MP sites analysed in this study.*

### 3.4 Volga-Oka area

In order to understand the genetic affinities of the published Iron Age samples from the Russian Volga-Oka area[^129^](https://paperpile.com/c/UPmHk7/OnSBn), we performed principal components analysis (PCA) (Methods) by projecting the published ancient genomes onto the first two principal components constructed from 940 present-day West Eurasian individuals from the Human Origins (HO) dataset as described earlier[^110^](https://paperpile.com/c/UPmHk7/yFRI7) (Fig. S9a). Agreeing with earlier results[^129^](https://paperpile.com/c/UPmHk7/OnSBn), we observe that the Iron Age genomes are shifted along PC2 outside the present-day Northeastern European genetic diversity into the direction of present-day Siberian populations such as Chuvash and Mansi and ancient genomes from Bolshoy Oleni Ostrov in the Murmansk Region of the Kola Peninsula[^130^](https://paperpile.com/c/UPmHk7/SCCUR). In contrast, most later (early medieval) genomes from the region are shifted along PC2 to the South, with the majority of samples clustering together with present-day Balto-Slavic-speaking populations.

We investigate the increased affinity to Eastern European populations during the Slavic period using F_4_ statistics of the form F_4_(YRI, Test; Polish, Finnish), where present-day Poles approximate the incoming Eastern European ancestry and present-day Finnish the local Uralic gene pool, respectively (Table S46). Indeed, all Iron Age individuals (with the exception of one outlier who is also shifted in PCA space) are symmetrically related to present-day Finns and Poles (Fig. S9b). In contrast, during the Middle Ages, most samples are significantly closer related to Poles than to Finns, evidencing an influx of ancestry from the West, specifically the area between the Baltic Sea and the Carpathian Arc, into the Volga-Oka area after the Iron Age.

However, of the early medieval samples only eight individuals overlap with the SP genetic diversity observed in Eastern Germany (and Poland) (GOS001d, GOS002d. GOS003d, SHE001d, SHE005d, SHE007d, SHE008d, SHE009d), yet four cluster in between the Volga-Oka Iron Age and Eastern German SP groups (SHE002d, SHE004d, SHE006d, GOR001d), one plots with the preceding Iron Age genomes (SHE003d), and two show substantial affinity to Central and Eastern Asian populations (SHK001d, SHK002d), indicating increased diversity and ongoing admixture during the Slavic Period. To assess these demographic changes quantitatively, we applied our supervised ADMIXTURE[^121^](https://paperpile.com/c/UPmHk7/7NnVp) model at *K* = 12 to the 31 Iron Age and medieval genomes (Fig. S9c). Consistent with PCA, the Iron Age gene pool is dominated by Baltic (BAL), Finnish (FIN) and Sibirian (NAS) ancestries (65% ± 2%, 17% ± 1%, and 15% ± 1%). During the early Middle Ages, these three components decreased substantially (to 55% ± 7%, 8% ± 2% and 8% ± 3%) (Wilcoxon rank sum test; *W* = 118, *p* = 0.002845 for FIN, *W* = 116, *p* = 0.004125 for NAS), yet small proportions of other, Western European ancestry appear which were previously completely absent (e.g. CNE, NOR, CWE & WAS, all between 3% and 6%). The presence of these more distant ancestry components clearly indicates that the incoming SP ancestry must have come from further West and was already mixed with more Southern and Western ancestries. However, we also identify two outliers (SHK001d, SHK002d) with predominantly Central/Eastern Asian-related ancestry, both from the site Shekshovo, who have been previously genetically associated with Kazakhs, Karakalpaks, Siberian Tatars, and other Turkic-speaking groups from Central Asia and Siberia. Consequently, we have to assume that immigrants from more distant regions in the East arrived as well in the Volga-Oka area after the Iron Age, resulting in a genetically more diverse SP population than contemporary groups in Eastern Germany and Poland/Ukraine. Furthermore, considering the intermediate location of present-day Russian and Mordovian individuals between SP samples from EGermany/Poland and IA samples from the Volga-Oka area in PCA space, we have to assume that SP individuals did not represent the vast majority of the population during the Middle Ages and did not completely replaced the Iron Age population. Instead, the integration and admixture with the local population appear to have been crucial in shaping the present-day gene pool of Northwestern Russia.


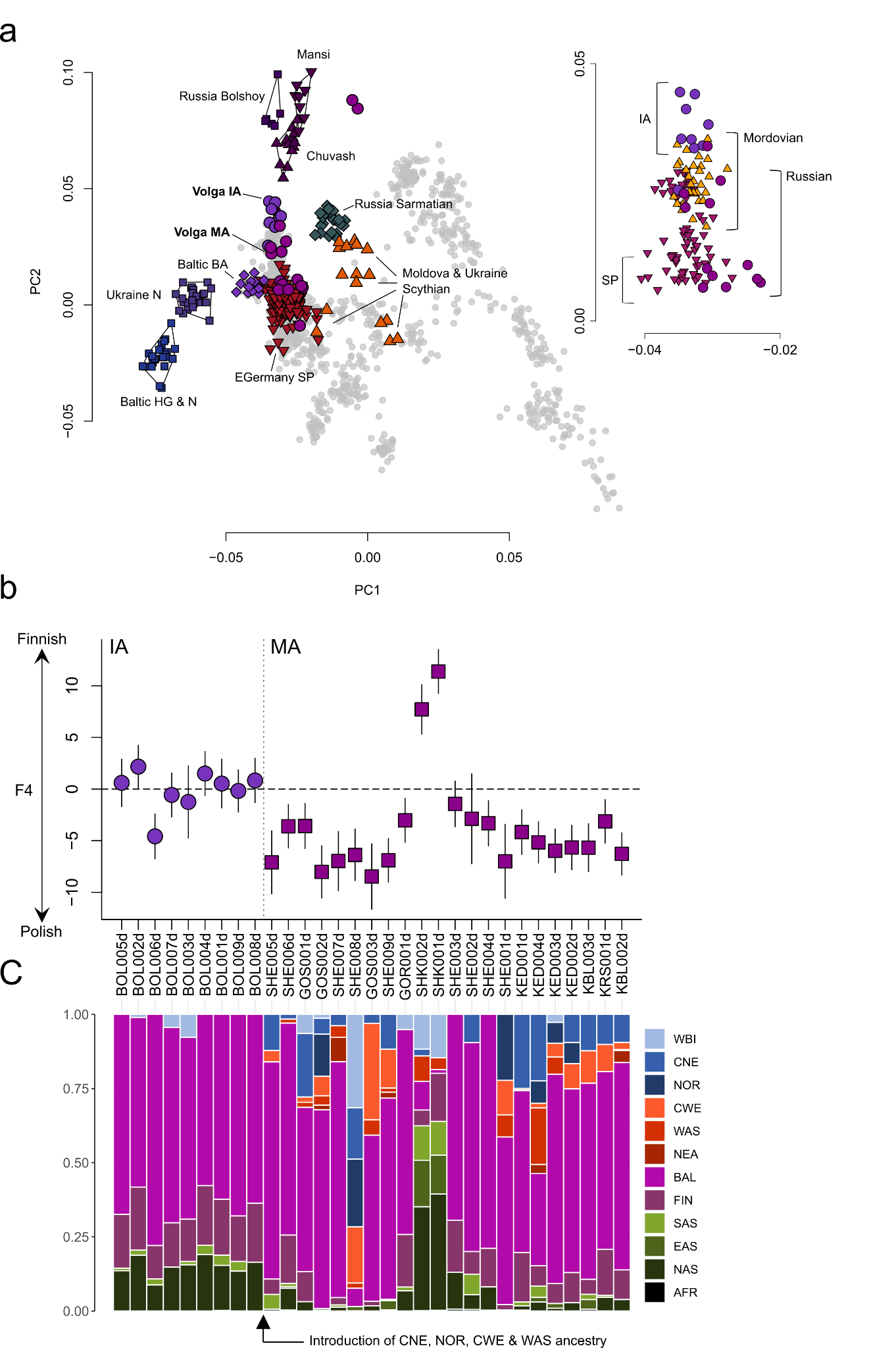


***Supp. Fig. 9. Genetic affinities of the pre-SP and SP populations from the Russian Volga-Oka region.*** *a) West Eurasian PCA (present-day individuals are depicted as grey background points) with projection of Volga-Oka samples (n = 24) and other relevant ancient (n = 335) and present-day (n = 24) individuals (coloured symbols). The inlet to the right visualises the diversity of IA (n = 9) and SP individuals (n = 15) as well as present-day Russians (n = 71) and Morodovians (n = 32) in PCA space. b) Results of the F_4_-statistic of the form F_4_(YRI, Test; Poland, Finland). The point estimates are ordered chronologically. Error bars indicate two standard errors.*

## 4. Genetic affinities of the SP population

### 4.1 PCA & ADMIXTURE

#### 4.1.1 PCA

After establishing an understanding of the pre-SP population structure of the three study transects, we proceeded to investigate the SP gene pool. First, we performed principal components analysis by projecting the pre-SP and SP genomes from the sampling transects onto the first two principal components constructed from 10,528 present-day European individuals from the European (EU) dataset (Fig. S10, S12). As described earlier, we observe considerable genetic structure across the three transects with MP genomes from the Northwestern Balkan plotting on top of present-day Italians, MP genomes from Eastern Germany on top of present-day Northern Germans, Dutch, and Danish, and MP genomes from Poland on top of present-day Norwegians and Swedes. Additionally, we also include IA genomes from the Russian Volga-Oka area, which cluster together with present-day Finns (Fig. S10, S12).

In contrast to the MP individuals, the later SP genomes from these four transects are significantly shifted along PC2 to the East (except the individuals from the Volga-Oka region who are significantly shifted to the West) (Wilcoxon rank sum test; *W* = 163, *p* < 2.2e-16 for the Northwestern Balkan, *W* = 1803, *p* < 2.2e-16 for Eastern Germany, *W* = 1963, *p* < 2.2e-16 for Poland-Northwestern Ukraine, and *W* = 155, *p* = 0.01571 for the Volga-Oka region) and plot nearly exclusively on top of present-day Slavic-speaking populations such as Polish, Belarussians, Russians, and Ukrainians. Specifically, most samples cluster in between the genetic diversities of present-day Sorbs (from Saxony, Eastern Germany) and Lithuanians, showing the highest similarity in PCA space to present-day Poles (Fig. S11). Although this general pattern is similar across all four study transects, subtle substructure is evident: While SP genomes from Eastern Germany, Poland, and Ukraine cluster rather homogeneous (with SP genomes from Eastern Germany being shifted slightly along PC2 into the direction of present-day Germans, Danish and Dutch), we show that SP genomes from Croatia are shifted notably along PC1 to the South in the direction of the MP population of Croatia. Likewise, the SP genomes from the Russian Volga-Oka area are shifted along PC2 to the East, in the direction of their respective Iron Age pre-SP predecessors. Consequently, we can assume that the SP populations of Eastern Germany and Poland underwent only minor admixture with the local MP populations, while the excess affinity to the IA/MP populations in Croatia and Northwestern Russia suggest a notable degree of mixture with the preceding population.

This is furthermore reflected in the variance of MP and SP genomes in PCA space (Fig. S10). For example, the variance in PC1 locations, indicative of Northern-Southern European admixture and mobility, significantly decreases in Eastern Germany and Poland-Northwestern Ukraine from the Migration Period to the Slavic Period (F-test; *p* < 2.2e-16 for Eastern Germany and *p* < 2.2e-16 for Poland-Northwestern Ukraine; we detect no such difference for the Northwestern Balkan, *p* = 0.9791). On the other hand, we observe more variance in PC2 locations (indicative of East-West contact) during the Slavic Period than during preceding Migration Period (F-test; *p* = 0.003405 for the Northwestern Balkan, *p* = 0.004636 for Eastern Germany, *p* = 0.001179 for Poland-Northwestern Ukraine, and *p* = 0.002299 for the Volga-Oka area). Furthermore, PC2 (East-West) variation was significantly higher than PC1 (North-South) variation in all four study transects during the Slavic Period (F-test; *p* = 0.0006071, *p* < 2.2e-16, *p* = 5.798e-14, *p* = 0.0008574 for the Northwestern Balkan, Eastern Germany, Poland-Northwestern Ukraine, and the Volga-Oka area, respectively).

This suggests that the genetic diversity of the SP populations in all four study transects was mostly the product of contacts between East and West. This deviates from the pattern observed in the preceding Late Antique/Migration Period populations, where the genetic diversity was mostly structured by North-South contacts.


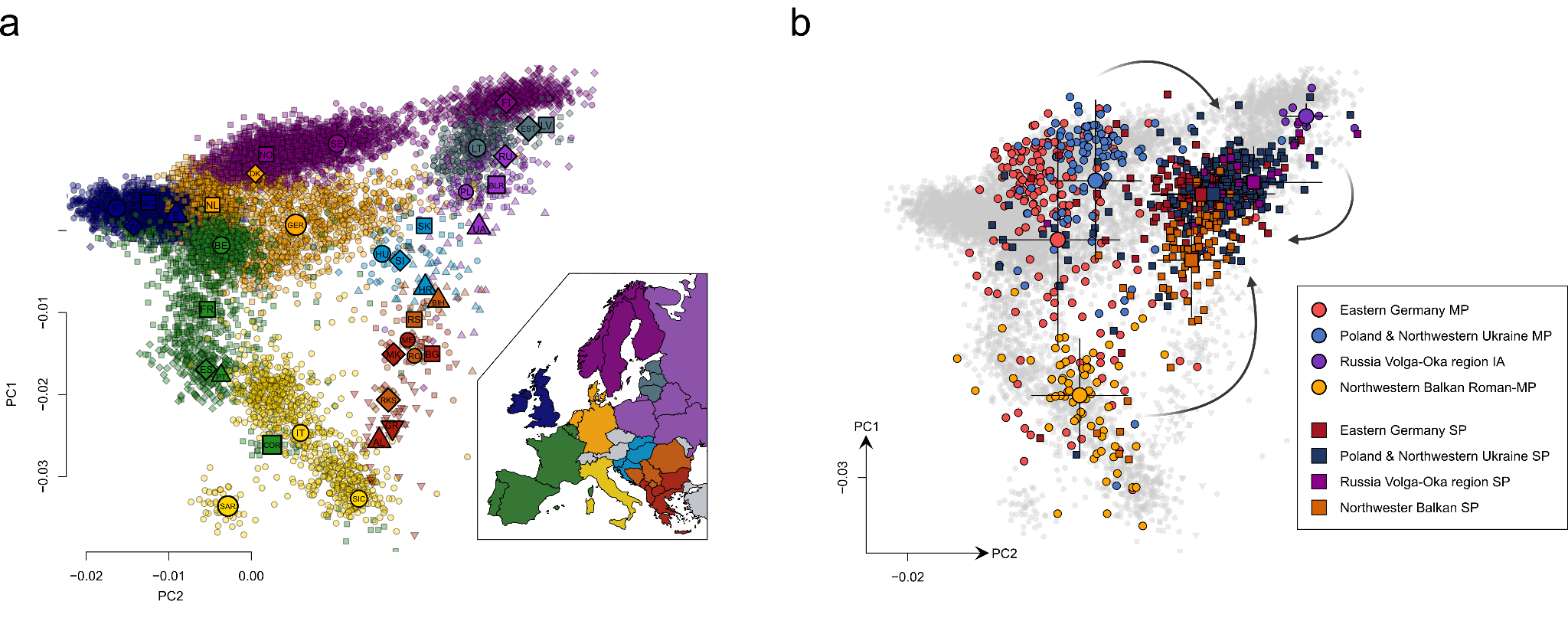


***Supp. Fig. 10. Population structure in central European pre-Sp and SP individuals.*** *a) The reference PCA computed using 10,528 present-day Europeans. The large symbols indicate the mean PC1 and PC2 coordinates of the respective population. The map to the right visualizes the geographical origin of the genomes. b) MP (n = 67, 181, 100, 9, resp.) and SP (n = 80, 240, 207, 15, resp.) genomes from the Northwestern Balkan, Eastern Germany, Poland-Northwestern Ukraine, and the Volga-Oka Valley projected onto the modern variation. The larger symbols denote the mean coordinates as well as 2 standard deviations along PC1 and PC2.*


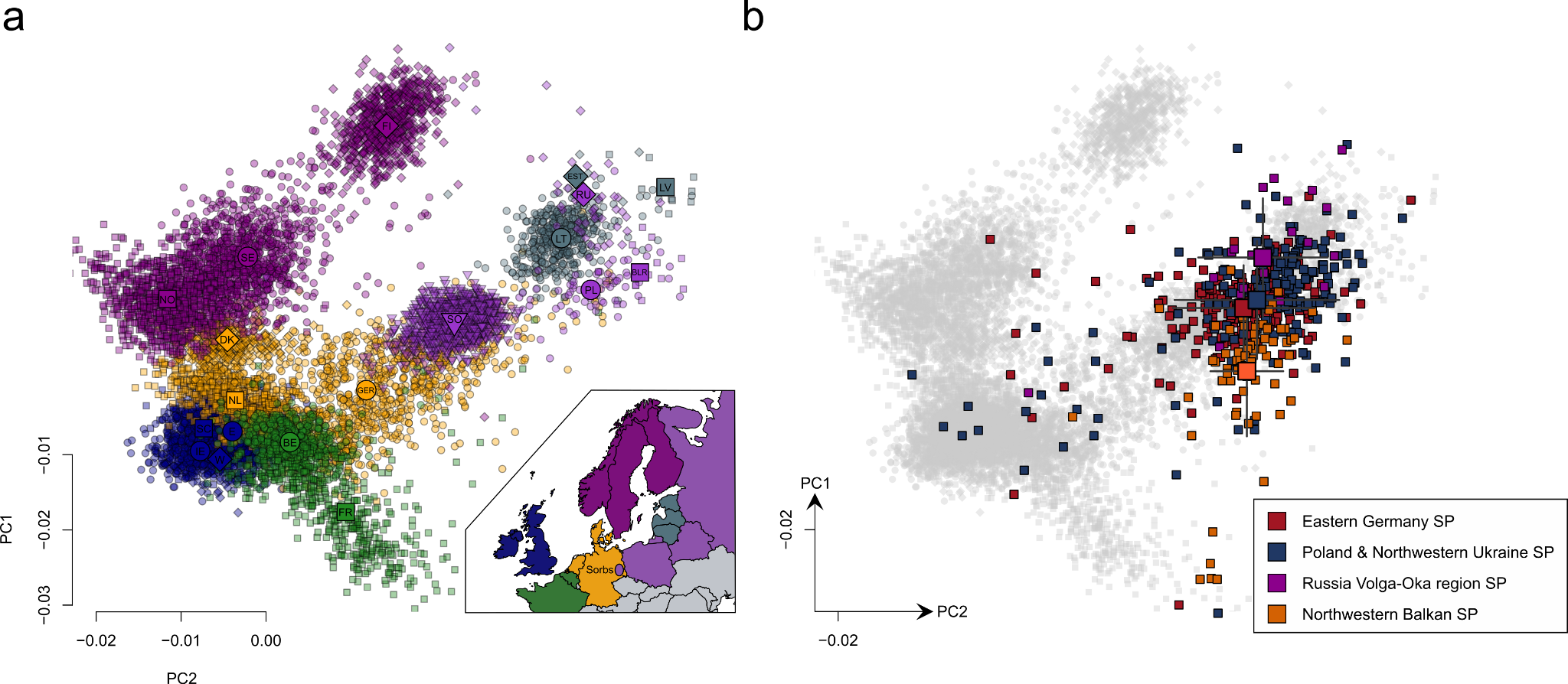


***Supp. Fig. 11. Population structure in central European SP individuals.*** *a) The reference PCA computed using 10,528 present-day Europeans. The large symbols indicate the mean PC1 and PC2 coordinates of the respective population. The map to the right visualizes the geographical origin of the genomes. b) SP genomes (n = 80, 240, 207, 15, resp.) from the Northwestern Balkan, Eastern Germany, Poland-Northwestern Ukraine, and the Volga-Oka Valley projected onto the modern variation. The larger symbols denote the mean coordinates as well as 2 standard deviations along PC1 and PC2.*


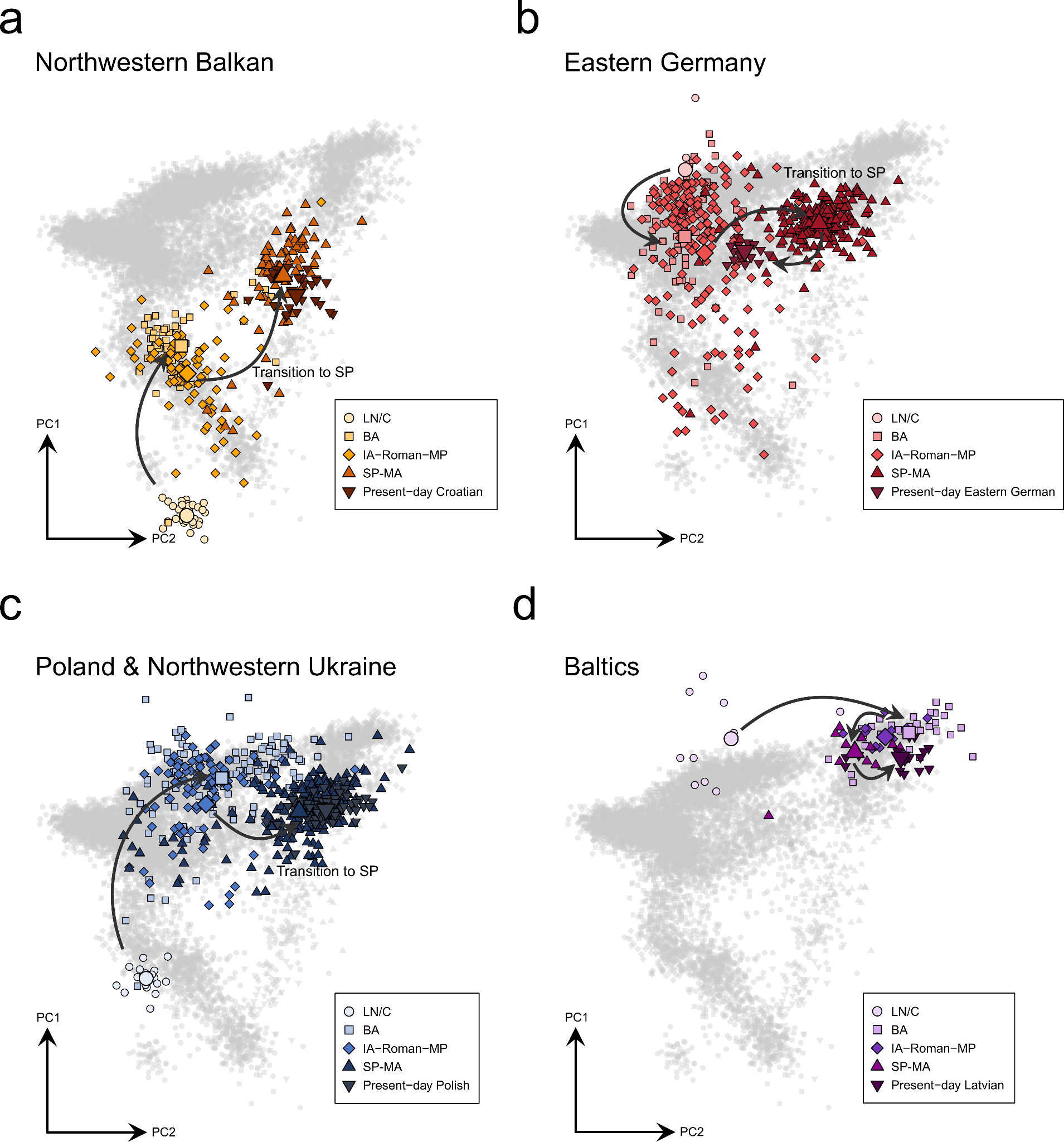


***Supp. Fig. 12. Population structure in four geographical transects throughout the last 5,000 years.*** *a) Projection of Late Neolithic/Chalcolithic (LN/C) (n = 32), Bronze Age (BA) (n = 63), Iron Age/Roman (IA-Roman-LA) (n = 80), early medieval /EMA) (n = 80) and present-day individuals (n = 28) from the Northwestern Balkan (coloured symbols). The large symbols indicate the mean PC1 and PC2 coordinates of the respective population. b) the same as a) but for individuals from Eastern Germany (n = 3, 61, 181, 240, 10, resp.). c) same as a) but for individuals from Poland and Northwestern Ukraine (n = 21, 132, 100, 207, 76, resp.). d) same as a) but for samples from the Baltics (Lithuania, Latvia, and Estonia) (n = 11, 30, 24, 27, 9, resp.).*

#### 4.1.2 unsupervised & supervised ADMIXTURE

We furthermore quantified the extent of the ancestry replacement and change in gene pool composition using ADMIXTURE[^121^](https://paperpile.com/c/UPmHk7/7NnVp). Initially, we decomposed the ancestry of 9,213 present-day individuals and 6,033 ancient genomes applying ADMIXTURE in unsupervised mode[^121^](https://paperpile.com/c/UPmHk7/7NnVp) (Fig. S13-14, Table S12-14).

Subsequently, we selected the present-day populations in which a component was maximized in the unsupervised approach at *K* = 9 to represent this cluster as source within a supervised ADMIXTURE setup as described in Gretzinger et al. 2022[^123^](https://paperpile.com/c/UPmHk7/tySmG) and Gretzinger et al. 2024[^131^](https://paperpile.com/c/UPmHk7/Tc5Kw) (Table S15, S16). Those components are:

- **WBI**_Western British Irish_ (maximized in Irish, northern Irish, Scottish, and Welsh; *n* = 667)
- **CNE**_Continental North European_ (maximized in Danes, northern Germans, and Dutch; *n* = 905)
- **NOR**_Norse_ (maximized in Swedes and Norwegians; *n* = 1910)
- **BAL**_Baltic_ (maximized in Russians, Belarussians, Latvians, Lithuanians, and Polish; *n* = 167)
- **FIN**_Finnish_ (maximised in Finns; *n* = 606)
- **WAS**_West Asian_ (represented by Greek, Cypriot, Turkish, Druze, and Adygei; *n* = 67)
- **CWE**_Continental Western European_ (maximized in Sardinians, Spanish and French; *n* = 812)
- **SAS**_South Asian_ (represented by Punjabi, Gujaratis, Brahui, and Balochi; *n* = 286)
- **EAS**_East Asian_ (represented by Han Chinese and Japanese; *n* = 207)

We further added the following three non-European sources in order to also detect Asian and African genetic variation:

- **NEA**_Middle Eastern_ (represented by Bedouins, Palestinians and Saudi; *n* = 121)
- **AFR**_African_ (represented by Esan, Mende, and Yoruba; *n* = 300)
- **NAS**_North Asian_ (represented by Yakut and Hezhen; *n* = 28)

Unsupervised ADMIXTURE shows that SP genomes from the three study transects, Eastern Germany, Croatia and Poland-Northwestern Ukraine, feature substantial fractions of ancestry maximized in present-day and ancient individuals from the Baltics (i.e. Lithuania, Latvia and Estonia) (Fig. S13-14). However, in contrast to ancient (and to a lesser extent present-day) individuals from the Baltics, the SP genomes from all three transects also exhibit considerable proportions of ancestry maximized in Southern and Western European as well as West Asian individuals. Thus, not all of their ancestry can be derived from Bronze and Iron Age Baltic individuals. Instead, admixture with non-Baltic-derived sources must have introduced this excess affinity to ancient and present-day Western and Southern Europeans. When comparing MP and SP genomes, a clear turnover is evident: IA and MP individuals from Eastern Germany and Poland-Northwestern Ukraine show predominantly Northwestern European-related components (maximized in ancient and present-day genomes from Britain and Ireland as well as Scandinavia and the North Sea area) but nearly no Baltic-derived ancestry (Fig. S13-14. Similarly, the Roman & MP genomes from the Northwestern Balkan feature mostly Southern European and West Asian ancestry components, yet only minor contributions from the Northeast (Fig. S13). Thus, the results from unsupervised ADMIXTURE agree with our observations from PCA, suggesting a major population turnover in Central and Southeastern Europe between the MP and SP.

We then used the unsupervised ADMIXTURE profiles in supervised modelling of target groups as mixtures from a pre-selected set of putative ancient source groups, using non-negative least squares implemented in the R package limSolve[^132^](https://paperpile.com/c/UPmHk7/w6do5). Our estimated ancestry proportions for SP target populations suggest that Baltic Bronze Age-related ancestry (here represented by Lithuania_BA) represents the dominant ancestry component in these individuals (>50%) (Fig. S15a). Interestingly, for all SP groups from Eastern Germany, Croatia, Poland, Ukraine and Russia, Lithuania_BA is preferred over Poland_MBA as the most proximal source group, agreeing with previous results demonstrating that SP individuals show excess affinity to Bronze Age Baltic individuals compared to Bronze Age Polish groups (although Poland_MBA and Baltic BA groups were genetically highly similar and closely related)[^133^](https://paperpile.com/c/UPmHk7/MbA4g). Besides this Baltic-related ancestry, all SP groups further show minor components of Scandinavian-related ancestry (here approximated by Sweden_IA), potentially due to long-term contact and admixture with East Germanic groups in Eastern Poland (who feature highest genetic similarity to ancient and present-day Scandinavians, especially Swedes[^134^](https://paperpile.com/c/UPmHk7/FJrFO)). Finally, all SP groups also carry varying fraction of Southeastern European and West Asian ancestry (approximated by Albania_BA_IA and Turkey_Hellenistic), indicating gene flow with groups located further south (most likely from the Balkan Peninsula), which is not detected in Baltic Bronze Age individuals[^133^](https://paperpile.com/c/UPmHk7/MbA4g). Using an ancient source group set, we show that this specific mixture, termed SP ancestry here (approximated by ancient individuals from Gródek, Poland), is still the dominant ancestry in Eastern and Eastern-Central Europe (i.e. Belarus, Poland, Ukraine, Slovakia, Hungary etc) as well as the Northwestern Balkan (Slovenia, Croatia & Serbia), decreasing towards the Aegean Sea (Fig. S15b).

In contrast, MP individuals feature depending on their geographical origin a diverse set of ancestries. In Eastern Germany, MP groups are predominantly modelled by Northern German IA-derived ancestry (represented by Roman individuals from Häven, Mecklenburg-Vorpommern). Yet, compared to later individuals from Lower Saxony, who similarly derive most of their ancestry from Häven, Mecklenburg-Vorpommern, the groups from Saxony-Anhalt feature substantially larger fractions of southern European and West Asian-related ancestry (Fig. S15a). The novel and previously published Roman Period individuals from Poland show predominantly Sweden_IA-derived ancestry, yet no Poland_MBA- or Baltic_BA-related ancestry, suggesting genetic discontinuity between the Bronze and Iron Age in (at least in some parts of) Poland. In Bubi’s Cave, Croatia, the ancestry of the Roman population is best approximated by a mixture of Southern European Iron Age sources without larger contributions of the Eastern Mediterranean, agreeing with our results obtained earlier. Finally, Iron Age samples from the Russian Volga-Oka valley carry more Baltic BA-, Russia_Bolshoy- and Finland_Levanluhta-related ancestry than the succeeding SP population (which shows on the other hands more ancestry from Southern and Northwestern Europe), supporting a major genetic turnover in all study regions after 600 CE.

This fundamental shift in genetic ancestry is not only visible on the regional level but can even be reconstructed for single sites. From Eastern Germany, at the site of Obermöllern (OBM), and from Eastern Poland, at the site of Gródek (GRK), we generated genomes dating to the earlier Roman/Migration Period as well as the later Slavic Period. For both sites, we demonstrate that the temporal divide is also reflected in the genetic diversity, with samples from the MP plotting on top of present-day Germanic-speaking Northwestern Europeans (Danish and Dutch in the case of OBM, Swedes and Norwegians in the case of GRK) and samples from the Slavic Period clustering with present-day Baltic- and Slavic-speaking populations from the Northeast, showing a significant divide in PC2 positions (Wilcoxon rank sum test; *W* = 21, *p* = 8.022e-11 for OBM and *W* = 4, *p* = 0.0001711 for GRK) (Fig. S16a,b).

To approximate geographically more confined admixture components that might better represent the locally-emerged gene pools of Europe during the later Bronze and Iron Age, we applied supervised ADMIXTURE as described above. Supervised ADMIXTURE modelling shows that this shift is characterized by a substantial decrease of Northwestern European ancestry (from 75% and 79% CNE+NOR ancestry to 8% and 21% in GRK and OBM, respectively) and a major increase of Northeastern European ancestry (from 14% and 4% BAL ancestry to 65% and 66% in GRK and OBM, respectively), indicative of large-scale population replacement. Yet, we also identify SP individuals resembling the MP population in both sites (Fig. S16c). Thus, we infer that this genetic change, while being fundamental, was not absolute, with remnants of the local populations being incorporated into the newcomers' society.

This is furthermore implied by the PCA position of the present-day populations inhabiting the study transects. For the Northwestern Balkan, we observe that present-day Croatians are shifted along PC1 to the South. This is consistent with a decrease of BAL ancestry (from 47% to 42%) and increase of Southern European CWE (from 25% to 36%) ancestry after the Early Middle Ages, suggesting later genetic influx from the local Balkan population (Fig. S12a).

In Eastern Germany, the present-day German-speaking population is shifted along PC2 in the direction of the MP genomes, mirroring the decrease of BAL ancestry (from 65% to 29%) and resurgence of CNE ancestry (from 17% to 38%) inferred using ADMIXTURE[^121^](https://paperpile.com/c/UPmHk7/7NnVp). In contrast, we do not observe this resurgence in the Slavic-speaking Sorbian population which retains CNE and BAL proportions more similar to the medieval Slavic population (18% and 54%, respectively) (Fig. S12b).

Similarly, no major decrease of BAL ancestry was detected in Poland (from 63% to 55%) agreeing with the substantial overlap of Slavic Period and present-day Polish genomes in PCA space. Thus, the incorporation of the MP population (or later influx from genetically similar sources) was seemingly less pronounced in Poland than in neighboring Eastern Germany, cosientent with the preservation of Slavic languages in the region (Fig. S12c).

Interestingly, in all of our three study transects we also measure temporary surges of BAL ancestry predating the Slavic Period, specifically in ancient individuals from the Middle and Late Bronze Age, corresponding to increased affinity to Northeastern European populations and higher levels of Western Hunter-Gatherer ancestry (WHG). These include (but may not be limited to) individuals associated with the Middle Bronze Age (MBA) Jagodnjak culture in Croatia[^43^](https://paperpile.com/c/e5c4dc/hakvl), the MBA Trzciniec Culture in Poland[^44^](https://paperpile.com/c/e5c4dc/NCKSG), and the Late Bronze Age Tollense battlefield in Northeastern Germany[^45^](https://paperpile.com/c/e5c4dc/XJ0KF). These surges seem to be mostly short-lived, as the Iron Age and Roman population of all three study regions is depleted of BAL ancestry, accounting for 6% ± 2%, 5% ± 1%, and 7% ± 2% of the total ancestry in the Northwestern Balkans, Eastern Germany, and Poland-Northwestern Ukraine, respectively.

*
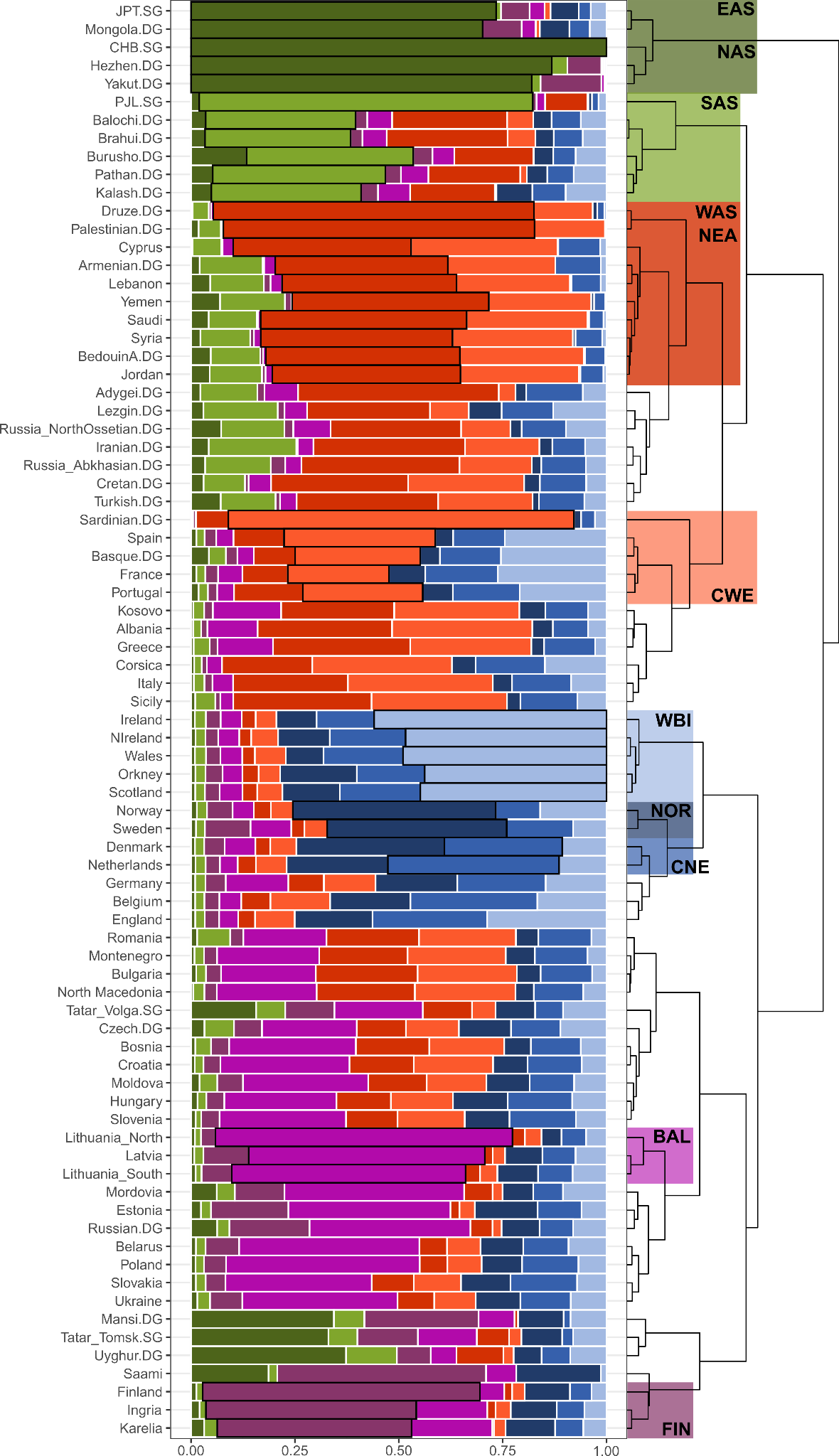
*

***Supp. Fig. 13. Averaged results from unsupervised ADMIXTURE analysis.*** *Relevant present-day individuals from Europe and Asia (n = 10142) were decomposed into 9 ancestral sources using an unsupervised clustering approach. Columns were ordered according to hierarchical cluster analysis applying Ward’s minimum variance method. The selection of present-day populations as ancestry proxies in supervised ADMIXTURE analysis as well as the defining ancestry components are highlighted.*

*
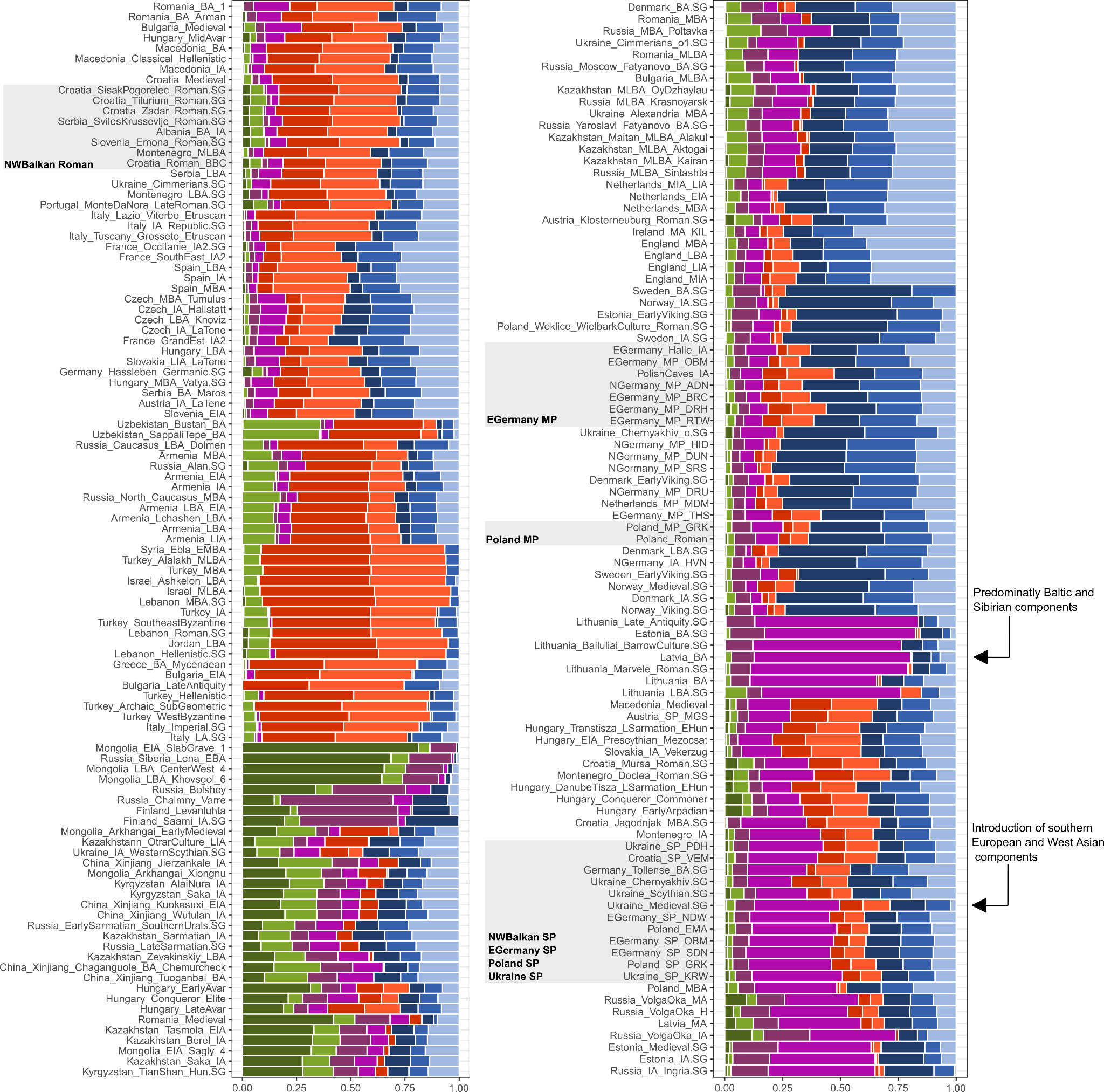
*

***Supp. Fig. 14. Averaged results from unsupervised ADMIXTURE analysis.*** *Relevant ancient individuals from Europe and Asia (n = 3090) were decomposed into 9 ancestral sources using an unsupervised clustering approach. Columns were ordered according to hierarchical cluster analysis applying Ward’s minimum variance method. The colors correspond to Supp. Fig. 11.*

*
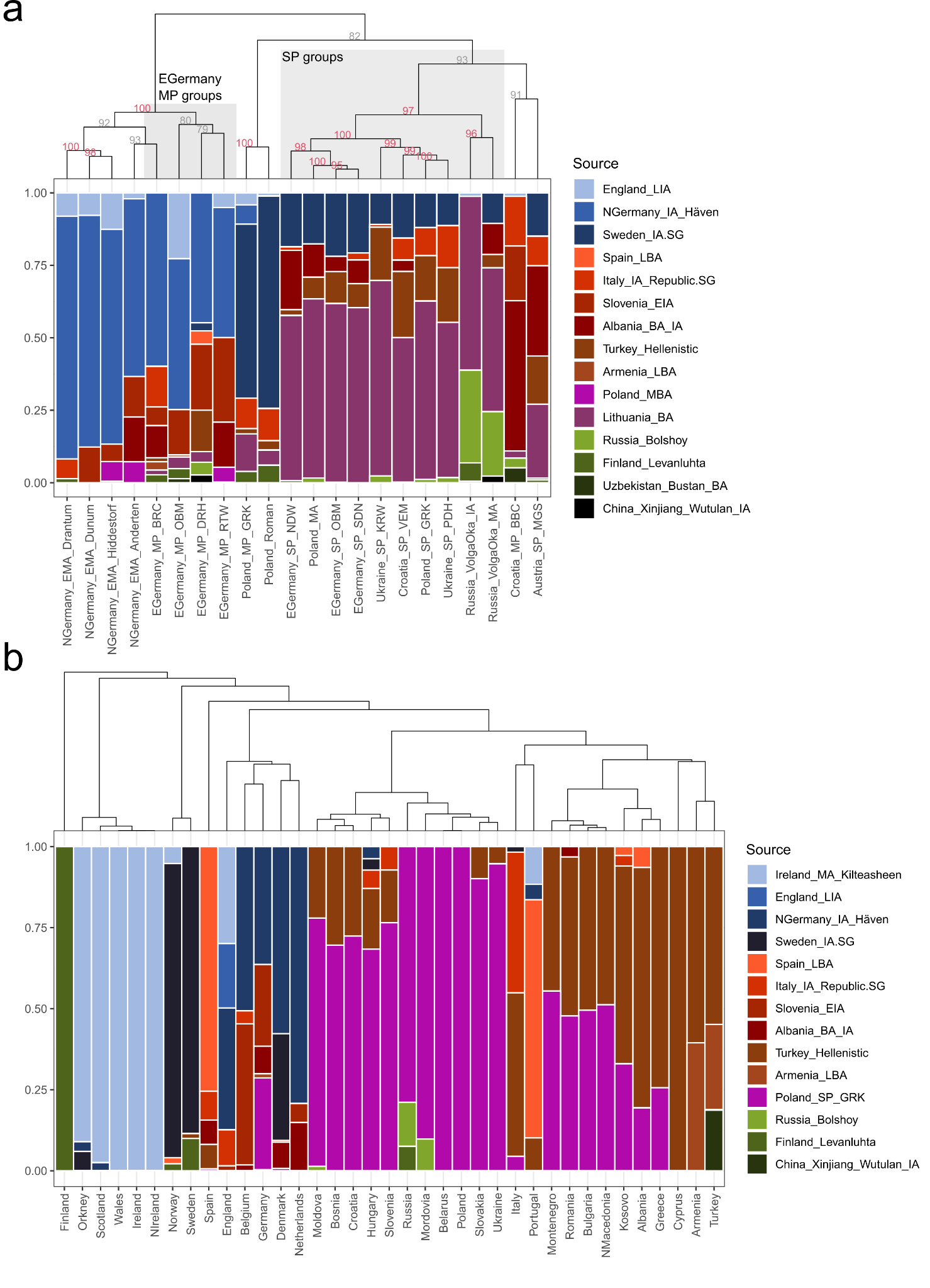
*

***Supp. Fig. 15. NNLS modelling using the Q matrix from unsupervised ADMIXTURE at K = 9.*** *a) Group-based results from NNLS admixture modeling for 22 selected ancient populations. b) Group-based results from NNLS admixture modeling for 37 selected present-day populations.*


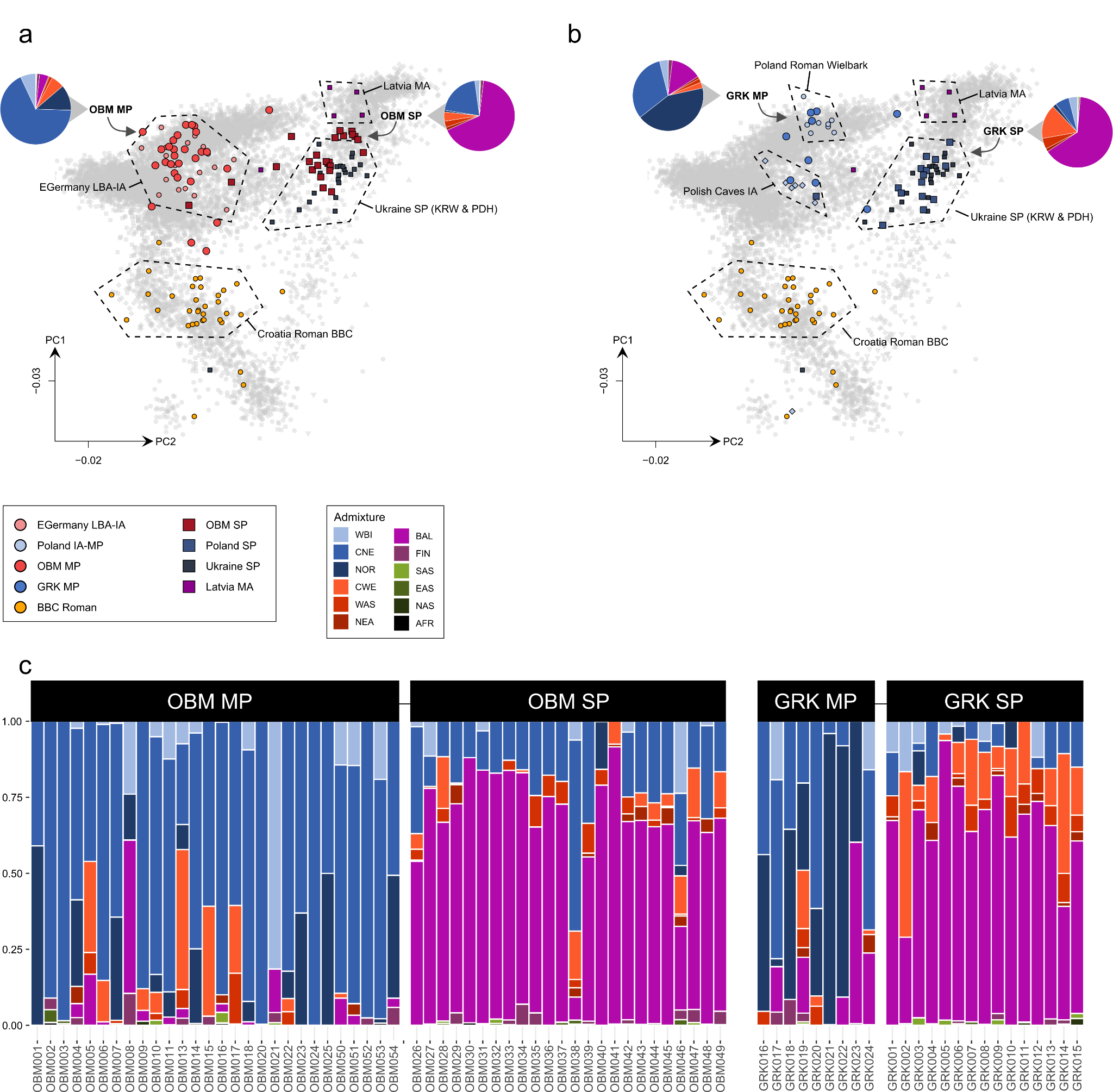


***Supp. Fig. 16. Population structure in two sites before and after the arrival of SP.*** *a) MP and SP individuals from Obermöllern (OBM) (n = 52), Eastern Germany, projected together with novel and previously published ancient genomes onto the modern variation. Averaged ancestry estimates from supervised ADMIXTURE analyses are shown as pie charts for the OBM MP and SP groups. b) MP and SP individuals from Gródek (GRK) (n = 24), East Poland, projected together with novel and previously published ancient genomes onto the modern variation*. *Averaged ancestry estimates from supervised ADMIXTURE analyses are shown as pie charts for the GRK MP and SP groups.*

### *4.2 F*-statistics

#### 4.2.1 F_4_ statistics

To formally test whether these patterns observed from PCA and ADMIXTURE[^121^](https://paperpile.com/c/UPmHk7/7NnVp) are consistent with gene-flow events from the East into our three study transects, we used *F*-statistics to quantify genetic affinities of SP individuals to preceding MP and succeeding present-day groups. We first compared the proportions of shared genetic drift with present-day populations using F_4_ statistics of the form F_4_(YRI, Test; MP, SP) on the group level (Table S22). In general, present-day populations from Western, Northwestern and Southwestern Europe are more closely related to MP than to SP genomes from all three study transects (Fig. S17). The only deviation from this pattern is observed for ancient samples from the Russian Volga-Oka region, where we measure higher genetic affinity between present-day Southern/Western Europeans and the SP population compared to the pre-SP population (Fig. S17). This agrees with the pattern observed in PCA and ADMIXTURE that, in contrast to the Northwestern Balkan, Eastern Germany, and Poland-Northwestern Ukraine, the arrival of Slavic-associated culture in Northwestern Russia was associated with a shift in PCA space to the West, a decrease of BAL ancestry, and the introduction of Western European ancestries such as CNE and CWE.

On the other hand, most present-day populations from East-Central and Eastern Europe (e.g. Polish, Ukrainians, Slovaks, Lithuanians and Latvians etc.) are significantly closer related to the SP than to the MP population (Fig. S17). Ancient genomes from the Volga-Oka region are again an exception, as we observe equal affinities of present-day Eastern Europeans to both the IA and SP populations.

We then proceeded to investigate these affinities on the individual level. We calculated F_4_ statistics of the form F_4_(CHB, TestA; Ireland, TestB) for each MP and SP individual (TestA) measuring its affinity to diverse present-day European populations (TestB) (compared to present-day Irish) (Fig. S18-19, Table S20). We chose Ireland here as outgroup as we do not expect any excess affinity to British-Irish genomes in our continental samples). We observe the following patterns:

- MP genomes from the Northwestern Balkan show the highest affinity to present-day Southern Europeans, especially Italians and Spanish. They are symmetrically related to Irish and present-day groups from the Balkans, East-Central Europe, and Northeastern Europe/the Baltics. The medieval SP genomes on the other hand show the highest affinity to present-day Latvians and Lithuanians, followed by other Eastern and East-Central European populations. They are also closer related to Irish than to Southern Europeans (such as Italians and Spanish), evidencing a general increase of Northern European affinity (Fig. S18a).
- MP genomes from Eastern Germany show the highest affinity to present-day Scandinavians, specifically Danes. Furthermore, they are closer related to Irish than to Southern and Northeastern European populations. The SP genomes are closest related to Baltic groups (Latvians, Lithuanians, Estonians), followed by East-Central and Eastern European populations such as Poles and Belarussians (Fig. S18b).
- Similar to the MP genomes from Eastern Germany, the MP genomes from Poland show the highest affinity to present-day Scandianvians, especially Swedes. They are furthermore closer related to Irish than to present-day groups from East-Central Europe and the Balkan. The SP genomes show, similar to SP individuals from Eastern Germany and Croatia, the highest affinity to Latvians, Lithuanians, Estonians, Poles, and Belarussians (Fig. S18c).

Additionally, we replicated this pattern in a group-based analysis, investigating genetic affinities from the Late Neolithic to the present-day using *F*-statistics of the form F_4_(CHB, TestA; Ireland, TestB) for six sub-regions in Central and East-Central Europe (Fig. S20). As described before, we detect a significant increase in affinity to Baltic populations in many Central European populations during the Early Middle Ages that either persists until today (e.g. in Croatia, Czech Republic, Poland or Ukraine) or subsequently declines (in Eastern Germany).


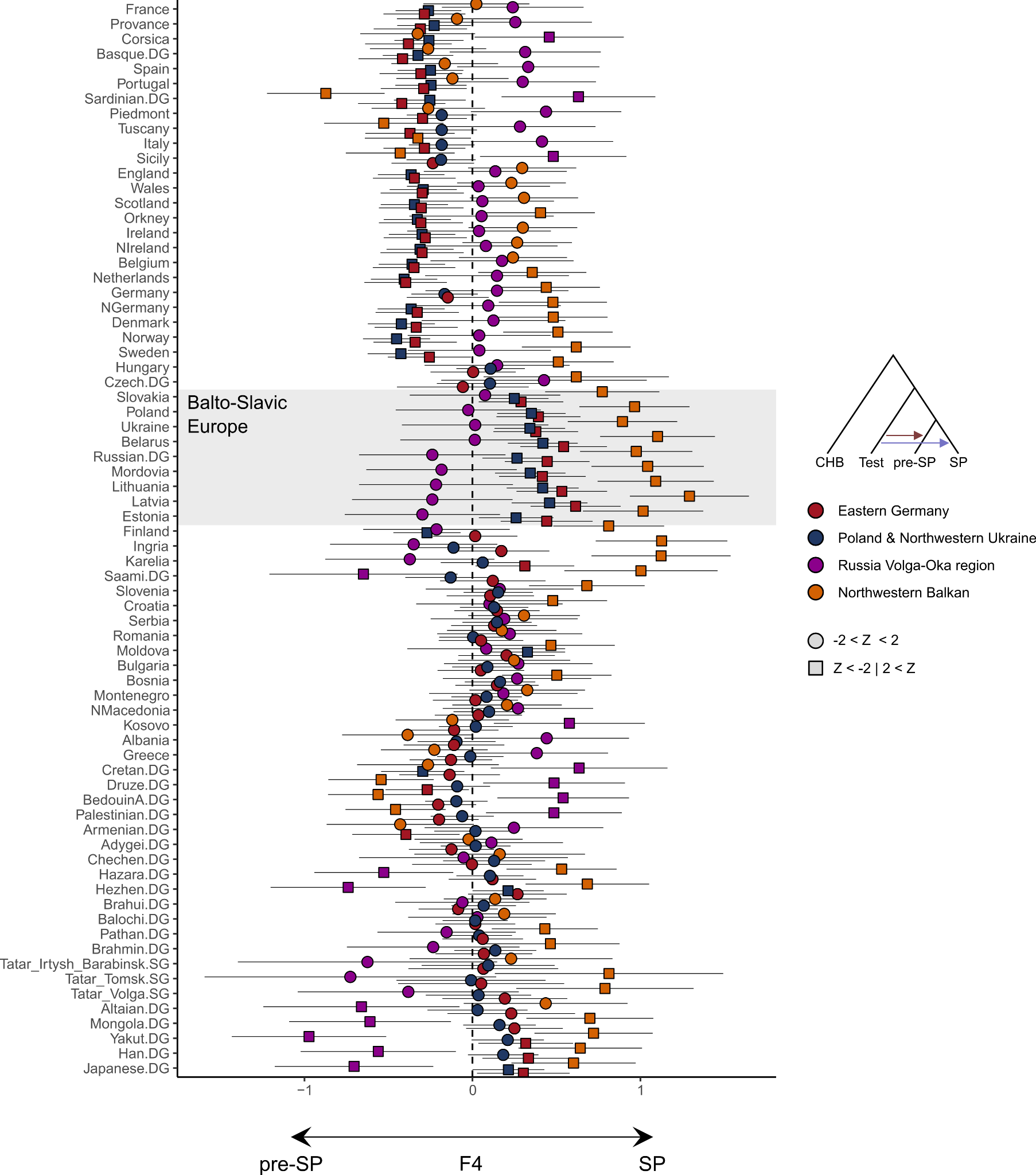


***Supp. Fig. 17. Genetic affinities of pre-SP (Roman and MP) and SP individuals from the Northwestern Balkan, Eastern Germany, Poland-Northwestern Ukraine and the Russian Volga-Oka region****. Shown are the results of the F_4_-statistic of the form F_4_(CHB, Test; pre-SP genomes, SP genomes) for 72 relevant present-day populations from Europe and Asia in comparison to pre-SP and SP genomes from the four study transects Northwestern Balkan, Eastern Germany, Poland-Northwestern Ukraine and Volga-Oka region (n = 67, 181, 100, 9 and n = 80, 240, 207, 15, resp.). Error bars indicate two standard errors.*

***
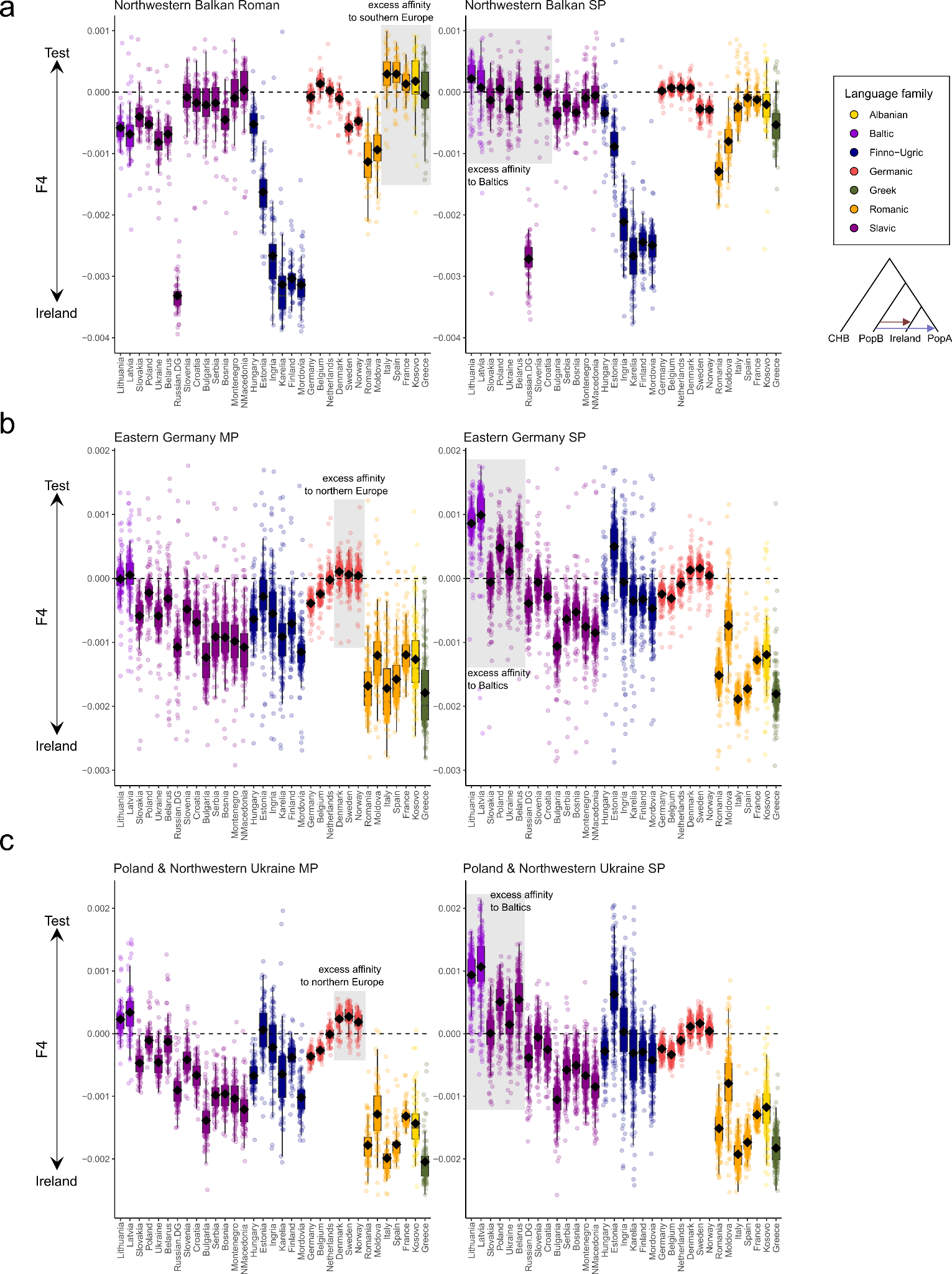
***

***Supp. Fig. 18. Genetic affinities of pre-SP and SP individuals from the Northwestern Balkans, Eastern Germany, and Poland-Northwestern Ukraine.*** *Shown are individual results of the F_4_-statistic of the form F_4_(CHB, PopB; Ireland, PopA) as boxplots, where PopB iterates through all MP (shown on the left) (n = 67, 181, 100) and SP (shown on the right) (n = 80, 240, 207) genomes from the three study transects and PopA iterates through 33 relevant present-day European populations. Bounds of the Box represent the 25^th^ and 75^th^ Percentile. The center represents the median. Whiskers represent the smallest value greater than the 25^th^ Percentile minus 1.5 times the interquartile range and largest value less than the 75^th^ Percentile plus 1.5 times the interquartile range, respectively. Outliers present the minimum and maximum values in the data.*


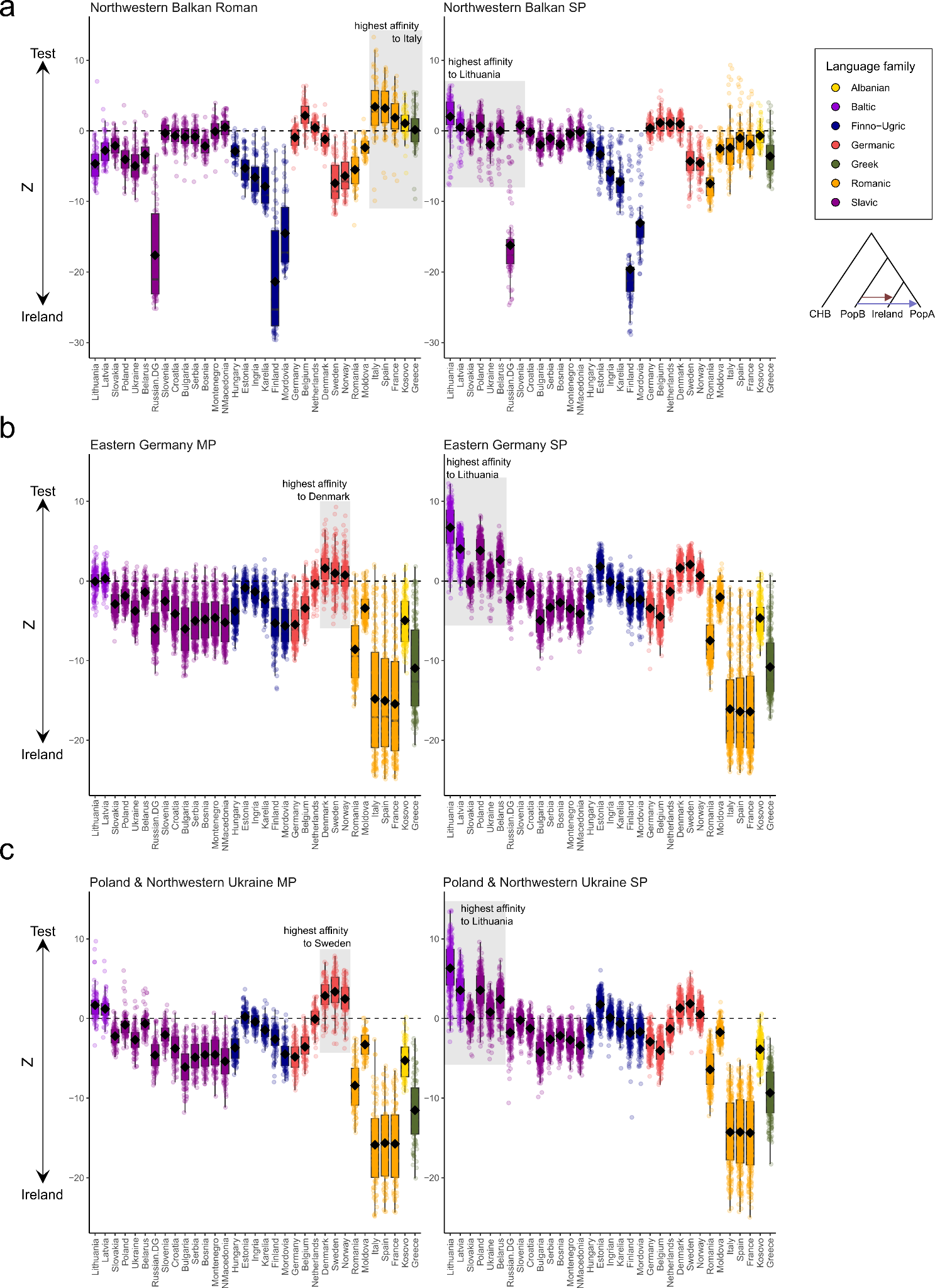


***Supp. Fig. 19. Genetic affinities of pre-SP and SP individuals from the Northwest Balkans, Eastern Germany, and Poland-North Ukraine.*** *Shown are individual Z values of the respective F_4_-statistics presented in Supp. Fig. 16 as boxplots. Bounds of the Box represent the 25^th^ and 75^th^ Percentile. The center represents the median. Whiskers represent the smallest value greater than the 25^th^ Percentile minus 1.5 times the interquartile range and largest value less than the 75^th^ Percentile plus 1.5 times the interquartile range, respectively. Outliers present the minimum and maximum values in the data.*


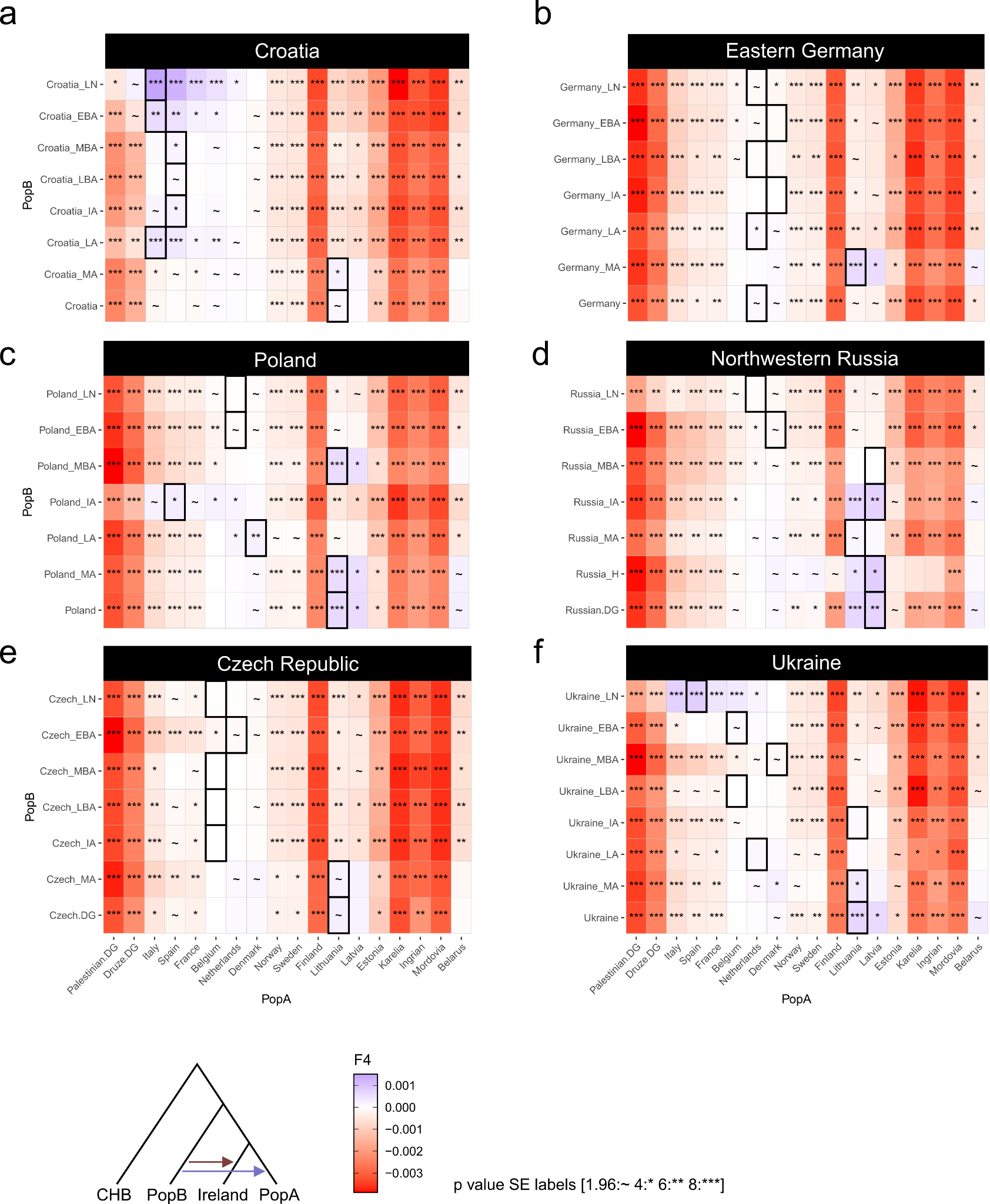


***Supp. Fig. 20. Changes in genetic affinity in Central Europe throughout the last 5,000 years.*** *a) Group-based F_4_-statistics of the form F_4_(CHB, PopB; Ireland, PopA). Negative values indicate that the test population is closer to Ireland than to PopB; positive values indicate that the test population is closer to PopB than to Ireland. PopB iterates through ancient and present-day populations from Croatia (n = 39, 4, 47, 26, 22, 67, 80, 28, resp.). b) same as a) but for populations from Eastern Germany (n = 88, 8, 1, 19, 184, 240, 1016). c) For populations from Poland-Northwestern (n = 39, 31, 72, 9, 100, 207, 76). d) For populations from Northwestern Russia (n = 26, 50, 32, 30, 62, 9, 71, resp.) For populations from the Czech Republic (n = 110, 258, 5, 35, 75, 4, 1, 1, resp.). f) For populations from Central Ukraine (n = 24, 10, 2, 3, 15, 3, 4, 27, resp.).*

#### 4.2.2 F_ST_

The divergence between pre-SP (Roman/MP) and SP groups is also verified in the distribution of genetic distances (F_ST_) (Table S21):

- Roman/MP genomes from the Northwestern Balkan exhibit lowest genetic differentiation from present-day Southern Europeans, especially Italians, Bulgarians, and Greeks. Furthermore, they show considerable affinity to other Balkan populations such North Macedonians, Serbians and Bosnians, as well as groups from the Iberian Peninsula. The medieval SP genomes exhibit the lowest genetic differentiation from East-Central European populations (e.g. Slovaks, Hungarians, Ukrainians, and Polish) as well as groups from the Balkan Peninsula (e.g. Slovenians, Bulgarians, Serbians and Bosnians) (Fig. S21a).
- MP genomes from Eastern Germany exhibit lowest genetic differentiation from Northwestern Europeans, especially present-day Germans, Belgians (from Flanders), Dutch, Danish, and English. In contrast, the medieval SP genomes show the highest affinity to East-Central and Eastern Europeans, e.g. Slovaks, Ukrainians, and Poles but also considerable affinity to groups from the Balkans, Baltics and Finno-Ugric-speaking populations in Europe (e.g. Hungarians and Estonians) (Fig. S21b).
- Roman/MP genomes from Poland-Northwestern Ukraine exhibit lowest genetic differentiation from Northwestern Europeans, especially Scandinavians. The medieval SP genomes show affinities nearly identical to the SP genomes from Eastern Germany, featuring highest genetic similarity to Ukrainians, Polish, and Slovaks, followed by Slovenians, Bosnians, Hungarians, and Baltic populations (Fig. S21c).

In summary, we observe that SP genomes from all three transects feature highly similar distributions of F_ST_ distances to present-day West Eurasian groups (Pearson's product-moment correlation for Eastern Germany SP and Poland-Northwestern Ukraine SP: *t* = 77.506, *df* = 47, *p* < 2.2e-16 and *r* = 0.996; for Poland-Northwestern Ukraine SP and Northwestern Balkan SP: *t* = 8.7308, *df* = 47, *p* = 2.115e-11 and *r* = 0.787; for Northwestern Balkan SP and Eastern Germany SP: *t* = 8.2954, *df* = 47, *p* = 9.263e-11 and *r* =0.771).

We find the highest genetic affinity between SP genomes from Eastern Germany, Poland-Northwestern Ukraine as well as the Northwestern Balkan and present-day populations of Eastern and East-Central Europe, especially Slavic-speaking groups from Poland, Slovakia, Ukraine, Belarus and Western Russia (Table S23). From this area, F_ST_ distances increase towards the West and East. We observe the highest F_ST_ distances (i.e. lowest genetic affinities) in Southern Europe (namely the Iberian and Italian peninsulas), with a gradual increase to the Southeast (across the Balkan Peninsula) and the East (across Western Russia) (Fig. S22a). This pattern generally mirrors the distribution of BAL ancestry in Western Eurasia, with the highest proportions in Northeastern Europe and decreasing fractions in the Southeast and East (Fig. S22b).

The high F_ST_ affinities of present-day populations from the Balkan with Northwestern Balkan SP genomes can be explained by the excess Southern European admixture that is evident in SP genomes from Croatia but absent from SP genomes in Eastern Germany and Poland-Northwestern Ukraine (based on PCA, F_4_, ADMIXTURE and qpAdm analysis).


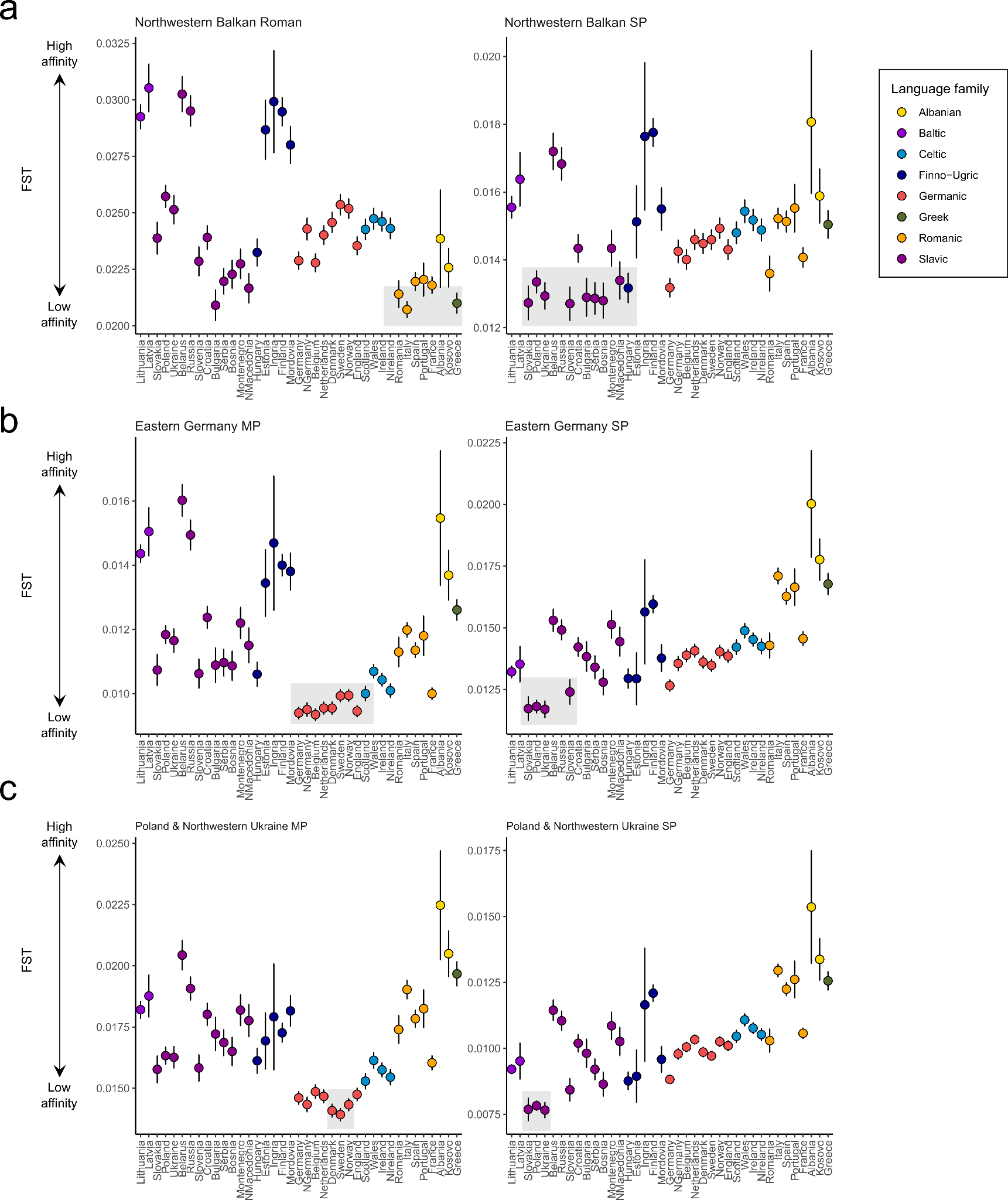


***Supp. Fig. 21. Population genetic affinities of MP and SP individuals from Central Europe.*** *a) Group-based F_ST_ estimates between pre-SP genomes (Roman & MP, n = 67) (shown on the left) or SP genomes (n = 80) (shown on the right) from the Northwestern Balkans and 39 relevant present-day populations from Europe. Present-day populations are colored based on their linguistic classification. Error bars indicate two standard errors. b) Same as a) but for MP (n = 181) and SP (n = 240) genomes from Eastern Germany. c) For MP (n = 100) and SP (n = 207) genomes from Poland-Northwestern Ukraine.*


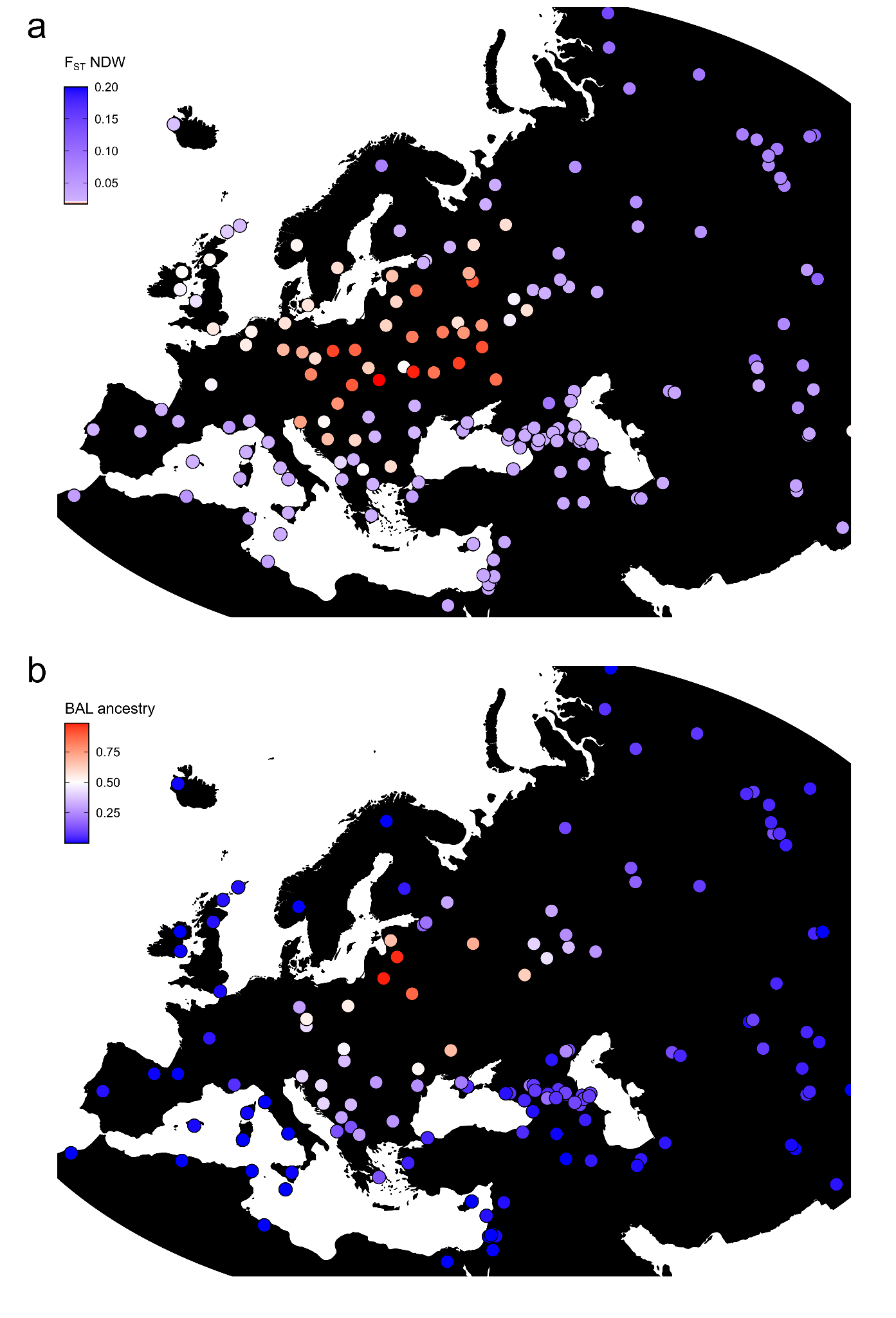


***Supp. Fig. 22. Genetic affinity to Northeast European ancestry in present-day West Eurasia.*** *a) F_ST_ point estimates between the Niederwünsch individuals from Eastern Germany (n = 182) and 215 present-day European, Asian, and North African populations. b) Average proportions of Northeastern European ancestry (BAL) in present-day individuals (n = 2826) from 194 groups in Europe, Asia, and North Africa populations, as measured using a supervised clustering approach implemented in ADMIXTURE.*

### 4.3 IBD sharing

#### 4.3.1 Present-day comparisons

Besides conventional unlinked SNP approaches, we also applied Haplotype-based methods, which have been shown to outperform Allele frequency-based methods in the detection of population substructure. We conducted Identity-By-Descent (IBD) analysis on imputed diploid genomes to reveal fine-scale affinities between the ancient genomes and present-day populations (as described in Methods).

We calculated the sum of IBD (sIBD) shared between each pair of individuals (ancient and present-day) and aggregated the mean sharing statistics across 263 Eurasian populations. Similar to the affinities detected in PCA and F_ST_ analysis, we observe the highest number of shared IBD segments between SP genomes and present-day Baltic- and Slavic-speaking Northeastern and Eastern European populations (Fig. S23b, Ex. Fig. 5). The SP genomes share the highest number of IBD segments with populations from the Baltics, namely Latvians and Lithuanians as well as Belarussians, followed by Poles and Sorbs in the West and Ukrainians from Sumskaya and Russians from Orel to the East (Ex. Fig. 5). This pattern may indicate an initial expansion of the SP gene pool from the Baltics to the South, followed by a split into a Western (through Poland into Eastern Germany) and an (North-)Eastern dispersal (through Sumskaya and Orel into Kaluga, Yaroslavl and Pskov) (Fig. S23a).

Less IBD sharing is seen with Slavic-speaking populations from the Balkan Peninsula, where the highest IBD sharing is observed in the Northwest (in Slovenia and Croatia) and lowest in the South (in Greeks, Albanians and Kosovars), reflecting the increasing fractions of Southern European ancestry and decreasing proportions of BAL/SP admixture.

Investigating the correlation between IBD-sharing and language, we find that the SP genomes share the largest fraction of IBD with Baltic-speaking populations (Latvians and Lithuanians), followed by North/(East-West)-Slavic speaking groups (Russians, Poles, Ukrainians, Sorbs). Slightly lower numbers were found in Finno-Ugric-speaking groups, where (as predicted by spatial proximity) populations from Europe (e.g. Hungarians, Estonians, Mordovians, Ingrians and Karelians) share more IBD with the SP genomes than populations from further East, in Siberia and the Ural mountain area (e.g. Mansi and Udmurt) (Fig. S24a). South Slavic-speaking groups appear more homogenous in their IBD sharing, with more northerly populations (Slovenians, Croatians and Serbians) sharing slightly more IBD with the SP genomes than more southerly groups (North Macedonians, Montenegrians and Bulgarians) (Fig. S24a).

Furthermore, we observe a general pattern of higher IBD sharing of the SP individuals with Northern European Celtic- and Germanic-speaking groups than with Southern European Italic- or Greek-speaking populations. We also detect several outlier groups that share more IBD with the SP genomes than what would be expected based on the linguistic affiliation (Fig. S24b). These include Eastern Germans (German-speaking), Romanians and Moldovans (Romance-speaking) as well as Chuvash, Gagauz and Tatars (Turkic-speaking) from East-Central Europe (Fig. S24b). For these groups, the close geographic proximity to Slavic-speaking populations appears to be a better predictor of their IBD sharing than their language affiliation.

These patterns of IBD-sharing with present-day groups are almost identical for all SP groups analyzed in this study. We measure correlation coefficients *r* > 0.97 for all pairs including SP genomes from Eastern Germany, Poland-Northwestern Ukraine, and the Northwestern Balkan (Fig. S25, Table S40). Only for genomes from the Russian Volga-Oka region we observe lower coefficients (0.83 - 0.88) due to the high genetic heterogeneity of this population and excess admixture with local Iron Age groups. Overall, sharing of segments identical-by-descent with present-day populations clearly shows high genetic similarity of all SP individuals across Central Europe (with minor introgression from the diverse local predecessors) and a common geographic origin in Eastern Europe, probably in close proximity to the Baltics.


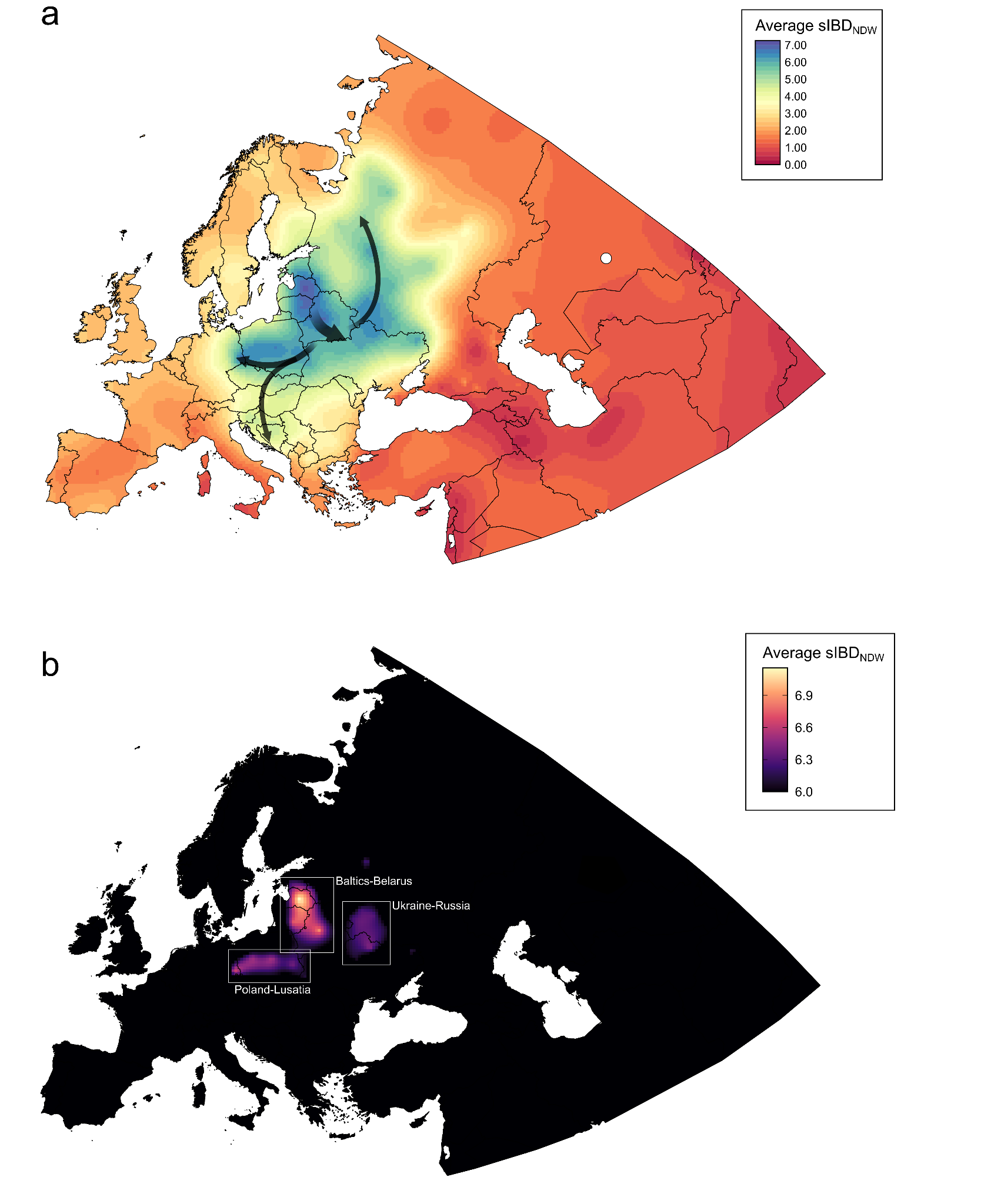


***Supp. Fig. 23. Genetic relatedness between present-day populations and ancient SP groups.*** *a) Kriging interpolation of the normalized, average IBD sharing between the NDW population from Eastern Germany and present-day 215 European, Asian, and North African populations. Arrows on map indicate a general direction of influences rather than discrete routes of migration. b) The three geographic regions with the highest interpolated normalized, average IBD are highlighted.*


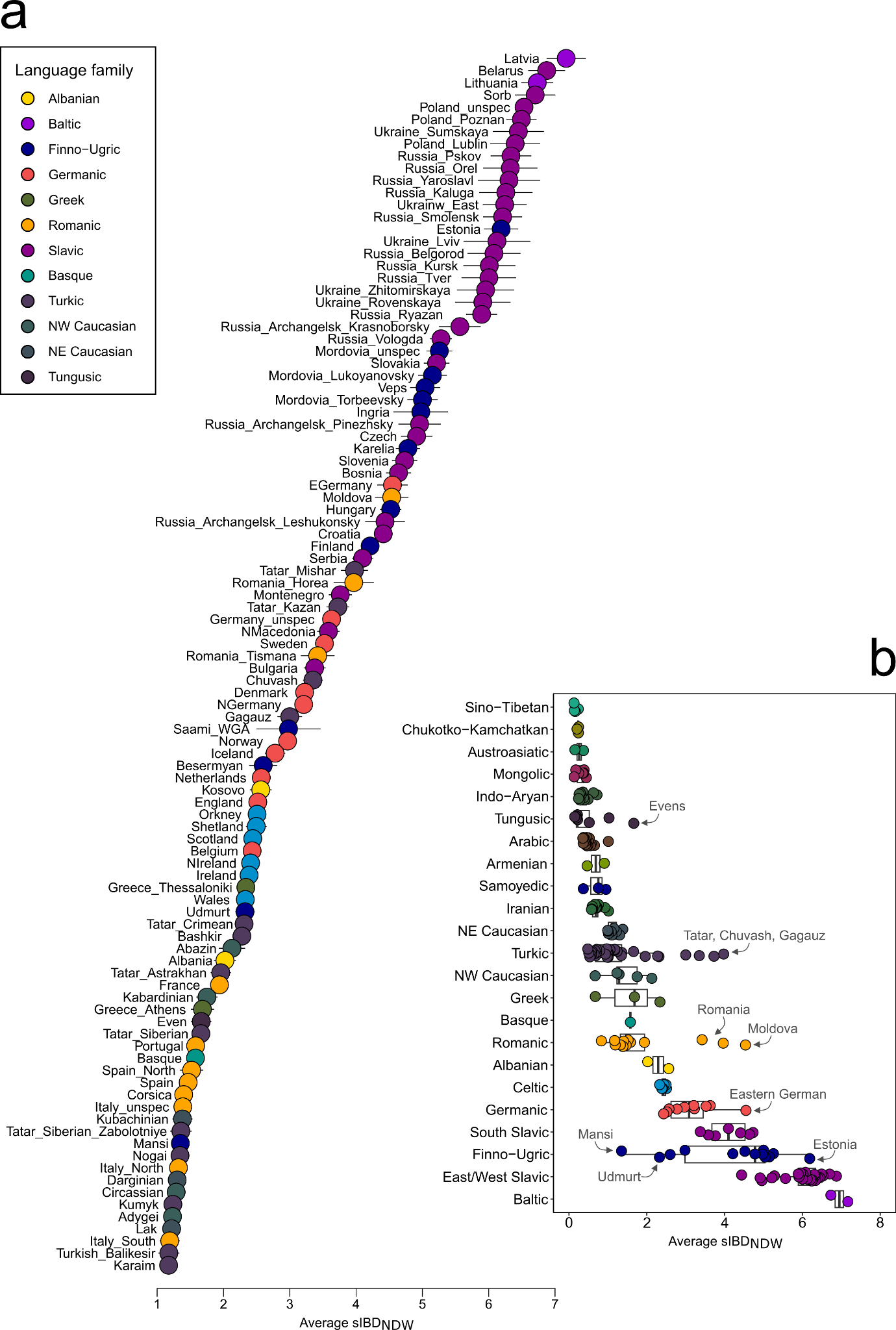


***Supp. Fig. 24. Genetic relatedness between present-day populations and SP groups.*** *a) Normalized, average IBD sharing between the NDW population from Eastern Germany and 200 present-day groups in Europe and Asia (only the top 100 groups with highest sIBD are shown). Present-day populations are colored based on their linguistic classification. Error bars indicate two standard errors. b) The same data as in panel a) but shown as boxplots for each major language family (and all 200 groups). Bounds of the Box represent the 25^th^ and 75^th^ Percentile. The centre represents the median. Whiskers represent the smallest value greater than the 25^th^ Percentile minus 1.5 times the interquartile range and largest value less than the 75^th^ Percentile plus 1.5 times the interquartile range, respectively. Outliers present the minimum and maximum values in the data.*


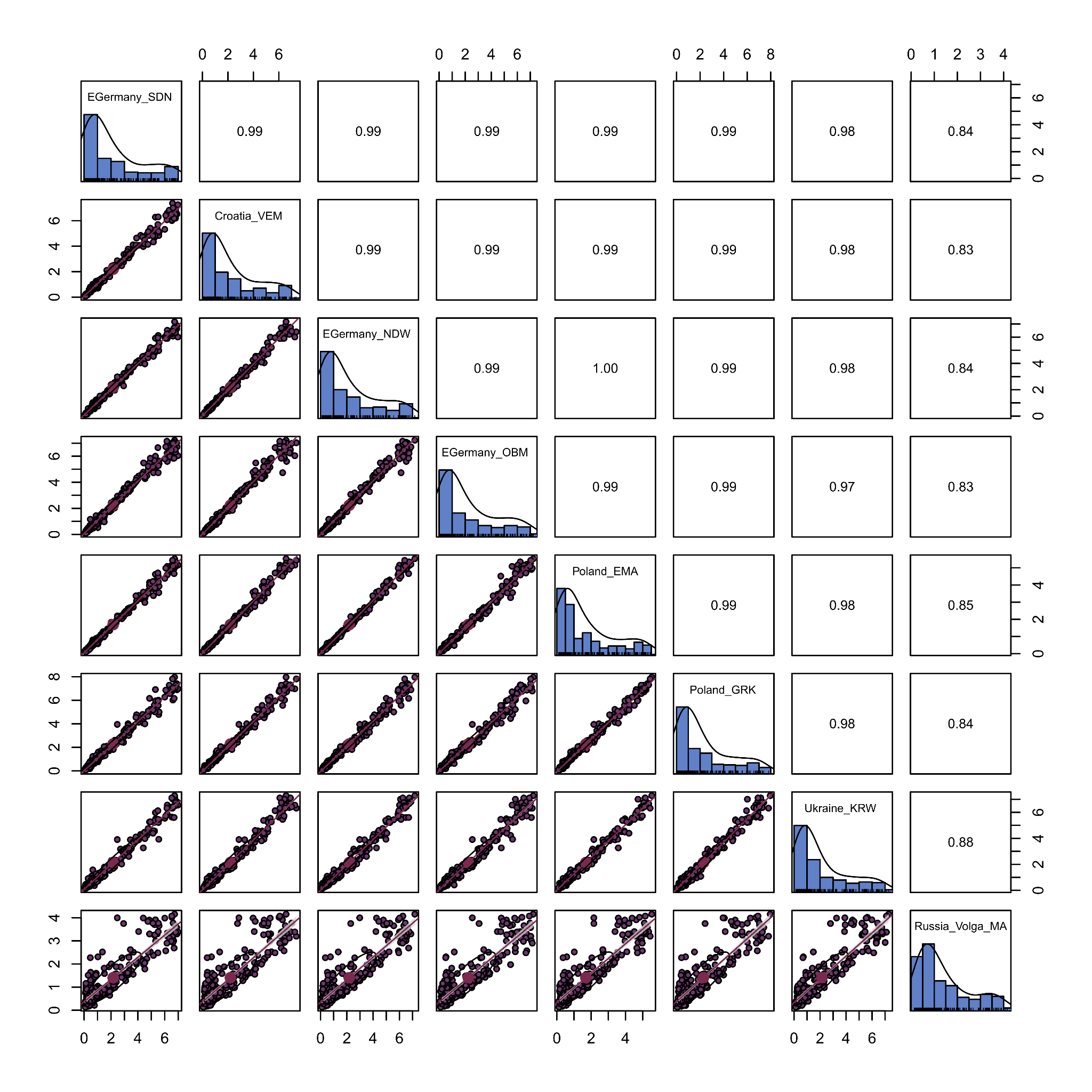


***Supp. Fig. 25. Similarity in IBD sharing across SP populations from Central Europe.*** *Correlogram illustrating pairwise correlations in sIBD sharing between 215 present-day Eurasian populations and 8 SP populations in Croatia (VEM), Eastern Germany (NDW, SDN, OBM) Poland (Poland_MA, GRK), Ukraine (KRW, PDH), and Russia (Russia_VolgaOka_MA). Pearson’s r for each pairwise correlation test are indicated in the upper triangle of the matrix. Error bands represent two standard errors.*

#### 4.3.2 Ancient DNA comparisons

To obtain a finer-scale characterization of recent and more distant genetic relatedness across space in the ancient DNA record, we followed an approach recently introduced by Allentoft and colleagues[^132,135^](https://paperpile.com/c/UPmHk7/w6do5+WDKi8). We performed genetic clustering of the ancient individuals using hierarchical community detection on a network of pairwise IBD-sharing similarities. In detail, we constructed a weighted network from pairwise IBD-sharing similarities between 2,657 ancient, post-Neolithic Eurasian individuals using the igraph package in R, with the fraction of the genome shared IBD between pairs of individuals as weights. For that, we calculated for all pairs the shared sum of IBD larger than 9 cM (estimated with ancIBD[^136^](https://paperpile.com/c/UPmHk7/k2aNe)). We excluded one individual from any pair sharing more than 150 cM[^137^](https://paperpile.com/c/UPmHk7/Ki9ha) (which are assumed to be more reflective of familial relationships) to avoid the formation of small clusters with only close relatives. Specifically, we preferentially excluded individuals featured in multiple pairs to minimize the total number of excluded individuals.

We then performed iterative community detection on this network using the Leiden algorithm[^138^](https://paperpile.com/c/UPmHk7/9f3c1), applying a resolution parameter of *r* = 0.5 as the starting value for each level of community detection[^132^](https://paperpile.com/c/UPmHk7/w6do5). If more than one community was detected, we split the network into the respective communities, and repeated the community detection step. If no communities were detected, we incremented the resolution parameter in steps of 0.5 until a maximum value of *r* = 3[^132^](https://paperpile.com/c/UPmHk7/w6do5). The initial clustering was completed when no more communities were detected at the highest resolution parameter, across all subcommunities. To convert the resulting hierarchy into a final clustering, we simplified the initial clustering by collapsing nodes into single clusters on the basis of observed spatiotemporal annotations of the samples (Table S38). As mentioned in Allentoft et al. 2024, we highlight that the obtained clusters should not be interpreted as “populations” in the sense of a local community of individuals[^132^](https://paperpile.com/c/UPmHk7/w6do5) but rather as sets of (recently or distantly) related individuals of potentially diverse ancestries.

By plotting the clusters on a map as well as investigating the relation between clusters and regions and comparing IBD communities with PCA and ADMIXTURE[^121^](https://paperpile.com/c/UPmHk7/7NnVp) estimates, a number of general patterns are apparent (Fig. S26-31):

- Cluster **1.1** (“Slavic Period”) contains mostly individuals from Slavic Period Eastern Germany, Poland and Ukraine, the Balkans as well as adjacent regions such as the Baltics, Southern Sweden (Gotland) and Northwestern Russia. It can be further split into clusters 1.1.1 (“Northern SP”), which contains most of the genomes from Eastern Germany, Poland, Ukraine, Czech Republic, Scandinavia, Russia and the Baltics, and cluster 1.1.2 (“Southern SP”), which contains most of the samples from Austria, Hungary, Croatia, Serbia, Italy, Albania, Greece, Bulgaria and Turkey. Both sub-clusters feature a majority of BAL-related ancestry but differ in their admixture proportions. In contrast to individuals from cluster 1.1.1, who show admixture with both western and southern Europe sources /indicated by high proportions of CNE, NOR, CWE and WAS ancestry), individuals from cluster 1.1.2 obtain nearly all of their non-BAL-related ancestry from southern European and West Asian sources (indicated by excess CWE, WAS, and NEA ancestry). Furthermore, individuals from cluster 1.1.2 feature overall lower levels of BAL ancestry, suggesting more regular (or impactful) admixture events with sources lacking Northeastern European-derived ancestry (Ex. Fig. 4).
- Cluster **1.2** (“Southern Scandinavia”) contains mostly individuals from Iron Age, Roman Period and MP continental Northwestern Europe (e.g. Denmark, northern Germany, and the Netherlands) but also MP genomes from England as well as Viking Period samples from Southern Sweden. Individuals from this cluster show predominantly CNE ancestry but also admixture with (predominantly) NOR-, WBI- and CWE-related sources. This cluster (and set of respective individuals) is highly congruent with the “Southern Scandinavian” cluster previously identified by McColl et al. using a similar hierarchical IBD clustering approach[^134^](https://paperpile.com/c/UPmHk7/FJrFO).
- Cluster **1.3** (“Northern Scandinavia”) contains mostly individuals from Iron Age, Roman Period and early Viking Period Sweden, the Baltics and Poland. Individuals from this cluster show predominantly NOR ancestry but also admixture with (predominantly) CNE-, BAL- and CWE-related sources. This cluster (and set of respective individuals) is highly congruent with the “Eastern Scandinavian” cluster previously identified by McColl et al. using a similar hierarchical IBD clustering approach[^134^](https://paperpile.com/c/UPmHk7/FJrFO).
- Cluster **1.6** (“Baltic”) contains mostly individuals from Iron Age, Roman Period and Viking Period Sweden, the Baltics and Finland. Individuals from this cluster carry nearly exclusively BAL ancestry.
- Cluster **2.1** (“CentralEurope BAIA”) contains mostly individuals from Bronze and Iron Age Czech Republic, southern Germany, Slovakia, Slovenia, Switzerland and Hungary.
- Cluster **2.2** (“SouthernEurope BAIA”) contains mostly individuals from Bronze and Iron Age Italy, Sardinia, Croatia, North Macedonia, Montenegro, Bulgaria and Slovenia. Individuals from this cluster show predominantly CWE ancestry as well as smaller proportions of WAS ancestry.
- Cluster **2.3** (“EasternEurope BAIA”) contains mostly individuals from Bronze and Iron Age Czech Republic, Hungary, Slovakia, Ukraine and Poland. It can be further split in clusters 2.3.1 (“EasternEurope BAIA West”), which contains most of the genomes from Czech Republic and Hungary, and cluster 2.3.2 (“Eastern Europe BAIA East”), which contains most of the samples from Poland, Ukraine and Slovakia.
- Cluster **3.1** (“England_EastYorkshire”) contains mostly individuals from Iron Age EastYorkshire, England. Individuals from this cluster show predominantly WBI ancestry as well as smaller proportions of CWE ancestry.
- Cluster **3.2** (“Britain BA”) contains mostly individuals from Bronze Age England, Scotland and Wales but also genomes from Bronze and Iron Age France and the Netherlands. Individuals from this cluster show predominantly WBI ancestry as well as smaller proportions of CWE ancestry.
- Cluster **3.3** (“Britain IA”) contains mostly individuals from Iron Age England and Scotland. Individuals from this cluster show predominantly WBI ancestry as well as smaller proportions of CWE ancestry.
- Cluster **3.4** (“Ireland & Scotland”) contains mostly individuals from Viking Period and Medieval Ireland, Scotland, Orkney and the Isle of Man. Individuals from this cluster carry nearly exclusively WBI ancestry.
- Cluster **3.5** (“Western Scandinavia”) contains mostly individuals from Iron Age, Viking Period and Medieval Norway, Iceland, Greenland, England and the Faroes. Individuals from this cluster show predominantly NOR ancestry but also substantial proportions of WBI ancestry. This cluster (and set of respective individuals) is highly congruent with the “Western Scandinavian” cluster previously identified by McColl et al. using a similar hierarchical IBD clustering approach[^134^](https://paperpile.com/c/UPmHk7/FJrFO).
- Cluster **4** (“Pannonia Avar”) contains mostly individuals from Avar Period Pannonia (Austria and Hungary) as well as Iron Age and Medieval genomes from Kazakhstan, Mongolia, and China. It can be further split into four sub-clusters: 4.1 (Avar Period genomes from Austria and Hungary), 4.2 (Avar Period genomes from Hungary), 4.2 (Medieval genomes from Mongolia) and 4.2 (Avar Period elite burials).
- Cluster **5** (“Western Steppe”) contains mostly individuals from Bronze and Iron Age western and central Russia, Kazakhstan, and Tajikistan but also genomes from Ukraine and Uzbekistan. It can be further split in clusters 5.1 (“WesternSteppe IA”), which contains most of the Iron Age genomes from Kazakhstan and Russia, and cluster 5.2 (“WesternSteppe BA”), which contains most of the Bronze Age samples from Russia, Kazakhstan and Tajikistan.
- Cluster **8** (“Spain BAIA”) contains mostly individuals from Bronze and Iron Age Spain. Individuals from this cluster carry nearly exclusively CWE ancestry.
- Cluster **9** (“Eastern Steppe”) contains mostly individuals from Bronze and Iron Age Mongolia, China, Kyrgyzstan and Kazakhstan.
- Cluster **11** (“Finland & Russia”) contains mostly individuals from Iron Age and Viking Period Russia and Scandinavia. Individuals from this cluster carry nearly exclusively FIN ancestry.
- Cluster **15** (“Armenia BAIA”) contains mostly individuals from Bronze and Iron Age Armenia.


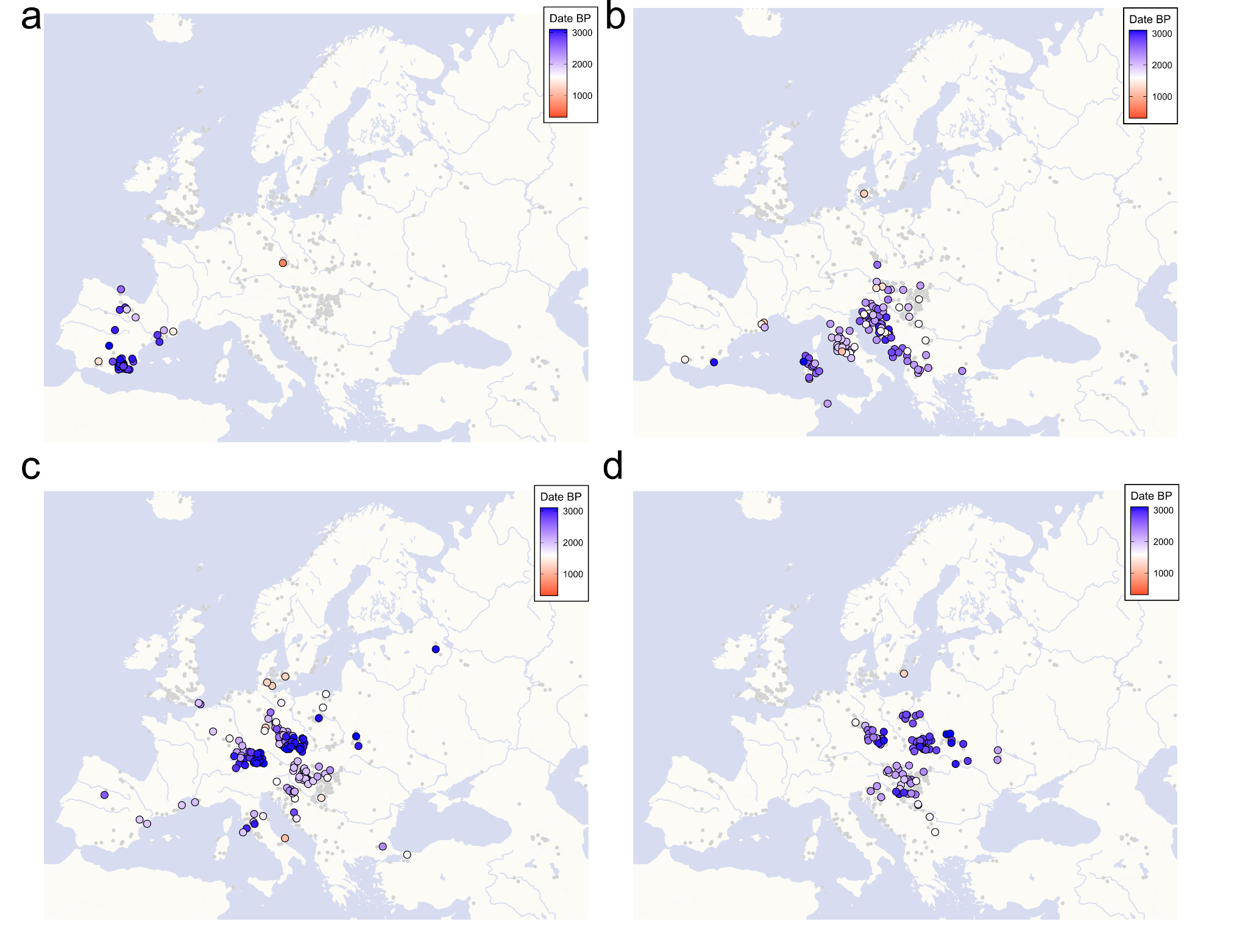


***Supp. Fig. 26. Maps highlighting IBD-sharing communities identified applying a hierarchical cluster detection approach to a network constructed from pairwise IBD-sharing similarities.*** *a) Cluster 8 (“Spain BAIA”). b) Cluster 2.2 (“SouthernEurope BAIA”). c) Cluster 2.1 (“CentralEuropeBAIA”). d) Cluster 2.3 (“EasternEuropeBAIA”).*


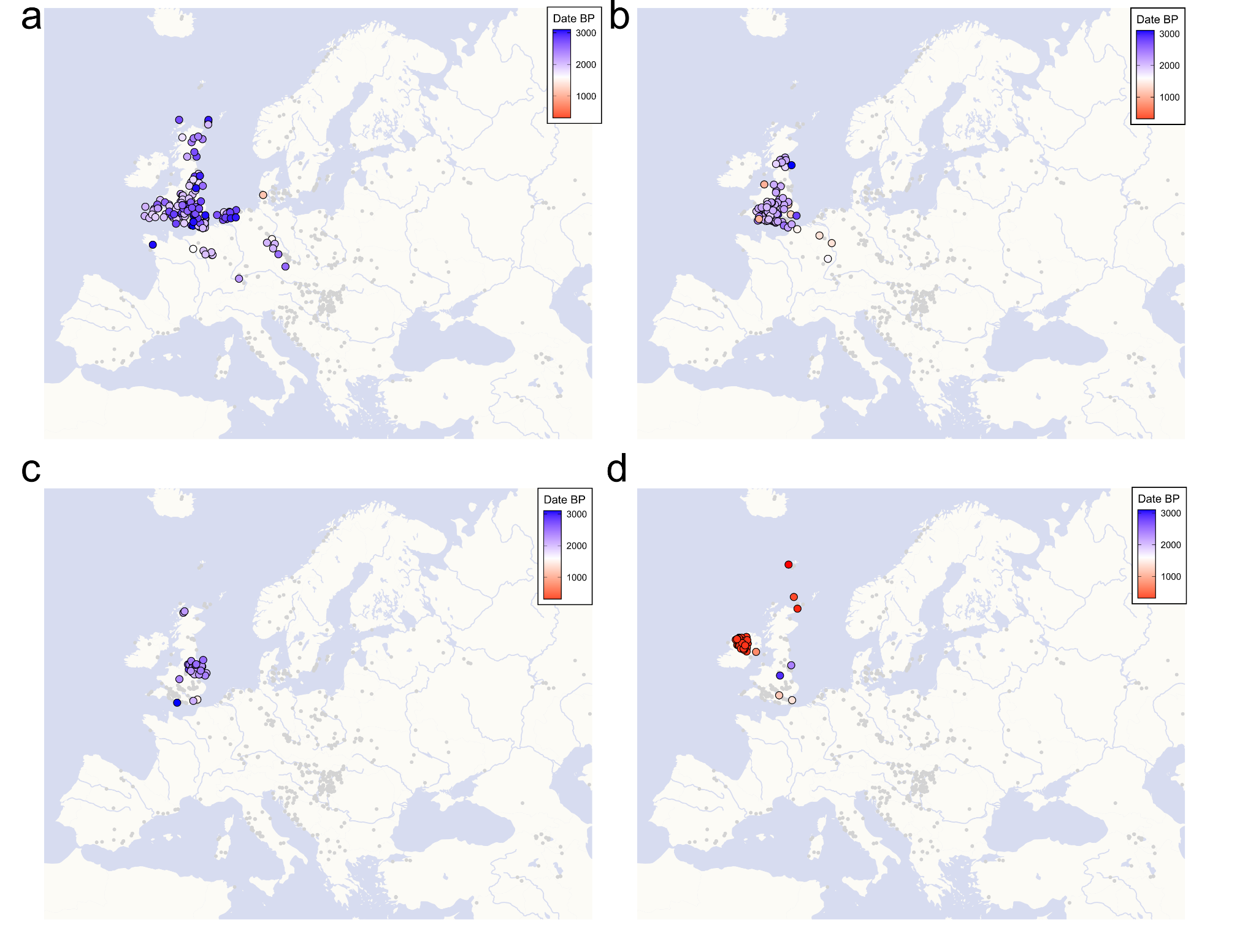


***Supp. Fig. 27. Maps highlighting IBD-sharing communities identified applying a hierarchical cluster detection approach to a network constructed from pairwise IBD-sharing similarities.*** *a) Cluster 3.2 (“Britain BA”). b) Cluster 3.3 (“Britain IA”). c) Cluster 3.1 (“EastYorkshire IA”). d) Cluster 3.4 (“Ireland & Scotland”).*


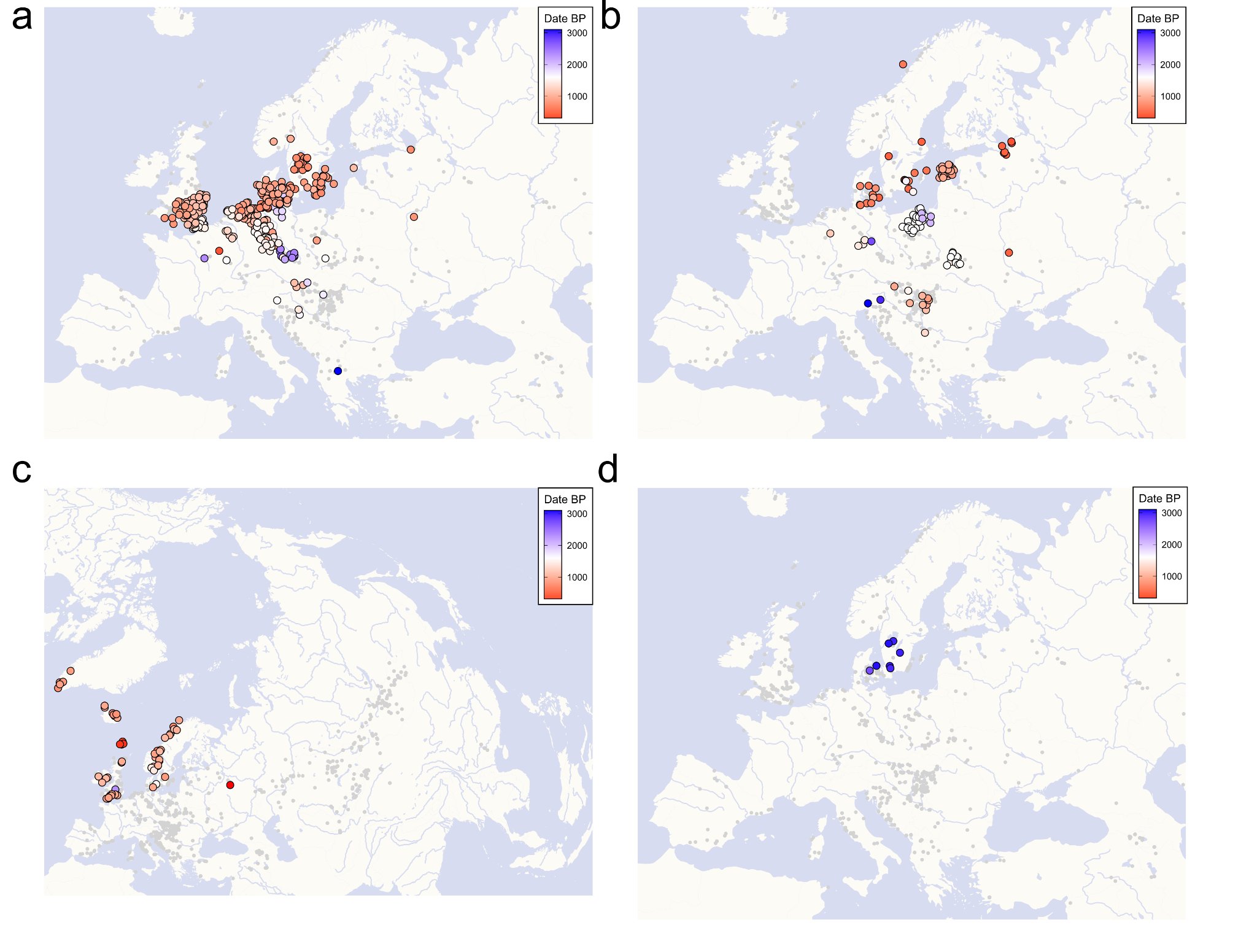


***Supp. Fig. 28. Maps highlighting IBD-sharing communities identified applying a hierarchical cluster detection approach to a network constructed from pairwise IBD-sharing similarities.*** *a) Cluster 1.2 (“SouthernScandinavia”). b) Cluster 1.4 (“NorthernScandinavia”). c) Cluster 3.5 (“WesternScandinavia”). d) Cluster 13 (“Scandinavia LateNeolithic”).*


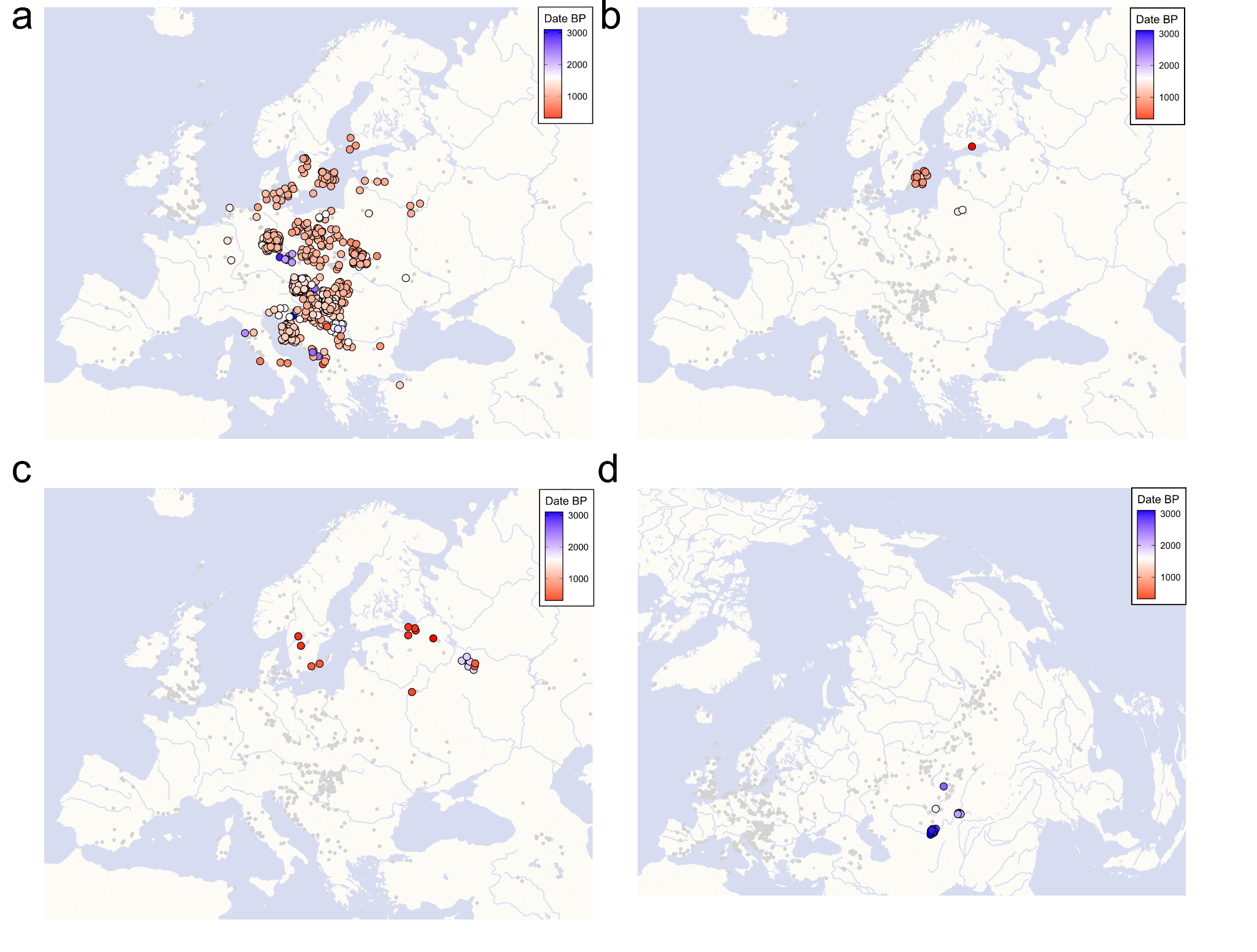


***Supp. Fig. 29. Maps highlighting IBD-sharing communities identified applying a hierarchical cluster detection approach to a network constructed from pairwise IBD-sharing similarities.*** *a) Cluster 1.1 (“Slavic Period”). b) Cluster 1.6 (“Baltic”). c) Cluster 11 (“Finland & Russia”). d) Cluster 10 (“Uzbekistan IA”).*


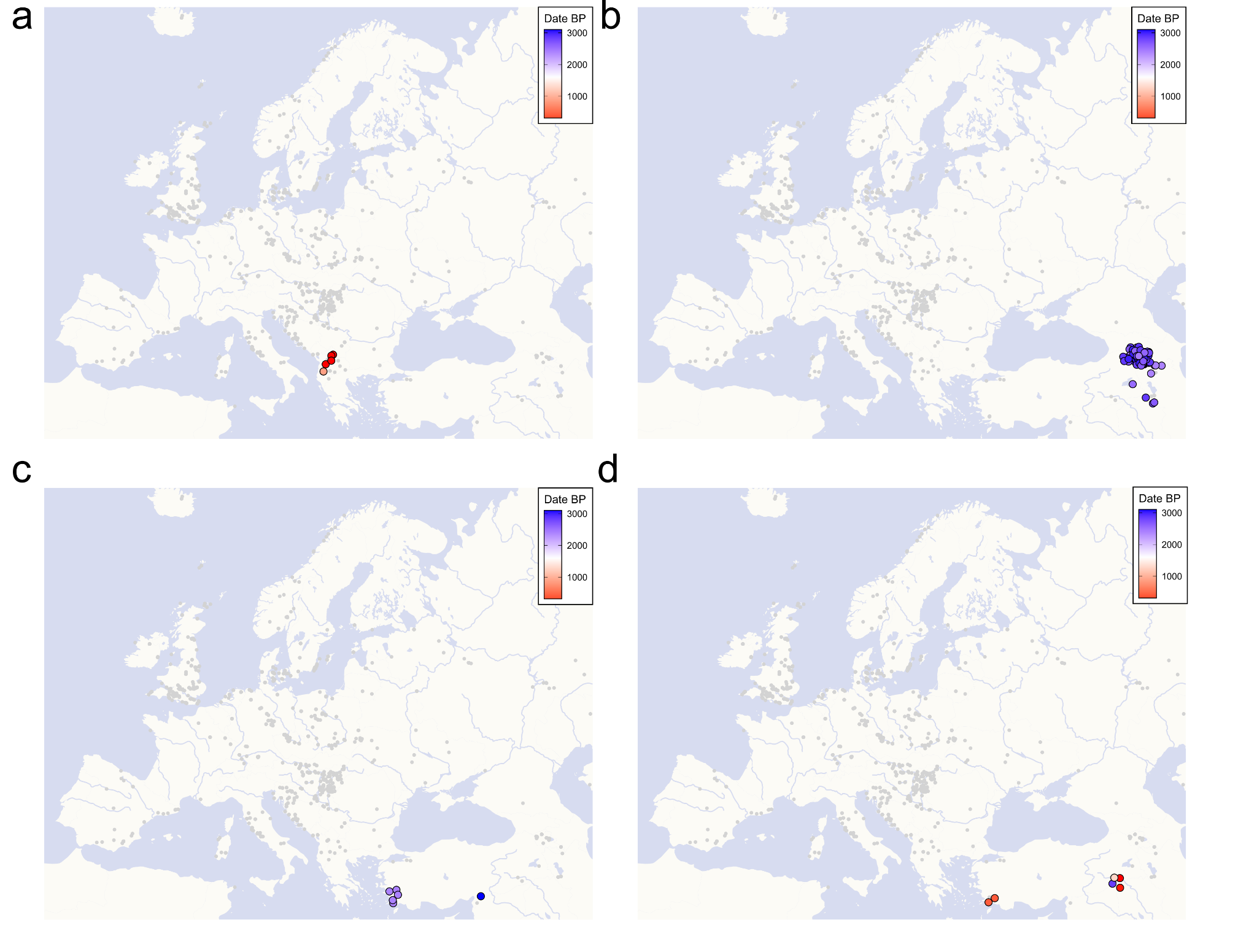


***Supp. Fig. 30. Maps highlighting IBD-sharing communities identified applying a hierarchical cluster detection approach to a network constructed from pairwise IBD-sharing similarities.*** *a) Cluster 14 (“Albania H”). b) Cluster 15 (“Armenia BAIA”). c) Cluster 6 (“Turkey Archaic”). d) Cluster 19 (“Turkey H”).*


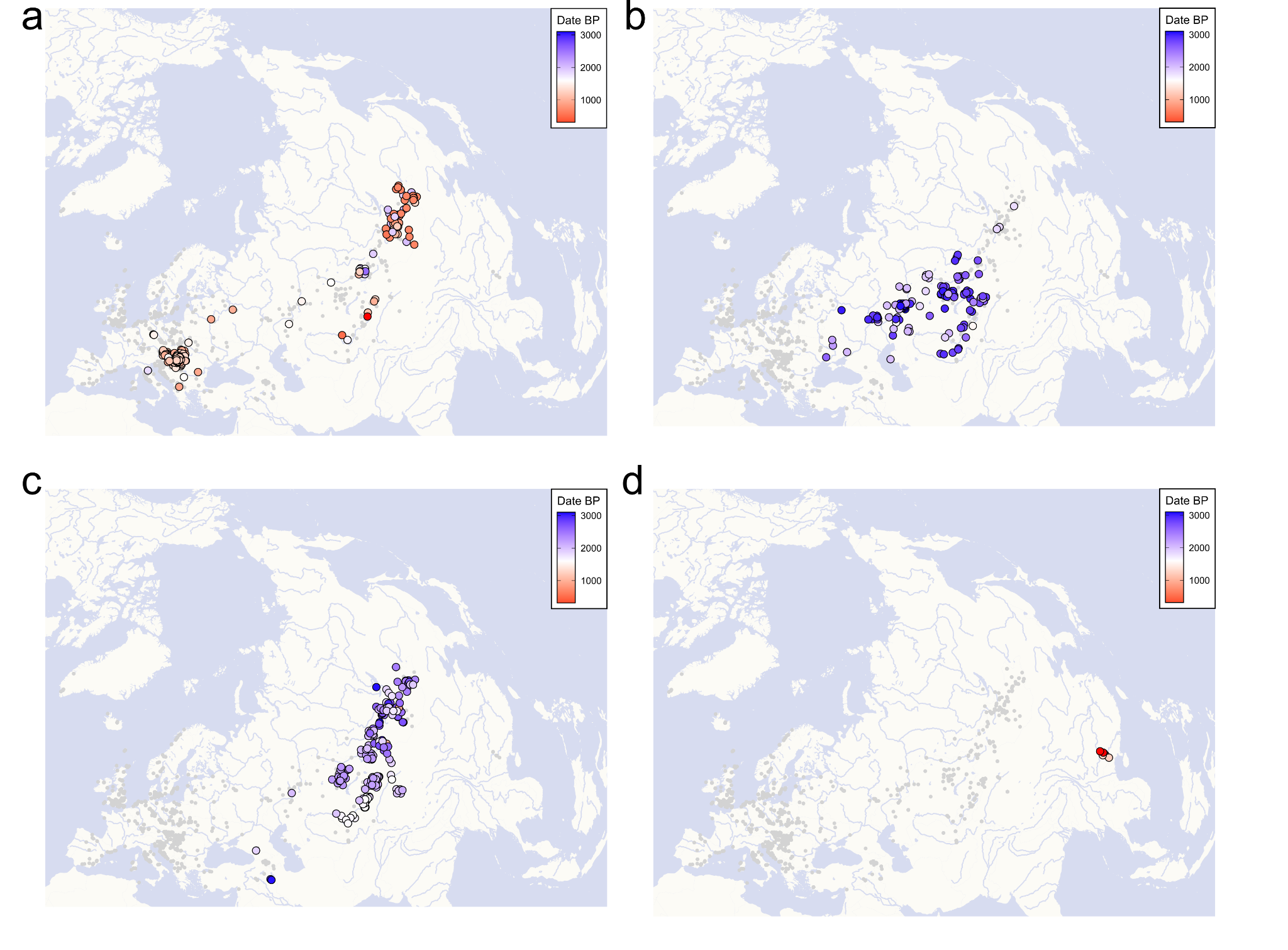


***Supp. Fig. 31. Maps highlighting IBD-sharing communities identified applying a hierarchical cluster detection approach to a network constructed from pairwise IBD-sharing similarities.*** *a) Cluster 4 (“Pannonia Avar”). b) Cluster 5 (“WesternSteppe”). c) Cluster 9 (“EasternSteppe”). d) Cluster 12 (“China Medieval”).*

Due to the lack of ancient DNA from Iron Age and Roman Period Ukraine, Belarus and Western Russia (which is partly the result of the predominance of cremation practices in these regions during this time period), the geographical locations of the earliest members of the “Slavic Period” cluster, predating its spread in the second half of the 1^st^ millennium CE, are indicative of the origin of this population. During the Roman Period (here from 0 CE to 400 CE), members of the SP cluster were already widespread across East-Central Europe (Fig. S32a). For example, we detect four individuals in present-day Poland, both in the Southeast (close to the Ukrainian and Belarussian borders) as well as in the North at the coast of the Baltic Sea. Even more genomes belonging to the Slavic Cluster can be identified in Pannonia and the western Balkans, specifically Croatia, Serbia and Montenegro, indicating a continuous presence of individuals with SP-related ancestry west of the Carpathians during the Roman Period, predating any later, large-scale migration.

Some of the earliest members of the SP cluster, dating 800 BCE to 200 BCE, are found in the Czech Republic, Hungary, Croatia, and Montenegro (Fig. S32b). The oldest individuals belonging to the SP cluster that also receive the majority of their ancestry from a Northeastern European-related source are an Iron Age individual from Montenegro (I13170), a Late Bronze Age individual (associated with the Knoviz culture) from the Czech Republic (I16089), and two Middle Late Bronze Age individuals from Croatia (I18721 and I18723).

In contrast, we identify no members of the SP cluster in Ukraine or Russia during this period. However, again this might be related to the generally low number of genomes from these regions during the Bronze Age, Iron Age and Roman Period. Furthermore, we highlight that, while many of the Southeastern and East-Central European individuals show direct links of genetic relatedness to later SP individuals, such links might not always be representative of their overall genetic make-up (capturing signals of distant relatedness rather than shared ancestry). Instead, our IBD results might recapitulate a network of long-term, recurrent genetic exchange between Pannonia/the Northern Balkans and the ancestors of the SP population in Eastern Europe (Fig. S32b), agreeing with the occurrence of high-BAL-ancestry outlier individuals (>50% BAL ancestry) in these regions since the Bronze Age (Fig. S33, e.g. I13170 (Montenegro_IA), I18183 (Hungary_IA_LaTene_o), I18721 (Croatia_MLBA), R3931.SG (Serbia_Viminacium_Roman_elite_1.SG), I20750 (Hungary_MBA_Fuzesabony), I12106 (Slovakia_IA_Vekerzug)). Without proper sampling of the Eastern Balkans, of Romania and Bulgaria, it remains speculative if these regions were part of this genetic network as well.

When directly comparing the IBD-sharing patterns (> 1 cM) between the SP population of Gródek (used here as proxy for the original SP gene pool due to their old age and close geographical proximity to Belarus and Ukraine), and other Bronze Age, Iron Age and Roman Period groups in Europe, we find the highest IBD sharing with Iron Age (Barrow Culture) and Roman Period individuals from Lithuania, followed by Roman Period individuals from Croatia and Serbia, as well as as Bronze Age individuals from Ukraine (Komarów Culture) and eastern Poland (Strzyżów Culture)[^109,139^](https://paperpile.com/c/UPmHk7/sHb1e+Ejy7a) (Fig. S32c, Table S39). Thus, highest genetic relatedness is established with these individuals and groups, although they form their own respective IBD clusters, suggesting an ancestral connection that predates more recent connections to East-Central Europe and the Balkans.

*
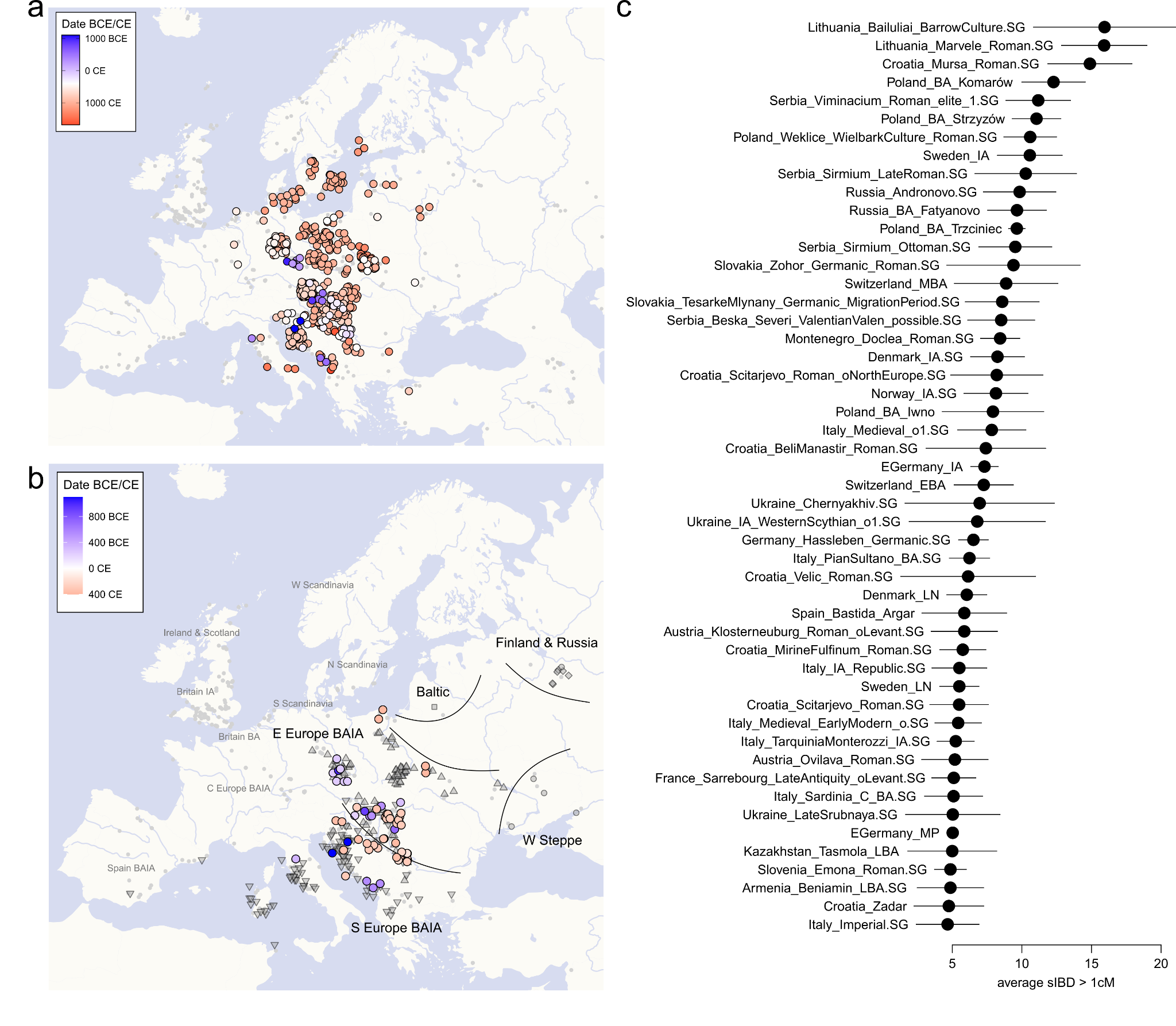
*

***Supp. Fig. 32. Affinities of the SP IBD-sharing community.*** *a) The complete cluster 1.1 (“Slavic Period”). Samples are coloured to according to their respective age. b) Only samples belonging to cluster 1.1 older than 400 CE are shown. Similarly, samples belonging to cluster 5 (“Western Steppe”), cluster 1.6 (“Baltic”), cluster 11 (“Finland & Russia”), cluster 2.2 (“SouthernEurope BAIA”) and cluster 2.3 (“EasternEuropeBAIA”) older than 400 CE are indicated. c) Average sIBD between GRK individuals and Bronze Age, Iron Age and Roman Period groups in Europe. Only the 50 highest estimates are shown.*

*
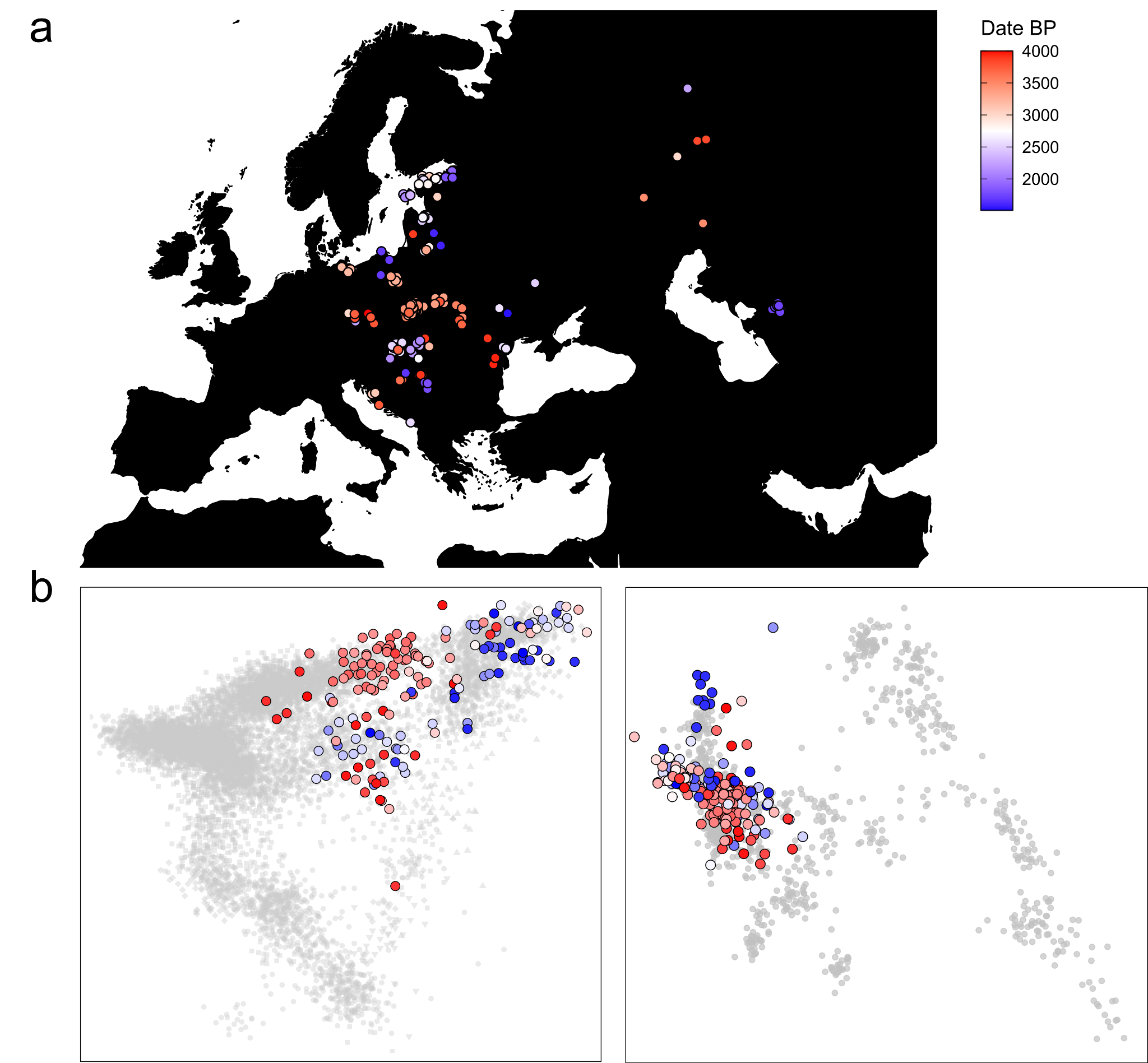
*

***Supp. Fig. 33. Eastern European ancestry through time and space.*** *a) Shown are the locations of 176 samples with more than 50% BAL ancestry. b) Shown is the distribution of these samples in PCA space; left: European PCA, right: West Eurasian PCA.*

### 4.4 Affinities to ancient population

#### 4.4.1 F_3_ and F_4_ statistics

Diverse analyses comparing the Central/East-Central European SP populations to present-day European populations suggest high genetic similarity between the different SP groups as well as highest genetic affinity to modern Eastern and Northeastern European populations. However, populations change gradually over time through drift and admixture, so present-day populations may be poor proxies for ancient groups of unknown genetic makeup.

Consequently, we compared our SP genomes to other ancient genomes dating between 3,000 BCE and 400 CE (Fig. S34a). First, we calculated outgroup F_3_ statistics of the form F_3_(GRK, Test; YRI) where individuals from Gródek (GRK), Eastern Poland, represent the surrogate for SP ancestry (due to its geographical proximity to the proposed origin of the SP gene pool) and Test iterates through 3,892 ancient individuals dating between 3000 BCE and 400 CE (i.e. shortly before the proposed Slavic expansion) (Table S25). In congruence with the analysis of the present-day genetic diversity, we observed the highest affinity to Bronze Age groups from Lithuania, Latvia and Estonia as well as Iron Age individuals from Ingria (Fig. S34b). A similar signal is observed when calculating pairwise F_3_-statistics on the group level: SP groups from Eastern Germany, Poland and Ukraine show highest amounts of shared drift with each other as well as with Bronze Age, Iron Age and medieval groups from Lithuania, Latvia and Estonia (Fig. S34c).

Next, we computed F_4_ statistics of the form F_4_(YRI, Test; MP, SP) for our three study transects as well as for the Russian Volga-Oka region using OBM, BBC, Poland_Roman and Russia_VolgaOka_IA as proxy for the MP ancestries and NDW, VEM, Poland_EMA and Russia_VolgaOka_MA as proxy for the SP ancestry in Eastern Germany (Fig. S35a, Table S28-31), the Northwestern Balkan (Fig. S35b), Poland-Northwestern Ukraine (Fig. S35c) and the Volga Oka area (Fig. S35d), respectively.

For Eastern Germany and Poland-Northwestern Ukraine, we note that Iron Age and early medieval Northwestern European populations such as Denmark_IA, Norway_IA, Sweden_IA, Denmark_EarlyViking or Sweden_Viking tend to be significantly (*Z* < -2) more closely related to the MP than to the SP population. On the other hand, Iron Age and Bronze Age Eastern Europeans (e.g. Lithuania_BA, Estonia BA, Latvia_BA, Lithuania_Marvele_Roman and Russia_VolgaOka_IA) as well as contemporaneous SP groups are significantly more closely related to the SP population (*Z* > 2).

In the Northwestern Balkan, we observe significantly higher genetic similarity between diverse ancient Southern European (e.g. Croatia_EIA, Bulgaria_EIA and Italy_Imperial), and West Asian groups (e.g. Israel_MLBA, Turkey_Alalakh_MLBA and Turkey_WestByzantine) and the pre-SP population than with Slavic Period individuals. In contrast, Northern European populations seem to be in general significantly more closely related to the SP individuals than to the pre-SP genomes. Specifically, the F_4_ statistics are maximized for ancient Northeastern Europeans and SP individuals (e.g. Lithuania_BA, Estonia BA, Latvia_BA, Lithuania_Marvele_Roman, Russia_VolgaOka_IA and Russia_Ingria_IA as well as NDW, SDN and Poland_EMA), evidencing highest genetic similarity to ancient groups from the Baltics and other SP populations and suggesting genetic influx from the East and Northeast (not from the Northwest).

In summary, the SP populations of all three study transects show significantly higher affinities to ancient Eastern and Northeastern Europeans than their pre-SP predecessors. The most notable exception from this pattern is detectable in the Volga-Oka area, where we find that most ancient Western and Central European populations are symmetrically related to the SP and pre-SP individuals. Within Europe and the Middle East, we only detect Italy_LA and Lebanon_MBA to be significantly more closely related to the SP individuals than to the pre-SP genomes, indicating a Southern/Western influence into the Volga Oka region during the Slavic Period, agreeing with results from PCA, ADMIXTURE and F_4_ statistics. This is furthermore supported by the fact that diverse ancient Central Asian (e.g. Kazakhstan_Zevakinskiy_LBA, Kazakhstan_Berel_IA, and Kazakhstan_Sarmatian_IA), East Asian (e.g. Taiwan_Hanben_IA, China_Xinjiang_Jierzankale_IA and Mongolia_EIA_SlabGrave_1) and Northeastern European (e.g. Russia_Bolshoy, Finland_Levanluhta and Latvia_BA) genomes show higher affinity to the pre-SP population, demonstrating the presence of excess Southern/Western European ancestry in the SP gene pool.

To investigate this signal of increased Eastern European affinity, we further calculated F_4_-statistics of the form F_4_(YRI, SP; Test, MP) to test which European groups are directly closer related to the SP genomes than to their MP predecessors (Fig. S36, Table S27). In congruence with the results above, we find Northeastern European (especially Lithuania_BA and Latvia_BA[^140^](https://paperpile.com/c/UPmHk7/PSVWs)) as well as contemporary SP genomes to be consistently more closely related to the SP genomes from our transects than their respective MP predecessors.

Next, we tried to identify the best proxy for this incoming Eastern European ancestry. For that, we computed F_4_-statistics of the form F_4_(YRI, Test1; Test2, Test3) to identify ancient populations most similar to our SP genomes (Fig. S37a). When applying hierarchical cluster analysis using Ward’s minimum variance method to the results we find that all tested SP groups form a clade together with Latvia_BA as outgroup (Fig. S37a). Based on these and the previously described results, we grouped Lithuania_BA and Latvia_BA together as *Baltics_BA* and calculated F_4_-statistics of the form F_4_(YRI, GRK; Baltics_BA, Test) to identify potential ancient genomes that may be even closer related to the Gródek individuals (Table S26). Across 512 post-Mesolithic groups from Europe and West Asia, we only identify Latvia_MN and Lithuania_EMN_Narva as being significantly more closely related to SP individuals from Gródek than *Baltics_BA* (Fig. S37b). Accordingly, we conclude that a population closely related to the Hunter-Gatherer populations of Lithuania and Latvia was the most likely donor of the WHG-enriched ancestry in the common ancestor of all SP populations.

Additionally, we also computed pairwise F_4_-statistics of the form F_4_(YRI, SP; TestA, TestB) iterating through nine WHG-enriched ancient (North-)Eastern European groups (Fig. S38). Throughout all combinations, we infer Lithuania_BA[^140^](https://paperpile.com/c/UPmHk7/PSVWs) and Latvia_BA[^140^](https://paperpile.com/c/UPmHk7/PSVWs) as showing the highest genetic similarity to the SP groups in Eastern Germany, the Northwestern Balkan, Poland-Northwestern Ukraine and the Volga-Oka region.


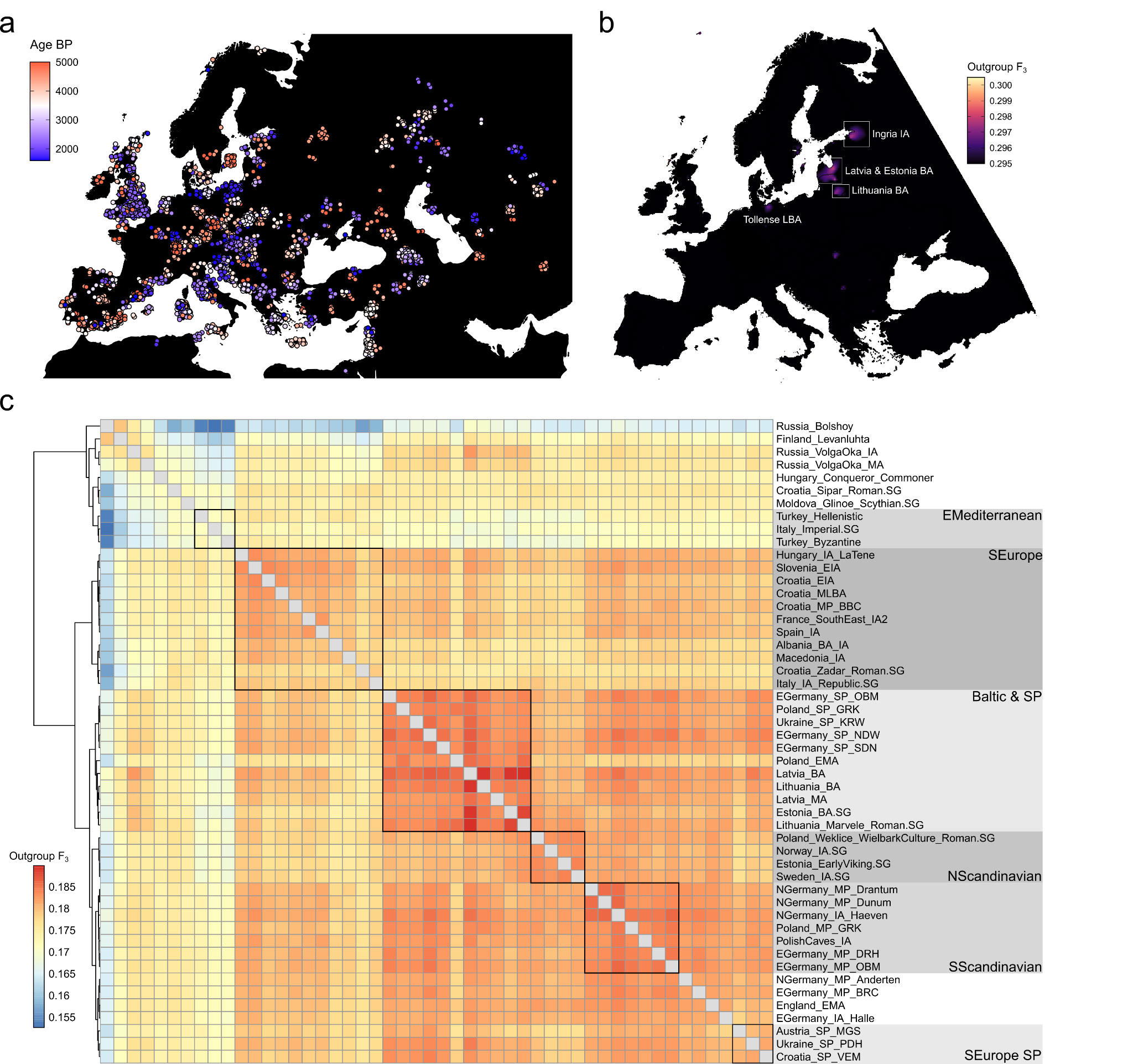


***Supp. Fig. 34. Allele sharing across Europe.*** *a) Geographic distribution of ancient samples (older than 400 CE) included in the analysis. Random jitter was added for sites with multiple individuals. b) Kriging interpolation of shared drift (as measured using outgroup F_3_-statistics of the form F_3_(YRI; GRK, Test)) between the early SP Gródek population from Eastern Poland (n = 8) and 5563 ancient Europeans predating 400 CE. c) Heatmap of pairwise F_3_-statistics of the form F_3_(YRI; TestA, TestB) between 53 selected ancient populations. Rows and columns were ordered according to hierarchical cluster analysis applying Ward’s minimum variance method.*


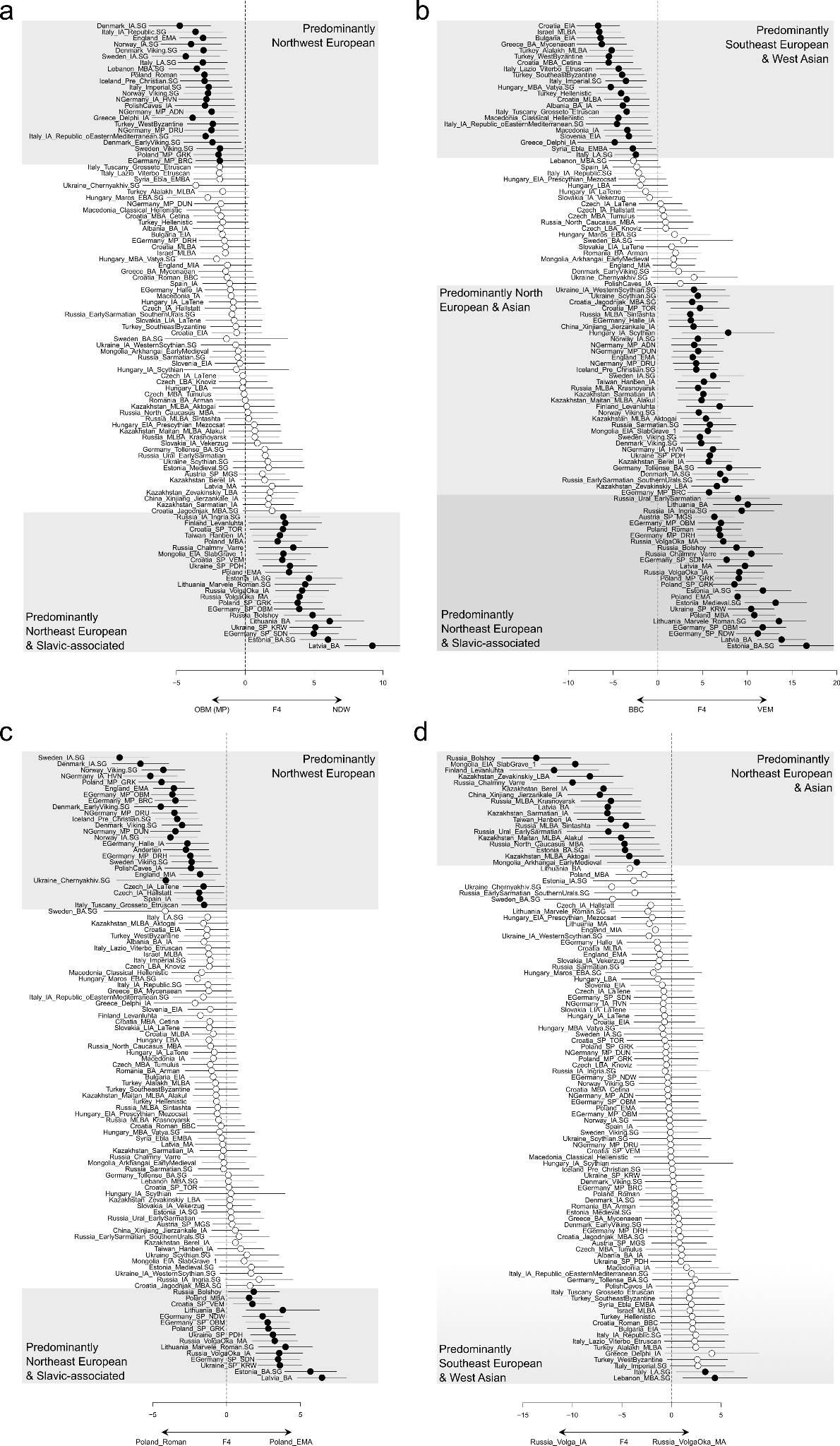


***Supp. Fig. 35. Genetic affinities of individuals of pre-SP (Roman Period & MP) and SP genomes****. a) Shown are the results of the F_4_-statistic of the form F_4_(YRI, Test; MP, SP) for MP (n = 181) and SP (n = 240) genomes from Eastern Germany in comparison to 102 relevant ancient European populations. b) same as panel a) but for pre-SP (n = 67) and SP (n = 80) genomes from Croatia. c) For pre-SP (n = 100) and SP genomes (n = 207) from Poland. d) For pre-SP (n = 9) and SP (n = 15) genomes from the Russian Volga-Oka region. Error bars indicate two standard errors. Significant statistics (|Z| > 2) are shown in black, non-significant statistics (|Z| < 2) in white.*


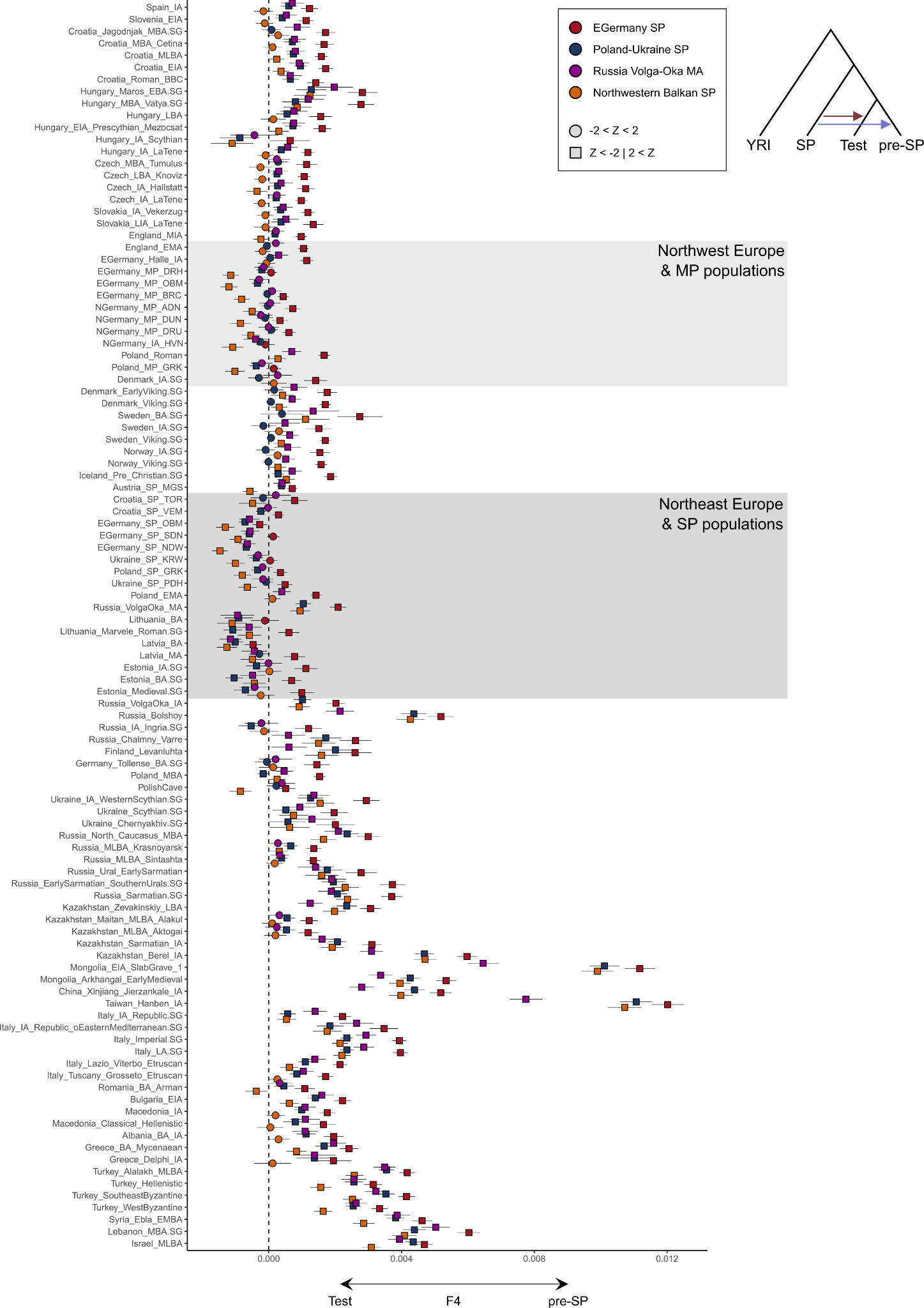


***Supp. Fig. 36. Genetic affinities of pre-SP (Roman Period & MP) and SP individuals from the Northwestern Balkans, Eastern Germany, Poland-Northwestern Ukraine and the Russian Volga-Oka region.*** *Shown are the results of the F_4_-statistic of the form F_4_(YRI, SP genomes; Test, pre-SP genomes) for 102 relevant ancient populations from Europe and Asia in comparison to pre-SP and SP genomes from four study transects (n = 67, 181, 100, 9 and n = 80, 240, 207, 15, resp). Error bars indicate two standard errors.*

*
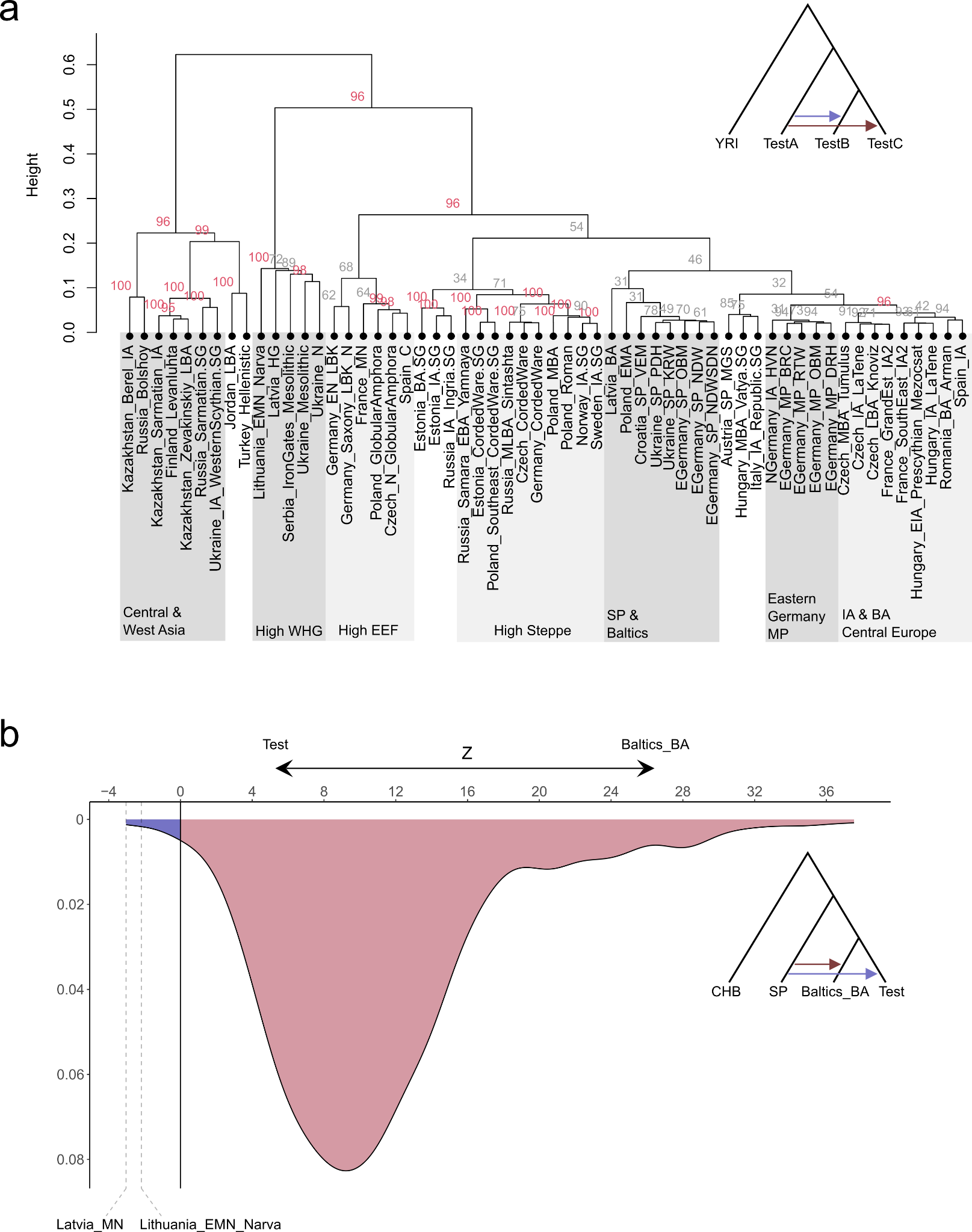
*

***Supp. Fig. 37. Genetic affinities of ancient SP individuals from the Northwestern Balkans, Eastern Germany, and Poland-Northwestern Ukraine.*** *a) Hierarchical cluster analysis applying Ward’s minimum variance method to the results of F_4_-statistics of the form F_4_(YRI, Test1; Test2, Test3) between 58 ancient groups. The dendrogram and statistical support for the bifurcations from multiscale bootstrap resampling are shown. b) Shown are Z values obtained from F_4_-statistics of the form F_4_(CHB, GRK; Baltics_BA, Test) as kernel density estimate. Baltics_BA includes ancient genomes from the groups Lithuania_BA and Latvia_BA (n = 14); Test iterates through 512 relevant Neolithic and post-Neolithic populations from Europe. The lowest Z-values in the data, for Latvia_MN and Lithuana_EMN_Narva, are highlighted.*


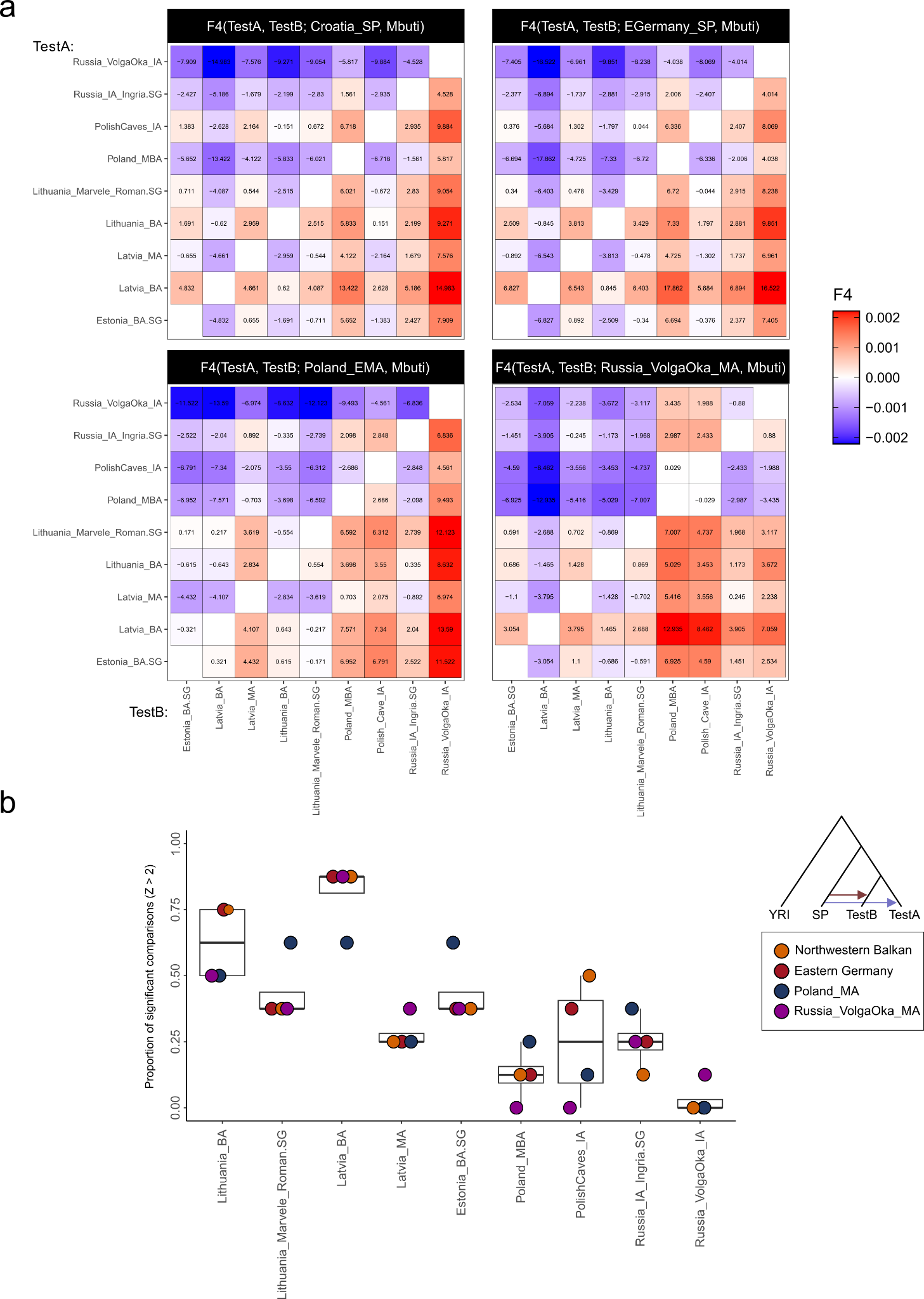


***Supp. Fig. 38. Genetic affinities of ancient SP individuals from the Northwestern Balkan, Eastern Germany, Poland-Northwestern Ukraine, and the Russian Volga-Oka region to post-Neolithic populations from the Baltics.*** *a) Shown are the results of pairwise F_4_-statistics of the form F_4_(YRI, SP; TestA, TestB) as a symmetrical heatmap for SP genomes from the four study transects (n = 80, 240, 207, 15). TestA and TestB iterate through 9 relevant post-Neolithic populations from Central-East Europe and the Baltics (n = 9, 5, 72, 4, 4, 5, 5, 10, 13). Z-values for each F_4_-statistics are indicated in the respective tile of the matrix. b) The percentage of significant comparisons from the F_4_-statistics presented in panel a) shown as boxplots for each of the TestA/TestB groups (highlighting the percentage of F_4_-statistics where the denoted population is more* closely *related to the SP genomes than the comparison). Bounds of the Box represent the 25^th^ and 75^th^ Percentile. The center represents the median. Whiskers represent the smallest value greater than the 25^th^ Percentile minus 1.5 times the interquartile range and largest value less than the 75^th^ Percentile plus 1.5 times the interquartile range, respectively. Outliers present the minimum and maximum values in the data.*

#### 4.4.2 Admixture with southern sources.

Our results (based on present-day and ancient data) suggest that the majority of the ancestry of the SP populations from our study transects was derived from a Baltic Bronze Age-related source. Yet, Bronze Age genomes from the Baltics do not overlap with the SP individuals in PCA space. Instead, the SP genomes are shifted on PC1 to South, indicating admixture with sources in Central and Southeastern Europe.

This is also evident from ADMIXTURE (Fig. S39): we observe that Bronze Age Baltic genomes from Lithuania, Latvia and Estonia can be modeled nearly exclusively with BAL ancestry. However, SP genomes from Eastern Germany, the Northwestern Balkan, Poland-Northwestern Ukraine all feature between ~25% and ~45% non-BAL-related ancestry, mostly CNE, WBI and CWE. All SP populations also feature small proportions of Aegean or West Asian-related ancestry (WAS and NEA) which is absent in earlier Baltic populations. Potential sources of these ancestries might have been the Iron Age populations of East-Central and Southeastern Europe, specifically those residing around the Carpathian mountains, the Pannonian Basin and the Northern Balkans.

We applied the PANE[^141^](https://paperpile.com/c/UPmHk7/a8hm) method that leverages PCA coordinates and Non-Negative Least Square (NNLS) to assess the ancestral compositions of admixed individuals given a set of donor populations[^141^](https://paperpile.com/c/UPmHk7/a8hm) (Fig. S40). Specifically, this approach utilizes the geometric relationship between PCA distances and *F*-statistics possible to infer admixture proportions of a target population using PCA coordinates of the target and potential donors. We applied PANEto a combined dataset of the representative first 10 PCs of three different PCA setups (I. the West Eurasian PCA, calculated on the HO panel, as well as II. the European and III. Northeastern European PCAs, calculated on our high-resolution panel). We then selected 300 projected SP individuals from NDW, SDN, OBM, GRK, KRW and PDH and decomposed their ancestries using 20 putative sources, representing 10 Northern European (Fig. S40a) and 10 Southern European (Fig. S40b) donor groups:

- Albania_BA_IA
- Croatia_EIA
- Czech_LBA_Knoviz
- Estonia_BA.SG
- Estonia_IA.SG
- Finland_Levanluhta
- Greece_Delphi_IA
- Hungary_IA_LaTene
- Latvia_BA
- Lithuania_BA
- Macedonia_IA
- Poland_MBA
- Poland_Roman
- Romania_BA_Arman
- Russia_IA_Ingria.SG
- Russia_VolgaOka_IA
- Slovenia_EIA
- Spain_IA
- Turkey_IA
- Ukraine_IA_WesternScythian.SG

For the Southern European-related component (Fig. S40b), we show that the majority of ancestry in the SP individuals is derived from Albania_BA_IA (10.4%), followed (by far) by Turkey_IA (3%), Hungary_IA_LaTene (2.9%) and Croatia_EIA (2.8%). This result seemingly supports previous observations of Eastern Mediterranean/West Asian-related ancestry in SP individuals (via ADMIXTURE) and might indicate a Southern Balkan-derived ancestry component in the SP gene pool (as previously postulated by Speidel et al.[^133^](https://paperpile.com/c/UPmHk7/MbA4g)). However, given the large geographical distance, it appears unlikely that the ancestors of the SP population admixed in an unmediated way with southern Balkan/Aegean-derived sources. Instead, this southern Balkan-related ancestry might have been transmitted to the North via (so far) unsampled vector populations (for example early Iron Age Thracian groups[^142^](https://paperpile.com/c/UPmHk7/0GMSk)).

For Northern European-related ancestry (Fig. S40a), in congruence with earlier *F*-statistic and IBD results, we measure the highest average contributions from Baltic Bronze Age-related sources, with Lithuania_BA, Latvia_BA and Estonia_BA.SG providing the largest average contributions to the SP gene pool (36.8%, 11.4% and 7.4% for Lithuania, Latvia and Estonia BA, respectively). Interestingly, both Ukraine_IA_WesternScythian.SG and Poland_MBA contribute substantially less ancestry to the SP individuals, although they constitute geographically much closer located sources than the Baltic populations (1.4% and 0.6%, respectively) and also derive the majority of their ancestry from Northeastern Europe.

Applying Ward’s minimum variance method to a symmetrical matrix of euclidean distances calculated from the ADMIXTURE ancestry profiles, we show the highest similarity of SP populations from our transects and the Volga-Oka area with Bronze and Iron Age groups from Lithuania, Latvia, Estonia and Ingria as well as Scythian individuals from Ukraine (Fig. S39). However, while these Iron Age Sythian genomes feature similar fractions of BAL ancestry, their average WBI ancestry proportions (15% to 28%) far exceed the fractions measured in the SP populations (e.g. 4%, 5%, 4%, 3%, 2% and 5% for Steuden, Niederwünsch, Gródek, Poland_EMA, Velim, and Russia_VolgaOka_MA). Furthermore, Sycthian groups from Ukraine show varying fractions of South Asian ancestry (between 5% and 12%), a component present in many ancient individuals from Moldova (e.g. Moldova_IA, Moldova_LBA and Moldova_MBA), Ukraine (Ukraine_Alexandria_MBA and Ukraine_BA_Catacomb.SG), Western Russia (e.g. Russia_EarlySarmatian.SG, Russia_MLBA_Potapovka, or Russia_MLBA_Sintashta) and the Caucasus (Russia_Caucasus_LBA_Dolmen and Russia_North_Caucasus_MBA) but (nearly) absent in the SP genomes from Central and East-Central Europe (<5%) (Fig. S42b). Consequently, we suggest that the Scythian groups in Eastern and Central Ukraine were genetically highly similar to the ancestors of the SP populations in Central Europe but did not directly contribute to the formation of the SP gene pool or only to a minor extent (<~15% ancestry).

This differentiation is also supported by differences in the proportion of Siberian/Central Asian-related Krasnoyarsk Krai BA ancestry[^143^](https://paperpile.com/c/UPmHk7/VDukl) (Table S33). We applied qpAdm to calculate estimates of the mixture coefficients α_WHG_, α_EEF_, α_Steppe_, and α_Krasnoyarski_ using the outgroups described in Petterson et al. 2021[^120^](https://paperpile.com/c/UPmHk7/iIPiM) (Fig. S41a). Mirroring the distribution of SAS ancestry, we measure high fractions of Krasnoyarsk Krai BA ancestry in diverse groups from Moldova, Ukraine and Western Russia yet no or only minor contributions to neighboring populations in the Baltics or SP populations in Central Europe (Fig. S41a). Thus, an Eastern European origin of the SP gene pool without substantial genetic influx from Central Asia/the Caucasus appears most likely. Overall, we note that the SP populations in Central Europe closely resemble other Northern European groups of the Bronze Age and Iron Age, especially in Scandinavia, due to their high proportions of Steppe and WHG-related ancestries (ranging in Eastern Germany, Poland and Ukraine between 47% - 49% and 16% - 18% for Steppe and WHG ancestry respectively) (Fig. S41b,c).

Yet, in comparison to the high WHG proportions observed in Bronze Age, Iron Age or medieval genomes from the Baltics (ranging between 21% and 27%), WHG ancestry is consistently reduced in the SP genomes. On the other hand, Early European Farmer (EEF) ancestry is increased, evidencing subsequent admixture with EEF-enriched, WHG-depleted sources most likely from Central-East or Southeastern Europe (e.g. present-day Poland, Slovakia, Hungary or Romania) (Fig. S41a,c). We highlight this as a major difference to the Middle Bronze Age genomes from Eastern Poland and Northern Ukraine which appear to be mostly admixed between a Baltic Bronze Age and a (Steppe-enriched) Corded Ware-derived sources (as proposed by Chyleński and colleagues[^139^](https://paperpile.com/c/UPmHk7/sHb1e)).

We suggest that WHG-enriched Baltic Bronze Age-related populations expanded and admixed multiple times with groups in Central Europe. In the North (in Germany and Poland), these Baltic Bronze Age-related groups encountered Steppe-enriched populations of mainly Bell Beaker and/or Corded Ware-derived ancestry (Fig. S42a). In the South (in Romania, Hungary and further West), the local populations derived more of their ancestry from the pre-Bronze Age Neolithic sources, resulting in higher fractions of EEF ancestry. For the SP gene pool, we tested which continental populations qualify as source for this EEF-enriched ancestry in a two-way model Baltic_BA + *Test* using qpAdm. We set the option allsnps: YES. For the *Test* population, we selected 473 prehistoric and historic groups from Europe and West Asia (Table S34). While 85 mostly Central, Southern and Southeastern European populations produce fitting models (*p* > 0.01), the majority of those producing *p*-values higher than 0.05 are located in Central-East Europe and the Northern Balkans, indicating that the donor of the EEF-enriched ancestry was most likely located in the Pannonian Basin, around the Carpathian mountains, or the eastern North European Plain (Fig. S43a). Based on these working models, we suggest that the SP gene pool received between ~29% (95 CI: 24% - 33.5%) from such an EEF-enriched source, a proportion very similar to previous estimates of Baltic Bronze Age and Southeastern European-related ancestry in medieval genomes from Poland[^133^](https://paperpile.com/c/UPmHk7/MbA4g) (Fig. S43b).

To closer investigate these differences in ancestry and link them to geographical patterns, we applied MOBEST[^122^](https://paperpile.com/c/UPmHk7/xUFfF) to the PCA coordinates of 15 high-coverage, unadmixed individuals from Niederwünsch, Steuden, Poland_EMA, and Velim along PC1, PC2, and PC3. The MOBEST analyses were run using a kernel size of 800. We set the predication grid to 50 by 50 km tiles. As reference we used 5,664 published individuals dating between 1,600 and 5,000 years BP[^144^](https://paperpile.com/c/UPmHk7/RV6n6). Related individuals and samples with less than 15k SNPs were excluded. We set the relative search time to 0, thus, the probability surface indicates the highest genetic-geographical match at the mean date of the respective individual[^122^](https://paperpile.com/c/UPmHk7/xUFfF). We then averaged the probabilities of each site to obtain a mean probability surface for the four test areas (Fig. S44).

We observe that the resulting similarity probabilities are highly similar between sites, with the highest probabilities being distributed in Northeastern Europe, specifically south of the Baltics (Lithuania, Latvia and Estonia) in the border region of present-day Belarus, Western Russia and Northern Ukraine (Fig. S44). Such a location for initial formation of the SP gene pool is consistent with our results from ADMIXTURE and qpAdm modeling, indicating a geographic origin between the Baltics and Southeastern Europe.


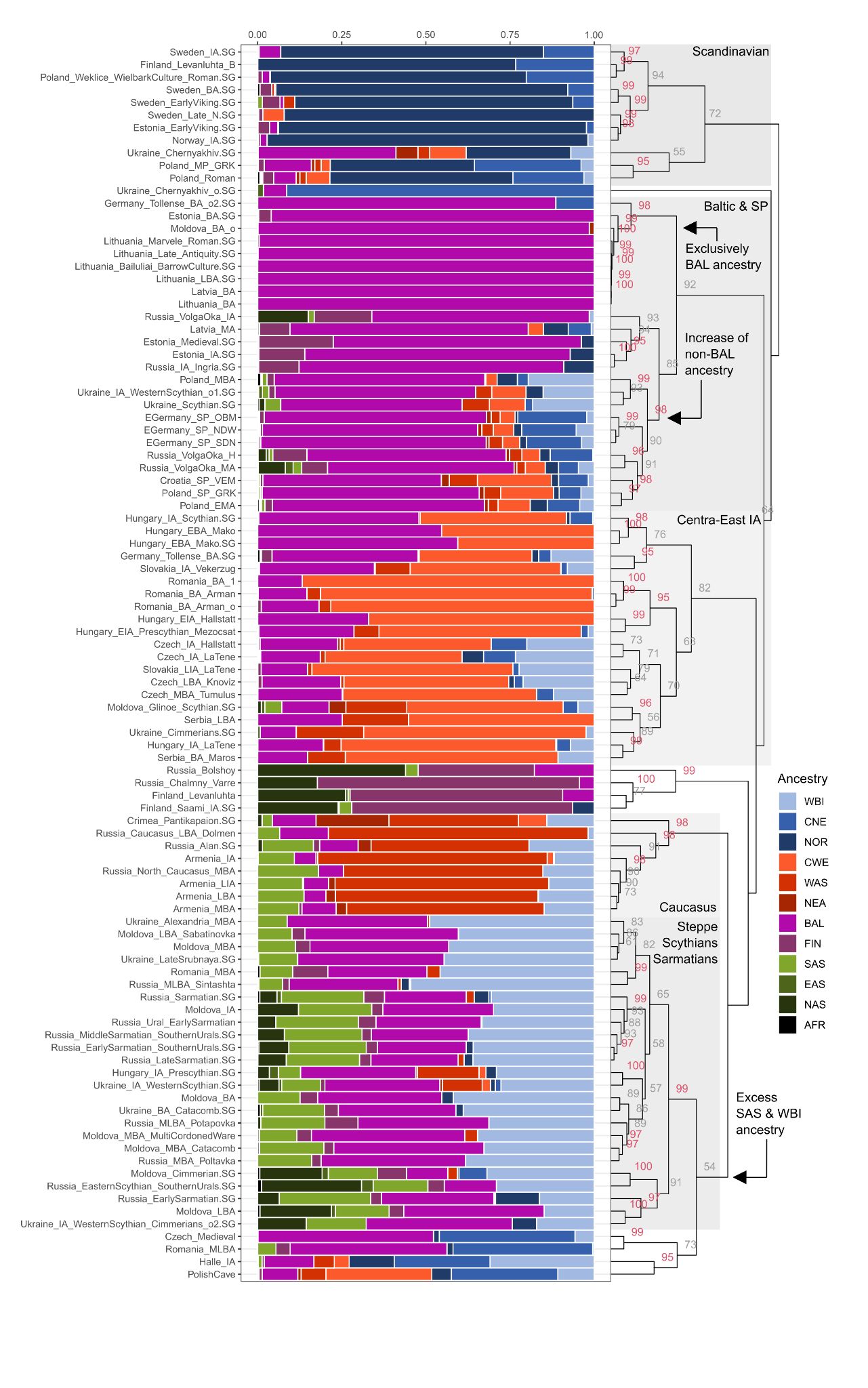


***Supp. Fig. 39. Results from ADMIXTURE analysis.*** *Relevant ancient individuals (n = 1180) from 98 Eastern and Southeastern Europe were decomposed into 12 ancestral sources using a supervised clustering approach. Columns were ordered according to hierarchical cluster analysis applying Ward’s minimum variance method. The dendrogram and statistical support for the bifurcations from multiscale bootstrap resampling are shown.*

*
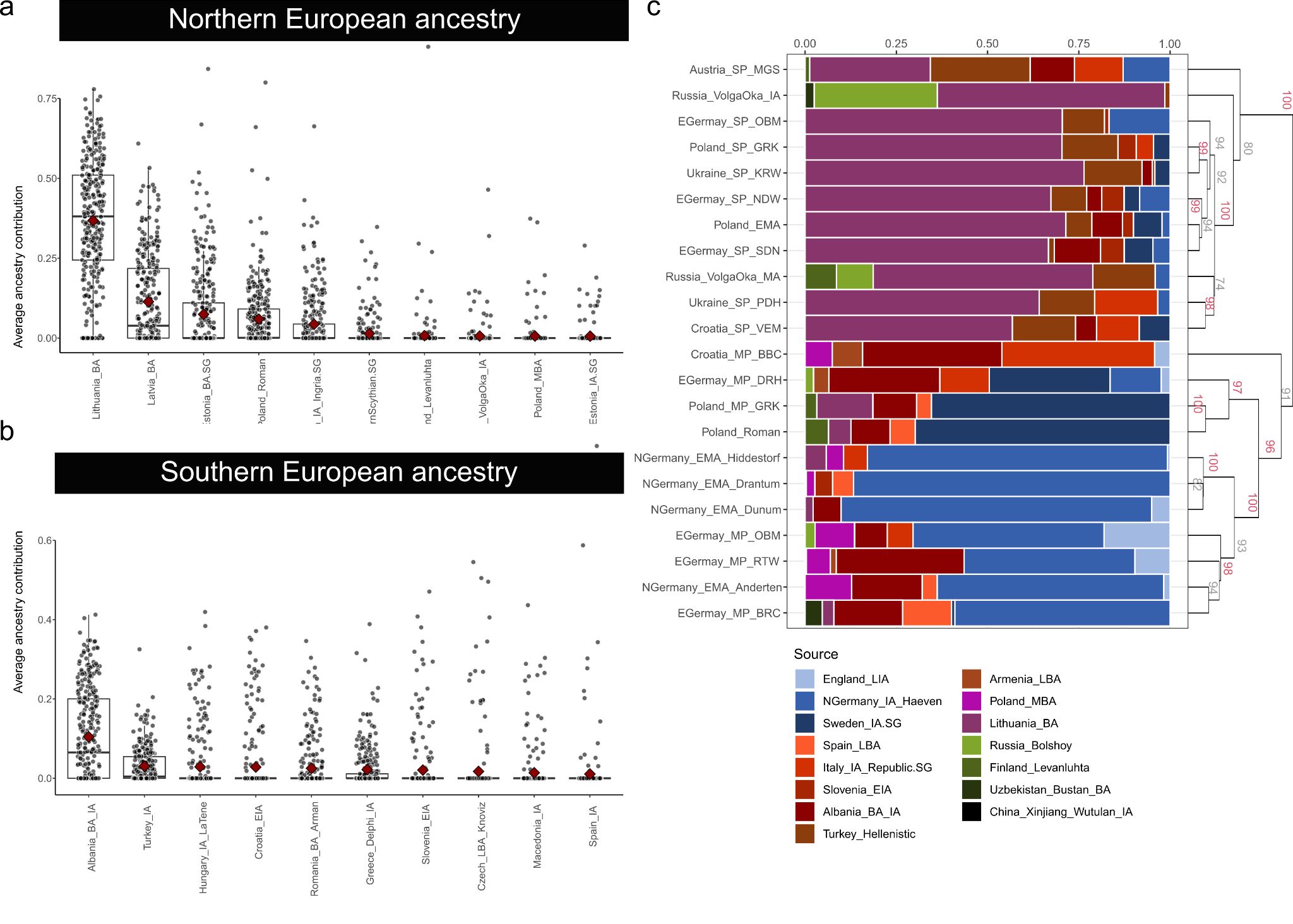
*

***Supp. Fig. 40. PCA inferred admixture contributions in SP individuals.*** *a) Individual-based results from PANE admixture modeling. For 300 SP individuals the contributions from 10 putative Northern European sources are depicted as boxplots. The mean per source population indicates a red diamond. b) Individual-based results from PANE admixture modeling. For 300 SP individuals the contributions from 10 putative Southern European sources are depicted as boxplots. The mean per source population indicates a red diamond. c) Group-based results from PANE admixture modeling.*


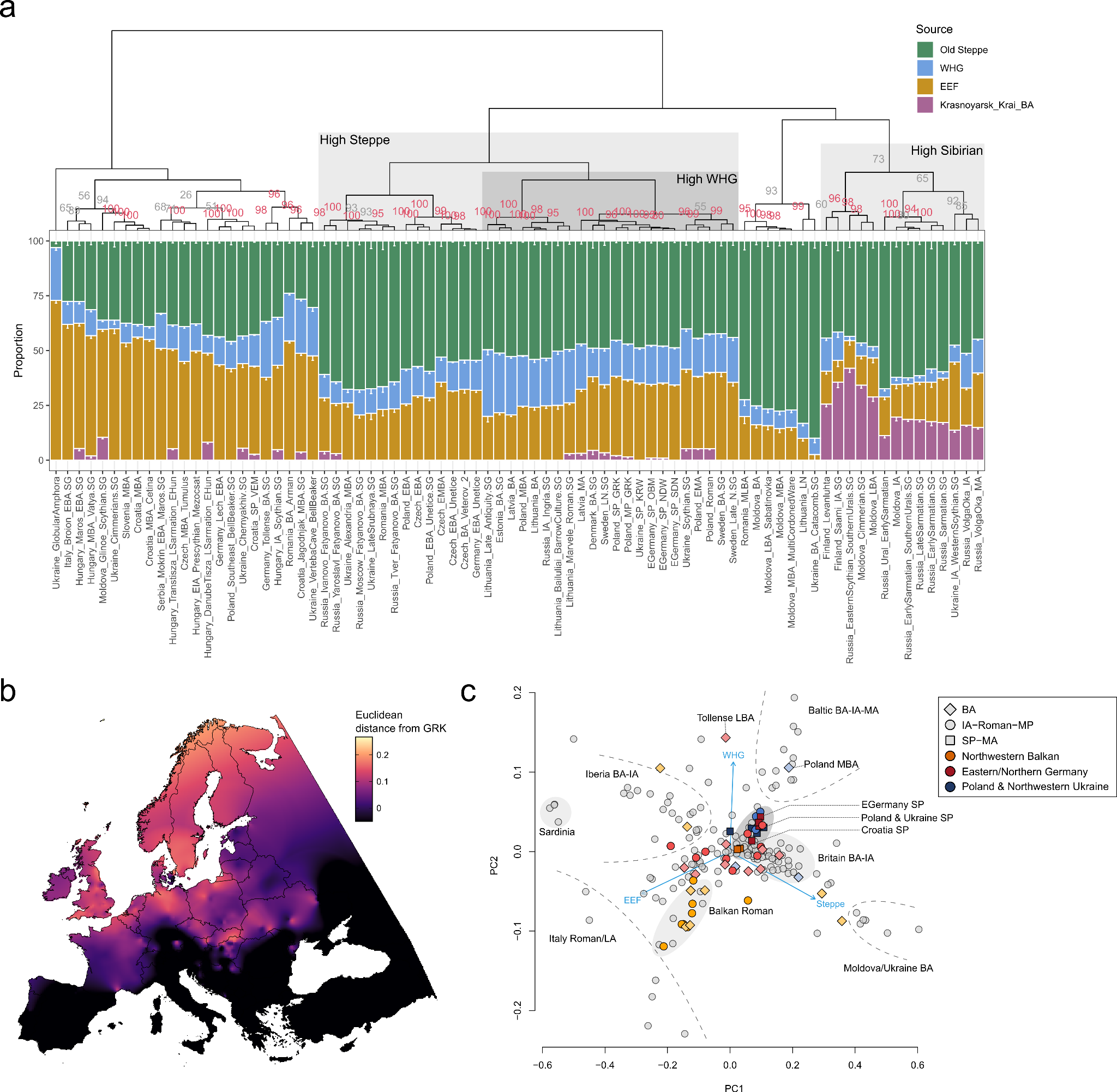


***Supp. Fig. 41. Deep ancestry composition of the SP gene pool.*** *a) Results from qpAdm analyses. A four-way model was fitted on 80 relevant ancient groups from Central-East and Northeastern Europe. Error bars indicate one standard error. Columns were ordered according to hierarchical cluster analysis applying Ward’s minimum variance method. The dendrogram and statistical support for the bifurcations from multiscale bootstrap resampling are shown. b) Kriging interpolation of euclidean distances between Gródek (n = 8) and post-Neolithic genomes from Europe (n = 6776) calculated on the individual results of a three-way qpAdm model (including WHG, EEF and Steppe as sources). c) PCA calculated on group-based results (n = 80) of the same three-way qpAdm model as in panel b).*

*
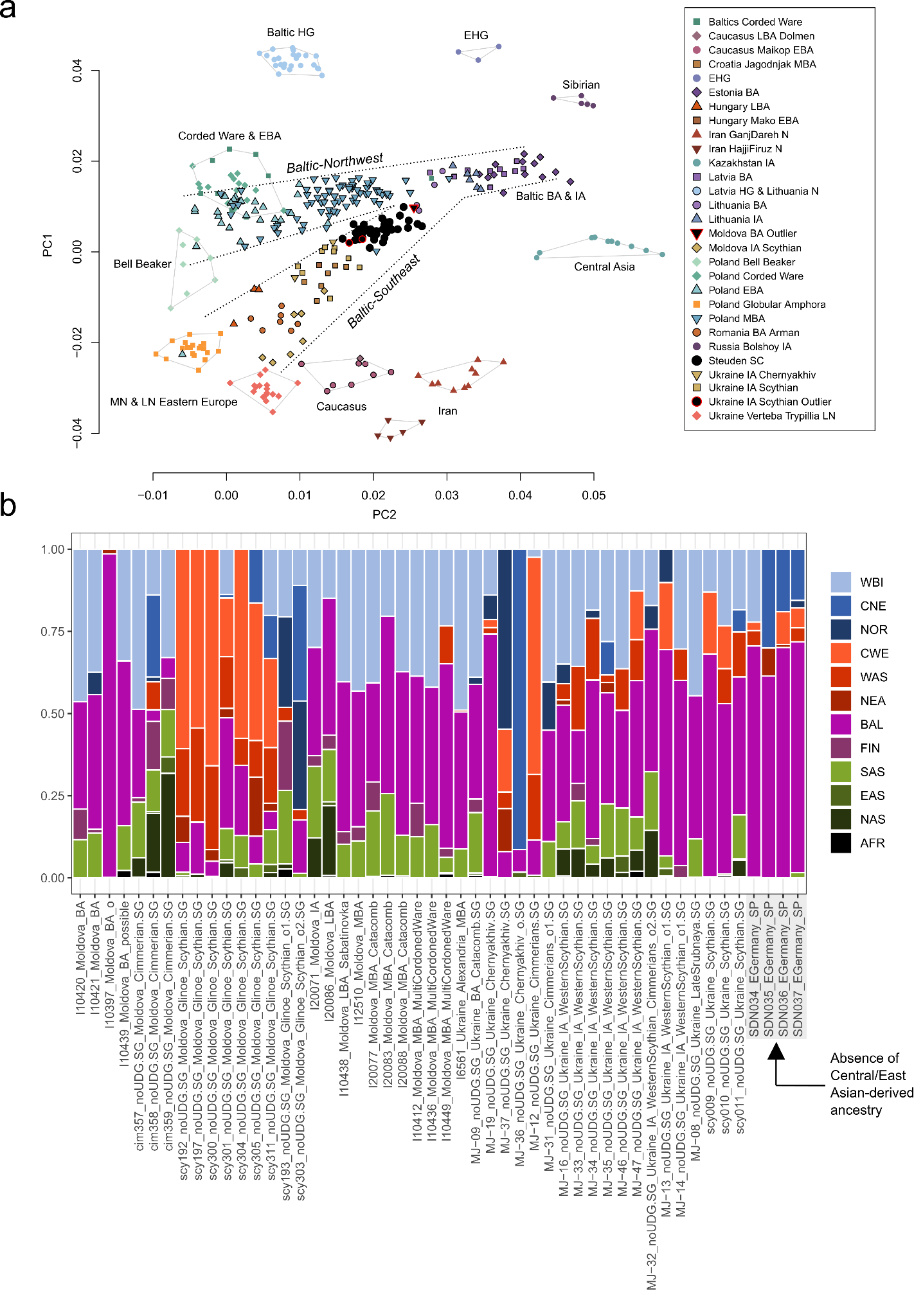
*

***Supp. Fig. 42. Population structure in East-Central and Eastern Europe during the last 5,000 years.*** *a) PCA computed using 10,528 present-day Europeans. Ancient genomes were projected onto the present-day variation. b) Individual results from ADMIXTURE analysis. Relevant ancient individuals from Eastern and Southeastern Europe (n = 50) were decomposed into 12 ancestral sources using a supervised clustering approach.*


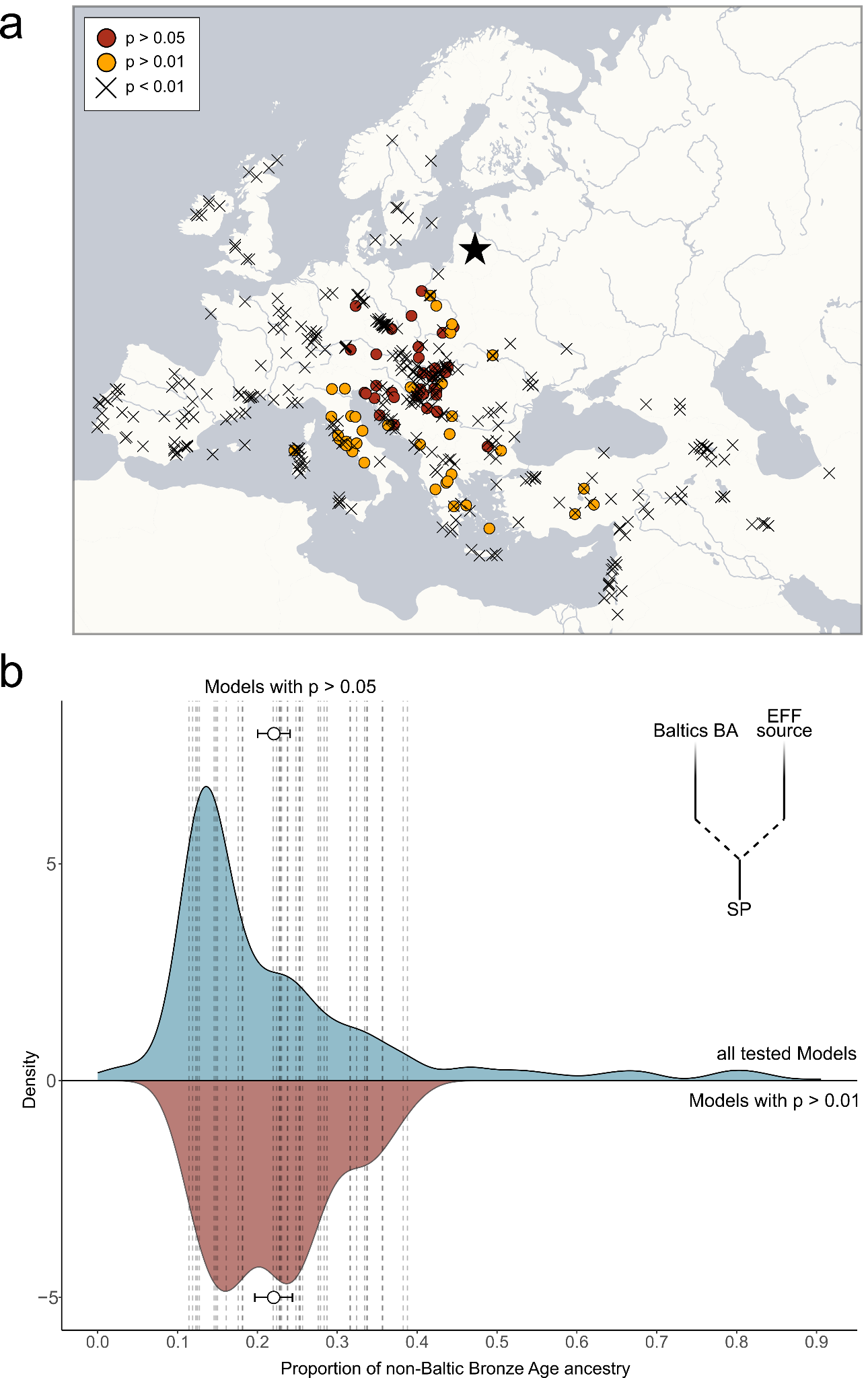


***Supp. Fig. 43. Formation of the SP gene pool****. a) Geographic distribution of ancient groups (n = 85) that constitute a fitting (p > 0.01) source in a two-way qpAdm model (using Baltics_BA (n = 14) as the other source) for the SP population from Eastern Germany (n = 240). b) Shown are the non-Baltics_BA component estimates obtained from the two-way qpAdm model as kernel density estimate (n = 473*)*. The distribution of all models (irrespective of their p-values) are shown in blue, the distribution of fitting models (with p > 0.01) in red (n = 85). The mean non-Baltics_BA estimate and respective standard error of the mean are indicated for both distributions.*


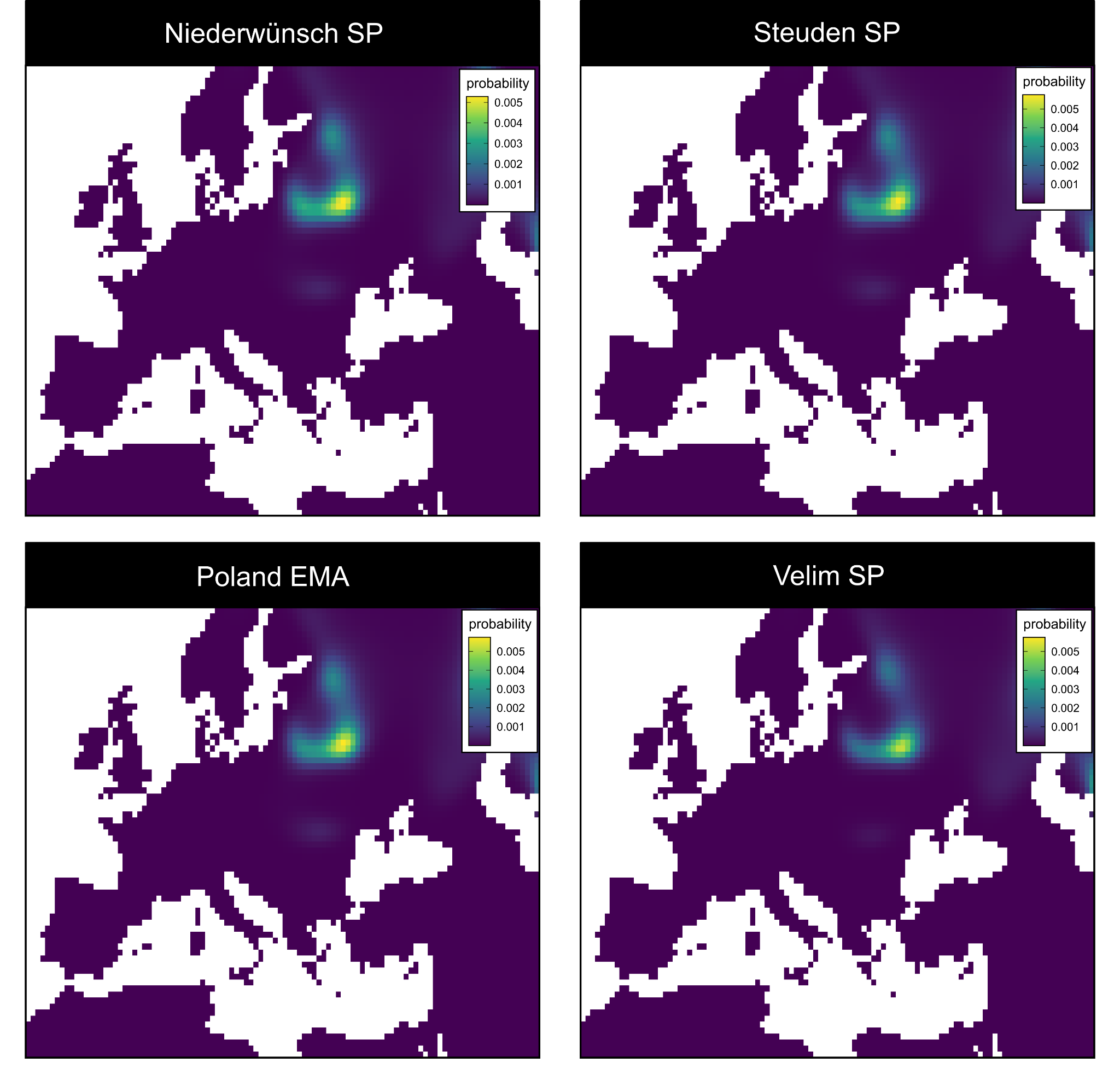


***Supp. Fig. 44. MOBEST predictions of the geographic regions where the ancestors of SP individuals in Central Europe originated from.*** *Shown are the averaged genetic similarity probability maps at search time 0 (CE) for 15 individuals from Niederwünsch (NDW), Steuden (SDN), Poland_EMA, and Velim (VEM), resp.*

#### 4.4.3 Possible admixture scenarios

As noted previously[^133^](https://paperpile.com/c/UPmHk7/MbA4g), the construction of ancestry models for Iron Age and medieval Europe in qpAdm is challenging, due to the limited statistical power offered by low sample sizes and reduced sequence quality of ancient genomes. This is especially related to the underlying *F*-statistics, which do not always have sufficient power to reconstruct events that involve closely related ancestries, even despite increasing sample sizes. Thus, the identification and qualitative assessment of complex admixture models with more than two sources remains difficult in qpAdm.

While other methods exist to detect and reconstruct fine-scale gene flow between closely-related sources[^133,145^](https://paperpile.com/c/UPmHk7/jyI9P+MbA4g), these methods are generally restricted to high-quality whole genome data suitable for imputation and diploid genotyping, a precondition that is currently only fulfilled by a minor fraction of the overall aDNA record. To utilize a larger panel of donors to model a given target admixed group, we again applied an NNLS approach to leverage the entire PC space[^141^](https://paperpile.com/c/UPmHk7/a8hm) or ADMIXTURE *Q* matrix obtained from analyses including large modern cohorts. For that, we selected Poland_EMA as target (representing a larger sample size than our novel data from Gródek) and iterated through all possible two- and three-way admixture model combinations involving the following set of 41 source groups:

Albania_BA_IA, Armenia_LBA, Bulgaria_EIA, Croatia_EIA, Czech_IA_Hallstatt, Czech_LBA_Knoviz, EGermany_Halle_IA, Estonia_BA.SG, Finland_Levanluhta, Germany_Hassleben_Germanic.SG, Hungary_EIA_Prescythian_Mezocsat, Hungary_IA_LaTene, Hungary_IA_Scythian.SG, Hungary_IA_Scythian_oAegean.SG, Latvia_BA, Lithuania_BA, Lithuania_Marvele_Roman.SG, Macedonia_IA, Moldova_Glinoe_Scythian.SG, NGermany_EMA_Hiddestorf, NGermany_IA_Haeven, Norway_IA.SG, Poland_MBA, Poland_Weklice_WielbarkCulture_Roman.SG, PolishCaves_IA, Romania_BA_Arman, Russia_Alan.SG, Russia_Bolshoy, Russia_EarlySarmatian_SouthernUrals.SG, Russia_EIA_Sargatka_IA, Russia_IA_Ingria.SG, Russia_LateSarmatian.SG, Russia_Sarmatian.SG, Russia_VolgaOka_IA, Slovakia_BytcaHrabove_Puchov_LaTene_Roman.SG, Slovakia_IA_Vekerzug, Slovakia_LIA_LaTene, Slovenia_EIA, Sweden_IA.SG, Ukraine_IA_WesternScythian.SG, Ukraine_Scythian.SG

Specifically, we tested 820 two-way (Fig. S45) and 10,660 three-way models (Fig. S46) to identify the best fitting constellations, selecting models that produce the lowest residuals (Table S49-50). We note that using output from unsupervised ADMIXTURE (at K=9) or the first 10 PCAs from our European PCA produces highly similar and significantly correlated residuals, indicating that PCA and ADMIXTURE detect the same underlying population genetic signal (Spearman's rank correlation; *r* = 0.9063601, *p* < 2.2e-16). For the two-way models using PCA output, we identify the following constellations as producing the lowest residuals (in increasing order):

- Lithuania_Marvele_Roman.SG + Moldova_Glinoe_Scythian.SG (0.000014200)
- Lithuania_Marvele_Roman.SG + Czech_IA_Hallstatt (0.000018900)
- Latvia_BA + PolishCaves_IA (0.000021100)
- Lithuania_Marvele_Roman.SG + EGermany_Halle_IA (0.000023600)
- Lithuania_Marvele_Roman.SG + PolishCaves_IA (0.000024500)

Using ADMIXTURE, we identify the following constellations as producing the lowest residuals (in increasing order):

- Russia_IA_Ingria.SG + Czech_IA_Hallstatt (0.01071026)
- Estonia_BA.SG + EGermany_Halle_IA (0.01250328)
- Russia_IA_Ingria.SG + Moldova_Glinoe_Scythian.SG (0.01382988)
- Russia_IA_Ingria.SG + Ukraine_Scythian.SG (0.01389348)
- Lithuania_Marvele_Roman.SG + EGermany_Halle_IA (0.01404399)

For the three-way models using PCA output, we identify the following constellations as producing the lowest residuals (in increasing order):

- Lithuania_Marvele_Roman.SG + Moldova_Glinoe_Scythian.SG + NGermany_IA_Haeven (2.48e-06)
- Lithuania_Marvele_Roman.SG + Moldova_Glinoe_Scythian.SG + NGermany_EMA_Hiddestorf (2.68e-06)
- Lithuania_Marvele_Roman.SG + Moldova_Glinoe_Scythian.SG + EGermany_IA_Halle (3.45e-06)
- Lithuania_Marvele_Roman.SG + Moldova_Glinoe_Scythian.SG + PolishCaves_IA (3.60e-06)
- Lithuania_Marvele_Roman.SG + Armenia_LBA + Hungary_IA_Scythian.SG (4.13e-06)

Using ADMIXTURE, we identify the following constellations as producing the lowest residuals (in increasing order):

- Estonia_BA.SG + Moldova_Glinoe_Scythian.SG + PolishCave_IA (0.001497854)
- Estonia_BA.SG + Hungary_IA_Scythian_oAegean.SG + PolishCave_IA (0.001792002)
- Estonia_BA.SG + Bulgaria_EIA + PolishCave_IA (0.001836536)
- Estonia_BA.SG + Slovakia_IA_Vekerzug + PolishCave_IA (0.001838154)
- Estonia_BA.SG + Macedonia_IA + PolishCave_IA (0.001900302)

Among the 500 3-way admixture models that produce the lowest residuals, Lithuania_Marvele_Roman.SG is involved as proxy for Baltic-related ancestry in 330 constellations, followed by Estonia_BA.SG in 98 models and Latvia_BA in 81 models. Interestingly, Poland_MBA is only featured in 25 cases, agreeing with previously published results[^133^](https://paperpile.com/c/UPmHk7/MbA4g) indicating a minor role of the Middle Bronze Age population of Poland in the formation of the SP gene pool. For the Southern European-enriched ancestry, Moldova_Glinoe_Scythian.SG is listed in 52 models, followed by Czech_IA_Hallstatt (49), Albania_BA_IA (40), Slovakia_IA_Vekerzug (40), Slovakia_BytcaHrabove_Puchov_LaTene_Roman.SG (33) and Ukraine_Scythian.SG (32). Finally, as proxy for Northwestern European ancestry, PolishCaves_IA is involved in 93 cases, EGermany_IA_Halle in 56 cases, NGermany_EMA_Hiddestorf in 43 cases and NGermany_IA_HVN in 28 cases. Poland_Weklice_WielbarkCulture_Roman.SG is only featured in 17 models, mirroring results for Norway_IA.SG (15) and Sweden_IA.SG (14) (Table S49-50).

Overall, these results are consistent with our previous results, indicating a majority of Baltic-derived ancestry (>50%), complemented by a major Southern European-derived ancestry component (>20%) and a minor Northwestern European-derived ancestry component (<20%) (on average 57%, 30% and 14% for the 10 best models using PCs). If the latter was acquired by all SP populations due to early contacts between the SP founding population and Germanic groups in East-Central Europe (e.g. in Poland and Ukraine) or did only enter groups that settled in regions previously inhabited by people of Northwestern European ancestry (e.g. in Eastern Germany, Poland, Czech Republic etc) due to acculturation and assimilation of the local population remains uncertain. Yet, we highlight that the earliest SP individuals from Gródek do not require such a Northwestern European component but instead feature minor proportions of Central Asian/Caucasus-related ancestry (<15%). Yet, again this signal might be related to demographic processes that postdate the initial expansion of SP groups.

Regarding the Southern European ancestry, we note that the preferred proxies are mostly located in Ukraine, Moldova, Hungary, Czech Republic, Slovakia and Romania, agreeing with our qpAdm modelling und suggesting a source from East-Central Europe bordering the southern extension of the Baltic gene pool for the formation of the SP founding population.

Interestingly, a recent article by Speidel and colleagues[^133^](https://paperpile.com/c/UPmHk7/MbA4g) identified using genome-wide genealogic tree inference a comparable fitting model for early medieval individuals from Poland involving Roman Iron Age samples from Lithuania, Hungarian Scythian/Slovakian La Tène Period samples as well as Sarmatians from the Caucasus. When reproducing this model using our PCA NNLS approach, we obtain highly similar admixture proportions (0.491377886 + 0.435619288 + 0.073002826 vs. 0.4841174 + 0.4244122 + 0.0914704, for the published and novel models respectively). However, the residual for their model is substantially worse (0.00001089943) compared to the residual identified for our best-fitting model (0.00000248), being placed on rank 48 of our 10,661 models.


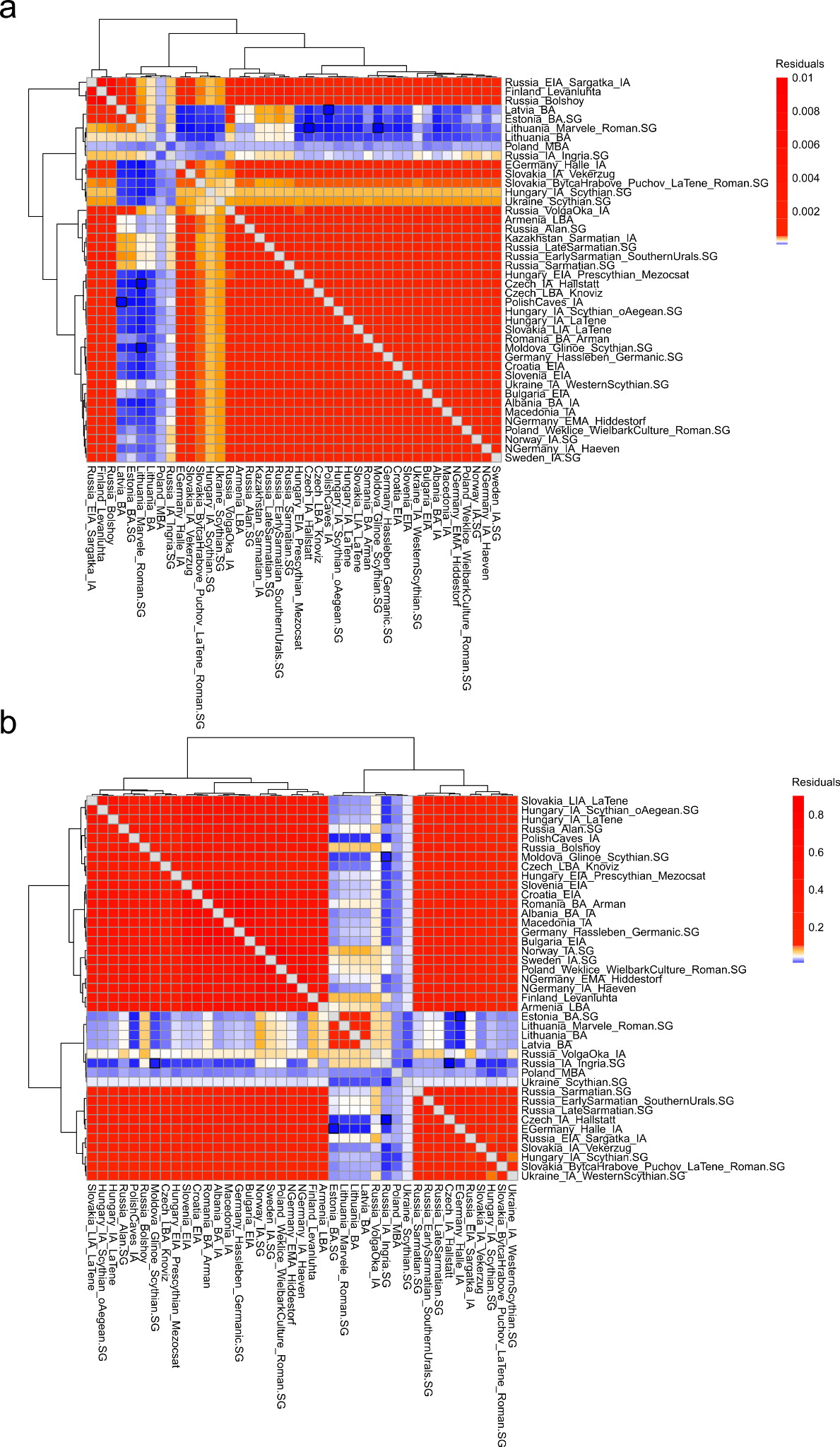


***Supp. Fig. 45. Residuals across 2-way models.*** *a) Shown are the residuals for two-way admixture models (involving pairs of 40 different groups) based on the top 10 PCs from the European PCA. The three models with the lowest residuals are highlighted. b) Shown are the residuals for two-way models based on the K = 9 unsupervised ADMIXTURE Q matrix. The three models with the lowest residuals are highlighted.*

###
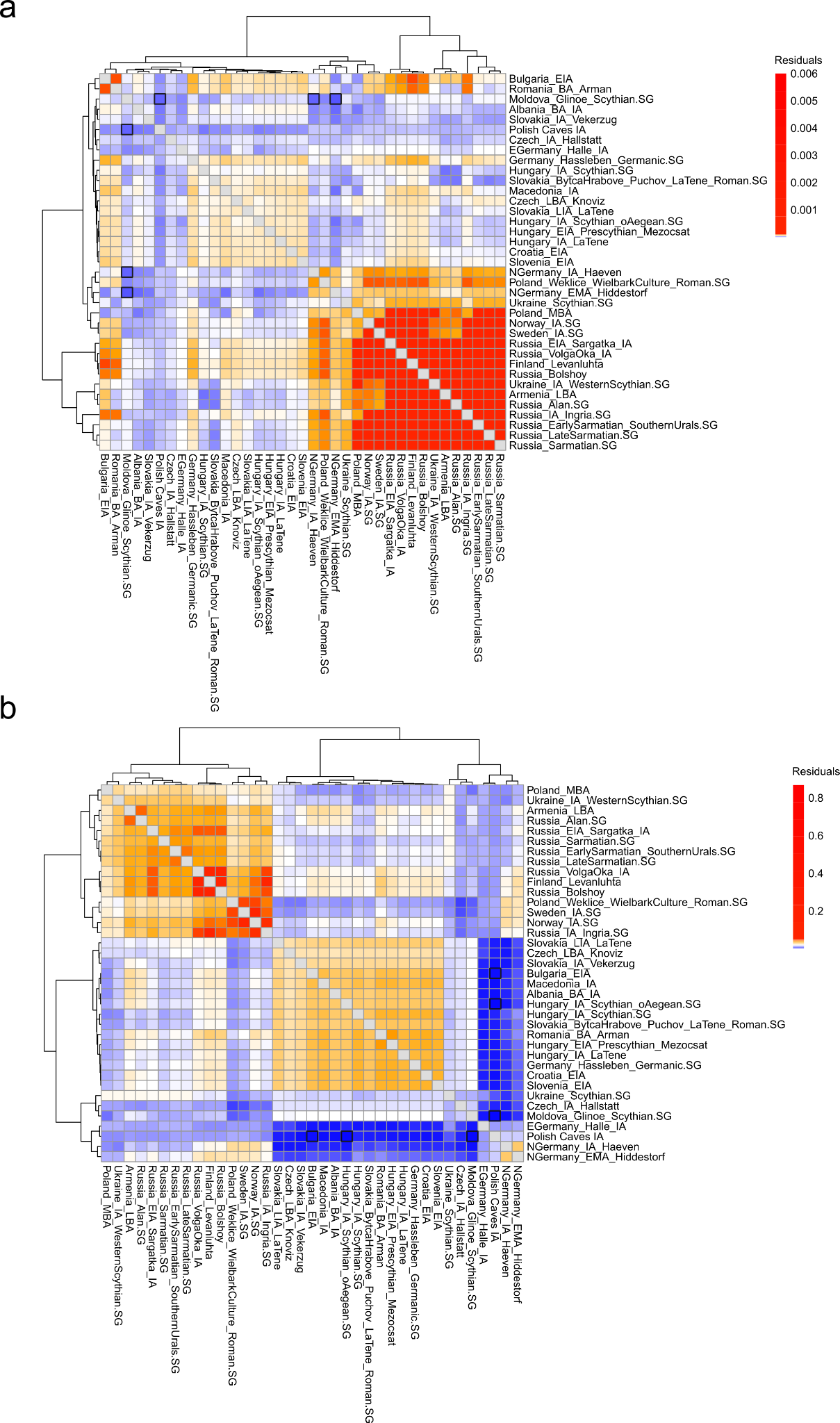


***Supp. Fig. 46. Residuals across 3-way models.*** *a) Shown are the residuals for three-way admixture models (involving pairs of 40 different groups) based on the top 10 PCs from the European PCA averaged across setups including Lithuania_BA (n = 4), Latvia_BA (n = 10), Estonia_BA.SG (n = 13) and Lithuania_Marvele_Roman.SG (n = 4). The three models with the lowest residuals are highlighted. b) Shown are the residuals for three-way models based on the K = 9 unsupervised ADMIXTURE Q matrix averaged across setups including Lithuania_BA, Latvia_BA, Estonia_BA.SG and Lithuania_Marvele_Roman.SG. The three models with the lowest residuals are highlighted.*

### 4.5 Extended Discussion I

In summary, we find that individuals across Europe (in Eastern Germany, the Northwestern Balkan, Poland-Northwestern Ukraine and the Russian Volga-Oka region) dating to the Slavic Period overwhelmingly show highest genetic affinities (measured using F_3_ and F_4_ statistics) to Bronze Age groups from the Baltics, namely from Lithuania, Latvia, Estonia and Ingria. Until today, this pattern is reflected in the present-day European gene pool with populations from Northeastern Europe (e.g. the Baltics, Poland, Ukraine, Belarus and Russia) being most closely related to our ancient SP individuals in F_ST_ and IBD analysis.

However, there are notable genetic differences between the Bronze Age populations of the Baltics and the SP individuals in Eastern Germany, Ukraine, Poland and Croatia. Specifically, the SP individuals exhibit lower fractions of WHG and higher fractions of EEF ancestry than Bronze Age Baltic individuals. For comparison, we measure varying fractions of WHG ancestry, ranging between 16% and 18%, e.g. α_WHG_ = 0.169 ± 0.004, α_WHG_ = 0.178 ± 0.005, α_WHG_ = 0.171 ± 0.005, α_WHG_ = 0.178 ± 0.005, α_WHG_ = 0.164 ± 0.005, α_WHG_ = 0.159 ± 0.006 and α_WHG_ = 0.174 ± 0.004 in Niederwünsch, Obermöllern, Steuden, Gródek, Korolivka/Korolówka, and Poland_EMA. Yet, in Iron Age and Bronze Age groups from the Baltics, these fractions are consistently higher, between 22% and 27%, e.g. α_WHG_ = 0.272 ± 0.008, α_WHG_ = 0.266 ± 0.008, α_WHG_ = 0.218 ± 0.013, α_WHG_ = 0.248 ± 0.015 and α_WHG_ = 0.243 ± 0.009 in Estonia_BA, Latvia_BA, Lithuania_BA, Lithuania_Bailuliai_BarrowCulture and Lithuania_Marvele_Roman.

Also, EEF fractions in Baltic Bronze Age groups are consistently smaller, ranging between 21% and 25%, e.g. α_EEF_ = 0.216 ± 0.009, α_EEF_ = 0.207 ± 0.008, α_EEF_ = 0.242 ± 0.014, α_EEF_ = 0.25 ± 0.017, α_EEF_ = 0.232 ± 0.009 in Estonia_BA, Latvia_BA, Lithuania_BA, Lithuania_Bailuliai_BarrowCulture and Lithuania_Marvele_Roman, respectively. Conversely, EEF values are higher in the SP genomes, ranging between 33% and 36%, e.g. α_EEF_ = 0.343± 0.005, α_EEF_ = 0.335 ± 0.006, α_EEF_ = 0.343 ± 0.005, α_EEF_ = 0.362 ± 0.006, α_EEF_ = 0.352 ± 0.007, α_EEF_ = 0.33 ± 0.004 in Niederwünsch, Obermöllern, Steuden, Gródek, Korolivka/Korolówka, and Poland_EMA.

Consequently, to explain this excess of EEF ancestry and deficiency of WHG ancestry in the SP gene pool, we must assume that additional Southern European-related ancestry was introduced to the SP gene pool during its formation. This would be congruent with the presence of considerable Western and Southern European-related CWE, WAS, WBI, and CNE ancestry proportions in ADMIXTURE analysis across all SP groups, which are on the other hand (nearly) absent in all tested Baltic Bronze and Iron Age populations. Using qpAdm and NNLS admixture modelling, we identify various possible Late Neolithic, Bronze and Iron Age proxies for the donor population of this additional Southern European ancestry, with most of these fitting proxies being located in East-Central and Southeastern Europe (e.g. Hungary, Poland, Ukraine, Moldova, Croatia, Romania, and Bulgaria). However, none of these groups might be the true donor of the EEF-enriched Southern European ancestry in the SP gene pool. Instead, we only consider them fitting proxies in the absence of (so far) unsampled populations from Late Neolithic/Bronze Age Poland, Moldova and Ukraine. Nevertheless, across all qpAdm models, we infer a mean contribution of 29% (95 CI: 24% - 33.5%) EEF-enriched Southern European ancestry to the mostly Baltic Bronze Age-derived SP gene pool.

Due to the lack of samples from Western Russia, Belarus and Northern Ukraine during the Middle and Late Bronze Age, we are not able to conclusively infer if this admixture process occurred in a single pulse or gradually over a prolonged period of time. Applying DATES, we calculate mean dates of this admixture event in SP individuals from Eastern Germany at 972 ± 250 BCE and in SP individuals from Poland at 906 ± 562 BCE (**Date 1**) (Fig. S47b). In this period, the expanding Baltic-related gene pool might have encountered and interacted with neighboring, EEF-enriched populations in the South. Individuals associated with the Trzciniec culture in Central and Eastern Poland (c. 2400-1300 BCE) display a substantial increase of WHG ancestry, which also emerges in Northeastern Germany (Tollense), Hungary (Halva, Fuzesabony & Vatya) and Croatia (Jagodnjak); this might indicate a general expansion of WHG-enriched groups from the Baltics to the West and South during this time.

How can we situate these dates in the chronological frames of other disciplines? These movements may ultimately have caused the divergence of the Balto-Slavic continuum and the formation of Baltic and Slavic language areas in the North and South, respectively. With phylogenetic linguistic methods, the divergence time of the Slavic and Baltic languages was calculated at a median time of 1660 BCE (95% HPD 3040 – 530 BCE)[^146^](https://paperpile.com/c/UPmHk7/emO5R). Archaeologically, the Late Bronze Age cultures in the areas where this process of admixture seems to have taken place were followed by the Milograd Culture (c. 7th c. BCE-1st c. CE) in Belarus and Northern Ukraine, which was later gradually replaced by the Zarubintsy culture (c. 3rd c. BCE - 1st c. CE). From the Zarubintsy culture, the so-called Kyivan culture (also known as the post-Zarubintsy culture during its early phase) developed during the 2^nd^ century CE in the same area, which is the first material culture featuring similarities with those early medieval cultural phenomena that can be surely associated with early Slavs.

Indeed, the geographical extent of the Milograd, Zarubintsy and Kyivan cultures in Northern Ukraine, Belarus and Western Russia agrees well with our aDNA evidence. Using MOBEST, we infer a region spanning the South of Belarus and North of Ukraine along the Dnjepr river in present-day Polesia as the best spatial proxy for the origin of the SP individuals in our three study transects. Such an area is further consistent with the archaeological consensus which locates the formation of the Slavs as an ethnic group in the southern area of the forest zone, mostly in the upper Dnjepr basin. Besides MOBEST, we highlight four other lines of evidence in support of our hypothesis (Fig. S47a):

(**A**) Both ancient and present-day populations from Lithuania and Latvia show the highest genetic similarity to SP individuals in Croatia, Eastern Germany, and Poland-Northwestern Ukraine (as measured via *F*-statistics (e.g. F_3_, F_4_, F_ST_) and IBD sharing). Specifically Lithuania_Marvele_Roman, Lithuania_BA and Latvia_BA appear as the best ancient proxies for the Baltic-related ancestry in SP individuals. Consequently, the SP gene pool must have formed in close vicinity of present-day Lithuania and Latvia (although, we emphasize that the Baltic gene pool may have extended significantly further south during the Late Bronze Age and Iron Age; Consequently, better (currently unsampled) sources of Baltic-related ancestry might be found in present-day Belarus and Northern Ukraine).

However, Bronze and Iron Age populations from further west cannot be considered plausible proxies. While SP individuals show excess affinity to MBA genomes from Southeastern Poland, this affinity is significantly less pronounced than their affinity to Lithuania_BA and Latvia_BA. Furthermore, during the Roman Iron Age, we find that the overwhelming majority of individuals from present-day Poland exhibited Northwestern European, Scandinavian-related ancestry (predominantly CNE and NOR ancestry) excluding the possibility that the SP gene pool persisted there over the Iron Age. This is especially noteworthy since the distribution of this Scandinavian ancestry extended from (present-day) Central Poland to the Polish-Ukrainian border (and potentially even beyond), highlighting the necessity that the SP gene pool was during that time located further east (at least east of the Vistula River).

(**B**) Neighboring ancient (e.g. Estonia_BA, Russia_Ingria_IA) and present-day populations (Estonia, Ingria, Karelia) to the Northeast are less related to SP individuals than populations from the West, namely Lithuanians, Latvians, and Belarussians. Furthermore, Bronze and Iron Age populations from Estonia, Ingria and the Volga-Oka area show too high proportions of FIN-related ancestry in ADMIXTURE analysis (>10%), a component virtually absent in SP individuals from Croatia, Eastern Germany, Poland and Ukraine (<2%). Thus, the formation of the SP gene pool must have occurred in closer vicinity of the Western than of the Eastern Baltics.

(**C**) Populations to the East and Southeast do not show excess affinity to our SP individuals and exhibit too high proportions of Steppe and/or Siberian-related ancestry to have formed the SP gene pool. Specifically, MBA, MLBA and Sarmatian period individuals from Russia, Moldova and Ukraine feature substantially larger Steppe (>60%) but lower WHG (<10%) ancestry components than the SP gene pool. Furthermore, many Iron Age Scythians from Central Ukraine, Cimmerian individuals from Moldova and Sarmatians genomes from Russia show additional Siberian-related (>10%) ancestry, which is mostly absent in SP individuals. This is also supported by ADMIXTURE analysis highlighting the absence of Central and/or East Asian-related SAS, EAS or NAS ancestry in the SP gene pools of Eastern Germany, Poland, Ukraine and Croatia. Consequently, mainly Central Asia/Steppe-derived populations from Moldova, Southern/Eastern Ukraine, West-Central Russia and the Caucasus cannot have contributed substantially to the formation of the SP gene pool. However, we note that the majority of Iron Age (e.g. Scythian) individuals from Moldova and Western Ukraine exhibit only minor fractions of Central Asian-related ancestry (<12%) and feature predominantly Southern European ancestry. Consequently, they might represent (also geographically) plausible source populations as indicated by NNSL admixture modelling.

(**D**) SP individuals are enriched in EEF- (>33%) and depleted in WHG (<18%) ancestry compared to ancient/modern populations in the Baltics (<25% and >21%), indicating admixture with EEF-enriched sources from the South. This is further supported by the presence of additional CWE and WAS ancestry in all SP groups. Thus, the formation of the SP gene pool cannot have occurred directly within the Baltics but further to the South. Multiple Late Neolithic/Bronze Age populations in East-Central and Southeastern Europe constitute working proxies for this donor gene pool. Yet geographically, Late Neolithic and Bronze Age sourcers from Eastern Poland, Western Ukraine and the Northern Carpathian mountains must be considered the most plausible proxies. Consequently, a formation area between the Baltics and the Northern Carpathian mountains seems most plausible.

We conclude that the formation of the SP gene pool must have occurred east of the Vistula river and south of the Baltics (specifically Latvia and Lithuania), yet, north of the Carpathians. Based on our different genetic analyses, we suggest a potential original area in present-day Western, Central and Northern Ukraine as well as (potentially) Southern Belarus. From there, the SP gene pool expanded, west-, south- and eastwards and encountered neighboring Indo-European- as well as non-Indo-European populations, which contributed additional ancestry to the SP gene pool in the different regions. This admixture, which can be genetically dated, can attest SP presence in the absence of aDNA evidence and consequently provide a rough approximation for the timing of the Slavic movements into the different parts of Europe. Specifically, we date the admixture between the SP and local gene pool in the Russian Volga-Oka region to 318 ± 174 CE (for Russia_VolgaOka_MA) and 722 ± 272 CE (for Russia_VolgaOka_H) (**Date 3**), respectively, and in Pannonia and the Northwest Balkans to 96 ± 129 (for Mödling, Austria) and 170 ± 120 CE (for Velim, Croatia) (**Date 2**) (Fig. S47b).

These approximate admixture dates trace the geographical dispersal of the SP gene pool over time. At the other end of the chronological range in which the Slavic expansion can be placed, our aDNA data and our new radiocarbon dates from Velim and Gródek suggest that until the 9^th^ century CE, SP groups already reached the westernmost regions of Croatia and other limits of Slavic settlement, including the the Volga river in the east. The written sources date Slavic expansion into the Balkans and eastern Central Europe to the 6^th^ and 7^th^ centuries, into the Northwestern Balkan rather precisely to around 600, and do not give any dates for Slavic expansion along the Volga. It appears unlikely that groups separated by more than 2,000 km still continued to preserve the same language over a prolonged period of time, so these movements ultimately resulted in the decrease of both genetic and linguistic cohesion and facilitated the divergence of the Slavic continuum. Thus, the split of East/West- and South Slavic languages should either fall within this time interval or slightly postdate it. Bayesian analysis suggests a divergence of East-West Slavic and South Slavic languages at around a median time of 510 CE (95% HPD 160–780 CE)[^146^](https://paperpile.com/c/UPmHk7/emO5R). This approximate range overlaps with the genetic dates for the admixture of the SP gene pool with local groups in the present-day East (the Volga-Oka region) and South Slavic (Croatia) language areas, potentially reflecting the geographical spread of SP ancestry and a co-dispersal of language and Eastern European-derived groups. From historical and historical-linguistic data, we would expect the development of specific Slavic languages towards the end of the Bayesian date range or after it. However, we highlight that this represents a generalized model that will most likely not accurately reflect the more intricate patterns of language spread, adoption, and diversification in medieval Central, Eastern and Southeastern Europe.

However, we caution against taking DATES estimates at face value. Several factors affect the informative value of these estimates:

- DATES as well as many other genetic admixture dating tools tends to provide slightly inflated date estimates for younger admixture events, especially in combination with a suboptimal choice of source population surrogates. The choice of Italy_Imperial.SG (in absence of any appropriate representative for the Roman Period population of Pannonia/Northern Balkan) is potentially connected to this issue.
- DATES can only identify and date a singular admixture event. However, most human populations are the product of a series of consecutive admixture events, often involving very similar source groups. In our case, we highlight that the SP gene pool is already a mixture between a Northeastern and Southeastern European source, which might inflate date estimates for later admixture processes that involve SP individuals and groups from the Balkan Peninsula.
- DATES estimates are point estimates that are associated with standard errors. It is thus necessary to consider all potential estimates within the 95% confidence as reasonably valid. Yet, in most cases the 95% CI spans more than ~400 years. While such ranges might be adequate for questions regarding the older prehistory of Europe, much more precise dates for migration events are available from historic sources and the archaeological record during the Late Antiquity and Early Middle Ages.

Overall, the observed shift from local to Eastern European-derived ancestry in the individuals pre- and post-dating the transition from the MP to the SP provides a much better temporal constraint for the arrival of SP ancestry than the DATES estimates. Nevertheless, the ranges of our DATES estimates are well consistent with the archaeologically and historically proposed timeframes for the arrival of Slavic groups in Southeastern and Eastern Europe.


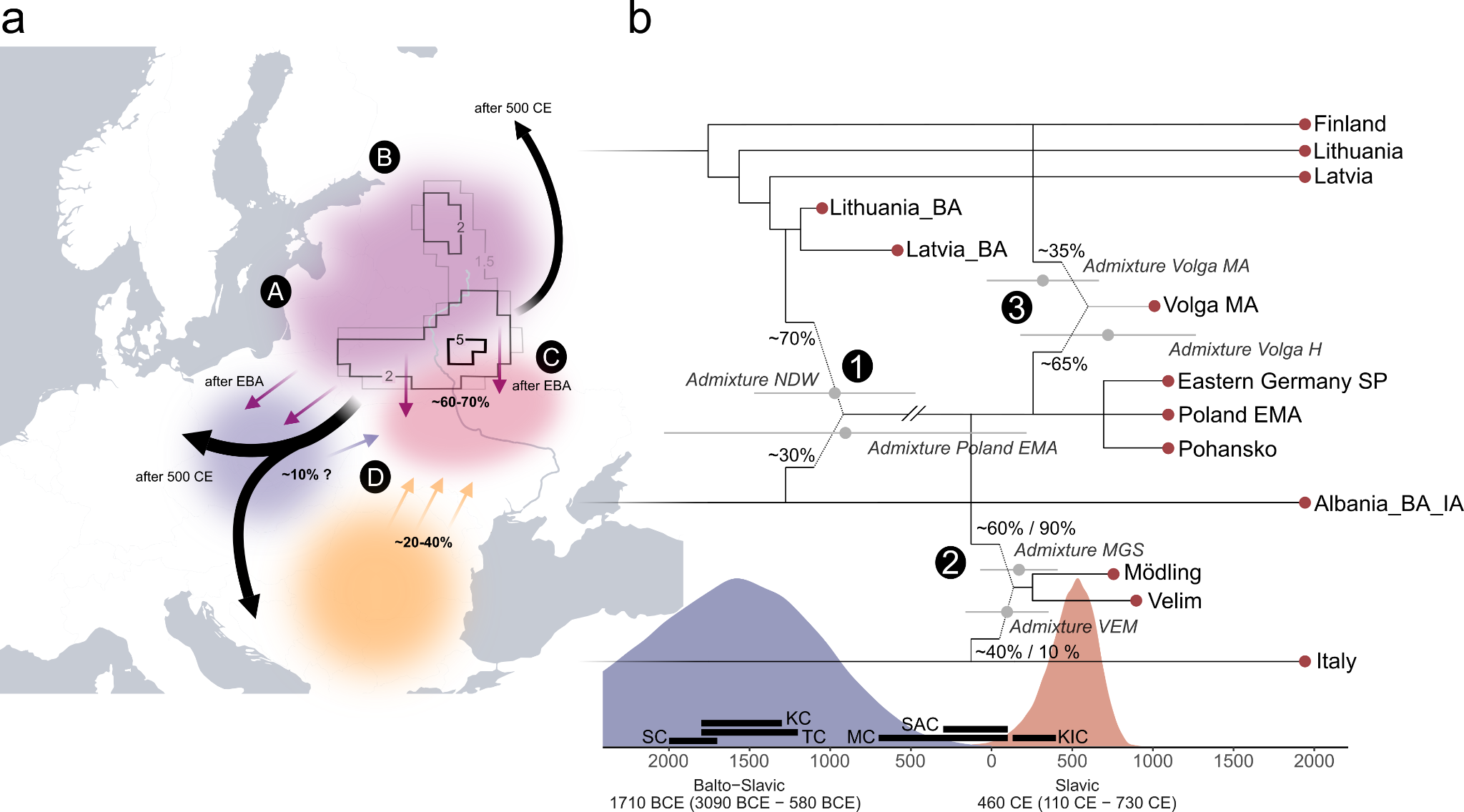


***Supp. Fig. 47. Temporal and geographical overview of the formation and spread of the SP gene pool****. a) Schematic overview of the geographic origin and formation process of the SP gene pool in Northeast Europe. The contours indicate the averaged maximum probability at search time 0 CE (multiplied by 100) for 20 high-coverage individuals from Niederwünsch (denoting the mean prediction of the geographic regions where the ancestors of these individuals originated) as inferred using MOBEST. We combine four major lines of evidence to support the MOBEST estimate: (A) Both ancient and present-day populations from Northeast Europe and the Baltics show the highest genetic similarity to SP individuals; (B) Ancient individuals from Estonia, Ingria and Karelia are less related to SP individuals than groups from the Lithuania and Latvia; (C) Populations in eastern Ukraine and western Russia feature too high proportions of Steppe and/or Siberian ancestry to have formed the SP gene pool; (D) SP individuals are enriched in EEF- and depleted in WHG ancestry compared to ancient/modern populations in the Baltics, indicating admixture with a EEF-enriched source south of the Baltics. b) The maximum likelihood skeleton tree of ancient and present-day populations was constructed using TreeMix. Dates for the admixture events were inferred using DATES, ancestry proportions using qpAdm. Error bars indicate two standard errors. The probability densities for the split of the Balto-Slavic and Slavic (East/West-South Slavic) languages are indicated as well as the simplified temporal ranges of relevant archaeological cultures in East Europe. The abbreviations are in chronological order: SP – Strzyżów Culture, KC – Komarów Culture, TC – Trzciniec Culture, MC – Milograd Culture, SAC – Zarubintsy Culture, KIC – Kyivan Culture. Relevant admixture dates mentioned in the text are indicated using numbers: (1) Admixture between WHG- and EFF-enriched sources measured in EGermany_SP and Poland_EMA; (2) Admixture between SP- and Italy_Imperial-related sources measured in Mödling, Austria, and Velim, Croatia; (3) Admixture between SP and Iron Age-related sources in the Russian Volga-Oka region.*

## 5. Present-day Europe

We measured the impact of the Slavic expansion on the gene pool of present-day Europe. For this, we selected 48 populations with excess BAL-ancestry in Southeastern, Central and Eastern Europe and applied qpAdm to calculate estimates of the mixture coefficient α_SP_ (represented by 7 individuals from Gródek (GRK), dating between the 7^th^ and 9^th^ century CE) in the following 851 two- and three-way admixture models:

1. Populations from the Baltics (e.g. Latvians, Lithuanians, and Estonians) were modeled using a two-way admixture model including GRK and either Lithuania_Marvele_Roman.SG, Latvia_BA, Estonia_BA or Estonia_IA.
2. Populations from Northeastern Europe (e.g. Ingrians, Karelians, Mordovians and Chuvash) were modeled using a two-way admixture model including GRK and either Finland_Levanluhta or Russia_Bolshoy.
3. Populations from the Southeast (e.g. Balkan Peninsula, Gagauz, Hungarians etc) were modeled using a three-way admixture model including GRK and a) either Balkan_Roman, Italy_IA_Republic_oEasternMediterranean, Italy_Imperial, Turkey_IA, Turkey_Hellenistic, or Turkey_WestByzantine and b) either Slovenia_EIA, Croatia_EIA, Hungary_IA_LaTene, Macedonia_IA, Albania_BA_IA or Bulgaria_EIA.
4. Populations from Central and East-Central Europe (e.g. Poles, Slovaks, Germans, Sorbs and Czechs) were modeled using a two-way admixture model including GRK and either Germany_EarlyMedieval, NGermany_EMA, Denmark_EarlyViking, Norway_IA.SG, Estonia_EarlyViking, Poland_Weklice_WielbarkCulture_Roman or Poland_Roman.

We then selected all 152 models producing fitting *p*-values (> 0.01) to assess the degree of variation in SP ancestry depending on the populations used as proxies for the local pre-SP ancestry (Ex. Fig. 6, Table S41).

We find that SP ancestry is today most common in East and West Slavic-speaking populations of Eastern Europe, reaching 100% in present-day Ukraine as well as comparably high fractions in Belarus, Poland and the western parts of Russia close to the Belarussian and Ukrainian borders (e.g. Smolensk and Belgorod). Similar to patterns of BAL ancestry, IBD sharing and F_ST_ distances, SP-related ancestry then decreases radially, with proportions ranging between 95% and 75% in Western and Central Russians, Slovaks and the (Slavic-speaking) Eastern German Sorbs. On the Balkan Peninsula, the SP component follows a North-to-South cline, representing 67% to 69% of the ancestry in Bosnians, Croatians, and Slovenians, 40% to 56% of the ancestry in Serbs, North Macedonians, Bulgarians, Montenegrians, (Romance-speaking) Romanians, (Romance-speaking) Moldavians and (Turkic-speaking) Gagauz, as well as 17% to 34% of the ancestry in Greeks, Cretans, Albanians and Kosovars (with slightly higher proportions in Greeks from Thessaloniki compared to Greeks from Athens). Along the Baltic coast in the North, we measure a substantially lower fraction of SP ancestry in Baltic- and Finno-Ugric-speaking groups, varying between 31% and 39% in Lithuanians, Latvians, Estonians, Ingrians and Karelians. Similarly, SP ancestry is reduced in populations from East-Central Europe to the West (e.g. (Germanic-speaking) Eastern Germans: 40%, (West Slavic-speaking) Czech 64% and (Finno-Ugric-speaking) Hungarians 67%), reflecting extensive admixture with the preceding local populations and subsequent immigration waves from the West, most likely from Germanic-speaking Central and Northwestern Europe.

As mentioned above, our estimates of genome-wide SP ancestry (approximated here by Gródek individuals from Eastern Poland) are well correlated with other measurements of affinity to the Northeastern European gene pool (Fig. S48a). For example, our qpAdm estimates are significantly correlated with both BAL ancestry (as inferred using supervised ADMIXTURE) (*r* = 0.647 and *p* = 9.052e-07) and IBD sharing with the early medieval GRK population (*r* = 0.696 and *p* = 5.566e-08). However, we note that this signal is most likely an underestimation of the actual correlation between these three measurements. This bias is mostly driven by the estimates of SP ancestry in the Baltic populations (Lithuanians, Latvians, Estonians, Ingrians and Karelians). These groups show lower fractions of SP ancestry since most of their ancestry is modeled by Baltic Bronze and Iron Age sources closely related to the Baltic ancestors of our SP individuals. Thus, groups from the Baltics show higher IBD sharing with the GRK population and higher amounts of BAL ancestry than what would be predicted based on their qpAdm SP ancestry estimates. Consequently, when removing these five populations the coefficients of correlation substantially increase (*r* > 0.91, *p* < 2.2e-16 for all 3 comparisons respectively) (Fig. S48b). Summarizing, the strong association between all three independent measurements of affinity indicates that our qpAdm models are able to retrieve precise estimates of the percentage of SP ancestry in present-day European populations and that these ancestries are robust in regard to the used ancient proxy sources. Consequently, our novel estimates for several present-day groups from the Balkan Peninsula are highly similar to and significantly correlated with previous results from smaller-scale studies[^113^](https://paperpile.com/c/UPmHk7/cvmfh) (Pearson's product-moment correlation; *r* = 0.9355021, *t* = 6.4856, *df* = 6, *p* = 0.0006387) (Fig. 52c).

Other measures of affinity to Northeastern European groups provide congruent evidence for a clinal distribution of SP ancestry in Europe. In groups from the Balkan Peninsula, estimates of BAL ancestry (from supervised ADMIXTURE), *F*-statistics of the form F_4_(CHB, Test; Italy, Poland) and mean euclidean distances from GRK individuals on PCA follow a North-to-South gradient, with higher affinities to Eastern Europe in the North and lower affinities in the South (Fig. S49-51). All three measurements are highly correlated (Fig. S52a) and, in case of F_4_-statistics, highly consistent with results obtained using ancient proxies (by replacing Poland with a pooled group of SP individuals from Eastern Germany, Poland, and Ukraine and Italy with a pooled group of IA, Roman, and EMA individuals from Turkey, respectively) (Pearson's product-moment correlation; *r* = 0.998306, *t* = 66.455, *df* = 15, *p* < 2.2e-16) (Fig. 52b). However, we observed comparable clines of gradually decreasing affinity to Eastern European-derived ancestry in Poland, the Czech Republic and Germany as well as in Western Russia.

For the Balkan Peninsula, our estimate of change in genome-wide ancestry is furthermore supported by independent evidence for population turnover from uniparental markers. As described in the Main Text as well as Supplementary Note Uniparental markers and sex bias, genome-wide SP ancestry is strongly associated with Y-chromosome haplotypes I2a and R1a. Specifically, R1a frequencies follow a North-to-South gradient that mirrors the gradient of affinity to West Asian and Northeastern European populations (Fig. S53), ranging from 6% to 8% in Turkey, Greece, and Kosovo in the South to 21% and 29% in Slovenia and Croatia in the North. We show that the relationship between GRK-related SP ancestry and fraction of R1a haplogroups in male individuals from the Balkan Peninsula and northern neighboring areas can be well modeled as a two-stage linear process (Regression Model with Segmented Relationship; adjusted *r*^2^ = 0.6462, *p* = 0.00213) (Fig. S53b), suggesting that R1a frequencies increase together with genome-wide SP ancestry towards the North, yet SP ancestry reaches a plateau at the border to East-Central Europe (e.g. in Hungary, Slovenia, Croatia and Moldova) while R1a haplotype frequency increases in northeastern direction (reaching 46% in Slovakia). The contrast of relatively high fractions of SP ancestry (67% and 69%) and comparably lower frequencies of R1a haplotype in Slovenia and Croatia (21% and 29%) is consistent with our observations of the haplotype diversity in the Slavic Period population of Velim, Croatia, which implies that the paternal gene pool of the Slavic populations expanding across the Balkan Peninsula was already more diversified than the paternal gene pool of closely related Slavic groups in Central-East Europe, where R1a frequencies are today still substantially higher, ranging between 38% (in Czechs) and 55% (in Belarussians).


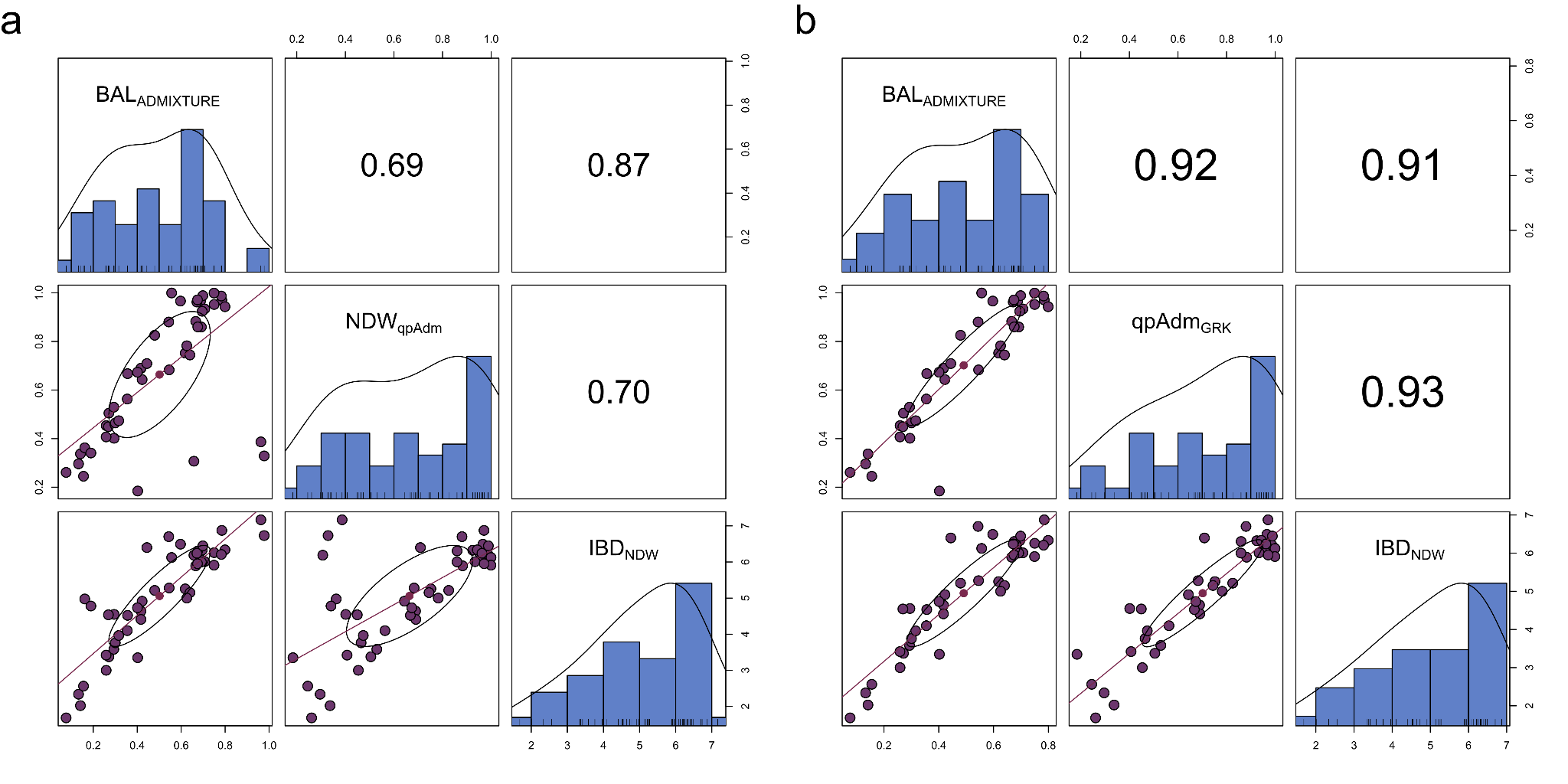


***Supp. Fig. 48. Similarity in supervised ADMIXTURE, qpAdm and IBD sharing results across present-day populations.*** *a) Correlogram illustrating pairwise correlations between BAL ancestry (from supervised ADMIXTURE), NDW-related ancestry (from qpAdm), and IBD sharing (with NDW) for present-day European populations (n = 47). Spearman's r for each pairwise correlation test are indicated in the upper triangle of the matrix. b) Correlogram for the same data after removing Baltic populations (Lithuanian, Latvian, Estonian, Ingrian, and Karelian).*

*
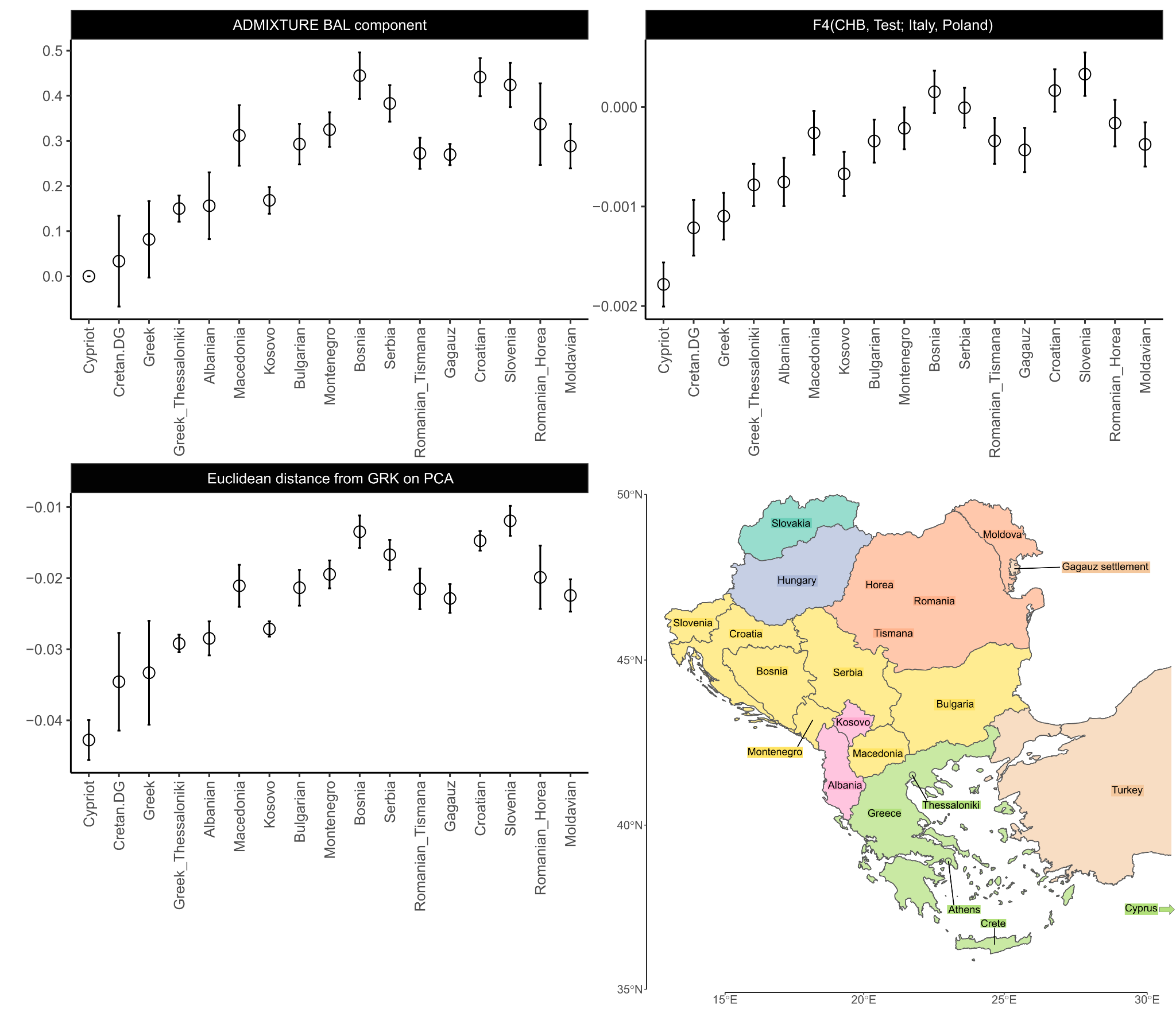
*

***Supp. Fig. 49. The genetic legacy of the Slavic expansion in Southeastern Europe.*** *Shown are different estimates of Eastern European affinity across 17 present-day groups (BAL ancestry from ADMIXTURE, the F_4_-statistic point estimates of the form F_4_(CHB, Test; Italy, Poland) and mean euclidean distances from GRK (n = 8) on the first 10 PCs of the European PCA).*

*
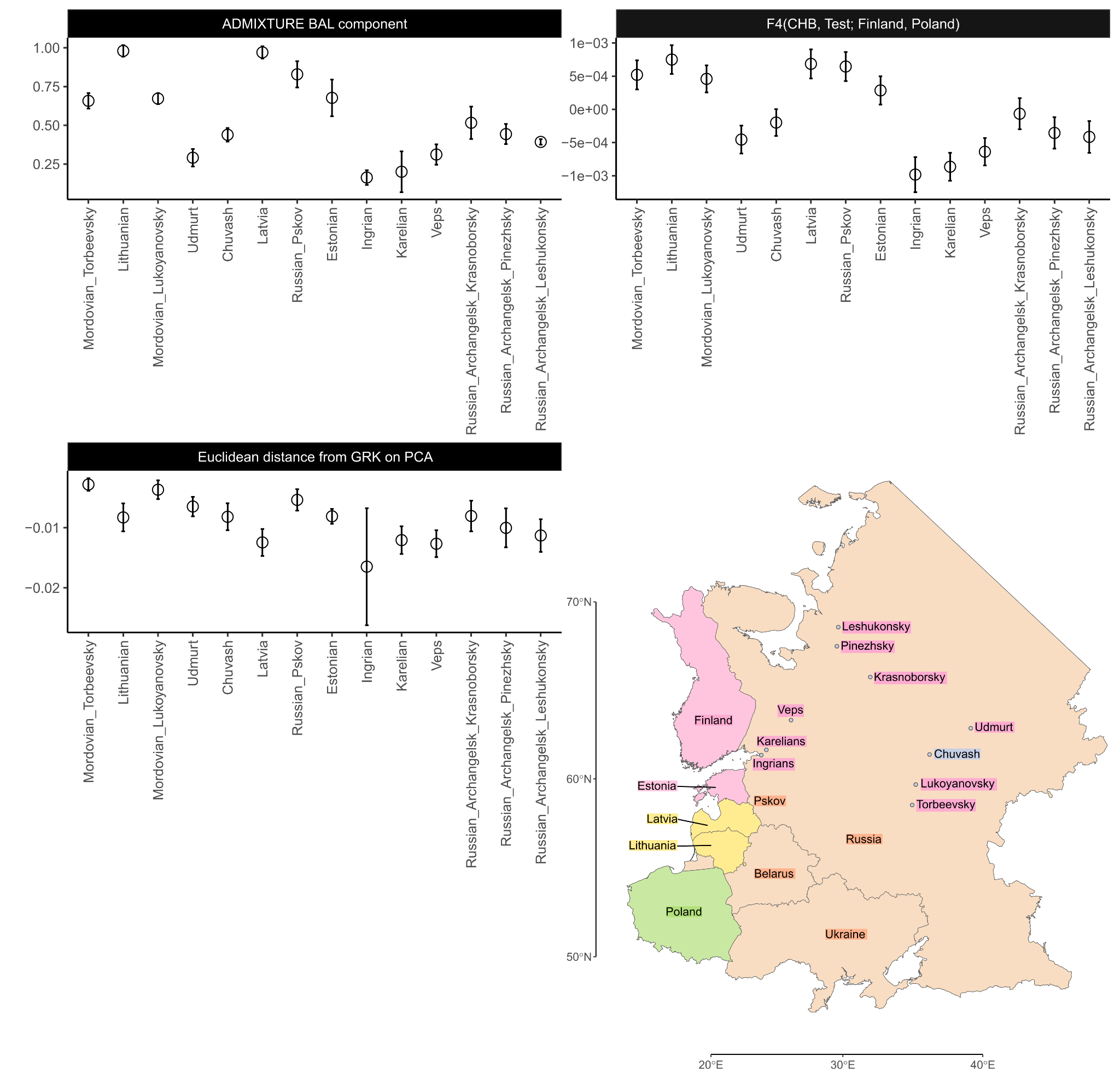
*

***Supp. Fig. 50. The genetic legacy of the Slavic expansion in Eastern Europe.*** *Shown are different estimates of Eastern European affinity across 14 present-day groups (BAL ancestry from ADMIXTURE, the F_4_-statistic point estimates of the form F4(CHB, Test; Italy, Poland) and mean euclidean distances from GRK (n = 8) on the first 10 PCs of the European PCA).*

*
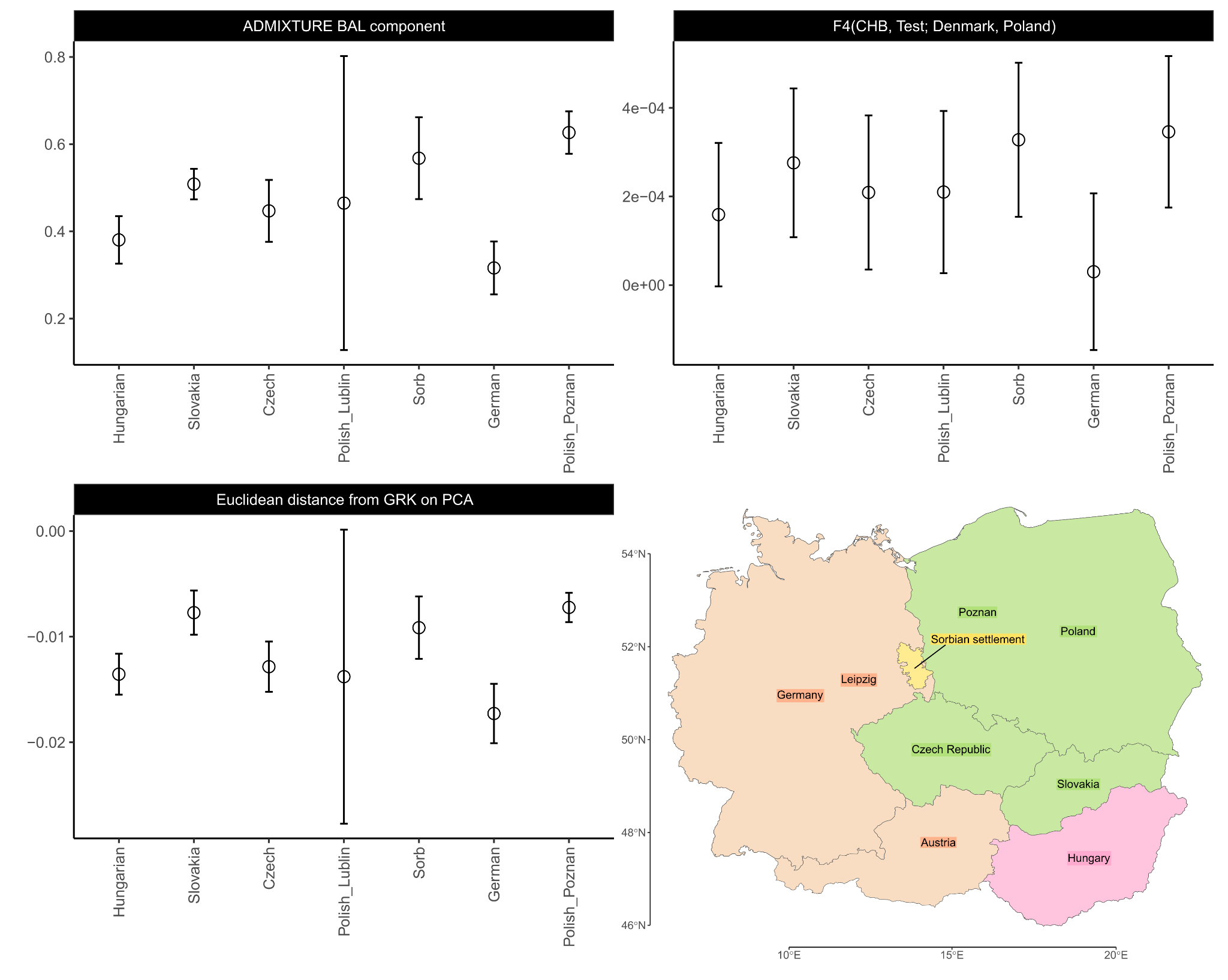
*

***Supp. Fig. 51. The genetic legacy of the Slavic expansion in Central Europe.*** *Shown are different estimates of Eastern European affinity across 7 present-day groups (BAL ancestry from ADMIXTURE, the F_4_-statistic point estimates of the form F4(CHB, Test; Italy, Poland) and mean euclidean distances from GRK (n = 8) on the first 10 PCs of the European PCA).*

*
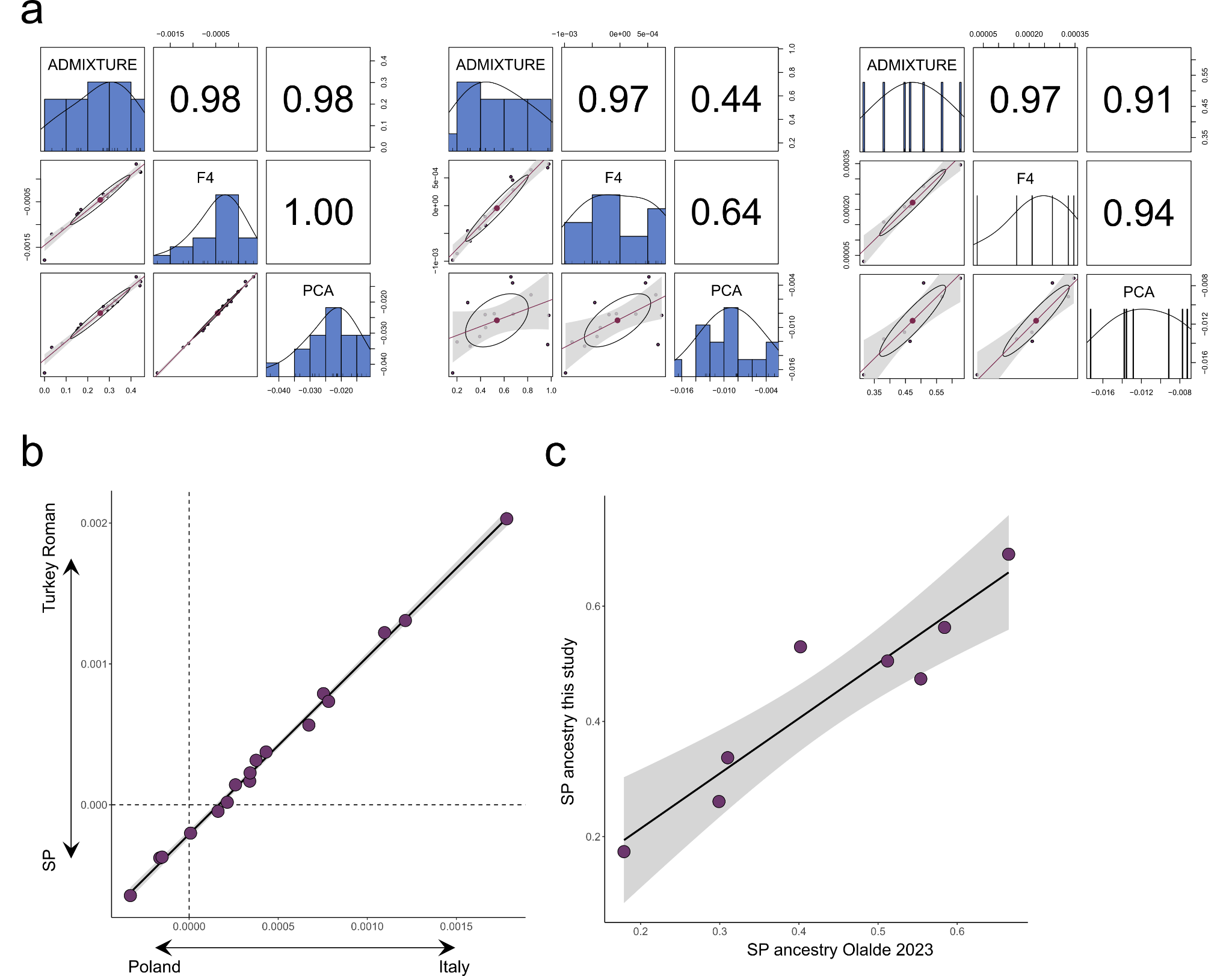
*

***Supp. Fig. 52. Cross-validation between different methods for estimating affinity to Eastern Europe.*** *a) Spearman's rho for the association between point estimates of BAL ancestry from ADMIXTURE, F_4_-statistic of the form F_4_(CHB, Test; Turkey_Hellenistic, NDW) and mean Euclidean PCA distance from GRK (n = 8) for groups from Southeastern Europe (n = 17), Eastern Europe (n = 14), Western Europe (n = 7), respectively. b) F_4_-statistics involving ancient or present-day references produce highly similar results for 17 present-day Southeastern European groups (Pearson's product-moment correlation; r = 0.998306, t = 66.455, df = 15, p < 2.2e-16). c) Novel estimates of Eastern European ancestry for 8 present-day groups from the Balkan Peninsula are highly similar to and significantly correlated with previous results from smaller-scale studies (Pearson's product-moment correlation; r = 0.9355021, t = 6.4856, df = 6, p = 0.0006387).*


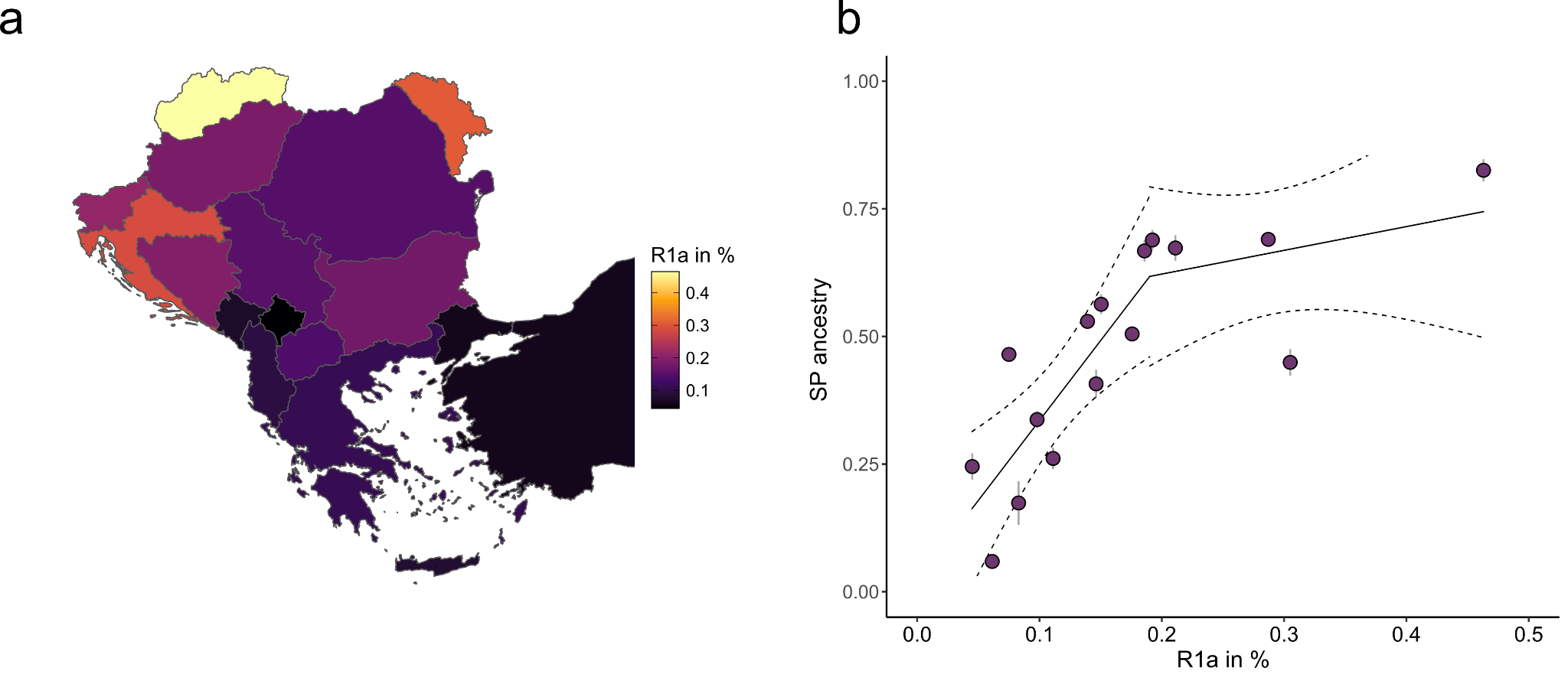


***Supp. Fig. 53. Relationship between autosomal and paternal ancestry.*** *a) The percentage of R1a haplogroups in the male population of 16 groups from the Balkan Peninsula and neighboring areas. b) The relationship between NDW-related ancestry and fraction of R1a haplogroups in male individuals from the Balkan Peninsula and neighboring areas modeled as a two-stage linear process.The dotted lines indicate the 95% confidence interval. Error bars indicate two standard errors.*

## 6. Diversity, Admixture and Population size

To identify trajectories of genetic diversity, we applied FSTruct[^147^](https://paperpile.com/c/UPmHk7/ParX9), a F_ST_-based method to quantify the variability of Q matrices (matrices with row vectors of ancestry coefficients) from ADMIXTURE in the form of the ratio F_ST_/F_ST_^max^. We computed F_ST_/F_ST_^max^ for different time groups (Late Neolithic, Bronze Age, Iron Age, Roman/Migration Period, medieval, and present-day) from each of the three transects (Northwestern Balkan, Eastern Germany, Poland-Northwestern Ukraine), measuring ancestry variability of the inferred cluster memberships within each of the 20 groups (Fig. S54a-c). We observe roughly similar patterns across the three transects: Late Neolithic groups tend to have greater variability in ancestry according to the F_ST_/F_ST_^max^ measure than postceding Bronze Age groups. This trend is reversed during the Roman Period and Migration Period when levels of membership coefficient variability increase substantially again, reaching ratios far exceeding any fractions measured during the Bronze and Iron Age (Roman/MP ratios for Northwestern Balkan, Eastern Germany and Poland-Northwestern Ukraine are 0.2130819, 0.3993415 and 0.396162 respectively). With the onset of the later Early Middle Ages, in our dataset associated with the expansion of Northeastern European ancestry, ancestry variability collapsed to pre-Roman Period levels. However, we note that this does not indicate that the SP gene pool was genetically impoverished since the Slavic Period F_ST_/F_ST_^max^ ratios equal or even exceed Bronze Age and Iron Age levels in the three transects (Slavic Period ratios for the Northwestern Balkan, Eastern Germany and Poland-Northwestern Ukraine are 0.1089308, 0.07735975 and 0.2006728 respectively). Rather, it suggests that the Roman and Migration Period stand out in their genetic diversity, surpassing the ratios of membership coefficient variability of all preceding post-Neolithic periods. Interestingly, membership coefficient variability observed in present-day Sorbs from Eastern Germany (0.07608005) is similar to Slavic Period genomes from the same area, yet higher than the diversity measured in present-day (Germanic-speaking) Eastern Germans from Leipzig (0.04829509), potentially indicating ongoing influx of German-related ancestry into the Sorbian population.

We also assessed demographic change throughout the last 5,000 years estimating runs of homozygosity (RoH), which can occur when the parents of an individual are closely related (Fig. S54d,e, Table S35, S36). The larger the pool of people from which individuals draw their mates, the less likely it is for parents to be closely related. Thus, the average number of 4–8 centimorgan (cM) runs of homozygosity segments is informative about the effective size of the pool of people within which people were mating in the approximately 600-year period before the time when the analysed individuals lived[^120,148^](https://paperpile.com/c/UPmHk7/6popn+iIPiM). Including all individuals that exceed a quality threshold of 300,000 SNPs[^148^](https://paperpile.com/c/UPmHk7/6popn), we observe a general reduction in average RoH from the Bronze Age to the Early Middle Ages in both the Northwestern Balkan and Poland-Northwestern Ukraine but not in Eastern Germany (where we unfortunately lack samples sizes for the Bronze Age that are comparable to the other two study transects). Interestingly, close kin unions were rare at all periods as reflected in the paucity of individuals harbouring >50 centimorgans (cM) of their genome in runs of homozygosity >12 cM[^120,148^](https://paperpile.com/c/UPmHk7/6popn+iIPiM). This is consistent with our overall observation that average sRoH (in all length categories) is not elevated in the SP gene compared to the preceding LA/MP gene pools. Instead, in the Northwestern Balkan, average sRoH of all length categories decrease substantially from 4.1 cM, 3.2 cM, 4.3 cM, and 11.8 cM to 1.2 cM, 0.9 cM, 0.0 cM, and 0.5 cM for sRoH[4,8], [8,12], [12,20] and [>20] respectively. A similar reduction, though less pronounced, is also evident in Poland-Northwestern Ukraine with a decrease from 5.1 cM, 1.1 cM, 4.6 cM and 11.7 cM to 2.2 cM, 0.4 cM, 0.0 cM and 0.0 cM on average for sRoH[4,8], [8,12], [12,20] and [>20] respectively. Only in Eastern Germany, we observe no major change in mean sRoH between the Migration Period and Slavic Period (from 1.3 cM, 0.8 cM, 0.1 cM and 0.2 cM to 2.4 cM, 0.9 cM, 0.4 cM and 0.0 cM for sRoH[4,8], [8,12], [12,20] and [>20] respectively).

Since sRoH[4,8] is driven by co-ancestry within the last few dozen generations[^148^](https://paperpile.com/c/UPmHk7/6popn), we can rule out that the recent ancestors of the SP populations in Central Europe sustained small effective population sizes or underwent a severe bottleneck that reduced the effective population size (*Ne*) to less than ~2,000. Instead, we measure similar effective population sizes of 15,433 (10,334-24,359 95% CI), 8,457 (6,920-10,551 95% CI) and 10,406 (7,467-15,221 95% CI) which are values typical for relatively large populations (Fig. S55a). Interestingly, in the case of the Northwestern Balkan and Poland-Northwestern Ukraine, these estimates even exceed the effective population size of the MP populations (8,938 (6,458-12,894 95% CI) and 4,354 (3,257-6,026 95% CI) for the NWBalkans and Poland-NWUkraine resp.). Only in Eastern Germany do we measure a larger *Ne* during the MP compared to the SP (14,603 (10,551-21,067 95% CI)) (Fig. S55a).

Furthermore, the absence of first cousin or closer unions across Slavic Period individual in all three study transects strongly suggests that consanguinity was avoided and knowledge of the individuals’ pedigrees was preserved despite the high rates of polgymay/serial monogamy and occurrence of extensive networks of biological relatedness.

Consequently, the observed demographic transformation was not only characterized by a replacement of ancestry but also by a homogenization of the gene pool. In all three study regions, variability in ancestry profiles[^52^](https://paperpile.com/c/e5c4dc/gekBg) significantly decreased compared to the Roman/Migration Period, indicating that less genetic outliers were present in the SP population and/or that admixture with divergent populations occurred less frequently (Fig. S54a-c). However, we highlight that this decrease is not indicative of a genetically depleted SP population, which shows in all three regions levels of genetic diversity similar to the preceding Bronze Age or even succeeding present-day groups. There is, furthermore, no evidence for excess parental relatedness compared to the MP populations[^53^](https://paperpile.com/c/e5c4dc/JJtpe) (Fig. S54d-e). Instead, our results emphasize the considerable genetic diversity during the Roman and Migration Period, both within and outside the borders of the (former) Roman Empire, compared to the preceding and succeeding time periods.


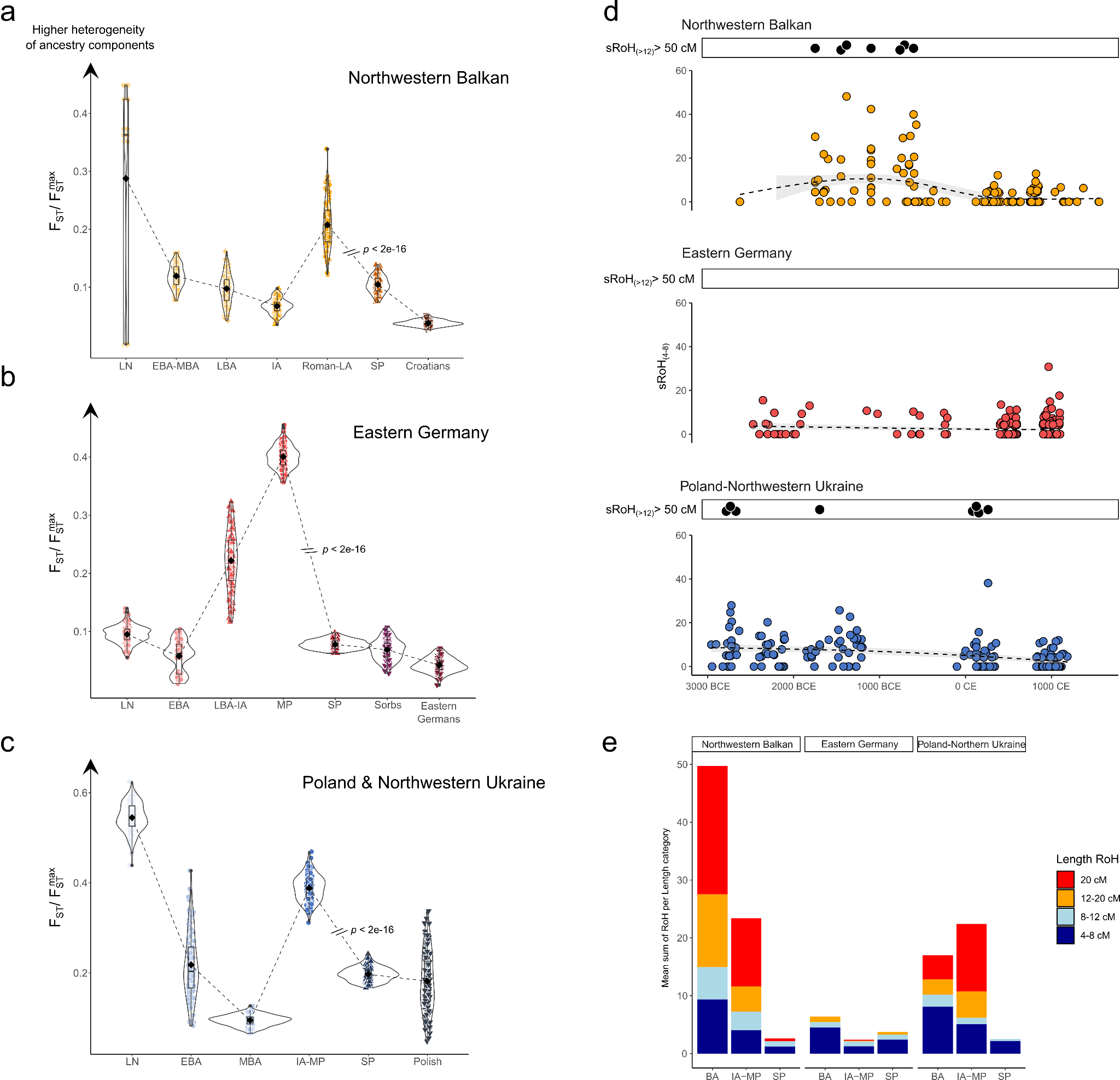


***Supp. Fig. 54. Changes in genetic diversity in Central Europe through time.*** *a-c) Violin and box plots depict the Bootstrap (n = 1000) distributions of the ancestry variability measure, F_ST_/F_ST_max, obtained from FStruct calculated on the Q matrix from supervised ADMIXTURE for each ancient and present-day group in our three sturdy transects. P-values from internal two-sided Wilcoxon rank sum tests comparing the bootstrap distributions of pre-SP (Roman/MP) and SP groups are highlighted. a) For ancient and present-day groups from the Northwest Balkan. b) For ancient and present-day groups from Eastern Germany. c) For ancient and present-day groups from Poland-North Ukraine. d) The sum of RoH (denoted here as sRoH) of size 4–8 cM for individuals (represented as circles) within the three geographic transects across time (n = 188, 273, 177, resp.). Close kin unions were rare at all periods as reflected in the paucity of individuals harbouring >50 centimorgans (cM) of their genome in runs of homozygosity (ROH) of >12 cM (black dots in top panel) (n = 9, 0, 8, resp.). e) Mean sum of RoH for Bronze Age (BA), Iron Age to MP (IA-MP), and SP/medieval populations from the three study transects represented by stacked vertical bars, where the length of each bar is determined by the mean sum of RoH of this group falling into four length classes (4–8, 8–12, 12–20, and >20 cM, color-coded).*

However, we note that the SP populations in Eastern Germany and the Velim experience substantial fluctuations in effective population size. We applied IBDne[^149^](https://paperpile.com/c/UPmHk7/GLMj1) to our ancient genomes, which estimates recent effective population size by using inferred long segments of identity by descent (IBD) (Fig. S55b). For SNP data the patterns of IBD sharing are information from around 4 generations to around 50 generations ago[^149^](https://paperpile.com/c/UPmHk7/GLMj1). We observe that the effective population sizes in both study regions remained stable until 500 BCE to 0 BCE when both populations experienced a substantial reduction in effective population size. Only between 400 CE and 900 CE, the effective population sizes dramatically increased again, quickly surpassing the initial population sizes, reaching estimates of *Ne* > 50,000 (Fig. S55b).

Arguably, this demographic trajectory might reflect the shared recent population history of the ancestors of the SP populations in Europe. Consequently, the reduction in effective population size might represent the bottleneck associated with the initial split and expansion of their population across the continent. This decrease is then followed by a sudden and rapid resurgence of the effective population size related to the settlement and rapid growth of the SP population in the study transects.

Interestingly, this signal inferred from the ancient genomes is consistent with patterns of IBD-sharing among present-day Slavic-speaking groups. As noted in previous publications[^150^](https://paperpile.com/c/UPmHk7/7H2vz), South Slavic-speaking populations share significantly fewer IBD segments for length classes 1–3 cM with their immediate geographic neighbors on the Balkan Peninsula (e.g. Albanians, Kosvarians and Greek) than North Slavic and Baltic-speaking populations (Fig. S56). Temporally this corresponds to a time depth of 1,500 years ago, since a common ancestor n generations in the past (2n meiosis) results on average in 100/2*n* cM segment length[^151^](https://paperpile.com/c/UPmHk7/UYuTp). Thus, IBD segments longer than 1 cM derive from common ancestors living ~50 generations in the past, or ~1,500 years ago, assuming an average human generation time of 30 years[^152,153^](https://paperpile.com/c/UPmHk7/HONP2+GTMNq). This period coincides with the historical expansion of the Slavs, the demographic signal of an initial bottleneck followed by rapid growth observed in the IBD sharing of the ancient genomes as well as the admixture dates obtained for the Velim and Mödling populations.


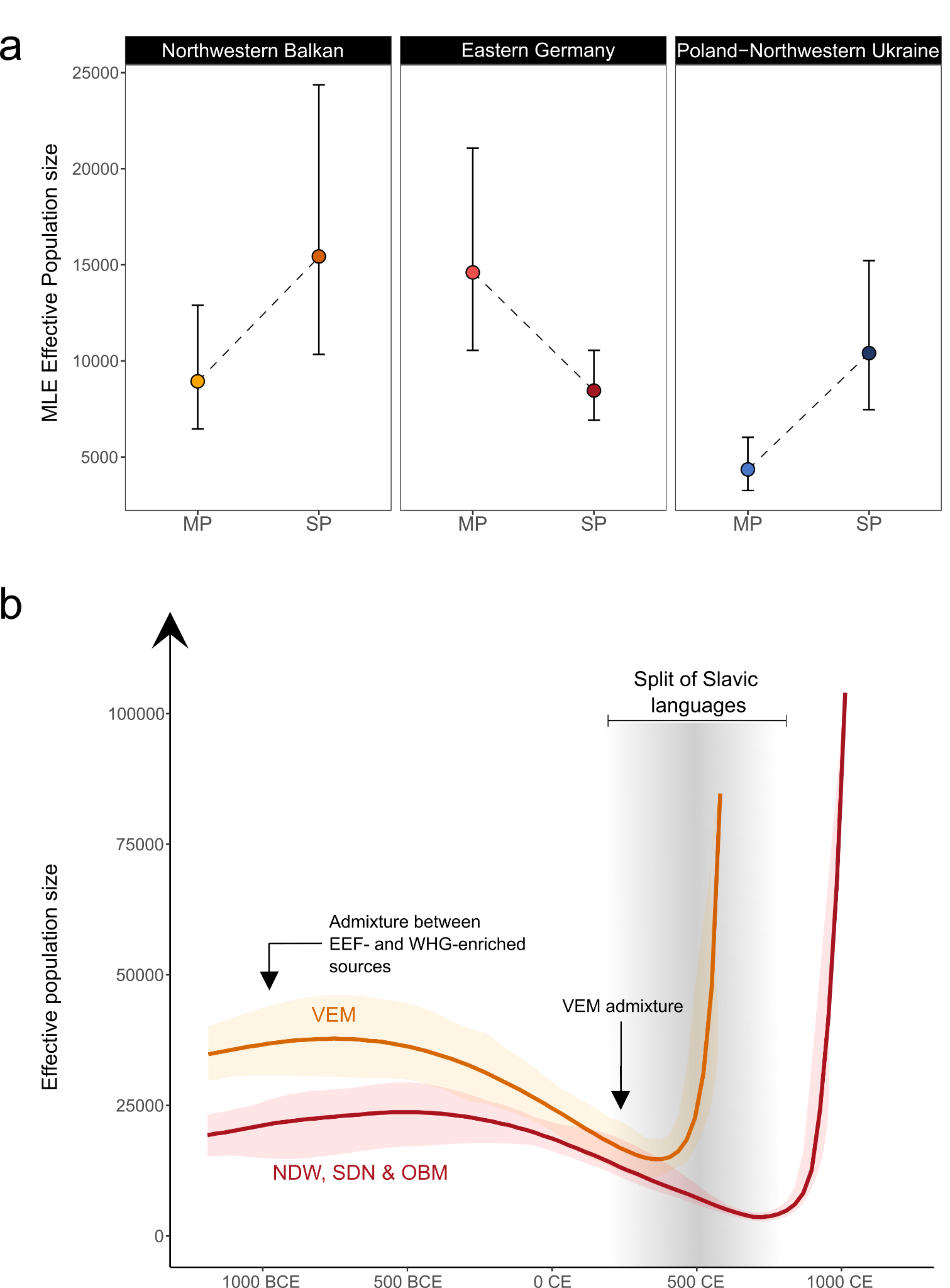


***Supp. Fig. 55. Changes in effective population size.*** *a) HapROH point estimates of effective population sizes for the grouped MP and SP sites from the Northwestern Balkan, Eastern Germany and Poland-Northwestern Ukraine inferred from RoH. b)* *IBDne estimates of effective population sizes for the grouped early medieval SP sites of NDW, OBM, and SDN from Eastern Germany (red) and Velim from Croatia (orange) over the last 100 generations (black line) inferred from IBD segments. The transparent bands indicate the 95% confidence interval. The threshold on inferred IBD length is 1 cM. Approximate admixture dates from DATES analysis and language split times from Bayesian phylogenetic inference are highlighted.*


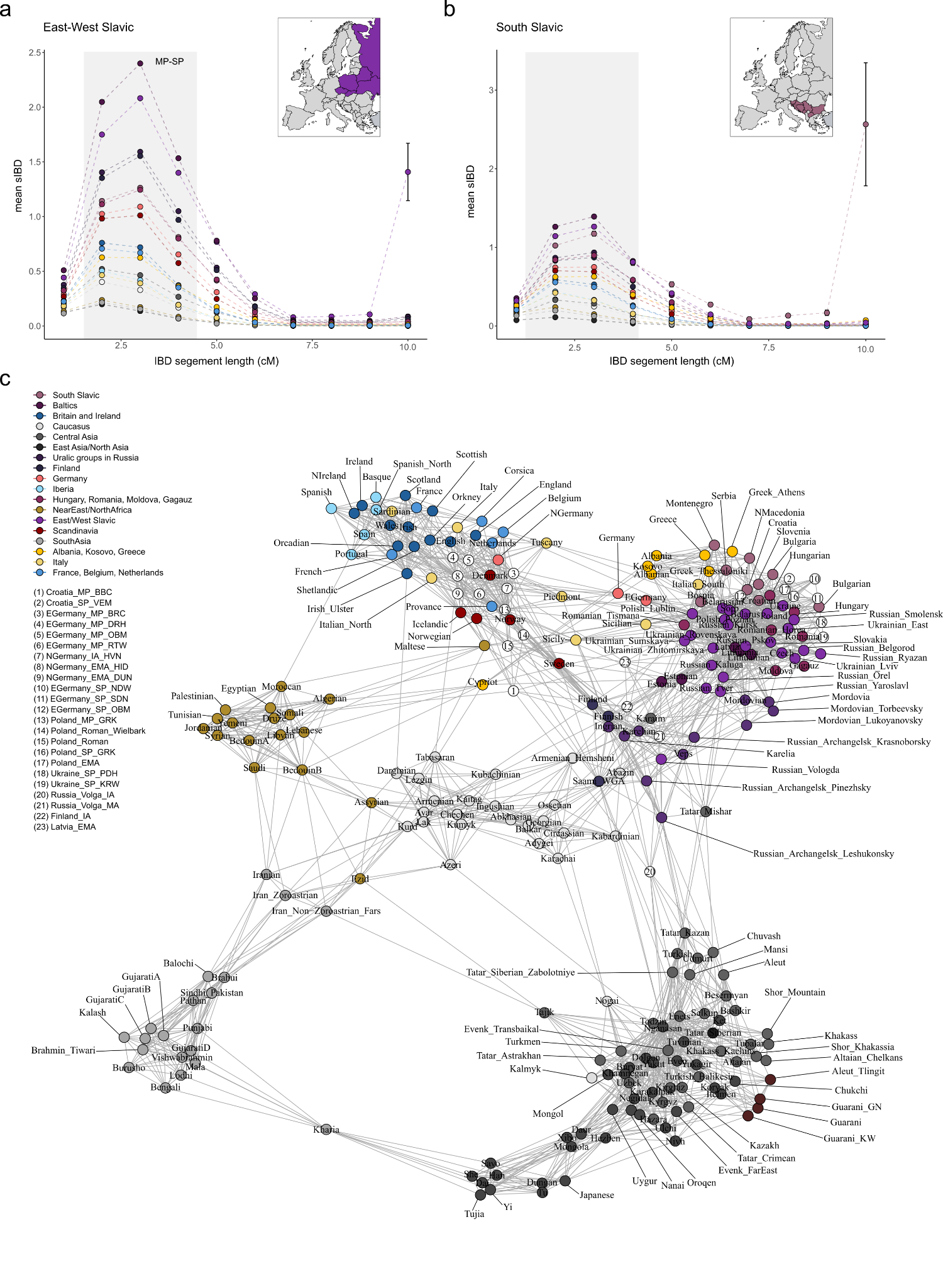


***Supp. Fig. 56. Genetic relatedness between groups in Europe through time.*** *a) Distribution of the average number of IBD segments shared between East-West Slavic groups and 17 other European populations. The x-axis indicates ten classes of IBD segment length (in cM); the y-axis indicates the average number of shared IBD segments per pair of individuals within each length class. Error bars indicate two standard errors. The map indicates which populations are considered part of the East-West Slavic group. b) same as panel a) but for the distribution of the average number of IBD segments shared between South Slavic groups and 17 other European populations. c) IBD network of ancient (n =23) and present-day populations (n = 215). Each node represents a population, and the width of edges connecting nodes indicates the fraction of the genome shared IBD between the respective pair of populations. Network edges were restricted to the 10 highest sharing connections for each population, and the layout was computed using the force-directed Fruchterman-Reingold algorithm. Duplicate populations represent data from different reference studies.*

## 7. Uniparental markers and sex bias

### 7.1 Uniparental markers

#### 7.1.1 MP

##### Eastern Germany

The Y-chromosome haplogroups observed in MP males from Eastern Germany show a typical Northwestern European distribution. We detect a prevalence of R1 lineages (57%), which is the most common haplogroup in Europe, with a majority of haplotypes belonging to lineage R1b-M343 (84%) and a minority belonging to lineage R1a-M420 (16%) (especially R1a-L260, a Central European branch of R1a-M420). While R1a-M420 is most commonly found in Northern and Eastern Europe[^154,155^](https://paperpile.com/c/UPmHk7/dzMYh+V4jQe), R1b-M343 peaks in the Iberian Peninsula as well as in the British-Irish Isles[^156,157^](https://paperpile.com/c/UPmHk7/CVVw8+PCbsR) and follows a west-east gradient in Central Europe[^154,158,159^](https://paperpile.com/c/UPmHk7/F9PZs+3sFXT+dzMYh). Within haplogroup R1b-M343, sub-haplogroup R1b-P312 has been, since the Bell Beaker period[^160^](https://paperpile.com/c/UPmHk7/VXDhR), the most frequent haplogroup in Europe west of the Rhine[^157,158^](https://paperpile.com/c/UPmHk7/F9PZs+PCbsR) and represents 17% of all the R1b-L151 lineages within our Thuringian Period sample. More frequently detected is haplotype R1b-U106 with a fraction of 79% of all haplogroups typed past R1b-L151, especially subclade R1b-L48 which peaks in present-day Friesland and North Holland (68% of all R1b-U106 haplogroups)[^161^](https://paperpile.com/c/UPmHk7/YgcGK). Today, the distribution of the R1b-U106 lineage in Europe is centred along the Rhine River[^158^](https://paperpile.com/c/UPmHk7/F9PZs). It is the dominant R1b-M343 sub-haplogroup in present-day Germany, the Netherlands, Denmark, Sweden and Norway[^157,158,162^](https://paperpile.com/c/UPmHk7/F9PZs+PCbsR+vdFRK) as well as the most prevalent haplogroup in early medieval males from England, Northern Germany and the Netherlands[^123^](https://paperpile.com/c/UPmHk7/tySmG).

The next-largest fraction of male lineages belongs to haplogroup I-M170 (25%), both in the I2-M438 (19% of I-M170 haplogroups) and, more abundantly, in I1-M253 sub-haplogroups (81% of I-M170 haplogroups). I1-M253 sub-haplogroups are only found in ancient and modern Northern Europe in high frequencies, especially in Scandinavia (reaching 52% in Central Sweden)[^123,125,153,163–166^](https://paperpile.com/c/UPmHk7/rBx3b+BPFMJ+tySmG+ZKQ13+pFUTr+GTMNq+ozxXN). In the ancient DNA record, subclade I1-M253 peaks in Iron Age and early medieval Northern Germany, as well as generally in Iron Age and Viking Age Denmark and Roman Iron Age Poland[^123–125,153,165^](https://paperpile.com/c/UPmHk7/tySmG+pFUTr+ZKQ13+GTMNq+LbEH5). Furthermore, we find the haplogroups E1b1b1a1b1-L618 (~7%), G2a2b2a1-Z764 (~7%) and J-M304 (both J1-L255 and J2-M172) (~4%), which today all show a Mediterranean distribution with a prevalence in Southern Europe, in particular across the Balkan, Iberian, and Italian Peninsulas[^167–169^](https://paperpile.com/c/UPmHk7/urIVN+AdZQh+B3C8T). However, they are also present in low frequencies in present-day and medieval Germany, the Netherlands and Scandinavia[^123,125,166^](https://paperpile.com/c/UPmHk7/ozxXN+ZKQ13+tySmG).

In summary, the MP population of the Eastern German Elbe-Saale region exhibits a diversity of Y-chromosome lineages highly similar to neighboring contemporaneous and later groups in Northern Germany. Both populations in Eastern and Northern Germany are characterized by a prevalence of R1b-L151 and I1-M253 haplogroups, followed by lower frequencies of I2-M438 and R1a-M420 lineages. While both groups feature also haplogroups with a more Southern European distribution (e.g. E, G, J and T), the frequencies of these haplotypes are substantially higher in Eastern Germany compared to Northern Germany (18% vs. 3%), mirroring the excess Southern European ancestry detected in the autosomal gene pool of MP Eastern Germany.

##### Poland & Northwestern Ukraine

Our Iron Age and MP sample from Poland shows a similar diversity of Y-chromosome lineages. Yet strikingly, R1b haplogroups are completely absent. The two most prevalent haplogroups are I-M170 (44%) and J2b2a1a1a1-Z2507 (33%). Among the I-M170 lineages, we find 3 cases of I1 (75%) and only one case of I2 (25%). Furthermore, we detect one instance (11%) of haplogroups E (E1b1b1a1b1-L618) and R1a (R1a1a1b1a2b-CTS1211), respectively. The West Eurasian R1a1a1b1a2b-CTS1211 subclade is today the most frequent R1a lineage in Eastern Europe, especially among Slavic-speaking groups. These frequencies are overall consistent with previously published Y-chromosome data from individuals associated with the Roman Iron Age Wielbark culture[^124^](https://paperpile.com/c/UPmHk7/LbEH5). Within this sample, haplogroup I-M170 was also identified as the prevalent lineage (51%), with I1-M253 sub-haplogroups representing 90% of all lineages. Haplogroup R1a was found in four males (9%), mirroring the fraction measured in our sample.

Haplogroups absent in our sample, namely G2a2b2a1a1b1a1a2-CTS6796 (a Northwestern branch of the Central European G-L497 lineage[^170,171^](https://paperpile.com/c/UPmHk7/We8hq+EAZTw)), R1b and N1a1a1a1a-L392 represent 12%, 14% and 5% of the lineages in these published male individuals. However, the underrepresentation of these haplogroups in our sample might simply be explained by the considerably smaller sample size, limiting our ability to identify rarer linageas.

Interestingly, both in our sample as well as among previously published Wielbark genomes[^124^](https://paperpile.com/c/UPmHk7/LbEH5), haplogroup I2 was only found in low frequencies (~5%). This contrasts with the Y-chromosomal diversity measured in preceding Early and Middle Bronze Age groups from Poland and Northwestern Ukraine[^139^](https://paperpile.com/c/UPmHk7/sHb1e). There, haplogroup I2 accounted for approximately 60% of all Y lineages, with I2a1a1b1a1-L233 representing 46% of all haplotypes. Yet, this lineage is virtually absent in the later Roman Iron Age and Migration Period[^124^](https://paperpile.com/c/UPmHk7/LbEH5). Similarly, haplogroup R1a is considerably more frequent, being found in ~30% of all males (with 4 individuals belonging to R1a1a1b1a2-Z280 lineages and only one to R1a1a1b1a1a-M458). On the other hand, haplogroup I1-M253 was only detected in a single individual (~2%), indicating a fundamental turnover in paternal ancestry after the Middle Bronze Age.

##### Northwestern Balkan

Within our Roman Period samples from Bubi’s cave, Croatia, we observe a comparably low diversity of Y lineages. 71% of the males belong to haplogroup J (9 samples/90% to J2b-M12 and 1 sample/10% to J1a-Z2215) and 21% to haplogroup G (mostly G2a-P15). We furthermore identified a single instance of haplogroup E1b1b1a1b1-L618 (7%). This is remarkable since haplogroup E (specifically E1b1b1a1b1a-L142.1) is the most common Y lineage in previously published Roman Period samples from Serbia and Croatia (~37%)[^109,111,113^](https://paperpile.com/c/UPmHk7/cvmfh+Roybd+Ejy7a). Today, this haplogroup peaks in the Southern Balkan Peninsula, in Albanians (>32%), followed by Greeks (~18%), North Macedonians (17%) and Bulgarians (16%)[^172^](https://paperpile.com/c/UPmHk7/dBRBd) and was identified in ancient individuals from Bulgaria, Turkey and North Macedonia[^111^](https://paperpile.com/c/UPmHk7/Roybd). Consequently, the increase of E1b-L142.1 in the Northern Balkan Peninsula during the Roman Period might be associated with the influx of Aegean/West Asian/Eastern Mediterranean ancestry subsequent to the incorporation of the region into the Roman Empire[^109,112,113^](https://paperpile.com/c/UPmHk7/PvwZ8+Ejy7a+cvmfh). As discussed previously, the population of Bubi’s cave exhibits substantially less introgression from the Eastern Mediterranean than groups from contemporaneous Roman colonies and larger supra-regional centers in the Northern Balkan Peninsula, potentially explaining the lack of E1b-L142.1 lineages in the Bubi´s Cave paternal gene pool. Overall, the BBC population thus preserves a Y-haplogroup composition highly similar to the preceding Iron Age population of Croatia, Slovenia and Serbia (featuring a prevalence of J2b-L283 haplogroups, accounting for 55% of all lineages), highlighting the regionally-contingent trajectories of immigration from the Southern Balkans and Asia Minor during the Roman rule.

Averaging across 53 new and published ancient genomes from Croatia and Serbia[^113^](https://paperpile.com/c/UPmHk7/cvmfh), we find that 30% of the males carry haplogroup E (mostly E1b1b1a1b1a-L142.1), 28% haplogroup J, 15% haplogroup G (predominantly G2a2-L1259), 15% haplogroup R1b (with the majority belonging to R1b-P312), 4% haplogroups R1a and I1, respectively, as well as 2% haplogroup T and 2% haplogroup I2 (which shows today the highest percentages in Serbia, Croatia and Bosnia-Herzegovina where it is also the most frequent paternal lineage in the male population[^173^](https://paperpile.com/c/UPmHk7/U0Xfw)). Especially haplogroups J and R1b were also frequent during the preceding Iron Age (55% and 30%, resp.) and are ultimately Bronze Age-derived (with frequencies of 41% and 43% for J and R1b during the MBA and LBA, resp.).

#### 7.1.2 SP

##### Eastern Germany

The Y-chromosome haplogroups observed in SP males from Eastern Germany evidence a fundamentally different composition of the paternal gene pool during the Slavic Period compared to the preceding Migration Period. Specifically, the diversity in Y-chromosome lineages drastically declined, with only a single instance of the haplogroups G2a-P15, I1-M253, and J2a-M410 found among males from Obermöllern, Steuden, Niederwünsch and Halle-Reideburg. Furthermore, the frequency of lineage R1b-M343 decreased from 84% to 6% (consisting of 50% R1b-P312 and 50% R1b-U106 lineages). On the other hand, proportions of haplogroups R1a-M420 and I2-M438 increased to 72% and 19%, respectively. Specifically, among the R1a haplotypes typed past R1a1a1b1-Z283, 86% of the Y lineages belong to R1a1a1b1a2b-CTS1211, which today peaks in Western Russia, Belarus and Ukraine[^174,175^](https://paperpile.com/c/UPmHk7/GkDJI+sJg29) (Fig. S57a). However, it is well possible that of the 74 R1a haplotypes up to 69 (93%) belong to this sub-haplogroup. Furthermore, we detect four lineages belonging to R1a1a1b1a1a1c1-L1029 (found in one medieval male from Ukraine[^125^](https://paperpile.com/c/UPmHk7/ZKQ13)) as well as one case of R1a1a1b1a3a1a-CTS4179 (a haplotype also detected in two Viking Age individuals from Scotland and Sweden). Among haplogroup I2, the majority of identified haplotypes (74%) belongs to I2a1a2b-L621, which is most prominent in Slavic-speaking populations from the Balkans and Eastern Europe[^174,175^](https://paperpile.com/c/UPmHk7/GkDJI+sJg29) (Fig. S57c). Interestingly, we also identify one male carrying supra-haplogroup Q1b-M346, a lineage today most commonly found in Central Asia[^176^](https://paperpile.com/c/UPmHk7/XHlBL). In the archaeogenetic record, his specific haplogroup Q1b1b1~ was also identified in an Iron Age Sargat male from Central Russia as well as a medieval Alan individual from the Caucasus[^177,178^](https://paperpile.com/c/UPmHk7/V7Sgx+0D3uW). Altogether, this strongly suggests a major turnover of paternal ancestry in the early medieval Elbe-Saale region that resulted in the replacement of nearly all local Y-chromosome lineages and the introduction of new paternal ancestry from the East.

##### Poland & Northwestern Ukraine

An almost identical pattern is seen for Poland and Northwestern Ukraine. Among our sites of Pidhirtsi/Podhorce, Korolivka/Korolówka (both Ukraine) and Gródek (Poland), we observe comparable high frequencies of R1a (50%) and I2 (28%) haplotypes, yet, not a single occurrence of haplogroup I1-M253 or R1b. The majority of high coverage R1a sequences belong to R1a1a1b1a2b-CTS1211 lineages, yet we also identify two instances of R1a1a1b1a1a1-Z2942 and one instance of R1a1a1b1a2a1a1a-YP569. Furthermore, we identify two males carrying E1b1b1a1b1-L618 and one male featuring G2a2b2a1a1a2a~ and J2a1a1a2b2a-Z1847 haplotypes, respectively, which might represent Iron Age-derived lineages that persisted during the major population turnover between the Wielbark Culture and the Slavic Period.

Grouping our new data together with previously published Y-chromosomal evidence from medieval Poland and Ukraine[^124,125^](https://paperpile.com/c/UPmHk7/LbEH5+ZKQ13), we identify R1a as, by far, the most prevalent Y-chromosome lineage during the Slavic Period in this region, accounting for 65% of all haplotypes and mirroring the turnover in paternal ancestry observed in Eastern Germany. In contrast to Eastern Germany, however, R1a1a1b1a2-Z280 haplotypes (including R1a-CTS1211) represent only 41% of all lineages genotyped past R1a1a1b1a-Z282. Instead, R1a1a1b1a1a-M458 represents with 52% the most dominant R1a haplotype of the Polish-Ukrainian SP population. Until today, R1a1a1b1a1a-M458 is more frequent in Poland (as well as in Croatia and the Czech Republic) than R1a-CTS1211, contrasting with most other populations in Central, Eastern and Southeastern Europe[^154^](https://paperpile.com/c/UPmHk7/dzMYh) (Fig. S57b). Since R1a1a1b1a2-Z280 lineages outnumber R1a1a1b1a1a-M458 haplotypes 4:1 during the Bronze Age in Poland and Western Ukraine[^139^](https://paperpile.com/c/UPmHk7/sHb1e) it appears unlikely that this divergence might be related to a Bronze Age-derived substrate in the region. Instead, patrilineal and patrilocal social practices could have contributed to the differentiation in R1a-Z280 diversity. The next most frequent haplogroup is I-M170 (16%), although most of its diversity is shaped by I2 lineages (75%) while I1 haplotypes are rare (25%). This change from I1 to I2 and R1a haplogroups clearly evidences the substantial transformation of the MP diversity of paternal ancestries by incoming lineages from the East.

Similar to Eastern Germany, we find a drastic reduction in the proportion of R1b-M343 lineages, representing 7% of the SP haplogroups. Furthermore, we continue to identify low frequencies of haplogroups E (especially E1b1b1a1b1-L618) (7%), G2a2b2a1a1a2a (~1%), J2a1a-L27 (especially J2a1a1a2b2a-Z1847) and N1a1a1a-L708 (2%), suggesting at least partial integration of the local gene pool into the SP communities. However, haplogroups E, G2a and J2a were also found further northeast, in SP individuals from the Russian Volga Oka region[^129^](https://paperpile.com/c/UPmHk7/OnSBn) (jointly accounting for 27% of all Y lineages in the sample). Thus, we can not conclusively state whether these haplogroups were integrated into the SP paternal gene pool when its members first started to expand westwards and were only subsequently brought to the region in a later migration wave or if these lineages were already part of the initial SP gene pool before its expansion across Europe. In the latter case, these haplogroups might not represent contributions of paternal ancestry from the local pre-SP populations. Instead, they might reflect the Southern European-related gene flow seen in the autosomal ancestry that formed, together with Baltic Bronze Age-related ancestry, the core gene pool of the initial SP population during the Middle or Late Bronze Age.

##### Northwestern Balkan

In contrast to Eastern Germany, Poland and Ukraine, the Northwestern Balkan (here especially Croatia) retains a much higher diversity of Y lineages during the Slavic Period. We identify the highest proportion of lineages belonging to haplogroup I-M170 (39%), especially I2-M438 (>61% of all I-M170 lineages). Haplogroup I-M170 is followed by J (24%) (especially J2b2a1a1a1b-CTS3617 and J2b2a1a1a1a1a1a-Z631 with 46% each), which is the most frequent Y supra-haplogroup in the Northern Balkans during the Iron Age[^111,120^](https://paperpile.com/c/UPmHk7/iIPiM+Roybd), and Eastern European-derived haplogroup R1a (17%). There are further lower frequencies of haplogroups E (especially E1b1b1a1b1a-L142.1), G2a2-CTS4367, N1a1a1a1a1a1a1-B215, and R1b1a1b1b3a1a1a-Y5587, which account for ~4% of the total Y-chromosomal diversity each. Indicative of Eastern European introgression, we identify besides haplogroup N1a1a1a1a1a1a1-B215 (which peaks in present-day Lithuanians, followed by Latvians as well as Estonians and was found in Iron Age, Viking Period and medieval males from Estonia)[^179–182^](https://paperpile.com/c/UPmHk7/AWjYJ+V5de8+raH7r+NkzNm), one instance of haplogroup Q (Q1a2a1) (~2%), which shows a Central and East Asian distribution and was also found in Iron Age individuals from Siberia and MP males from Kyrgyzstan[^177,183^](https://paperpile.com/c/UPmHk7/GmVUo+V7Sgx). Interestingly, we show that haplogroup E is nearly absent in our sample, although it is the most frequent local haplogroup during the Roman Period[^109,111,113^](https://paperpile.com/c/UPmHk7/Ejy7a+cvmfh+Roybd). This divergence might be related to the overrepresentation of Roman colonies and cosmopolitical trade centers that experienced higher influx of West Asia/Eastern Mediterranean ancestry during the Roman Period in the pre-SP sample, resulting in an overestimation of the frequency of haplogroup E in the Northern Balkans during the Roman Period. Finally, we highlight that the frequency of R1a (mostly represented by R1a1a1b1a2b-CTS1211) is substantially lower than the proportions measured in Eastern Germany, Poland and Ukraine. Instead, haplogroup I2 appears to be the dominant Y lineage of the SP gene pool in Croatia during the second half of the first millennium CE. For example, outside Velim, we identify in the SP sites of DUC, DUG and TOR exclusively I2a1a2b-L621 haplotypes. This seemingly changes (to some extent) after the Middle Ages as the frequency of Haplogroup R1a increases to more than 28% in the present-day population[^154,159^](https://paperpile.com/c/UPmHk7/3sFXT+dzMYh). Yet, this difference could be also (partly) due to sampling bias as these SP sites are patrilinear.

#### 7.1.3 Extended Discussion II

We visualize trends in Y-chromosomal diversity as dendrograms by determining the absolute and relative frequencies of Y-chromosome haplogroups across ancient and present-day samples from this and previous studies and applying hierarchical cluster analysis. In accordance with the autosomal ancestry, we find that the LA/MP populations from Eastern Germany and Poland cluster together with ancient and present-day groups from Northwestern Europe (Fig. S58a). Specifically, the MP population from Eastern Germany shows the highest similarity to present-day Dutch as well as Iron Age and early medieval groups from the continental North Sea coast (the Netherlands, Northern Germany and Denmark). The Wielbark culture samples from Poland cluster on the other hand with ancient and present-day Scandinavians, especially Viking Period genomes from Norway and Sweden as well as present-day Swedish. Consequently, the Y chromosomal data suggest a relatively stable, Northwestern European-derived paternal gene pool in the two study transects during the Iron Age and Migration Period with a predominance of R1b-M343 (especially R1b-U106) and I1-M253 haplotypes.

Interestingly, in the Balkans, we observe a notable shift in the Y lineage diversity with a marked decrease of R1b-L151 as well as J2b2a1-L283 haplotypes and surge of E1b1b1a1b1a-L142.1 haplotypes, causing higher affinities to Iron Age groups from the Southern Balkans (Greece, Albania, North Macedonia etc.). Most probably this reflects the influx of Aegean/West Asian-related ancestry observed in the autosomal DNA during the 1^st^ to 3^rd^ century CE, associated with immigration from the Eastern Mediterranean subsequent to the incorporation of the region into the Roman Empire[^109,112,113^](https://paperpile.com/c/UPmHk7/PvwZ8+cvmfh+Ejy7a).

In contrast, during the SP period, we observe an almost identical pattern across the three study transects. The SP paternal gene pool is characterized by a substantial increase of non-local, Eastern European-derived haplogroups, especially R1a and I2a lineages (from 13% ± 3% and 15% ± 4% to 90% ± 4% and 75% ± 4% of all haplogroups, respectively) (Fig. S58a,b). In Eastern Germany and Poland-Northwestern Ukraine this turnover amounts to a nearly-complete replacement of the local Y-chromosome diversity, mirroring our results from genome-wide analysis. As a consequence of this transformation, the Y diversity of the SP paternal gene pool largely resembles the composition of earlier Corded Ware Central and Eastern European as well as Western Bronze Age Baltic groups, featuring a prevalence of R1a and I2 haplotypes, yet, a lack of N lineages associated with the Bronze and Iron Age in the Eastern Baltics and Western Russia (Fig. S58a & S59a). Since R1a and I2 frequencies appear to be associated with BAL ancestry over time (Fig. S59b,c), we tested if autosomal BAL ancestry was correlated with non-local Y-chromosome haplogroups (here R1a, I2, N, and Q) in ancient post-Neolithic individuals. We find that ancient post-Neolithic individuals from the three study transects with immigrant Y-chromosome haplogroups carried significantly more BAL ancestry on the autosomes (Wilcoxon rank sum test; *W* = 161, *p* = 8.579e-05; *W* = 840.5, *p* = 5.984e-15; and *W* = 487, *p* = 3.301e-15 for the Northwestern Balkans, Eastern Germany and Poland-Northwestern Ukraine, respectively) than those with local haplogroups. Interestingly, with the exception of Poland-Northwestern Ukraine (*W* = 181, *p* = 1.852e-06), this effect was not significant when only considering SP individuals (*W* = 129, *p* = 0.7101 and *W* = 399, *p* = 0.8838 for the Northwestern Balkans and Eastern Germany, respectively), indicating that local Y haplogroups are (at least on the individual level) not a good proxy for local autosomal ancestry in the Slavic settlement areas further to the West and South.

We formally tested if these patterns of differentiation were statistically significant using Fisher’s exact test. We find that both the LA/MP populations of Eastern Germany, the Northwestern Balkan and Poland-Northwestern Ukraine significantly differ in the composition of their paternal gene pools from their SP successors (for all three *p* < 0.0005). Furthermore, we infer that the SP populations of Eastern Germany and Poland-Northwestern Ukraine differ significantly in their Y-chromosome frequencies (*p* = 0.02014). This is mostly related to the substantially higher frequencies of non-local haplogroups (R1a and I2) in Eastern Germany (72% and 19%, resp.) compared to Poland-Northwestern Ukraine (65% and 10%), while Poland-Northwestern Ukraine retains higher frequencies of local Y lineages (e.g. E, I1 and J2a). Multiple mechanisms could produce this differentiation between these generally closely-related groups: i) Higher rates of admixture with the local gene pool might have diversified the paternal gene pool in Poland and the Ukraine; ii) the Y-chromosomal gene pool in Poland and Ukraine, regions closer to the origin area of the SP gene pool, was more heterogeneous than the paternal gene pool of SP Eastern Germany, which faced a loss of diversity and homogenisation due to the bottleneck associated with the expansion to the West; or iii) the highly patrilineal and patrilocal reproductive strategies practiced among SP groups in Eastern Germany caused subsequent homogenisation and regionally contingent segmentation of the Y-chromosomal diversity. A substantial social contribution to the distribution of Y lineages seems likely since for Eastern Germany fractions of Y lineages seem to be strongly site-specific during the Slavic Period. For example, the Slavic Period cemetery of Obermöllern is dominated by I2 lineages (especially I2a1a2b1a1-CTS5966), which are rare in Niederwünsch. Conversely, the vast majority of males from Niederwünsch carry R1a haplogroups which are completely absent in Obermöllern. Only in Steuden we find approximately equal fraction of both haplotypes (as well as of lineage R1b, with both R1b-P312 and R1b-U106 haplotypes being present). Within Steuden, we also measure a higher diversity of R1a haplotypes, representing the only site among our novel data where R1a-M458 is more frequent than R1a-CTS1211 (also dubbed: R1a-M558) (Fig. S57c).

Notably, such a differentiation is not seen in the distribution of mtDNA haplogroups. While this might be partially explained by the prevalent practice of patrilocality and female exogamy in the Slavic Period, we caution that mtDNA frequencies are generally underpowered to detect meaningful differences between sites and periods (Fig. S58b).

In contrast, we detect no such differentiation between the SP population of the Poland-Northwestern Ukraine transect and the present-day paternal gene pools of Poland (*p* = 0.4414), Ukraine (*p* = 0.07334) and Belarus (*p* = 0.07424), suggesting that the Y chromosome diversity of these populations was indeed formed during the Slavic Period and remained mostly unchanged during the last 1,000 years. In contrast, the SP population of Croatia deviates from this shared pattern. We highlight that its paternal gene pool significantly differs from the LA/MP as well as from the present-day (Croatian) population (*p* < 0.0005). In the Slavic Period, we measure a substantially lower proportion of Eastern European-derived haplogroups (38% ± 7%) than what would be expected based on the estimated autosomal SP contribution. While the SP Croatian genomes generally also show less genome-wide Eastern European ancestry than the contemporary populations in Eastern Germany and Poland, potentially indicative of a smaller influx of newcomers, this observation might as well suggest that the immigrating SP population incorporated additional Y-haplotypes from local groups while passing through the Balkan Peninsula.


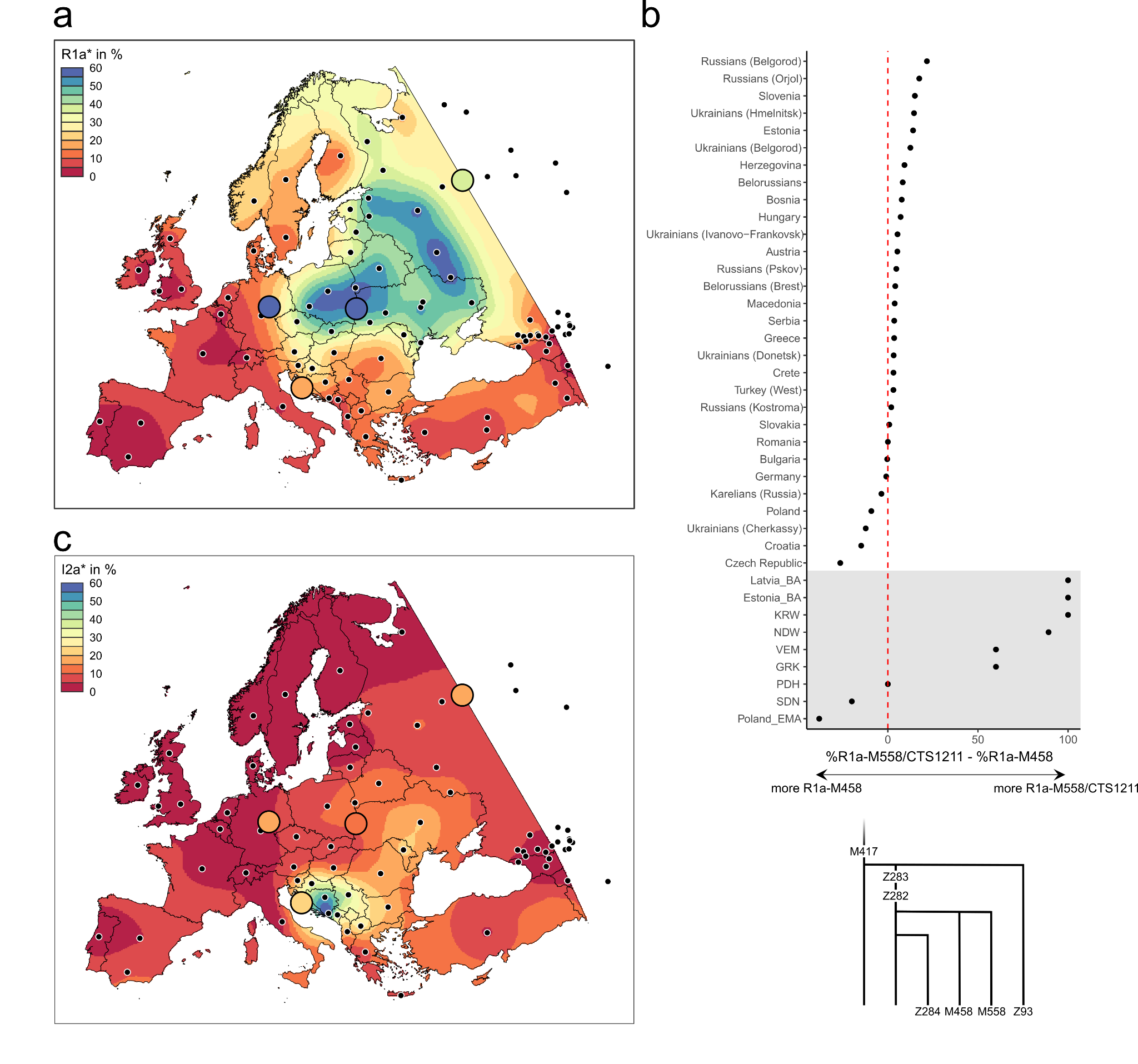


***Supplementary Figure 57. Distribution of Y chromosome haplogroups.*** *Shown are interpolated maps of population frequencies (in %) of Y haplogroups R1a (a) and I2a (c) in 92 European populations. Data was collected from Mirabal et al. 2010*[*^159^*](https://paperpile.com/c/UPmHk7/3sFXT) *and Underhill et al. 2014*[*^154^*](https://paperpile.com/c/UPmHk7/dzMYh)*. The frequencies of the SP populations from Eastern Germany, Poland-Northwestern Ukraine, Croatia and the Russian Volga-Oka region are highlighted as large dots. b) Phylogeny of R1a-M458 and R1a-M558/CTS1211 sub-haplogroups and their prevalence in ancient (n = 9) and present-day groups (n = 30). Depicted is the percentage of R1a-M558/CTS1211 minus the proportion of R1a-M458 among all R1a-M420 haplotypes.*


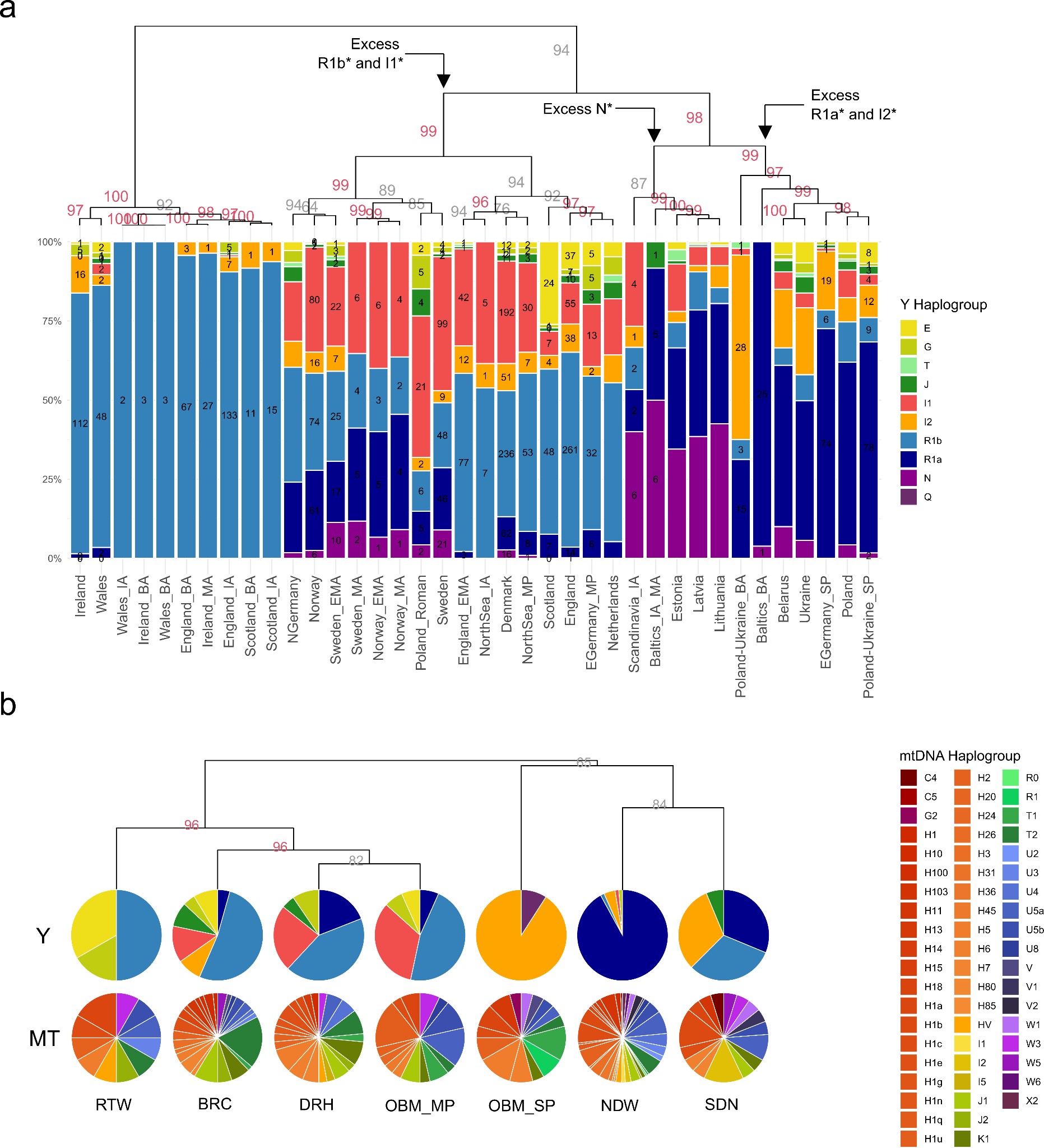


***Supp. Fig. 58. Changes in ancestry of the paternal and maternal gene pool.*** *a) Relative and absolute frequencies of Y-chromosome haplogroups across ancient (n = 23) and present-day (n = 15) male samples from Northern Europe from this and previous studies. Columns were ordered according to hierarchical cluster analysis applying Ward’s minimum variance method. The dendrogram and statistical support for the bifurcations from multiscale bootstrap resampling are shown. b) Relative frequencies of Y-chromosome and mtDNA haplogroups across pre-SP and SP samples from sites in Eastern Germany. Each pie chart represents one MP or SP site from Eastern Germany (total number of individuals: n = 15, 56, 49, 30, 22, 182, 36). Pie charts in the upper row represent Y-chromosome diversity, pie charts in the lower row mtDNA diversity. Pie charts were ordered according to hierarchical cluster analysis of Y-chromosome haplogroup proportions applying Ward’s minimum variance method. The dendrogram and statistical support for the bifurcations from multiscale bootstrap resampling are shown.*


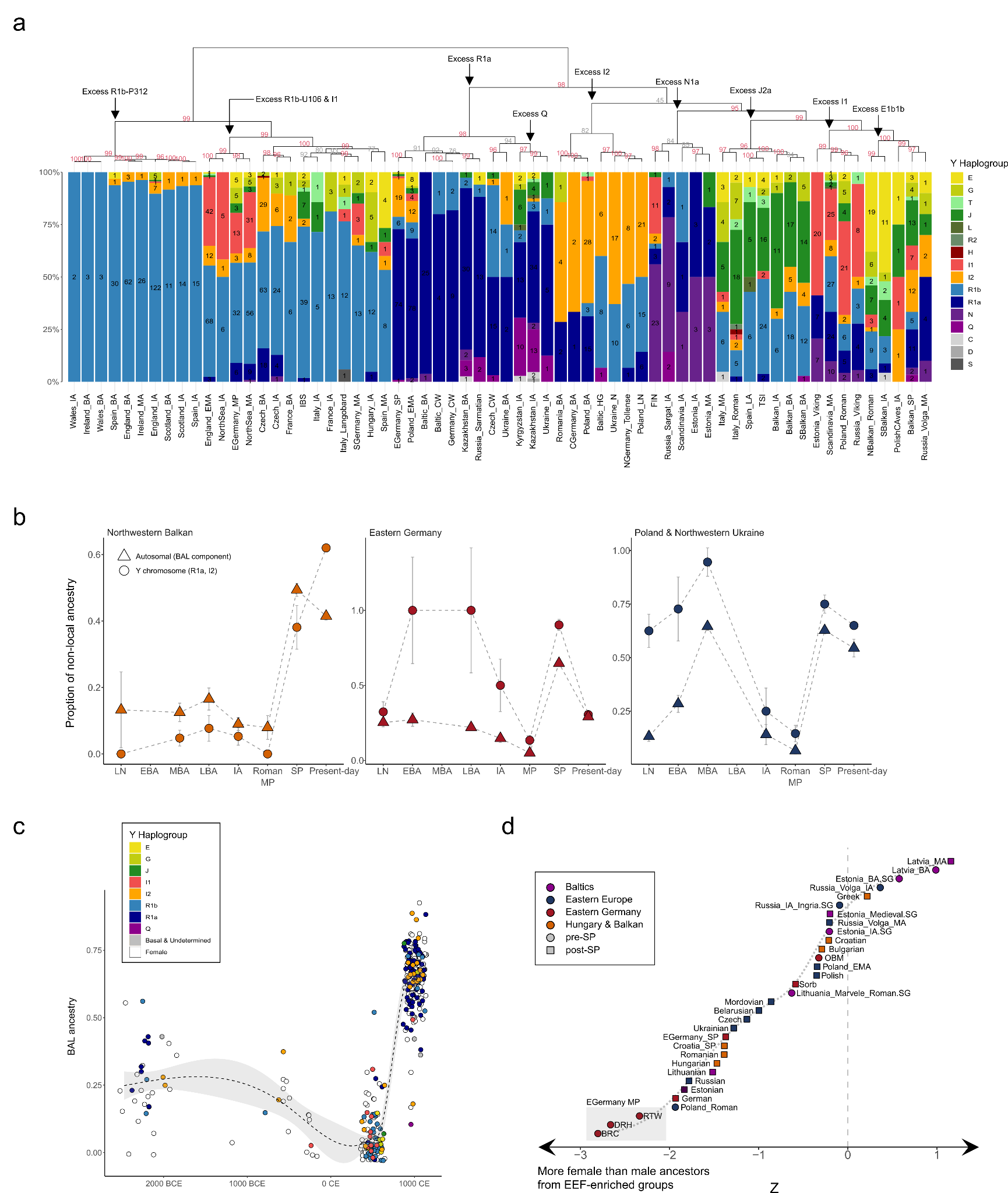


***Supp. Fig. 59. Changes in ancestry of the paternal and maternal gene pool.*** *a) Relative and absolute frequencies of Y-chromosome haplogroups across ancient male samples (n = 2533) from 347 groups reported in this and previous studies. Columns were ordered according to hierarchical cluster analysis applying Ward’s minimum variance method. The dendrogram and statistical support for the bifurcations from multiscale bootstrap resampling are shown. b) Proportions of BAL ancestry (from supervised ADMIXTURE) and R1a haplotypes in ancient and present-day males from our three study transects, Northwest Balkan (left) (n = 99), Eastern Germany (middle) (n = 226), and Poland-Northwestern Ukraine (right) (n = 261). Error bars indicate two standard errors. c) Relative proportion of the BAL ancestry component for each ancient, post-Neolithic individual from Eastern Germany (from supervised ADMIXTURE) (n = 415). White dots represent female individuals and filled dots male individuals, coloured according to their Y-chromosome haplogroup. d) Z scores for the difference between Early European Farmer ancestry on the autosomes and that on the X chromosome, when ancient and present-day populations are modeled as a mixture of WHG, EEF, and Old Steppe using qpAdm. Negative values indicate more EEF-related ancestry on the X chromosome than on the autosomes, and thus female-biased admixture process with EEF-enriched sources (e.g. from South Europe).*

### 7.2 Sex bias

To investigate if a possible sex bias in the admixture process might have contributed to the bottleneck and homogenisation in paternal ancestry, we compared the sex-specific fractions of Northeastern European BAL ancestry within our new SP samples from Croatia and Eastern Germany. We first highlight that there is no significant difference in BAL ancestry between males and females in Eastern Germany (Wilcoxon rank sum test; *W* = 5922, *p* = 0.5493). SP males carry 0.002% less autosomal BAL ancestry than females on average (95% CI: -0.027% - 0.0315%). A similar pattern was also observed in Velim, Croatia. There, SP males carry 0.006% more autosomal BAL ancestry than females on average (95% CI: -0.064% - 0.0535%).

We further observe no significant difference in the distribution of admixture across sexes in both study areas (Two-sample Kolmogorov-Smirnov test; *D* = 0.1073, *p* = 0.5456 and *D* = 0.15446, *p* = 0.7515 for Eastern Germany and the Northwestern Balkan, respectively). Neither females nor males appear more often admixed (10% < BAL < 50%) or unadmixed (BAL < 10% or BAL> 50%) in absolute counts (Fisher's Exact Test for Count Data; *p* = 0.5951 and *p* = 0.2142 for Eastern Germany and the Northwestern Balkan, respectively).

However, these tests are limited in their power to detect signals of less recent admixture. Thus, we compared estimates of SP ancestry on the X-chromosomes and the autosomes to identify proportion differences indicative of male biased admixture. For that, we selected 7 individuals from Gródek (the oldest Slavic site in our dataset, with individuals dating between the 7^th^ and 9^th^ century CE) in Eastern Poland to represent the non-admixed SP gene pool. As proxies for the pre-SP gene pool in Eastern Germany, Poland-Northwestern Ukraine, the Northwestern Balkan and the Volga-Oka region, we selected EGermany_MP, Poland_Roman Croatia_Roman or Russia_VolgaOka_IA, respectively (Table S51). Then, we applied qpAdm to calculate estimates of the mixture coefficients α_SP_ and α_pre-SP_ on the autosomes (default option) and on the X-chromosome (option “chrom: 23”) using the outgroups described in the Methods section. To quantify potential sex bias, we used the formula described in Mathieson et al. 2018 which compares the SP ancestry proportions on the autosomes (*pA*) and the X-chromosome *pX*)[^184^](https://paperpile.com/c/UPmHk7/Rnj63). Thus, a positive *Z*-score means that there is more SP admixture on the autosomes than on the X chromosome, indicating that the SP admixture was male-biased. While we calculate positive *Z* scores for all four study transects, none of these are significant (*Z* = 0.031, 0.511, 0.85, 0.268 and *p* = 0.975, 0.61, 0.395, 0.789 for EGermany_SP, Poland-NorthwesternUkraine_SP, NorthwesternBalkan_SP, and Russia_VolgaOka_MA, respectively) (Fig. 4c, Table S47).

Interestingly, this deviates from the pattern observed in MP Eastern Germany. For Brücken, Deersheim, Obermöllern and Rathewitz, we calculated the fraction of (local) Northwestern European ancestry (approximated by Roman Iron Age genomes from Häven[^123^](https://paperpile.com/c/UPmHk7/tySmG)) and (incoming) Southeastern European ancestry (approximated by Italy_Imperial.SG[^112^](https://paperpile.com/c/UPmHk7/PvwZ8)) on the autosomes and X-chromosome and found evidence that the Southeastern European admixture was significantly female biased (*Z* = -2.517, -2.449, -1.372, -2.216 and *p* = 0.012, 0.014, 0.17, 0.027 for Brücken, Deersheim, Obermöllern and Rathewitz, repetitively. This suggests an excess of females over males with Southern European origin among their ancestors (Fig. 4c, Table S47).

Our estimate of change in genome-wide ancestry in Central Europe is supported by independent evidence for population turnover from uniparental markers. Before the advent of Slavic groups in Central Europe, males from the three sampling regions exhibited a large diversity of different Y-chromosome haplogroups, mirroring the heterogeneity observed in the autosomal DNA. In the Northern Balkans, the Mediterranean supra-haplogroups E-V68, J-M172, and G-P15 represent the majority of haplotypes during the Iron Age and Roman Period, while in Eastern Germany and Poland most males carried haplogroups more frequent in Northwestern Europe such as R1b-M343 and I1-M253 (summed up to 67% and 54% in Germany and Poland respectively) (Fig. S57-59).

Contrary to this, the SP males from Eastern Germany and Poland exhibited less variation in paternal lineages and instead featured a substantial fraction of Eastern Europe-derived haplotypes belonging to major haplogroups R1a-M420 and I2-M438. In particular Y-chromosome haplogroup R1a-M420, which was nearly absent in Iron Age and Roman Period males from Eastern Germany (9% ± 3%) and Poland (10% ± 2%), but was identified in more than 72% ± 5% and 65% ± 5% of the later SP populations respectively. Altogether, haplogroups (nearly) absent in Roman and MP Eastern Germany and Poland (namely R1a-M420 and I2-M438) represent at least 90% ± 4% and 75% ± 4% of the Y-chromosomes in our SP sample from these two regions, mirroring the turnover estimates from autosomal data and evidencing a major influx of males from the East (Fig. 4c).

Regarding potential sex bias in the admixture process between immigrating Eastern European and local ancestries, we consider that our proximal qpAdm model presented in the main text may be prone to inaccuracy as the involved ancestries are highly similar. As a consequence, we take advantage of the fact that the MP and SP populations of all three study regions differ considerably in their ancestral ancestry components. In particular, the pre-SP groups exhibit less Western Hunter-Gatherer (WHG) and Steppe pastoralist but more Early European farmer (EEF) ancestry. Consequently, differences in EEF ancestry on the autosomes and X-chromosome are indicative of sex biased admixture. Since these ancestries are deeply divergent, they can be precisely measured[^120^](https://paperpile.com/c/UPmHk7/iIPiM). Using this distal approach, we obtain results similar to the proximal modeling (*Z* = -1.369, -0.345, -1.386, -0.205 for EGermany_SP, Poland-NorthwesternUkraine_SP, Northwestern_Balkan_SP, and Russia_VolgaOka_MA, respectively) (Table S48). Thus, both models provide no evidence for sex biased admixture in SP populations. In summary, while we cannot exclude subtle levels of sex bias during the admixture, our results are overall consistent with a model of no sex bias, suggesting that the newcomers from the East included both men and women who mixed at similar levels with the local population (Fig. S59d, Table S48).

This observation contrasts with evidence for sex-biased admixture in the preceding Migration Period in Eastern Germany, where we demonstrate that significantly more females than males with excess EEF ancestry admixed with the local population (*Z* = -2.804, -2.662, -0.325, -2.338 for Brücken (BRC), Deersheim (DRH), Obermöllern (OBM), and Rathewitz (RTW) (Fig. S59d, Table S48).

## 8. Changes in social structure

### 8.1 Grave goods

As described earlier, we identify considerable genetic diversity within the Eastern German MP population. While the majority of the ancient genomes cluster closely together with present-day Northwestern European individuals as well as preceding Iron Age genomes from Sachsen-Anhalt, most likely representing the local Eastern German gene pool, a considerable portion of individuals shows significantly closer affinity to Southern Europeans or is located on an admixture cline between both sources. Interestingly, we highlight that multiple of these individuals with excess Southern European ancestries have relatives at the sites who have less Southern but substantially more Northern European ancestry indicative of recent admixture with the local population (although we highlight that contemporaneous groups from Northern Germany and Denmark show a highly similar genetic make-up to MP genomes from Eastern Germany, rendering it difficult to identify potential newcomers from these regions further north). Thus, we can conclude that these two genetically-differentiated groups (Central/Northern European locals and Southern European newcomers) did not remain separated but reproduced regularly and mixed with each other (Fig. S60a,b). This is further supported by the fact that we cannot detect significant physical segregation of individuals with Southern and Northern European ancestry at the cemetery of Brücken (for which complete spatial information was available). Specifically, we calculated euclidean distances from the ADMIXTURE ancestry profiles of all individuals and compared for each pair the genetic and spatial distances between the graves (Mantel statistic based on Spearman's rank correlation rho using 9999 permutations; Mantel statistic *r* = 0.05655, *p* = 0.1166). Similar patterns of repeated admixture between incoming and local ancestries were also detected in other MP sites in England, Hungary and Italy[^123,185,186^](https://paperpile.com/c/UPmHk7/q0ozB+tySmG+zrW2X), yet often in association with a spatial separation between burials of different genetic backgrounds.

To examine to what extent the broader biological backgrounds of individuals (as determined by genomic ancestry) beyond close biological kinship were acknowledged as meaningful social ties, we also compared burial customs (demonstrating variation) with PC1 coordinates (reflecting North-South genetic differentiation) using a logistic regression framework (Fig. S60c, Table S4).

In contrast to previous studies of MP societies in England, Italy and Hungary[^123,185,186^](https://paperpile.com/c/UPmHk7/q0ozB+tySmG+zrW2X), we observe no significant association between the presence of various jewelry types, weapons, and dress accessories and genetic variation. We found the only significant association between PCA results and pit graves (*p* = 0.0309). Interestingly, other burial constructions such as coffin or wood-covered graves featured no statistically significant association (*p* = 0.0853 and *p* = 0.2185 , resp.).

Thus, these results point to individuals with Southern European genetic backgrounds being treated similar in death to individuals with local Central/Northern European ancestry by their respective communities, perhaps marking abolishment of cultural and/or social differences between locals and more recent migrants in the region. Otherwise, the choice of burial items might have been a more selective process on the level of an individual or their family rather than on a societal level, revolving around individual life histories rather than geographic origin.

We also used correspondence analysis (CA) to explore the relationships between different combinations of grave goods and burial construction types, and the individuals who had them (Fig. S60d). We find that Dimension 1 mostly differentiates male (based on weapons such as swords, arrows, spears, axes but also riding equipment and chamber graves) and female individuals (based on brooches, jewelry, beads, and spindles), with burials considered displaying high social status being located at the ends of the clines. On the other hand, Dimension 2 mostly separates low status from high status burials (based on the presence/absence of grave goods but also grave construction type, with low status individuals featuring predominantly pit graves). In congruence with our logistic regression framework, we observe that individuals with excess Southern European ancestry (indicated by low PC1 values) are scattered across the full extent of the clines. Specifically, we find individuals with excess Southern European ancestry both in the diversity of high status and low status burials. Multiple female individuals with Southern European-related ancestry were buried with local/Northern European brooches and jewelry. Furthermore, we find Southern European (admixed) male individuals that were buried with weapons, deviating from the pattern observed in MP Italy or Hungary[^185^](https://paperpile.com/c/UPmHk7/q0ozB). Instead, our results are in line with previous research of MP communities in England, where both locals and immigrants were buried with weaopns[^123^](https://paperpile.com/c/UPmHk7/tySmG), a pattern disagreeing with historical sources[^187^](https://paperpile.com/c/UPmHk7/676dD).

Since grave goods are generally sparse and homogenous in Niederwünsch and Steuden (Table S5), we compared these results with the SP population in Velim, Croatia (Table S6). Similar to the Eastern German MP sites, we detect notable diversity in regard to Northeastern and Southern European ancestry in Velim (Fig. S61c). Thus, we conducted logistic regression comparing grave goods/burial construction type to our PCA results among the 67 sequenced individuals (Fig. S61a). Again, we find no significant difference within the treatment of individuals according to their ancestry (no *p* < 0.05). This is also visually confirmed in correspondence analysis where we detect three clusters (Fig. S61b): cluster 1 contains two high status female individuals buried with beads and jewelry, cluster 2 contains low status pit graves in North-South orientation, and cluster 3 contains both high and low status burials all in East-West orientation. Indicatively, individuals with excess Southern European ancestry (indicated by lower PC1 values) are found in all three clusters. Furthermore, we cannot detect significant physical segregation of individuals with Southern and Northeastern European ancestry at the cemetery when comparing spatial and genetic distances (obtained from ADMIXTURE analysis) between pairs of individuals (Mantel statistic based on Spearman's rank correlation rho using 9999 permutations; Mantel statistic *r* = -0.02417, *p* = 0.6794).


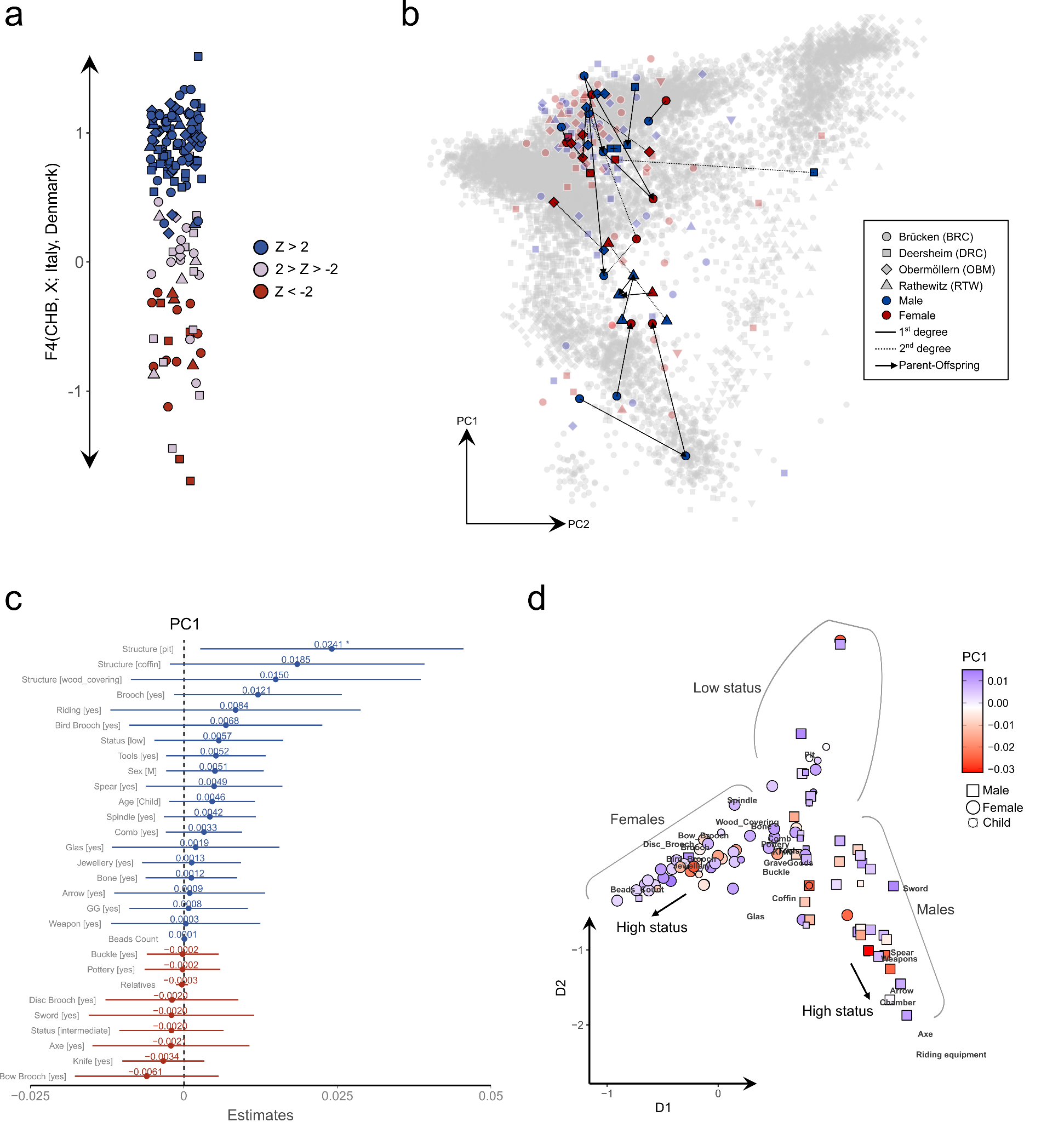


***Supp. Fig. 60. Comparing genetic and archaeological data from MP Eastern Germany.*** *a) Shown are the results of the F_4_-statistic of the form F_4_(CHB, Test; Italy, Denmark) for MP individuals from Eastern Germany (n = 181). b) MP individuals from Brücken (BRC, n = 56), Deersheim (DRH, n = 49), Obermöllern (OBM, n = 30), and Rathewitz (RTW, n = 15) projected onto the modern variation. 1^st^ and 2^nd^ degree genetic relationships are indicated with lines between individuals. If a 1^st^ degree relationship is vertical, the direction is indicated from the parent to the offspring with an arrow. Males are shown in blue, females in red. c) Estimate size plot of a generalized linear model (poisson glm) testing the effects of individual and grave traits (sex, number of relatives, grave construction, grave goods etc) on PC1 position in our European PCA of Roman/LA individuals from Eastern Germany. The asterisks indicate p < 0.05. d) Correspondence analysis of the counts of different grave goods types per individual. Dimension 1 differentiates between males and females, with graves considered representing higher status individuals being located on the extremes of the cline. Dimension 2 differentiates between graves of higher and lower social status. Males are shown as squares, females as circles. Sub-adult individuals are indicated with smaller symbols. The symbols are colored according to their PC1 position in our European PCA.*


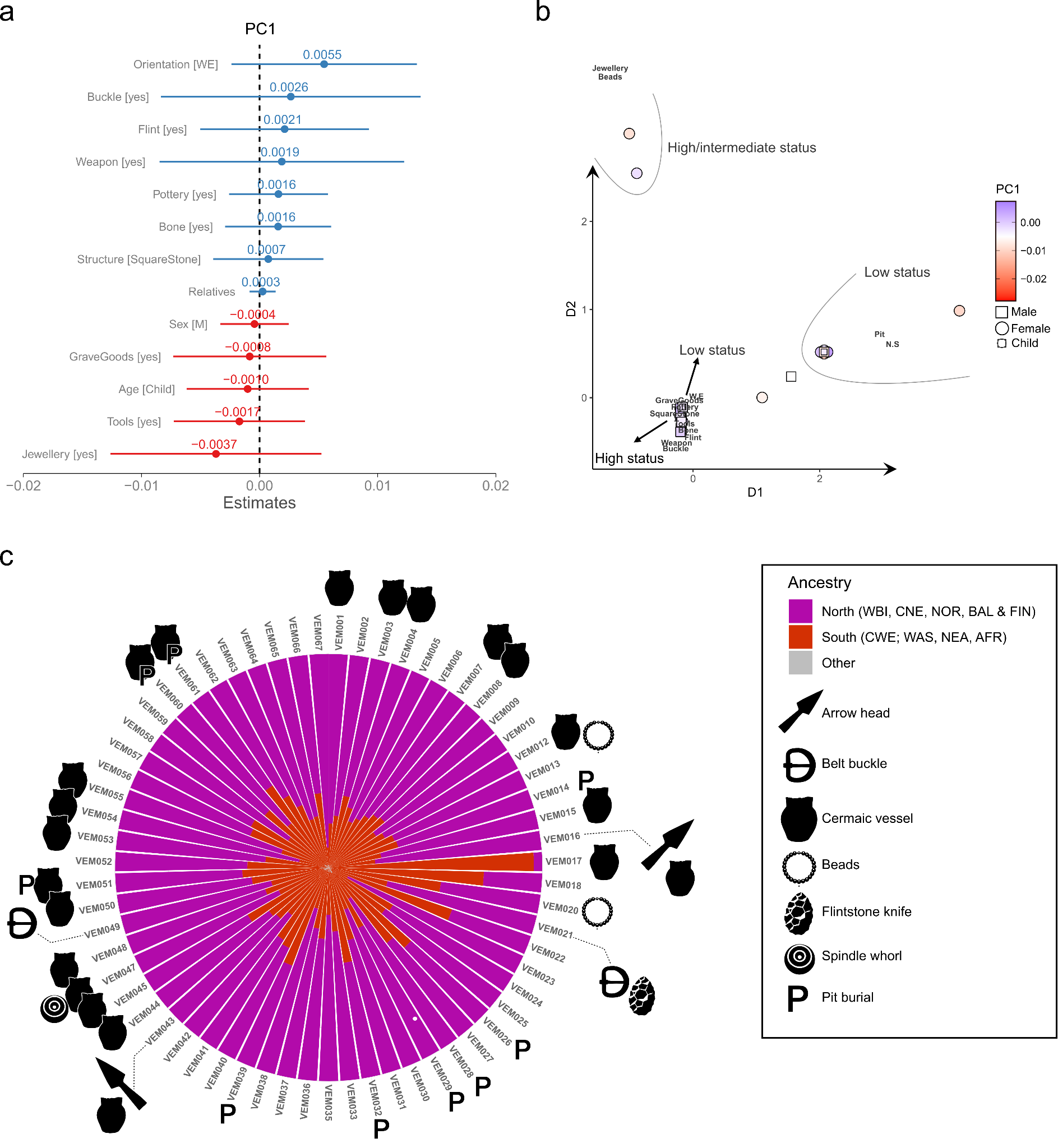


***Supp. Fig. 61. Comparing genetic and archaeological data from SP Velim, Croatia.*** *a) Estimate size plot of a generalized linear model (poisson glm) testing the effects of individual and grave traits (sex, number of relatives, grave construction, grave goods etc) on PC1 position in our European PCA of SP individuals from Velim, Croatia (n = 67). No p-values < 0.05 were identified. b) Correspondence analysis of the counts of different grave goods types per individual. Dimension 1 differentiates between graves considered representing higher and lower status individuals. Dimension 2 differentiates between graves with and without jewelry/beads and individuals buried in pits or square stone graves . Males are shown as squares, females as circles. Sub-adult individuals are indicated with smaller symbols. The symbols are colored according to their PC1 position in our European PCA. c) Schematic distribution of different artifact types among the Velim burials. The respective ancestry composition of each individual in respect to northern (WBI, CNE, NOR, BAL & FIN) and southern ancestry (CWE, WAS, NEA & AFR) (from supervised ADMIXTURE) is indicated as a circular bar chart.*

### 8.2 Spatial Structure

We next investigated the spatial organization of the cemeteries (for which spatial data was available) by measuring the physical and genetic distance between pairs of individuals. Within all four sites, Brücken (BRC), Niederwünsch (NDW), Steuden (SDN), and Velim (VEM), we observe significantly closer spatial proximity between pairs of closely related individuals (closer than 4^th^ degree) compared to non-(closely)-related individuals (Fig. S62-65):

i) For Brücken, we find that closely-related pairs of individuals are buried on average 11 m (95% CI: 7.24 m - 14.87 m) closer to each other than non-(closely)-related individuals (Wilcoxon rank sum test with continuity correction; *W* = 34287, *p* = 1.812e-07) (Fig. S65a).

ii) For Niederwünsch, we find that closely-related pairs of individuals are buried on average 4.05 m (95% CI: 3.55 m - 4.56 m) closer to each other than non-(closely)-related individuals (Wilcoxon rank sum test with continuity correction; *W* = 5633320, *p* < 2.2e-16) (Fig. S65b).

iii) For Steuden, we find that closely-related pairs of individuals are buried on average 8.17 m (95% CI: 5.77 m - 10.6 m) closer to each other than non-(closely)-related individuals (Wilcoxon rank sum test with continuity correction; *W* = 11778, *p* = 5.503e-14) (Fig. S65c).

iv) For Velim, we find that closely-related pairs of individuals are buried on average 7.34 m (95% CI: 3.41 m - 11.34 m) closer to each other than non-(closely)-related individuals (Wilcoxon rank sum test with continuity correction; *W* = 41140, *p* = 0.000269) (Fig. S65d).

This pattern is reflected within the cemeteries of Brücken, Steuden and Velim in the form of small, definite and non-overlapping burial groups of biological relatives that often consist of parents and their offspring or pairs of siblings (Fig. S62-64). In contrast, in Niederwünsch, the burial groups are less defined and located in close spatial proximity to each other, with some degree of overlap (Ex. Fig. 8). However, this is most likely related to the considerably larger pedigrees and an overall higher degree of biological relatedness among the buried population in Niederwünsch, especially compared to Brücken and Velim where all pedigrees do not last more than 3 generations.

Interestingly, when comparing the overall structure of the cemeteries, we highlight that in the Slavic Period cemeteries of Niederwünsch, Steuden, and Velim spatial distances between graves are significantly correlated with the genetic distances between buried individuals. This notably deviates from the layout of Brücken, where we do not find such a relationship. Specifically, we record for the sites (Fig. S65a-d):

i) Brücken: Mantel statistic based on Spearman's rank correlation rho using 9999 permutations; Mantel statistic *r* = -0.02073, *p* = 0.6332.

ii) Niederwünsch: Mantel statistic based on Spearman's rank correlation rho using 9999 permutations; Mantel statistic *r* = 0.09517 , *p* = 1e-04.

ii) Steuden: Mantel statistic based on Spearman's rank correlation rho using 9999 permutations; Mantel statistic *r* = 0.2653, *p* = 1e-04.

ii) Velim: Mantel statistic based on Spearman's rank correlation rho using 9999 permutations; Mantel statistic *r* = 0.1044, *p* = 1e-04.

This might indicate that SP cemeteries across Central Europe, in contrast to their MP predecessors, were planned and constructed around (biological) kin groups, despite the substantial differences in overall biological relatedness among the buried populations of the sites.

Comparing the spatial distances between graves and their degree of genetic relatedness within the SP sites of Steuden and Niederwünsch, we observed that sisters, mothers and their daughters as well as fathers and their daughters are on average buried closer together than any other 1^st^- or 2^nd^-degree relatives, a pattern that is evident whether considering only adults or both adults and subadults (Fig. S66). This is consistent with the overall pattern of related females being buried closer to each other than related males (on average 2.5 meters closer, i.e. 4.21 m vs. 6.71 m) (Wilcoxon rank sum test; W = 3071.5, p = 5.058e-05). We further observe on average closer spatial proximity between sister, mother–daughter and father–daughter pairs than any other related pair. Moreover, with the exception of aunt-niece relationships, all pairings of female relatives tend to be on average spatially closer to each other than all pairings of male relatives, including brother, father–son and uncle–nephew pairs. Yet, in the case of sisters, we note that we do not identify any pair of adult sisters, highlighting the exogamous practices of the NDW and SDN communities. Consequently, we suggest that women of genetically exogenous provenance were spatially integrated into their reproductive partner’s burial area, indicative of social integration into the host group. Finally, we observe that half-siblings tend to be on average buried closer to each other than full brother and brother–sister pairs, perhaps suggesting that their relatedness was considered equal to full siblings. However, this is potentially also related to the overall high fraction of subadult half-siblings in our sample.


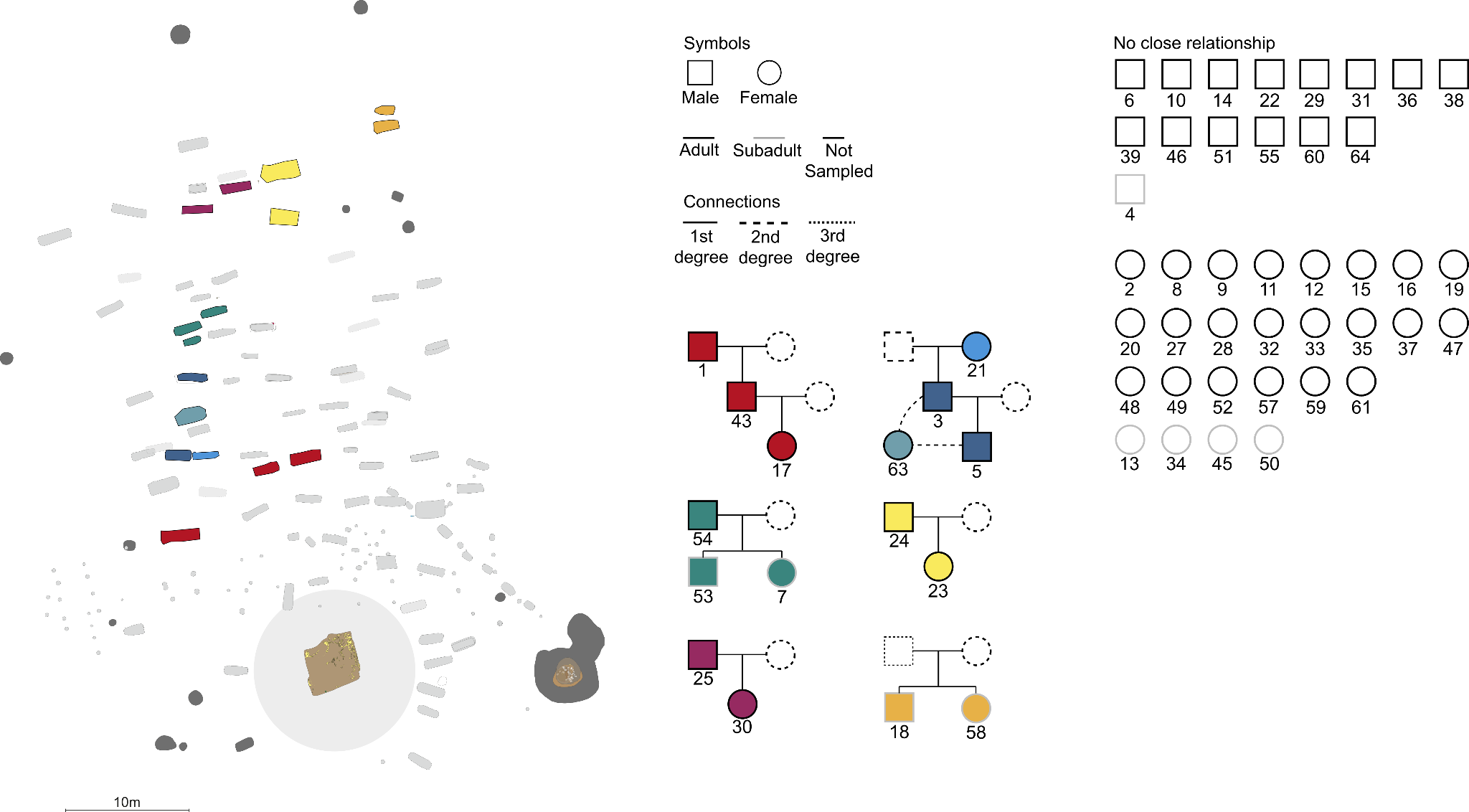


***Supp. Fig. 62. Cemetery and family structure in Eastern Germany during the Migration Period.*** *Reconstructed pedigrees of Brücken, coloured according to family lineages. The site layout is shown on the left, representing the spatial distribution of family lineages coloured as in the pedigrees. Numbers correspond to the Genetic IDs listed in Supplementary Table 1.*


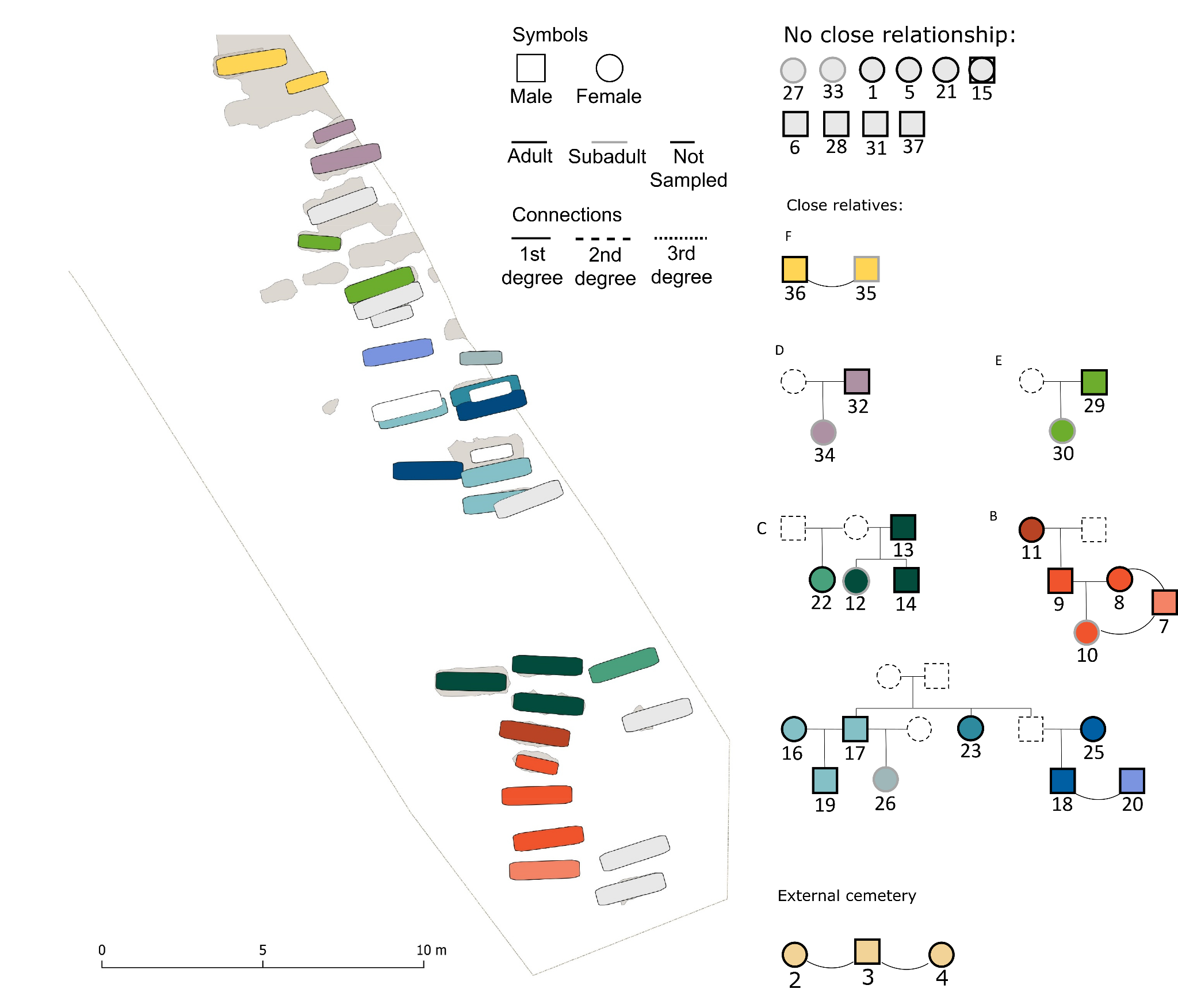


***Supp. Fig. 63. Cemetery and family structure in Eastern Germany during the Slavic Period.*** *Reconstructed pedigrees of Steuden, coloured according to family lineages. The site layout is shown on the left, representing the spatial distribution of family lineages coloured as in the pedigrees. Numbers correspond to the Genetic IDs listed in Supplementary Table 1.*


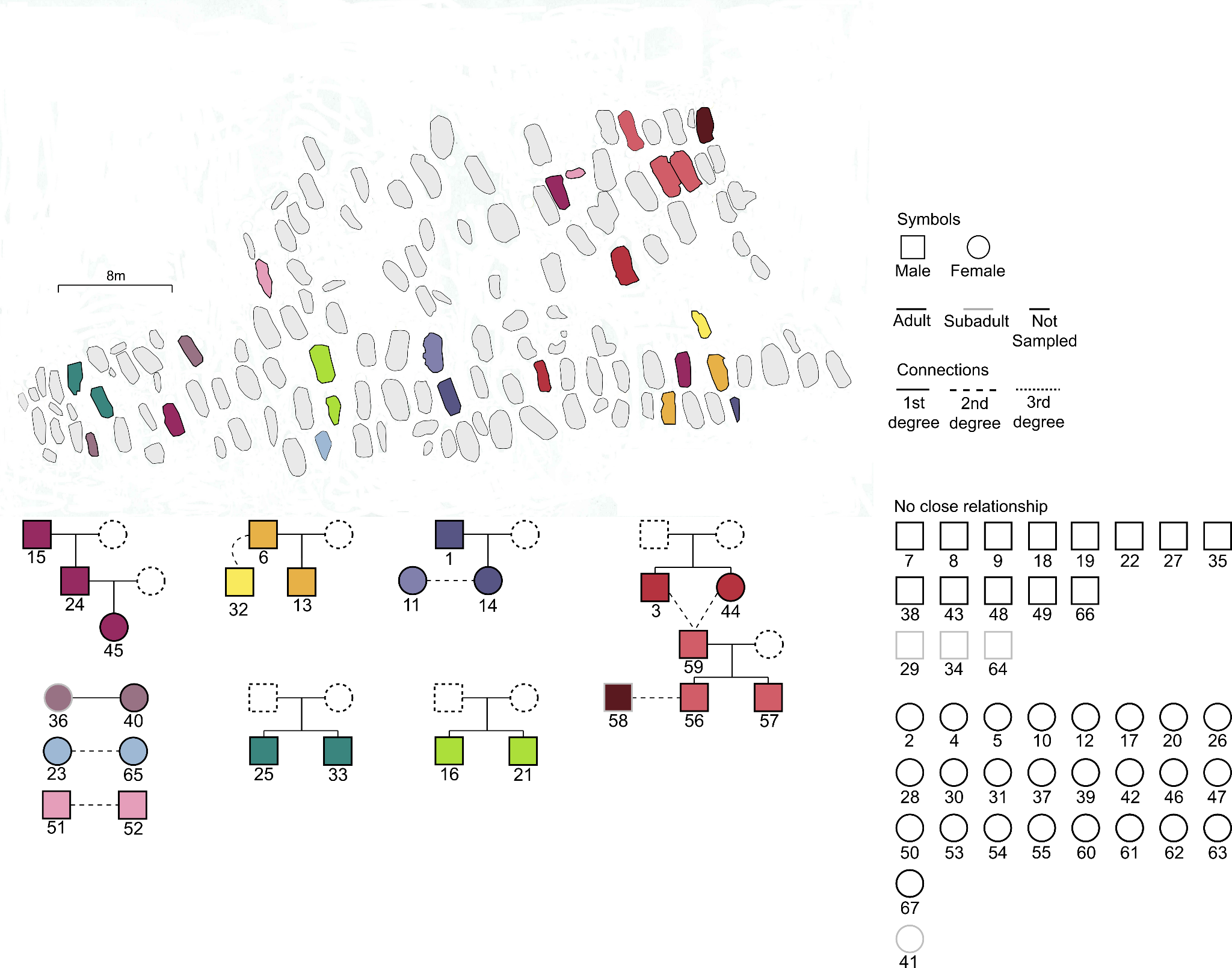


***Supp. Fig. 64. Cemetery and family structure in Croatia during the Slavic Period.*** *Reconstructed pedigrees of Velim, coloured according to family lineages. The site layout is shown on the left, representing the spatial distribution of family lineages coloured as in the pedigrees. Numbers correspond to the Genetic IDs listed in Supplementary Table 1.*


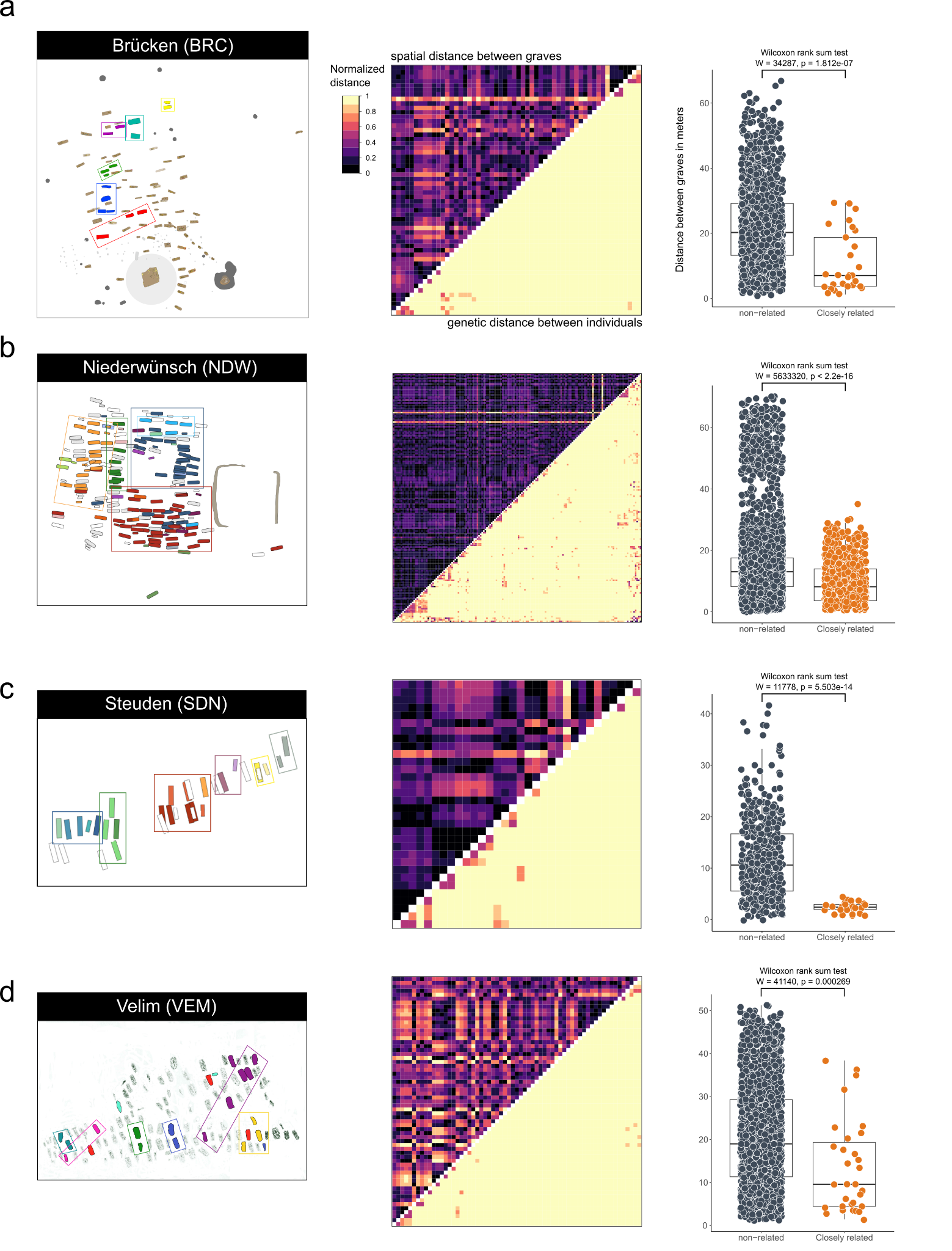


***Supp. Fig. 65. Correspondence between genetic and spatial distances.*** *Shown are i) the cemetery layouts of the sites, with groups of genetically-related individuals coloured accordingly (left), ii) heatmaps of normalized spatial (upper left triangle) and genetic distances (lower right triangle) between burials (middle), and iii) boxplots of spatial distances between pairs of closely-related (≤ 3^rd^ degree) and non-related burials (> 3^rd^ degree) (right). a) for Brücken (BRC, n = 56), Eastern Germany. b) for Niederwünsch (NDW, n = 182), Eastern Germany. c) for Steuden (SDN, n = 36)), Eastern Germany. d) for Velim (VEM, n = 67), Croatia.*

*
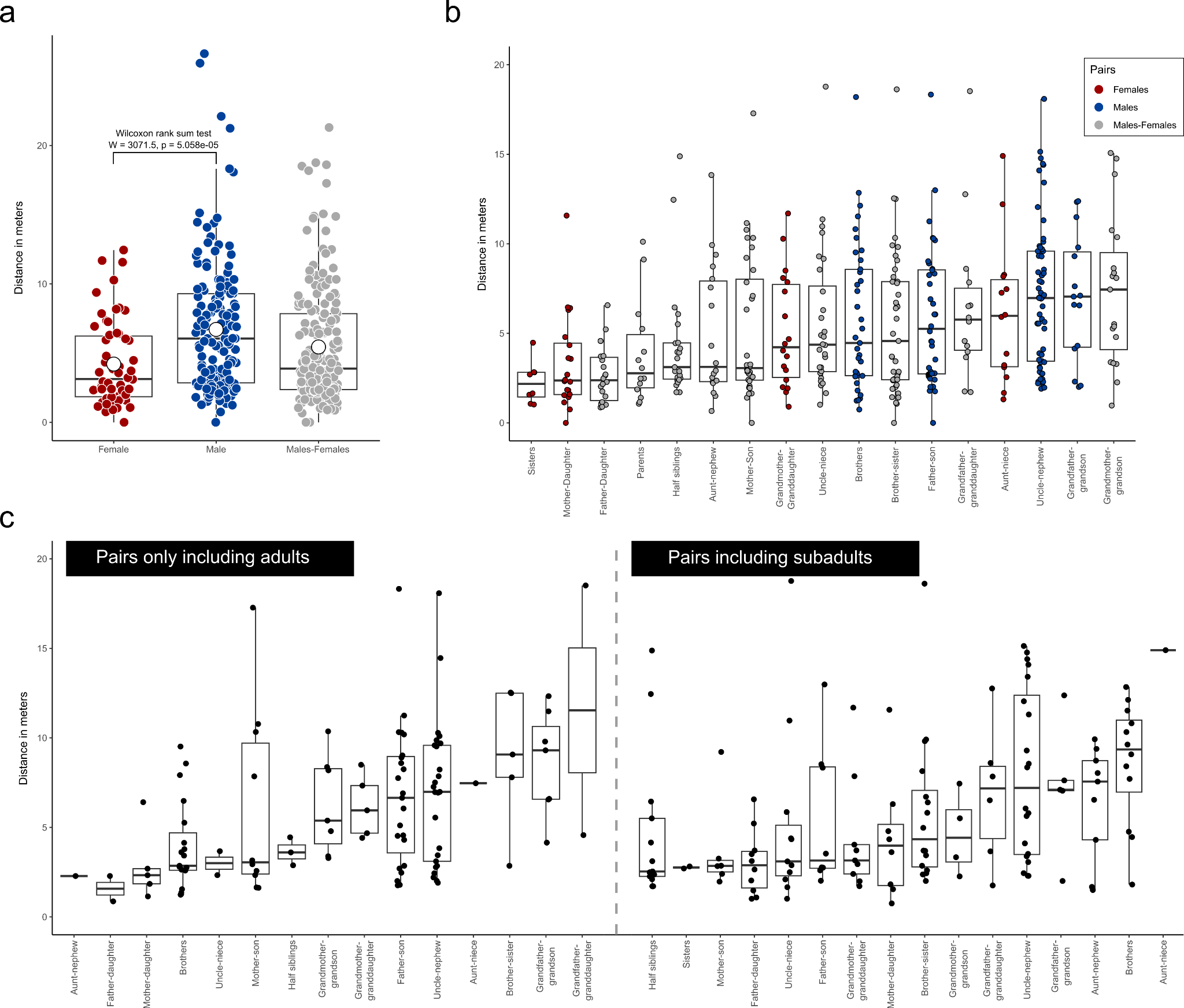
*

***Supp. Fig. 66. Correspondence between genetic relatedness and spatial distances.*** *a) Shown are spatial distances between male, female, and mixed pairs of 1^st^ and 2^nd^ degree related individuals from NDW and SDN as boxplots (n_m_ = 97, n_f_ = 121). b) Boxplots of spatial distances between pairs of individuals according to the type of their 1^st^ and 2^nd^ degree genetic relationships. Additionally, parents are also included in the comparison. Only pairs of individuals were considered for which the type of relationship was securely determined. c) Same data as shown in panel b) but subsetted for pairs of relatives that only include adult individuals (left) or pairs that include at least one subadult individual (right), respectively.*

### 8.3 Relatedness

As presented in the main text, the majority of the identified offspring in the SP sites of Steuden and Niederwünsch were male individuals, pointing towards additional unsampled female siblings (to statistically account for an equivalent number of females born) as well. Notably, for all unions (for which at least one parent was identified at the site), we find 52 sons (95% CI: 51-72%) yet only 32 daughters (95% CI: 28-49%) (exact binomial test; *p* = 0.03753). This skewed ratio of 1.63:1 strongly deviates from the natural male/female sex ratio at birth of 1.05:1. This imbalanced ratio is partially corrected by the unrelated individuals of which the majority are adult females (*n*_female_ = 25 vs *n*_male_ = 11) (Exact binomial test; *p* = 0.02882). Taken together, these observations suggest a practice of female exogamy.

To formally test the relatedness of individuals and therefore possible signs of matri- or patrilocality/lineality, we performed an analysis of variance of the pairwise mismatch rates (PMMR) estimated using BREADR using the genetic sex of a pair of individuals as predictors (Fig. S67d,e). When looking at the PMMR between different genetic sex pairings (XY/XY, XY/XX, XX/XX), we find significantly lower PMMR for XY/XY pairs when compared to XX/XX and XX/XY pairs (0.2537261 vs 0.2553185 for XY/XY vs XX/XX pairs). This pattern demonstrates that male individuals are on average more closely related to each other than they are to females, or than female individuals are to each other. This result is furthermore supported by the number of close relatives estimated for each male and female individual at the sites (Welch Two Sample t-test; *t* = 9.0374, *df* = 6063.9, *p* < 2.2e-16). Not only is the number of males that have any close relative (> 4^th^ degree) at the same site significantly higher than the number of females (Fisher's Exact Test for Count Data; *p* = 0.008353) (Fig. S67e), they do also feature more close relatives in the same sites than females (9.88 vs 4.81) (Wilcoxon rank sum test with continuity correction; *W* = 2087.5, *p* = 7.771e-08) (Fig. S67f). Overall, males tend to have 5 more close relatives at the site than females (95% CI: 3-6).

When comparing also more distant relationships via sharing of IBD fragments longer than 12 cM, we find a pattern consistent with the evidence of PMMR. Specifically, 46.9%, 28.1% and 22.9% of all intrasite IBD within Niederwünsch, Steuden and Obermöllern is shared among XY/XY pairs, yet only 12.7%, 5.2% and 19.8% between XX/XX pairs, evidencing substantially lower relatedness between pairs of females compared to pairs of males (Ex. Fig. 9c). On the other hand, on the supraregional level, pairs of males share substantially fewer IBD (14.8%, 6.7% and 0% of all intersite IBD between pairs from NDW-SDN, NDW-OBM, and SDN-OBM, respectively) compared to females (39.2%, 29% and 29.5% of all intersite IBD between pairs from NDW-SDN, NDW-OBM, and SDN-OBM, respectively) (Ex. Fig. 9c). Thus, while males were more connected within each site, females exhibited considerably stronger intersite biological connectedness.

Interestingly, we observe a similar but not identical pattern of sex-biased mobility indicative for patrilineality and patrilocality also at the SP site of Velim in Croatia (Fig. S67g-i). Similar to the SP sites in Eastern Germany, PMMR is significantly lower in XY/XY pairs compared to XX/XX (and XX/XY) pairs (0.2560145 vs 0.2572529 for XY/XY vs XX/XX pairs) (Welch Two Sample t-test; *t* = 2.9302, *df* = 697.24, *p* = 0.003498). This is again also reflected in a higher number of males with relatives at the sites (Fisher's Exact Test for Count Data; *p* = 0.02723) as well as a significantly higher number of biological relatives (1.38) compared to females (0.69) (Wilcoxon rank sum test with continuity correction; W = 394, *p* = 0.02585). While we identify relatively fewer biological relatives in Velim compared to Niederünwsch or Steunden, we infer for all unions (for which at least one parent was identified at the site) again a surplus of sons compared to daughters. Specifically, we identify 4 sons yet only 2 daughters. When also including pairs of siblings for which no parents could be identified at the site, these numbers increased to 9 and 3 for sons and daughters, respectively. Also similar to the SP sites in Eastern Germany, the majority of unrelated individuals are adult females (n_female_ = 25 vs n_male_ = 13)(Exact binomial test; *p* = 0.07295).

Notably, this contrasts with results obtained for the MP sites in Eastern Germany where we find a significantly lower PMMR rate in XY/XY pairs (0.2574410) compared to XX/XX pairs (0.2577479) (Welch Two Sample t-test; *t* = 2.3454, *df* = 3705.8, *p* = 0.01906) yet not difference in the absolute number of males and females with relatives at the sites (Fisher's Exact Test for Count Data; *p* = 0.7411) or in the average number of relatives per male/female individual (0.96 vs 0.424 for males and females, respectively) (Wilcoxon rank sum test with continuity correction; *W* = 305, *p* = 0.1422) (Fig. S67a-c). Specifically, we only detect a significant difference in PMMR when comparing individuals across all sites, yet there is not significant differentiation for the individual sites of Brücken, Deersheim, Obermöllern and Rathewitz (Welch Two Sample t-test;  *t* = 1.759, *df* = 277.82, *p* = 0.07968; *t* = 0.67965, *df* = 379.9, *p* = 0.4971; *t* = 1.7451, *df* = 12.061, *p* = 0.1064; *t* = 0.63554, *df* = 223.84, *p* = 0.5257, for BRC, DRH, RTW and OBM respectively). Moreover, while we identify more sons (n_sons_ = 10) than daughters (n_daughters_ = 5) across unions (for which at least one parent was identified at the site), this difference is not significant (exact binomial test; *p* = 0.3018) and becomes even less pronounced when considering siblings without parents at the site (n_sons_ = 13 vs n_daughters_ = 8) (Exact binomial test; *p* = 0.3833). At Brücken, the ratio of males (*n* = 14) and females (*n* = 22) across the adult unrelated individuals is similar to the SP sites of Eastern Germany (1.6:1) but again (due to the small sample size) not significantly different (Exact binomial test; *p* = 0.243). This might indicate that while MP communities in Eastern Germany practiced patrilocality, the frequency of female exogamy was less substantial compared than during the following Slavic Period. Additionally, the difference in site occupation duration between the MP and SP might limit our ability to detect signals of female exogamy during the Migration Period.


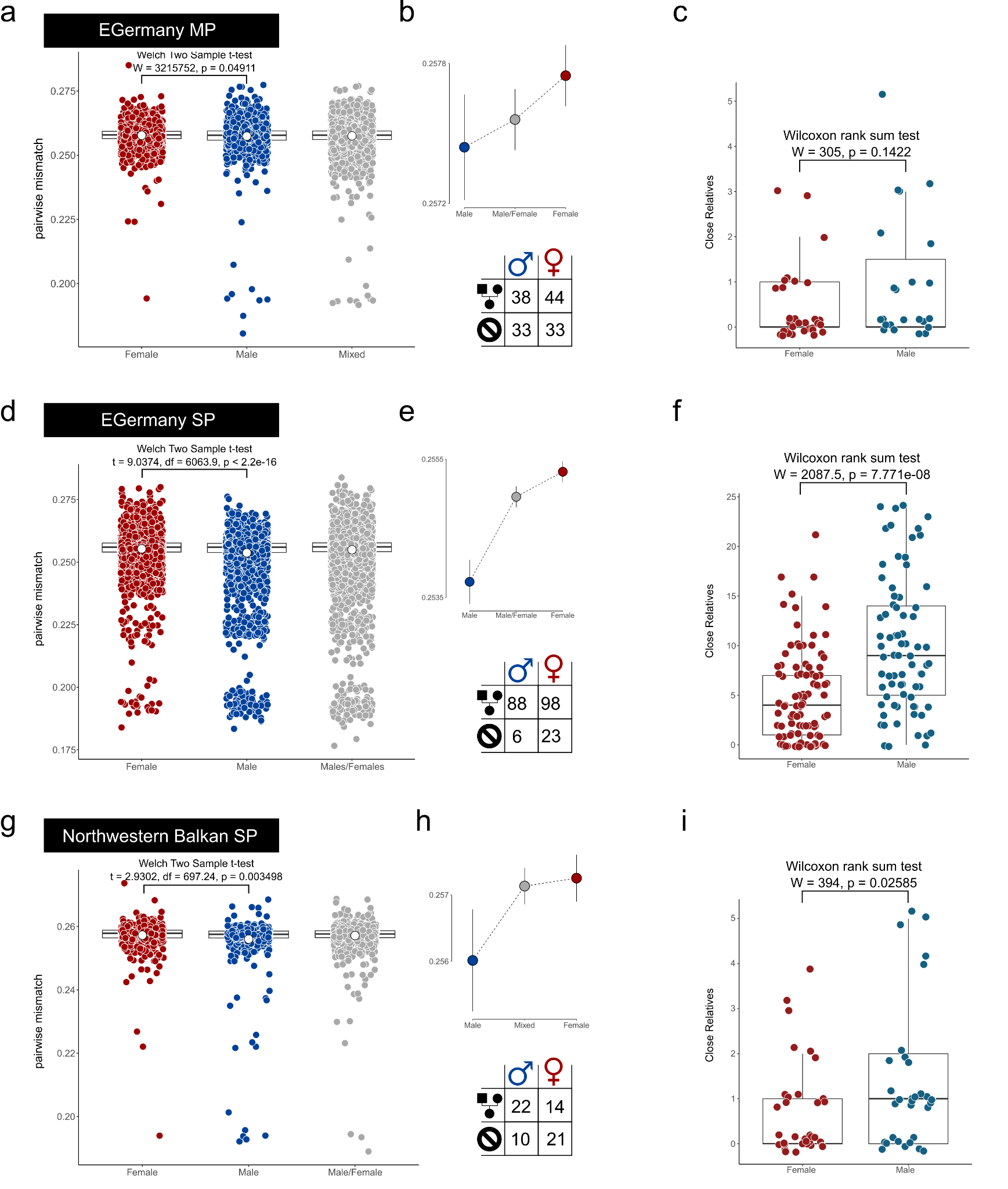


***Supp. Fig. 67. Differences in relatedness patterns between males and females****. Data is shown for MP Eastern Germany (a-c) (n_m_ = 71, n_f_ = 77), SP Eastern Germany (d-f) (n_m_ = 94, n_f_ = 121), and SP Velim, Croatia (g-i) (n_m_ = 32, n_f_ = 35). a) Data from Brücken (BRC), Deersheim (DRH), Obermöllern (OBM), and Rathewitz (RTW), Eastern Germany. Shown are boxplots of pairwise mismatch rates between pairs of female (red), male (blue) and mixed/male-female (grey) individuals. The results of a two-sided Wilcoxon rank sum exact test between the male-pairs and female-pairs group are indicated above the boxplots. b) Top: Average pairwise mismatch rates for pairs of male (blue), female (red), and mixed/male-female (grey) individuals. Error bars indicate two standard errors. Bottom: The contingency table used for Fisher's exact test, summarizing the total count of males and females with and without any close relatives (≤ 3^rd^ degree) at the site. c) Boxplots of the total number of close relatives (≤ 3^rd^ degree) identified at the site for each male (blue) and female (red). The results of a two-sided Wilcoxon rank sum exact test between the male and female group are indicated above the boxplots.*

*
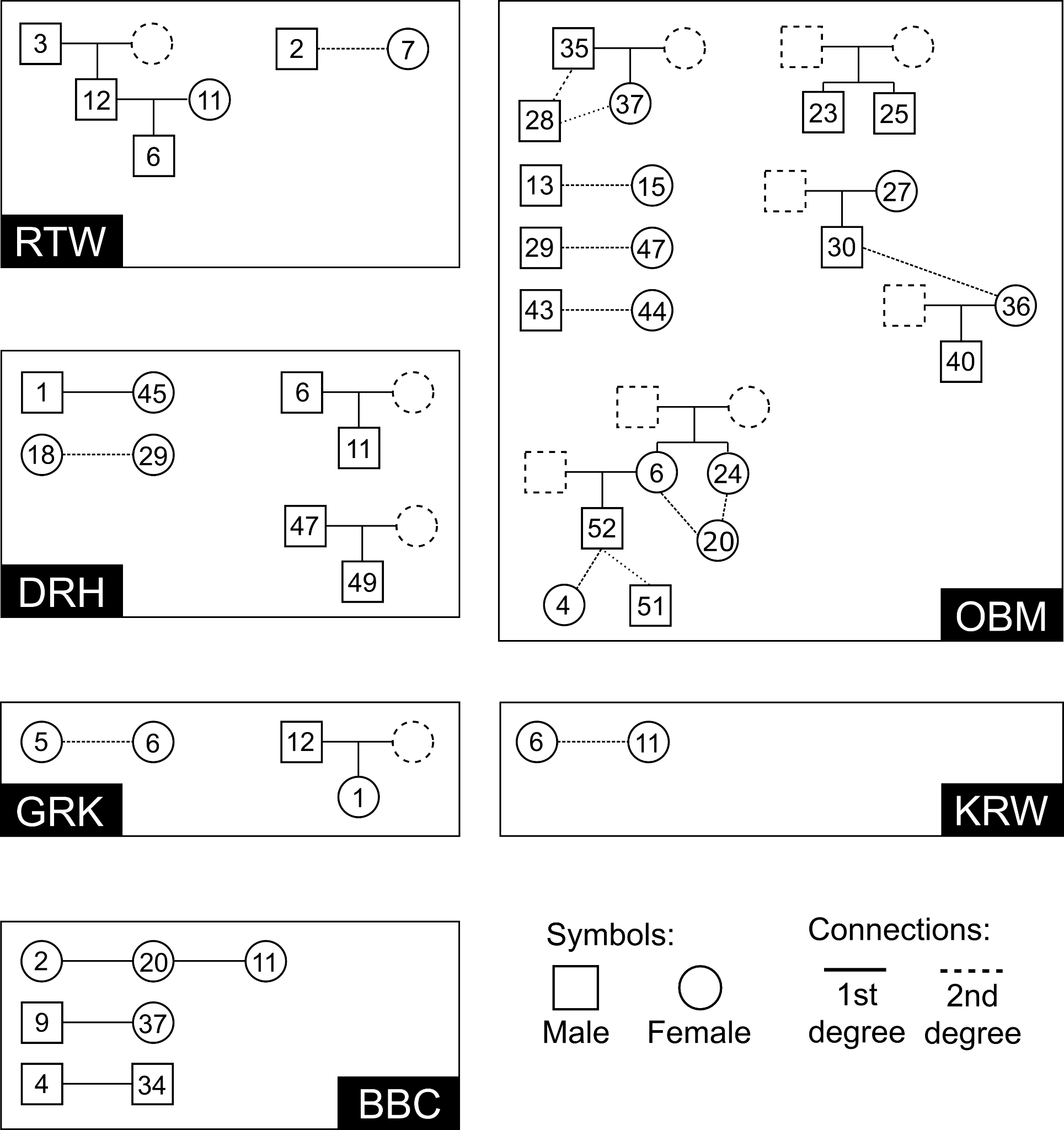
*

***Supp. Fig. 68. Additional pedigrees from MP and SP sites****. Reconstructed pedigrees from the sites Rathewitz (RTW), Obermöllern (OBM), Deersheim (DRH), Gródek (GRK) and Korolivka/Korolówka (KRW) for which no complementary spatial or anthropological data was available. Numbers correspond to the Genetic IDs listed in Supplementary Table 1.*

## 9. Debates about the emergence of Slavic language and languages

The emergence of the Slavic languages and of their common ancestor, Common Slavic, has been debated for a long time[^31,188^](https://paperpile.com/c/UPmHk7/ZtbAn+g8tSj). Apart from some words and names already recorded since the 5^th^/6^th^ centuries, the first transmissions of longer Slavic texts come in late-9^th^/10^th^ century manuscripts, mostly but not exclusively featuring religious content. Almost all of them, save for the so-called Freising Documents (written in Carolingian minuscule), are associated with the tradition founded by late-9^th^ century missionaries Constantine/Cyril and Methodius and originally based on an East South Slavic dialect spoken in the vicinity of Thessaloniki. This first written language of the Slavs, influenced strongly by Greek literary models as well as by the already diverging Slavic vernaculars, is conventionally called Old Church Slavonic and was initially written in the specially created Glagolitic script. Some of the earliest extant manuscripts include the Kiev Leaves and the Prague Fragments[^189^](https://paperpile.com/c/UPmHk7/6zmQp). Also highly significant for early Slavic literacy are the lives of Consantine/Cyril and Methodius, allegedly written around the same time but transmitted only in 15^th^ century East Slavic (“Rus’ian”) manuscripts, in a local, later recension of Church Slavonic[^190^](https://paperpile.com/c/UPmHk7/HG8iB). There are West, but also South Slavic elements in them, so they may have been written by a pupil of Methodius. However, recent research by the editor of the text, Thomas Daiber, has made it plausible that the transmitted Slavic version is a translation from a Greek original[^190^](https://paperpile.com/c/UPmHk7/HG8iB). This result would mean that the literate language, Old Church Slavonic, may have been influenced even more by Greek than previously assumed. The highly mobile group that developed a literate Slavic language moved between Byzantium, Rome, Moravia, Pannonia and Bulgaria, and the various Western and Southern Slavic elements in the text may reflect their attempts to cater to a variety of audiences. Further linguistic research can clarify what that means for linguistic theories about the emergence of separate Slavic languages.

Since the 19^th^ century, historical linguists have used these texts – as well as data from later-attested Slavic languages, their dialects, etc. – to reconstruct the language they called Common Slavic, i.e. the hypothetical ancestor of the medieval and modern Slavic languages[^191^](https://paperpile.com/c/UPmHk7/SWHv9). They also employed linguistic methods to arrive at common ancestors of words, sounds and grammatical features later documented in writing. Common Slavic can in turn be classified as an Indo-European language, together with, e.g., the Romance and Germanic languages, Greek, Sanskrit or Persian; however, the reconstruction of the ancestral Proto-Indo-European language in any detail is projected much further into the past and is even more hypothetical. The Indo-European branch closest to Slavic are the Baltic languages (Lithuanian, Latvian, and the extinct Old Prussian). What they looked like in the time when the Slavic languages emerged is difficult to project, given that the earliest Lithuanian texts are Catholic Catechisms printed in the 16^th^ century. Nowadays, most linguists agree that there ever was a common Balto-Slavic branch. Yet, some scholars propose that the similarities between these languages are due to many centuries of neighborhood.

A further spiny question is the debate about the supposed primordial homeland of the Slavs; arguments have even drawn from toponyms (thought to preserve archaic name forms) or the most ancient Slavic terminology for trees and plants (as clues to their most likely habitats)[^192^](https://paperpile.com/c/UPmHk7/r8yOD). These endeavors remained as hypothetical as they were controversial. They also provoked the question whether there had ever been a primordial Slavic homeland. Many scholars assume “the territory north-east of the Carpathians including the middle part of the Dnjepr basin as the ethnic and linguistic center of the Slavs”[^193^](https://paperpile.com/c/UPmHk7/xCenZ).

A third controversial question in Slavic linguistics is the emergence of the particular Slavic languages as they exist nowadays. What most linguists found striking was that even today’s languages are quite similar to each other in many ways, and the split probably happened only after the arrival in their medieval and modern settlement areas[^194^](https://paperpile.com/c/UPmHk7/XpbAW). As is generally assumed nowadays, the erosion of Common Slavic linguistic unity was a long process. How we distinguish a dialect within a language and a language of its own is a matter of definition. Mainstream opinion is that the Slavic family is divided into the groups of West, South and East Slavic, which can be broken up into further subgroups and languages. Old Church Slavonic, as a literary language from its inception, was a separate and eclectic entity, though primarily relying on a South Slavic basis (see above). A principal linguistic method to trace the process of the breakup of Common Slavic is by analyzing borrowings from the languages of neighboring peoples. For instance, borrowings from German can be dated by the two Germanic sound shifts of the Early Middle Ages. These then allow some indications as to when speakers of the respective Slavic languages may have been in contact with German/ic speakers[^191^](https://paperpile.com/c/UPmHk7/SWHv9).

Recently, some scholars employ Bayesian calculations in phylogenetic linguistics to date the emergence of Indo-European languages, Slavic among them; for instance, in a recent article by Heggarty et al. in Science 2023[^146^](https://paperpile.com/c/UPmHk7/emO5R). This approach, and the exact methodological procedure, require further debate. The analysis is based on a relatively small section of lexical terms subjected to reductive encoding, and excludes the effects of language contact. Dating is insecure and built on what is called a “relaxed clock” method, which in fact means a chronology of lexical change chosen in what may risk becoming a circular argument. It is debatable whether the scarce evidence one has about the development of early Slavic provides enough reliable data to construct such a model. After all, this means building algorithms on linguistic hypotheses about which there is no consensus among linguists. In short, the resulting dates for the diffusion of Slavic can hardly be used as a prop for archaeological or historical dating of migrations, especially if these have already been used to calibrate the “relaxed clock”. However, as long as we regard the phylogenetic chronology as a hypothesis it remains a valuable contribution.

It should also be noted that even where one has plausible linguistic dates about the arrival of Slavic speakers, we cannot be sure whether all Slavic speakers were regarded or regarded themselves as Slavs, and whether all Slavs spoke Slavic[^2^](https://paperpile.com/c/UPmHk7/QYHrb). One hypothesis maintains that Slavic was used, and spread across Eastern Europe as a *lingua franca*[*^194^*](https://paperpile.com/c/UPmHk7/XpbAW). Some scholars also assume that the late Avars may have spoken Slavic, because there is a lack of Turko-Mongolic place names in the Carpathian Basin. The Bulgars at some point also adopted the Slavic language from their Slavic subjects. Non-Slavs who became Slavicized may have embraced Slavic identity before or after Slavic language. Some of those that we class as Slavs on good grounds may have felt they belonged to Sorbs, Moravians or Carantanians rather than Slavs. So not everyone who spoke Slavic necessarily identified as a Slav, and vice versa. It is generally plausible to assume that Slavs spoke Slavic, but the evidence is too elusive to prove it in individual communities. As Tomáš Klír argued, “the question of the relations between Slavic language, ethnicity, and material culture has differing answers in different geographical and political contexts”[^31^](https://paperpile.com/c/UPmHk7/g8tSj).

## References

1. [Curta, F. *The Making of the Slavs: History and Archaeology of the Lower Danube Region, C. 500-700*. vol. 52 (Cambridge University Press, Cambridge, 2001).](http://paperpile.com/b/UPmHk7/GOPpA)

2. [Curta, F. *Slavs in the Making History, Linguistics, and Archaeology in Eastern Europe (ca. 500 – Ca. 700)*. (Routledge, London, 2021).](http://paperpile.com/b/UPmHk7/QYHrb)

3. [Gnecchi-Ruscone, G. A. *et al.* Network of large pedigrees reveals social practices of Avar communities. *Nature* **629**, 376–383 (2024).](http://paperpile.com/b/UPmHk7/RIikQ)

4. [Jones, A. H. M. *The Later Roman Empire, 284-602*. (Johns Hokins, Baltimore, 1964).](http://paperpile.com/b/UPmHk7/FRUJk)

5. [*Late Antiquity: Empire and Successors, A.D. 423-600*. (Harvard University Press, Cambridge, 2000).](http://paperpile.com/b/UPmHk7/Wv71f)

6. [James, E. *Europe’s Barbarians, AD 200-600*. (Pearson, Harlow, 2008).](http://paperpile.com/b/UPmHk7/szD37)

7. [Woolf, A. *Tales of the Barbarians. Ethnography and Empire in the Roman West*. (Wiley Blackwell, Malden & Oxford, 2014).](http://paperpile.com/b/UPmHk7/b8FhQ)

8. [Pohl, W. & Steinacher, R. Wenden, Slawen, Vandalen. Eine frühmittelalterliche pseudologische Gleichsetzung und ihr Nachleben bis ins 18. Jahrhundert. in *Auf der Suche nach den Ursprüngen. Von der Bedeutung des frühen Mittelalters* (ed. Pohl, W.) 329–353 (Verlag des ÖAW, Vienna, 2004).](http://paperpile.com/b/UPmHk7/FyEkC)

9. [Heather, P. *The Fall of the Roman Empire: A New History of Rome and the Barbarians*. (Oxford University Press, Oxford, 2005).](http://paperpile.com/b/UPmHk7/KFxeB)

10. [Pohl, W. *The Avars: A Steppe Empire in Central Europe, 567–822*. (Cornell University Press, Ithaca, 2018).](http://paperpile.com/b/UPmHk7/y6eI6)

11. [Halsall, G. *Barbarian Migrations and the Roman West, 376–568*. (Cambridge University Press, Cambridge , 2007).](http://paperpile.com/b/UPmHk7/uH0Ma)

12. [Pohl, W. *Die Völkerwanderung. Eroberung Und Integration*. (Kohlhammer, Stuttgart , 2002).](http://paperpile.com/b/UPmHk7/M6G6C)

13. [Pohl, W. *Die Germanen*. (De Gruyter, Oldenbourg, 2000).](http://paperpile.com/b/UPmHk7/RwPWt)

14. [Wolfram, H. *The Roman Empire and Its Germanic Peoples*. (University of California Press, Berkeley, 1997).](http://paperpile.com/b/UPmHk7/Y045i)

15. [Meier, M. *Geschichte Der Völkerwanderung: Europa, Asien Und Afrika Vom 3. Bis Zum 8. Jahrhundert n.Chr*. (C.H.Beck, München, 2019).](http://paperpile.com/b/UPmHk7/ZMgez)

16. [Schmidt, B. *Die Späte Völkerwanderungszeit in Mitteldeutschland. Katalog (Nord- Und Ostteil)*. vol. 29 (M. Niemeyer, Halle (Saale) , 1976).](http://paperpile.com/b/UPmHk7/3XRJl)

17. [Mildenberger, G. *Sozial- Und Kulturgeschichte Der Germanen von Den Anfängen Bis Zur Völkerwanderungszeit*. (Urban, Stuttgart, 1972).](http://paperpile.com/b/UPmHk7/jH63f)

18. [Steinacher, R. *Rom Und Die Barbaren. Völker Im Alpen- Und Donauraum (300-600)*. (Kohlhammer, Stuttgart, 2017).](http://paperpile.com/b/UPmHk7/n8NGj)

19. [*The Baiuvarii and Thuringi. An Ethnographic Perspective*. (Boydell, Woodbridge, 2014).](http://paperpile.com/b/UPmHk7/aikft)

20. [Wood, I. N. The frontiers of Western Europe: Developments east of the Rhine in the sixth century. in *The Sixth Century. Production, Distribution and Demand* (eds. Hodges, R. & Bowden, W.) 231–253 (Brill, Leiden, 1998).](http://paperpile.com/b/UPmHk7/1KaGe)

21. [Pohl, W. Historiography and Identity – Methodological Perspectives. in *Historiography and Identity 1: Ancient and Early Christian Narratives of Community* (eds. Pohl, W. & Wieser, V.) 7–50 (Turnhout, Brepols, 2019).](http://paperpile.com/b/UPmHk7/7USVJ)

22. [*Jordanes, Getica. MGH AA 5,1*. (Weidmann, Berlin, 1882).](http://paperpile.com/b/UPmHk7/n61Do)

23. [*Procopius, Bella*. (Harvard University Press, Cambridge, 1979).](http://paperpile.com/b/UPmHk7/l81UN)

24. [Strategikon. *Maurice’s Strategikon. Handbook of Byzantine Military Strategy*. (Verlag der ÖAW, Vienna, 1984).](http://paperpile.com/b/UPmHk7/r1yUA)

25. [Dzino, D. *Becoming Slav, Becoming Croat: Identity Transformations in Post-Romanand Early Medieval Dalmatia, East Central and Eastern Europe in the Middle Ages,450–1450*. (Brill, London, 2010).](http://paperpile.com/b/UPmHk7/x791X)

26. [Simocatta, T. *The History of Theophylact Simocatta, Trans. by Whitby, M*. (Oxford University Press, Oxford, 1986).](http://paperpile.com/b/UPmHk7/BJR5h)

27. [Wołoszyn, M. *Theophylaktos Simokates Und Die Slawen Am Ende Des Westlichen Ozeans - Die Erste Erwähnung Der Ostseeslawen? : Zum Bild Der Slawen in Der Frühbyzantinischen Literatur : Eine Fallstudie. Teofilakt Simokatta I Słowianie Znad Brzegu Oceanu Zachodniego - Najstarsze świadectwo Obecności Słowian Nad Bałtykiem?* (Instytut Archeologii i Etnologii Polskiej Akademii Nauk, Kraków, 2014).](http://paperpile.com/b/UPmHk7/2WxCK)

28. [Barford, P. M. *The Early Slavs: Culture and Society in Early Medieval Eastern Europe*. (Cornell University Press, Ithaca, 2001).](http://paperpile.com/b/UPmHk7/ES1FT)

29. [Gleirscher, P. *Karantanien: Slawisches Fürstentum Und Bairische Grafschaft*. (Hermagoras, Klagenfurt, 2018).](http://paperpile.com/b/UPmHk7/AJsxv)

30. [Mühle, E. *Die Slawen Im Mittelalter Zwischen Idee Und Wirklichkeit*. (Böhlau, Cologne, 2020).](http://paperpile.com/b/UPmHk7/iTVIC)

31. [*New Perspectives on the Early Slavs and the Rise of Slavic. Contact and Migrations*. (Universitätsverlag Winter, Heidelberg, 2020).](http://paperpile.com/b/UPmHk7/g8tSj)

32. [Wenskus, R. *Stammesbildung Und Verfassung. Das Werden Der Frühmittelalterlichen Gentes*. (Böhlau, Vienna, 1977).](http://paperpile.com/b/UPmHk7/SJ7Sd)

33. [Pohl, W. Debating Ethnicity in Post-Roman Historiography. in *Historiography & Identity 2: Post-Roman Multiplicity and New Political Identities* (eds. Heydemann, G. & Reimitz, H.) 27–70 (Turnhout, Brepols, 2020).](http://paperpile.com/b/UPmHk7/HNqRw)

34. [Urbańczyk, P. Słowianie byli „produktem” przypadku historycznego!? Slavs as A ‘product’ of A historical coincidence!? *Archeologia Polski* **68**, 285–315 (2023).](http://paperpile.com/b/UPmHk7/ryq1F)

35. [Kazanski, M. Archaeology of the Slavic Migrations. in *Encyclopedia of Slavic Languages and Linguistics Online* (ed. Greenberg, M. L.) (Brill, London, 2020).](http://paperpile.com/b/UPmHk7/qtBso)

36. [Szmoniewski, B. Ethnogenesis of Slavs Viewed from Polish Perspective. *Soka University Bulletin of Russian and Slavic Studies* **12**, 23–43 (2020).](http://paperpile.com/b/UPmHk7/IrXn3)

37. [Dulinicz, M. *Frühe Slawen Im Gebiet Zwischen Unterer Weichsel Und Elbe. Eine Archäologische Studie*. (Wachholtz, Neumünster, 2006).](http://paperpile.com/b/UPmHk7/myi8o)

38. [Kazanski, M. Ukraine, archäologisch. in *Reallexikon der germanischen Altertumskunde* vol. 31 373–391 (Walter de Gruyte, Berlin, 2006).](http://paperpile.com/b/UPmHk7/prnsU)

39. [*The Early Slavic Settlement in Central Europe in the Light of New Dating Evidence*. vol. 3 (Institute of Archaeology and Ethnology of the Polish Academy of Sciences, Wrocław, 2013).](http://paperpile.com/b/UPmHk7/GUKHc)

40. [Biermann, F., Dalitz, S. & Heußner, K.-U. Der Brunnen von Schmerzke, Stadt Brandenburg an der Havel, und die absolute Chronologie der frühslawischen Besiedlung im nordostdeutschen Raum. *Prähistorische Zeitschrift* **74**, 219–243 (1999).](http://paperpile.com/b/UPmHk7/tSbTw)

41. [*Welt Der Slawen. Geschichte, Gesellschaft, Kultur*. (Beck, München, 1986).](http://paperpile.com/b/UPmHk7/PA1Ic)

42. [Brather, S. *Archäologie Der Westlichen Slawen. Siedlung, Wirtschaft Und Gesellschaft Im Früh- Und Hochmittelalterlichen Ostmitteleuropa*. (De Gruyter, Berlin, 2008).](http://paperpile.com/b/UPmHk7/eCGn1)

43. [*Zentralisierungsprozesse Und Herrschaftsbildung Im Frühmittelalterlichen Ostmitteleuropa*. (Habelt, Bonn, 2014).](http://paperpile.com/b/UPmHk7/X3SQx)

44. [*Archeologia O Początkach Słowian. Materiały Z Konferencji, Kraków, 19-21 Listopada 2001*. (Instytut Archeologii Uniwersytetu Jagiellońskiego, Kraków, 2005).](http://paperpile.com/b/UPmHk7/w4KVm)

45. [Ziółkowski, A. Pollen, brooches, solidi and Restgermanen, or today’s Poland in the Migration Period. Review of: A. Bursche, J. Hines, A. Zapolska (eds), The Migration Period between the Oder and the Vistula, East Central and Eastern Europe in the Middle Ages, 450–1450, Leiden/Boston 2020. *Millennium* **19**, 174–196 (2022).](http://paperpile.com/b/UPmHk7/ED8le)

46. [*Kulturwandel in Mitteleuropa: Langobarden, Awaren, Slawen. Akten Der Internationalen Tagung in Bonn Vom 25. Bis 28. Februar 2008*. (Habelt, Bonn, 2008).](http://paperpile.com/b/UPmHk7/zy9lQ)
[truncated: 31,562 more chars]
